# A case of forensic genomics in Uganda reveals animal ownership and low exotic genetic introgression in indigenous cattle

**DOI:** 10.1002/vms3.1272

**Published:** 2023-09-19

**Authors:** Charles Masembe, Kirungi Katali Benda, Oluyinka Opoola, Mayega Johnson Francis, Ruth Pamela Ndinawe, Peter Beine, Robert Mukiibi

**Affiliations:** ^1^ Department of Zoology, Entomology and Fisheries Sciences, College of Natural Sciences Makerere University Kampala Uganda; ^2^ National Animal Genetics Resources Centre and Data Bank (NAGRC&DB) Entebbe Uganda; ^3^ The Roslin Institute and Royal (Dick) School of Veterinary Studies University of Edinburgh Edinburgh UK; ^4^ Centre for Tropical Livestock Genetics and Health (CTLGH) University of Edinburgh Edinburgh UK

**Keywords:** bovine genomics, genetic analysis, genetic diversity, MHC genes, single nucleotide polymorphisms

## Abstract

**Background:**

The cattle industry contributes to Uganda's agricultural output. It faces challenges that include theft and parentage ascertainment. These challenges can benefit from recent molecular genomics and bioinformatics technologies.

**Objectives:**

We employed genomic analyses to establish potential ownership of a group of nine cattle that were being claimed by two farmers in Uganda. We investigated the genetic relationship of Ugandan cattle with regional indigenous breeds as well as exotic breeds that are currently present in Uganda. In addition, we investigated regions that are likely to be under selection in the Ugandan cattle.

**Methods:**

Hair samples were collected from seven and two animals from farmers A and B, respectively. They were genotyped for 53,218 Single Nucleotide Polymorphism markers. To establish genetic relationships between the sampled animals, we performed genomic analyses including, principal component analysis (PCA), hierarchical clustering analysis and identity by state/descent. We also performed admixture and runs of homozygosity analyses to assess the ancestry composition and identify regions potentially under selection in Ugandan cattle, respectively.

**Results:**

The seven animals from Farmer A were genetically close to each other but showed minimal relationship with the disputed animals. The two animals from Farmer B were genetically distant from each other but showed greater similarity to four of the disputed animals. Four of the disputed animals showed great dissimilarity from the animals of both farmers. Comparison of these with the reference breeds revealed minimal European exotic genetic introgression into these animals, but rather high similarity to the Sheko. Results also revealed high homozygosity in the major histocompatibility complex regions.

**Conclusions:**

Our results demonstrate the use of currently available genomic tools to empirically establish the ownership of cattle; these could be scaled up as a resourceful and viable tool that could be employed to support conflict resolution where reliable livestock identification is unavailable.

## Graphical abstract

The cattle industry contributes significantly to agricultural output, although it faces challenges that include theft and parentage ascertainment. We used Single Nucleotide Polymorphism genomic analyses to establish potential ownership of nine cattle that were being claimed by two farmers in Uganda. Seven animals from Farmer A were genetically close to each other but showed minimal relationship with the disputed animals, while two animals from Farmer B were genetically distant from each other but showed greater similarity to the disputed animals.

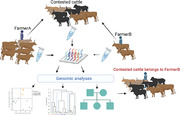

## 1 INTRODUCTION

The livestock production industry is an important component of the Ugandan economy contributing up to 3.8% of the national annual gross domestic product (GDP; Uganda Bureau of Statistics, 2020). Uganda has a population of about 14.8 M heads of cattle, most (94%) of which are from indigenous breeds (Uganda Bureau of Statistics, 2020). In addition to providing employment to Ugandans, the cattle production industry produces economically valuable products such as milk, beef and hides for both local and foreign markets (Chauvin et al., 2017; Mbabazi & Ahmed, 2019; Uganda Bureau of Statistics, 2020; Waiswa et al., 2021). Like the rest of the world, Uganda has a rapidly increasing population size coupled with increasing per capita income, especially in the urban areas, which has consequently increased the demand for livestock products locally (Mottaleb et al., 2021). This local and growing foreign demand for Uganda's animal products has attracted several farmers into extensive and intensive livestock production (Bingi & Tondel, 2015), and rearing large numbers of animals. The increased value of cattle, coupled with poor farm fencing infrastructures, limited cattle branding, ear tagging, and reliable record keeping especially for those farmers with large herds, usually leads to animal theft and creates conflicts between farmers over animals whose ownership the farmers cannot prove or defend with precision.

Recent technological advances in genomics avail us with numerous reliable tools, some of which can be used to empirically solve such puzzles or disputes as well as parentage assignments. For example, as few as 50 single nucleotide polymorphisms (SNPs) can be used to determine parentage in cattle (Bovine HapMap Consortium et al., 2009). Parentage or kinship assignment helps in managing inbreeding in closed populations, and pedigree reconstruction which is needed in genetic merit estimation for selective breeding (Panetto et al., 2017; Vandeputte & Haffray, 2014). Additionally, genome‐wide markers have also been utilised in genomic selection to improve the productivity of different livestock species (Meuwissen et al., 2013). In Uganda, SNPs and microsatellite markers distributed all over the genome have been used for the genetic characterisation of different livestock species populations including cattle (Kugonza et al., 2011), goats (Onzima et al., 2018a, 2018b) and pigs (Babigumira et al., 2021). These population genetic structure and composition studies are based on the expectation that individuals sharing recent common ancestry show a higher degree of genome similarity between them, as compared to distantly related individuals. It is upon this hypothesis that we designed the current study with the main objective to employ genomic analyses to determine ownership of a group of cattle, which were being claimed by two cattle herd owners. Additionally, we investigated the genetic structure and composition or breed admixture of the studied Ugandan cattle via genome‐wide DNA marker genotype comparative analyses with reference animals from other African breeds and exotic European breeds that are commonly used for crossbreeding in Uganda. We also investigated the genomic regions that are potentially under artificial or natural selection in Uganda cattle via runs of homozygosity analysis for the studied animals.

## 2 MATERIALS AND METHODS

**FIGURE 1:**
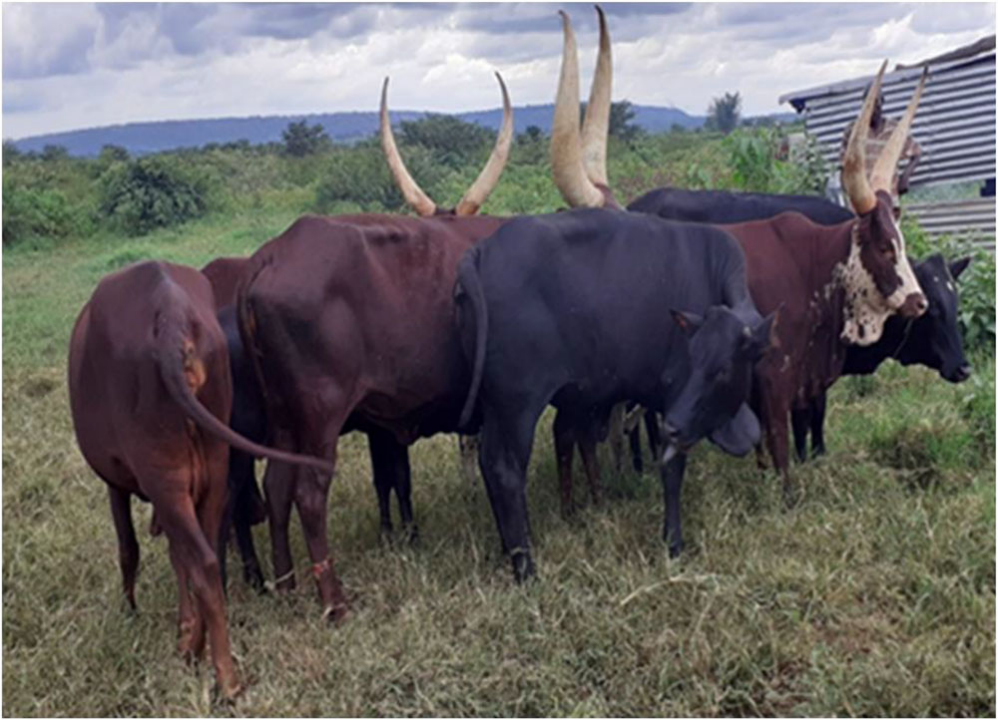
Picture of some of the contested animals investigated in this study.

This is a pilot forensic study involving the application of genomics to the cattle industry in Uganda. In the month of November 2019, hair samples were collected from 18 cattle from three groups of animals in Central Uganda (Figure 1). These animals included nine animals (C1, C2, C3, C4, C5, C6, C7, C8 and C9) whose ownership was being contested by two claimant farmers and we refer to them as contested animals, seven (A1, A2, A3, A4, A5, A6 and A7) and two (B1 and B2) animals were from the farmers (we referred to as Farmer A animals and Farmer B animals, respectively) claiming ownership of the nine animals as reference relative animals to the contested animals. All animals had neither records nor unique tags or morphological identifiers. These cattle included males (*n* = 3) and females (*n* = 15), with varying horn morphology and coat colour as described in the Table S1 in Supplementary File 1. Hair samples from each animal were individually labelled and bagged at collection, and then stored at −20°C until shipping for DNA extraction and subsequent genotyping. DNA extraction and genotyping was performed by *Macrogen Inc*. (Seoul, South Korea http://dna.macrogen.com/). DNA sample of each animal was individually genotyped for 53,218 Single Nucleotide Polymorphism (SNP) markers using the commercially available Illumina BovineSNP50‐24 version 3 BeadChip (Matukumalli et al., 2009). This genotype data has been deposited to Dryad an open‐access research data repository (https://doi.org/10.5061/dryad.fj6q57410). Quality control was performed on the genotype data using PLINK (Purcell et al., 2007) version 1.9 (Purcell et al., 2007) first by checking whether the individual sample's recorded sex (at sampling) matched the individual's sex inferred from the SNP genotype data using the *–check‐sex* flag. Subsequently, we removed mitochondrial (*n* = 13) and sex chromosome SNP markers (*n* = 1168), markers that were not assigned to any chromosome (*n* = 759), markers that did not show substantial variability between study individuals (–maf 0.05, *n* = 15,992), those that showed significant deviation from Hardy–Weinberg equilibrium (–hardy –hwe 1E‐4, *n* = 0), and those whose genotyping rate was less than 95% (–geno 0.05, *n* = 1270). Using the cleaned genotype data (34,016 SNPs), PLINK (Purcell et al., 2007) was further used to perform principal component analysis (PCA) for the genotyped animals. The first two principal components were then used to visualise genetic distribution between the studied animals in R version 4.0.3 (R Core Team, 2013). We also visualised the PCA results in the form of a dendrogram using the R package vegan version 2.6‐4 (Oksanen et al., 2022). Additionally, to investigate recent shared between animals between and within the three subgroups we used PLINK (Purcell et al., 2007) to perform genome‐wide identity by state (IBS) or identity by descent (IBD) estimation using the –genome flag.

To investigate the potential genetic relationship between the studied animals with other cattle breeds some of which have history of being used for crossing with Ugandan local cattle, we further performed PCA using PLINK (Purcell et al., 2007), and admixture analysis using admixture software version 1.3.0 (Alexander et al., 2009) for the studied animals and animals from reference breeds. The reference breeds used included N'Dama (*n* = 24), Brahman (*n* = 49), Sheko (*n* = 18), Jersey (*n* = 42), Holstein (*n* = 67) and Angus (n = 47). Genotype data and breed description information for the reference animals were obtained from publicly available data from the Bovine HapMap project (Bovine HapMap Consortium et al., 2009). We further investigated the potential genomic regions in Ugandan animals that have undergone natural or human‐directed selection through runs of homozygosity (ROH) analysis using PLINK (Purcell et al., 2007) following the ROH analysis in livestock species guidelines from Meyermans et al. (2020). Parameters used in this analysis included the following: scanning window of 40 SNPs across the genome (*–homozyg‐window‐snp 40*), a maximum of one heterozygous SNP allowed in the window (–*homozyg‐window‐het 1*), a maximum of five SNP with missing genotypes allowed in the window (*–homozyg‐window‐missing 5*), a scanning window hit rate threshold of 0.05 (*–homozyg‐window‐threshold 0.05*), maximum gap between two homozygous SNPs of 500 kb (*–homozyg‐gap 500*), a minimum density of one SNP per 60 kb in the homozygous segment (*–homozyg‐density 60*), minimum length of the ROH of 1000 kb (*–homozyg‐kb 1000*) and a minimum number of 100 consecutive SNPs per the ROH (*–homozyg‐snp 100*). The Ensembl biomart tool (http://www.ensembl.org/biomart/martview) was used to obtain genes located within the identified ROH regions from the ARS‐UCD1.2 bovine reference genome hosted by Ensembl genome browser (105 release). To elucidate on the collective biological functions and processes of the genes located in the identified ROH regions, functional enrichment analysis was further performed in Panther classification system (Mi et al., 2021) using Ensembl stable gene IDs of the candidate genes in the ROH as the input gene list and *bos taurus* as the target species.

## 3 RESULTS AND DISCUSSION

The contested animals in the current study showed variability in terms of coat colour with five of the nine animals having predominantly black coat and four being predominantly brown. Six of these animals were females, and three were males. In terms of age, five of the animals were adult cattle, while four were juveniles or yearlings. The horn morphology of the animals varied with three animals being long‐horned, three polled one short‐horned and one had lateral short horns. The animals from Farmer A were all predominantly brown, of which six of the seven animals were adult females. The animals were mostly (six of seven) long‐horned with only one animal having short lateral horns. The animals from Farmer B were predominantly brown, adult females of which one was polled and the other long‐horned. The detailed morphological descriptions of the animals used in this study are provided in Table S1 in Supplementary File 1. Due to this variability in morphological features between the contested animals and animals from both farmers, as well as lack of defined or reliable animal identification on the farms of both farmers, it was difficult to empirically infer the ownership of the contested animals based on the morphological features. Therefore, we considered utilisation of genomics as a more objective methodology to infer ownership of these animals. It is worth highlighting that this is the first study in Uganda and, on the African continent, to demonstrate viability of genomics as tools to settle conflicts in the livestock sector. Our study is also the first one to employ genome‐wide marker genotype analyses to characterise Ugandan indigenous cattle.

**FIGURE 2:**
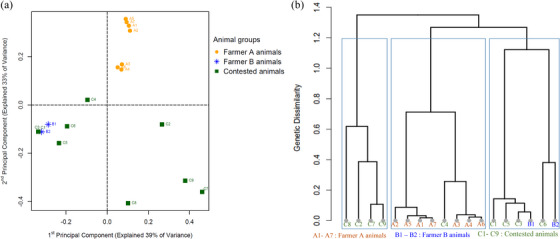
(a) Principal component plot showing genetic distribution and clustering of the investigated animals. (b) A dendrogram hierarchical plot showing the genetic similarity or dissimilarity among the studied animals.

We obtained high‐quality genotype data with a genotyping call rate of 99.6% per sample and an average minor allele frequency of 0.18 ± 0.16. For all the 18 genotyped cattle in the current study, the sex inferred from SNP genotype data corresponded with the sex recorded at sampling or pedigree sex (Table S2 in Supplementary File 2). Genetic analysis revealed three genetic groups of the animals involved in the study as shown in Figure 2a and b. The 1st and 2nd principal components together explained 72% of the genomic variation among the investigated animals, and visualisation of the animals by these two dimensions showed that all the animals from Farmer A (A1–A7) clustered together under two subclusters, and under these subgroups, animals were genetically close to each other (Figure 2a and b). The animals from Farmer B (B1 and B2) also clustered together though with some appreciable genetic distance from each other (Figure 2a and b). The contested animals showed two genetic groups, with some of the animals showing close genetic relationships with Farmer B's animals, and others forming an independent group where individuals were sparsely distributed (Figure 2a and b). Four of the contested animals (C8, C2, C7 and C9) clustered together but with low genetic similarity within the cluster; the other four (C1, C3, C5 and C6) clustered with Farmer B's animals where C1, C3 and C5 showed close genetic similarity to animal B1 (Figure 2a and b). Animal C4 clustered with Farmer A's animals, but the animals it subclustered with were more similar to each other than to C4.

In agreement to these results, our IBD analyses also revealed very low IBD relationship (0–0.027) between the contested animals and Farmer A's animals ranging between 0 to 0.025, while the former showed predominantly high IBD relationships (0–0.5) with Farmer B's animals (Table S3 in Supplementary File 3). It is worth highlighting that six of the contested animals (C1, C3, C4, C5, C6 and C8) show evidence for at least 3rd degree relatedness to B animals (IBD proportion higher than 0.125), while all contested animals are essentially unrelated to A animals (IBD proportion lower than 0.027). For the C4 animal whose ownership between the two farmers was difficult to establish from genetic clustering analyses, IBS/IBD estimation demonstrated that this animal had a greater genetic connection with B animals (IBD proportion of 0.1 and 0.18) than with the A animals (IBD proportion ranging between 0 and 0.025). In addition, the IBD relationship between animal C6 and B2 was 0.5 which translates into an offspring–parent genetic relationship between these two animals; this concurred with the claim by Farmer B who at the time of selecting animals related to the contested animals identified B2 as the mother of C6. All these genetic analysis results concurred with the information obtained about the breeding and production practices by the two farmers. Farmer B is majorly a cattle trader whose herd is composed of animals purchased from different markets and farms, hence a genetically diverse herd. However, Farmer A is a closed cattle breeder who practices a closed on‐farm mating or breeding system, with little or no external genetics imported into his herd, hence a genetically very similar or related herd.

The results from these genomic analyses demonstrate that indeed all the contested animals are most likely to belong to Farmer B than Farmer A. It is worth highlighting that livestock management systems in Africa especially cattle are predominantly characterised by transhumance and migratory movement to livestock production zones and grazing locations during the dry season in the search of lustrous pasture and water (Motta et al., 2018). As a result, some of the cattle might be lost or stolen during these migrations into other cattle populations with little or no information recorded on migratory movement and grazing locations. Therefore, cattle farmers are unable to keep adequate records of their cattle head number counts and identification, which limits our insights on production, herd health, management practices and cattle ownership in many parts of Africa. With this lack of identification, it is also difficult to ascertain ownership of lost or stolen cattle in such migratory activities, resulting into within countries and between countries conflicts. Indeed, our study demonstrates how such conflicts can be empirically resolved through genomic analyses.

**FIGURE 3:**
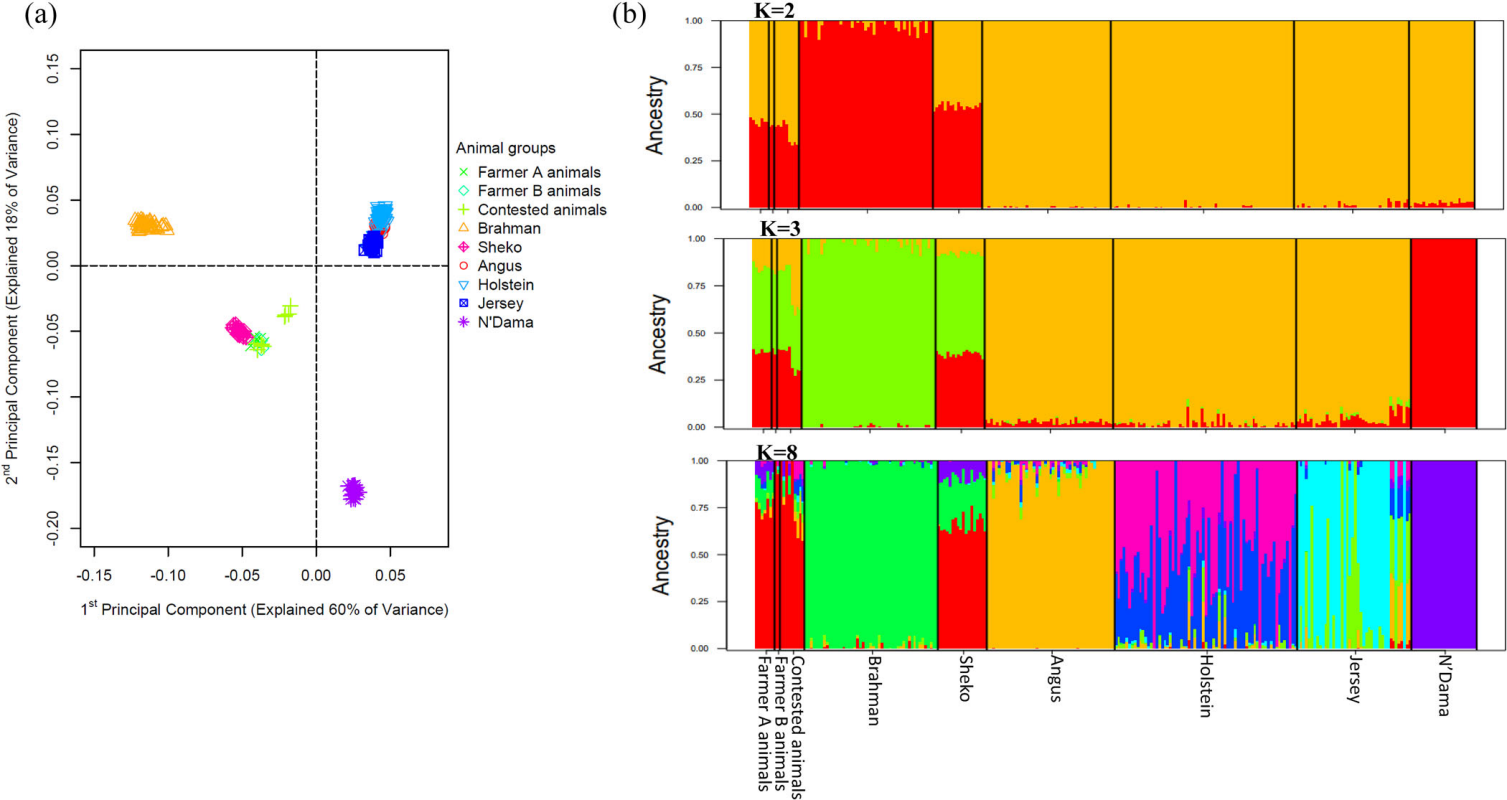
(a) Principal component plot showing genetic distribution and clustering of the investigated animals and reference animals of the Bovine HapMap project. (b) Admixture bar plots showing each individual animal's (investigated and reference) genomic composition when we assumed two (K = 2), three (K = 3) and 8 (K = 8) ancestral populations.

Principal component analysis of the investigated animals with the reference animals revealed genetic distinction of the studied animals from most of the reference breeds including those that are most commonly used for crossbreeding in the Ugandan cattle production industry such as Holstein, Jersey and Angus (Figure 3a). However, we observed close genetic relationship between the investigated animals and the reference animals from the Sheko breed, although few (*n* = 4) of the contested animals were distant from the rest of the sampled animals and Sheko animals. For the admixture analyses, when we assumed only two ancestral populations (*K* = 2), the results showed that the investigated animals had both taurine and indicine proportions similar to that of the Sheko breed reference animals. Results with higher presumed ancestral populations demonstrated further that the investigated animals shared great ancestry (79.5 ± 3.26% at *K* = 8) with Sheko animals, as compared to other reference breeds (Figure 3b). Sheko is a taurine × zebu East African indigenous breed historically from southwestern Ethiopia (Bahbahani et al., 2018; Hassen et al., 2007), and shares ancestry with Ankole cattle (Makina et al., 2016). The Ankole cattle has also been described as an intermediate between zebu and taurine cattle (Kim et al., 2017; Taye et al., 2017). Close genetic relationship between Ankole and Sheko cattle has been previously observed in several studies (Alshawi et al., 2019; Makina et al., 2016; Mekonnen et al., 2019). Most of the cattle raised in Uganda are Ankole cattle (Kugonza et al., 2011), characterised with their large and long horns (Kugonza et al., 2011). Indeed, most of the animals sampled had long curving large horns and were described by the farmers as Ankole cattle (Figure 1). The high genetic similarity of these Ugandan animals with Sheko despite indiscriminate crossbreeding in the country (Kugonza et al., 2011; Ndumu et al., 2008), demonstrates that Ugandan's local animals still retain a significant level of indigenous ancestry in spite of the crossbreeding in the country. It is this indigenous ancestry that confers adaptation and resilience to the different local environment challenges to locally evolved breeds (Mwai et al., 2015) and remains significantly high, and there has been extremely very low introgression by exotic breeds. These observations should be given critical attention when designing conservation and breeding programs for locally indigenous animals.

**FIGURE 4:**
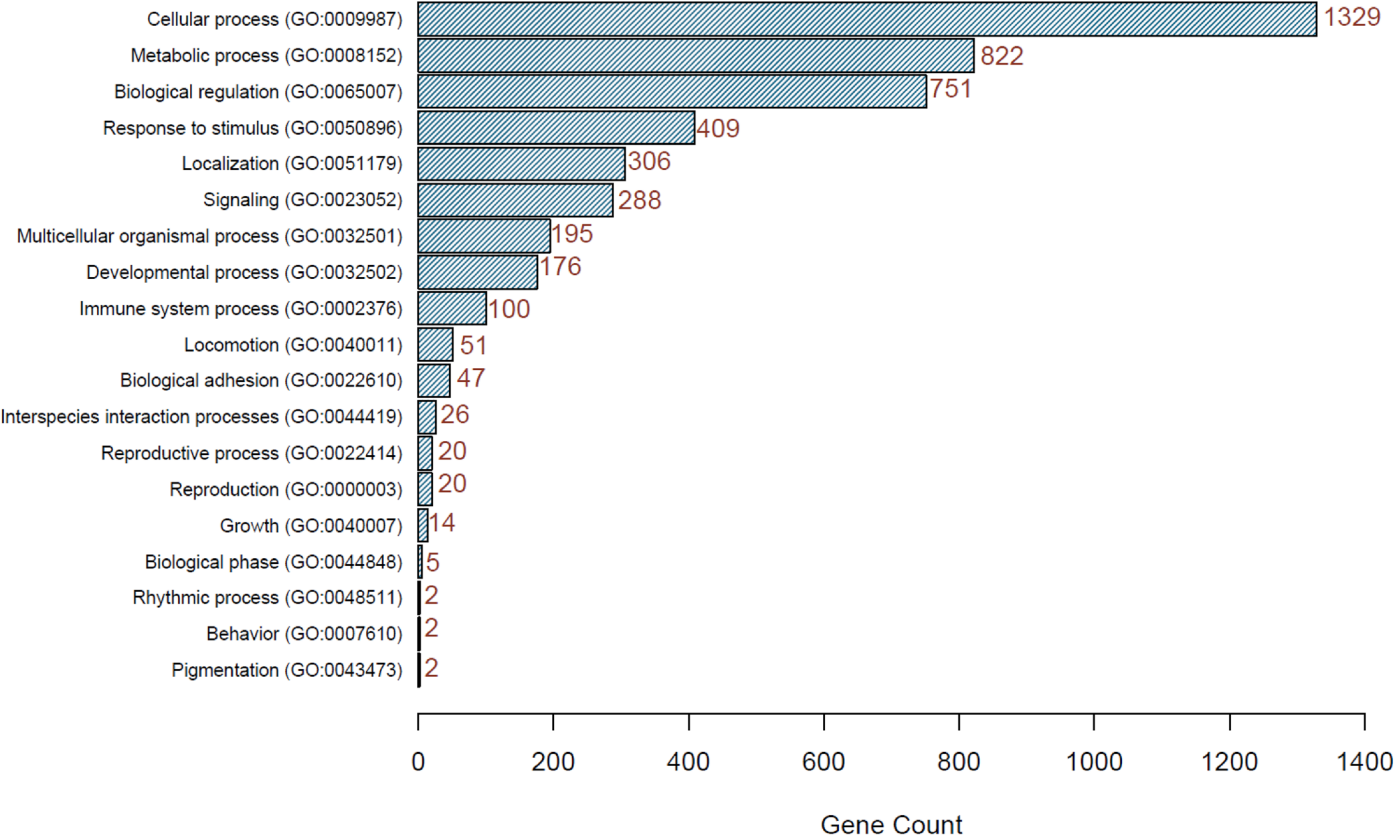
Bar plot showing major biological processes enriched by the genes located within the runs of homozygosity regions in Ugandan cattle. The numbers of genes within each biological process are presented at the end the bar of each process.

Furthermore, we identified 45 potential regions with high levels of homozygosity with lengths of 1021 kb to 42,468 kb (Table S4 in Supplementary File 4), and these regions harbour 3807 genes (Table S5 in Supplementary File 5). Of these genes, 3050 (80.1%) mapped to Panther's databases, from which 1481 mapped to characterised biological processes in Panther. These genes are mainly involved in cellular processes (GO:0009987), metabolic processes (GO:0008152), biological regulation (GO:0065007) and response to stimulus (GO:0050896) as shown in Figure 4. It is also worth noting that 100 of the genes we identified as located in the ROH regions are involved in immune response processes including antigen processing and presentation (GO:0019882), leukocyte activation (GO:0045321), immune system development (GO:0002520) and activation of immune response (GO:0002253). Interestingly these immune genes included major histocompatibility complex (MHC) genes such as *BOLA‐DBQ, BOLA‐DQA5, BOLA‐DRA, BOLA‐NC1, C4A, CYP21, BoLA‐A* and *BoLA‐B* (Behl et al., 2012; Ellis & Hammond, 2014). These genes play important roles in the immune response of animals against different pathogenic agents. *BoLA‐A*, *BoLA‐B* and *BOLA*‐*NC1* encode for the bovine lymphocyte antigen alpha chain A, bovine lymphocyte antigen alpha chain B, and nonclassical bovine lymphocyte antigen alpha chain A proteins, respectively (Behl et al., 2012; Ellis & Hammond, 2014). These are MHC class I protein molecules that are located on the membrane of all nucleated body cells and are responsible for presenting intracellular peptides to CD8+ T cells that kill pathogen‐infected or malignant tumour cells (Behl et al., 2012; Ellis, 2004; Ellis & Hammond, 2014). *BOLA*‐*DBQ*, *BOLA*‐*DQA5* and *BOLA*‐*DRA* encode for major histocompatibility complex class II bovine leukocyte antigen protein molecules that are expressed on the cell surface of antigen processing cells (Behl et al., 2012). These MCH class II proteins are tasked with the presentation of peptides derived from phagocytosed pathogens to the CD4 helper T cells that initiate inflammatory response, and antibody secretion by B cells against the invading pathogen (Behl et al., 2012). The genes *C4A* and CYP21 encode for MHC class III proteins complement component 4A and steroid 21‐hydroxylase, respectively (Behl et al., 2012). Complement component 4A is a component of the complement system immune response to pathogen infection (Janeway et al., 2001). The identification of ROH regions containing these key genes in immune response correlates with the available reports of indigenous animals being resistant to endemic diseases and parasites (Kasaija et al., 2021). Therefore, phenotype/genotype association studies should be pursued as they might identify causal mutations for these unique and important characteristics and potentially inform selective breeding decisions in these cattle populations.

## 4 CONCLUSIONS

Livestock rightful ownership conflicts are an important problem in the livestock farming communities in Africa. This problem is exacerbated by the lack of reliable animal identification systems in these communities that can be used to establish the rightful animal owners when conflicts arise. In our current study, we have demonstrated the soundness of utilising currently available genomic tools to establish livestock owners between conflicting parties. Our genome‐wide genomic analyses determined the most likely owner of a group of nine cattle whose ownership two conflicting herd owners were claiming. In addition, genomic characterisation analyses showed low exotic genetic introgression into Uganda indigenous cattle and also identified MHC gene harbouring genomic regions as potentially under natural or artificial selection in Ugandan indigenous cattle. We acknowledge the limitation that our observations are made on a small sample size of Ugandan cattle; however, these results contribute to the growing genomic characterisation of indigenous Ugandan livestock that has the potential to guide selective breeding cattle in Uganda.

## AUTHOR CONTRIBUTIONS

KKB, CM and RM conceptualised the study. KKB, CM, RM, RPN and JM contributed to sample collection and processing. RM performed the genetic data analyses. RM, CM and KKB explored and interpreted the results from the analyses. PB, CM and KKB mobilised the financial recourses for study. OO provided the comparison genotype dataset used in this study. RM and KKB drafted the original manuscript. PB and CM oversaw and supervised the implementation of the whole study. RPN, KKB, CM and RM contributed to manuscript final formatting. All authors contributed to revision, editing and final structuring of the manuscript.

## CONFLICT OF INTEREST STATEMENT

The authors declare no conflict of interest.

## FUNDING INFORMATION

This research was funded by Government of Uganda through the National Animal Genetics Resources Centre and Data Bank (NAGRC&DB).

## ETHICS STATEMENT

This study was approved by National Animal Genetics Resources Centre and Data Bank (NAGRC&DB). Ethical approval number: SVAR_IACUC/104/2022.

### PEER REVIEW

The peer review history for this article is available at https://publons.com/publon/10.1002/vms3.1272

## Supporting information


**Table S1**: Morphological descriptions of the studied animals.

**Table S2**: Plink SNP genotype‐based sex validation (–check‐sex).

**Table S3**: Identity by descent (IBD) analysis results.

**Table S4**: Runs of homozygosity identified in the Ugandan indigenous cattle.

**Table S5**: Candidate genes within the identified runs of homozygosity regions.

## Data Availability

The data that support the findings of this study are available on request from the corresponding author and on a public web based database.
