# Supplementary material for: A case of forensic genomics in Uganda reveals animal ownership and low exotic genetic introgression in indigenous cattle
Source: Vet Med Sci. 2023 Sep 19;9(6):2844–51. doi: 10.1002/vms3.1272 (PMC10650367; doi:10.1002/vms3.1272)
Supplement: Supplementary file 1 — Table S1: Morphological descriptions of the studied animals. [file VMS3-9-2844-s004.docx]

**Supplementary File 1**

**Table S1:** Morphological descriptions of the studied animals

| **Sample ID** | **Animal group** | **Coat Colour** | **Sex** | **Age group** | **Horn Description** | **PEDSEX** | **SNPSEX** | **STATUS** | **F** |
| --- | --- | --- | --- | --- | --- | --- | --- | --- | --- |
| A1 | Farmer A | Spotted brown | Male | Adult | Long-horned | 1 | 1 | OK | 0.8831 |
| A2 | Farmer A | Spotted brown | Female | Adult | Long-horned | 2 | 2 | OK | -0.00812 |
| A3 | Farmer A | Brown with some spots on the abdomen | Female | Adult | Lateral short-horned | 2 | 2 | OK | 0.1352 |
| A4 | Farmer A | Dark brown | Female | Adult | Long-horned | 2 | 2 | OK | -0.1718 |
| A5 | Farmer A | Brown | Female | Adult | Long-horned | 2 | 2 | OK | 0.04551 |
| A6 | Farmer A | Brown | Female | Adult | Long-horned | 2 | 2 | OK | -0.0418 |
| A7 | Farmer A | Brown | Female | Adult | Long-horned | 2 | 2 | OK | 0.07871 |
| B1 | Farmer B | Brown and white patches | Female | Adult | Long-horned | 2 | 2 | OK | -0.05055 |
| B2 | Farmer B | Brown with white patches on the head | Female | Adult | Polled | 2 | 2 | OK | -0.1478 |
| C1 | Contested | Brown | Female | Adult | Lateral short-horned | 2 | 2 | OK | -0.1296 |
| C2 | Contested | Brown | Female | Adult | Long horned | 2 | 2 | OK | 0.05224 |
| C3 | Contested | Brown with white patches on head | Female | Adult | Long-horned | 2 | 2 | OK | -0.04469 |
| C4 | Contested | Black | Female | Adult | Polled | 2 | 2 | OK | -0.1298 |
| C5 | Contested | Brown | Female | Adult | Long-horned | 2 | 2 | OK | -0.06849 |
| C6 | Contested | Black with white patches on the head | Female | Yearling | Polled | 2 | 2 | OK | -0.1386 |
| C7 | Contested | Black with brown ears | Male | Yearling | Polled | 1 | 1 | OK | 0.892 |
| C8 | Contested | Black and white | Female | Yearling | Short-horned | 2 | 2 | OK | -0.3367 |
| C9 | Contested | Black | Male | Yearling | Polled | 1 | 1 | OK | 0.8922 |
