# Supplementary material for: A case of forensic genomics in Uganda reveals animal ownership and low exotic genetic introgression in indigenous cattle
Source: Vet Med Sci. 2023 Sep 19;9(6):2844–51. doi: 10.1002/vms3.1272 (PMC10650367; doi:10.1002/vms3.1272)
Supplement: Supplementary file 2 — Table S2: Plink SNP genotype‐based sex validation (–check‐sex). [file VMS3-9-2844-s003.docx]

**Supplementary File 1**

**Table S2**: Plink SNP genotype-based sex validation (--check-sex)

| **Sample ID** | **Recorded sex** | **PEDSEX (1 = Male, 2=Female)** | **SNPSEX (1 = Male, 2=Female)** | **STATUS** | **F** |
| --- | --- | --- | --- | --- | --- |
| A1 | Male | 1 | 1 | OK | 0.8831 |
| A2 | Female | 2 | 2 | OK | -0.00812 |
| A3 | Female | 2 | 2 | OK | 0.1352 |
| A4 | Female | 2 | 2 | OK | -0.1718 |
| A5 | Female | 2 | 2 | OK | 0.04551 |
| A6 | Female | 2 | 2 | OK | -0.0418 |
| A7 | Female | 2 | 2 | OK | 0.07871 |
| B1 | Female | 2 | 2 | OK | -0.05055 |
| B2 | Female | 2 | 2 | OK | -0.1478 |
| C1 | Female | 2 | 2 | OK | -0.1296 |
| C2 | Female | 2 | 2 | OK | 0.05224 |
| C3 | Female | 2 | 2 | OK | -0.04469 |
| C4 | Female | 2 | 2 | OK | -0.1298 |
| C5 | Female | 2 | 2 | OK | -0.06849 |
| C6 | Female | 2 | 2 | OK | -0.1386 |
| C7 | Male | 1 | 1 | OK | 0.892 |
| C8 | Female | 2 | 2 | OK | -0.3367 |
| C9 | Male | 1 | 1 | OK | 0.8922 |

F= The actual X chromosome inbreeding (homozygosity) estimate. A male sex was called for F > 0.8, while the Female call was made if F< 0.2.
