# Supplementary material for: A case of forensic genomics in Uganda reveals animal ownership and low exotic genetic introgression in indigenous cattle
Source: Vet Med Sci. 2023 Sep 19;9(6):2844–51. doi: 10.1002/vms3.1272 (PMC10650367; doi:10.1002/vms3.1272)
Supplement: Supplementary file 3 — Table S3: Identity by descent (IBD) analysis results. [file VMS3-9-2844-s005.docx]

**Supplementary File 1**

**Table S3:** Identity by descent (IBD) analysis results

| **FID1** | **IID1** | **FID2** | **IID2** | **RT** | **EZ** | **Z0** | **Z1** | **Z2** | **PI_HAT** | **PHE** | **DST** | **PPC** | **RATIO** |
| --- | --- | --- | --- | --- | --- | --- | --- | --- | --- | --- | --- | --- | --- |
| 1 | C1 | 2 | C2 | UN | NA | 0.9627 | 0.0373 | 0 | 0.0186 | -1 | 0.713193 | 0.8695 | 2.0942 |
| 1 | C1 | 3 | C3 | UN | NA | 0.291 | 0.4659 | 0.243 | 0.476 | -1 | 0.833164 | 1 | 8.6489 |
| 1 | C1 | 4 | C4 | UN | NA | 0.6545 | 0.3084 | 0.0372 | 0.1914 | -1 | 0.757089 | 1 | 3.4346 |
| 1 | C1 | 5 | C5 | UN | NA | 0.4611 | 0.5105 | 0.0284 | 0.2837 | -1 | 0.776408 | 1 | 5.0521 |
| 1 | C1 | 6 | C6 | UN | NA | 0.6858 | 0.3142 | 0 | 0.1571 | -1 | 0.727719 | 1 | 3.2043 |
| 1 | C1 | 7 | C7 | UN | NA | 1 | 0 | 0 | 0 | -1 | 0.686372 | 0.4682 | 1.9936 |
| 1 | C1 | 8 | C8 | UN | NA | 0.5564 | 0.4436 | 0 | 0.2218 | -1 | 0.728921 | 1 | 3.4702 |
| 1 | C1 | 9 | C9 | UN | NA | 1 | 0 | 0 | 0 | -1 | 0.688718 | 0.1395 | 1.9156 |
| 1 | C1 | 10 | B1 | UN | NA | 0.3859 | 0.494 | 0.1201 | 0.3671 | -1 | 0.800941 | 1 | 6.4774 |
| 1 | C1 | 11 | B2 | UN | NA | 0.4329 | 0.48 | 0.0871 | 0.3271 | -1 | 0.789949 | 1 | 5.8627 |
| 1 | C1 | 12 | A1 | UN | NA | 1 | 0 | 0 | 0 | -1 | 0.707198 | 0.2411 | 1.9441 |
| 1 | C1 | 13 | A2 | UN | NA | 1 | 0 | 0 | 0 | -1 | 0.706459 | 0.4354 | 1.9868 |
| 1 | C1 | 14 | A3 | UN | NA | 1 | 0 | 0 | 0 | -1 | 0.708834 | 0.0393 | 1.8628 |
| 1 | C1 | 15 | A4 | UN | NA | 1 | 0 | 0 | 0 | -1 | 0.710485 | 0.4946 | 1.9989 |
| 1 | C1 | 16 | A5 | UN | NA | 1 | 0 | 0 | 0 | -1 | 0.708733 | 0.0454 | 1.8677 |
| 1 | C1 | 17 | A6 | UN | NA | 1 | 0 | 0 | 0 | -1 | 0.711977 | 0.6583 | 2.0336 |
| 1 | C1 | 18 | A7 | UN | NA | 1 | 0 | 0 | 0 | -1 | 0.710587 | 0.5652 | 2.0135 |
| 2 | C2 | 3 | C3 | UN | NA | 1 | 0 | 0 | 0 | -1 | 0.712585 | 0.9386 | 2.1307 |
| 2 | C2 | 4 | C4 | UN | NA | 0.8488 | 0.1512 | 0 | 0.0756 | -1 | 0.727169 | 1 | 2.4517 |
| 2 | C2 | 5 | C5 | UN | NA | 0.9435 | 0.0565 | 0 | 0.0282 | -1 | 0.714859 | 0.9507 | 2.1417 |
| 2 | C2 | 6 | C6 | UN | NA | 1 | 0 | 0 | 0 | -1 | 0.693599 | 0.9516 | 2.1396 |
| 2 | C2 | 7 | C7 | UN | NA | 0 | 1 | 0 | 0.5 | -1 | 0.795945 | 1 | 175.7143 |
| 2 | C2 | 8 | C8 | UN | NA | 1 | 0 | 0 | 0 | -1 | 0.692426 | 0.9166 | 2.1156 |
| 2 | C2 | 9 | C9 | UN | NA | 0.6991 | 0.3009 | 0 | 0.1504 | -1 | 0.719334 | 1 | 2.8647 |
| 2 | C2 | 10 | B1 | UN | NA | 1 | 0 | 0 | 0 | -1 | 0.71567 | 0.8722 | 2.0952 |
| 2 | C2 | 11 | B2 | UN | NA | 0.9448 | 0.0552 | 0 | 0.0276 | -1 | 0.709022 | 0.8907 | 2.1032 |
| 2 | C2 | 12 | A1 | UN | NA | 0.9704 | 0.006 | 0.0235 | 0.0265 | -1 | 0.720521 | 0.8738 | 2.0966 |
| 2 | C2 | 13 | A2 | UN | NA | 1 | 0 | 0 | 0 | -1 | 0.71454 | 0.4946 | 1.9989 |
| 2 | C2 | 14 | A3 | UN | NA | 1 | 0 | 0 | 0 | -1 | 0.709674 | 0.2399 | 1.9435 |
| 2 | C2 | 15 | A4 | UN | NA | 0.959 | 0.0287 | 0.0123 | 0.0267 | -1 | 0.719754 | 0.7523 | 2.0568 |
| 2 | C2 | 16 | A5 | UN | NA | 1 | 0 | 0 | 0 | -1 | 0.71218 | 0.0253 | 1.8487 |
| 2 | C2 | 17 | A6 | UN | NA | 0.9793 | 0 | 0.0207 | 0.0207 | -1 | 0.718233 | 0.9196 | 2.1191 |
| 2 | C2 | 18 | A7 | UN | NA | 0.9893 | 0.0107 | 0 | 0.0053 | -1 | 0.712411 | 0.5869 | 2.0182 |
| 3 | C3 | 4 | C4 | UN | NA | 0.8113 | 0.1887 | 0 | 0.0943 | -1 | 0.731716 | 1 | 2.5596 |
| 3 | C3 | 5 | C5 | UN | NA | 0.3789 | 0.5879 | 0.0332 | 0.3271 | -1 | 0.78614 | 1 | 6.4895 |
| 3 | C3 | 6 | C6 | UN | NA | 0.7457 | 0.2543 | 0 | 0.1271 | -1 | 0.720999 | 1 | 2.9884 |
| 3 | C3 | 7 | C7 | UN | NA | 1 | 0 | 0 | 0 | -1 | 0.686112 | 0.6052 | 2.0215 |
| 3 | C3 | 8 | C8 | UN | NA | 0.5406 | 0.4594 | 0 | 0.2297 | -1 | 0.732165 | 1 | 3.7291 |
| 3 | C3 | 9 | C9 | UN | NA | 1 | 0 | 0 | 0 | -1 | 0.688979 | 0.029 | 1.8545 |
| 3 | C3 | 10 | B1 | UN | NA | 0.2604 | 0.5728 | 0.1668 | 0.4532 | -1 | 0.822839 | 1 | 9.9331 |
| 3 | C3 | 11 | B2 | UN | NA | 0.3577 | 0.6423 | 0 | 0.3211 | -1 | 0.775062 | 1 | 6.0474 |
| 3 | C3 | 12 | A1 | UN | NA | 0.9989 | 0 | 0.0011 | 0.0011 | -1 | 0.713425 | 0.3571 | 1.9705 |
| 3 | C3 | 13 | A2 | UN | NA | 1 | 0 | 0 | 0 | -1 | 0.706604 | 0.0267 | 1.8502 |
| 3 | C3 | 14 | A3 | UN | NA | 1 | 0 | 0 | 0 | -1 | 0.709906 | 0.1612 | 1.9216 |
| 3 | C3 | 15 | A4 | UN | NA | 1 | 0 | 0 | 0 | -1 | 0.711875 | 0.2449 | 1.9449 |
| 3 | C3 | 16 | A5 | UN | NA | 1 | 0 | 0 | 0 | -1 | 0.711948 | 0.0129 | 1.8275 |
| 3 | C3 | 17 | A6 | UN | NA | 1 | 0 | 0 | 0 | -1 | 0.712411 | 0.4783 | 1.9955 |
| 3 | C3 | 18 | A7 | UN | NA | 1 | 0 | 0 | 0 | -1 | 0.709138 | 0.2615 | 1.9488 |
| 4 | C4 | 5 | C5 | UN | NA | 0.7824 | 0.2126 | 0.005 | 0.1113 | -1 | 0.737524 | 1 | 2.6296 |
| 4 | C4 | 6 | C6 | UN | NA | 0.8283 | 0.1717 | 0 | 0.0859 | -1 | 0.714873 | 1 | 2.5096 |
| 4 | C4 | 7 | C7 | UN | NA | 1 | 0 | 0 | 0 | -1 | 0.69189 | 0.9659 | 2.1544 |
| 4 | C4 | 8 | C8 | UN | NA | 0.8748 | 0.1252 | 0 | 0.0626 | -1 | 0.703389 | 0.9984 | 2.2586 |
| 4 | C4 | 9 | C9 | UN | NA | 0.9086 | 0.0914 | 0 | 0.0457 | -1 | 0.700521 | 0.7615 | 2.0584 |
| 4 | C4 | 10 | B1 | UN | NA | 0.8343 | 0.1492 | 0.0165 | 0.0911 | -1 | 0.733961 | 1 | 2.5249 |
| 4 | C4 | 11 | B2 | UN | NA | 0.6684 | 0.3122 | 0.0195 | 0.1756 | -1 | 0.752426 | 1 | 3.0723 |
| 4 | C4 | 12 | A1 | UN | NA | 0.9811 | 0.0179 | 0.001 | 0.01 | -1 | 0.715351 | 0.5271 | 2.0056 |
| 4 | C4 | 13 | A2 | UN | NA | 1 | 0 | 0 | 0 | -1 | 0.71134 | 0.0018 | 1.7797 |
| 4 | C4 | 14 | A3 | UN | NA | 1 | 0 | 0 | 0 | -1 | 0.711166 | 0.0176 | 1.8375 |
| 4 | C4 | 15 | A4 | UN | NA | 0.9752 | 0 | 0.0248 | 0.0248 | -1 | 0.719508 | 0.3224 | 1.963 |
| 4 | C4 | 16 | A5 | UN | NA | 1 | 0 | 0 | 0 | -1 | 0.713237 | 0.0835 | 1.8917 |
| 4 | C4 | 17 | A6 | UN | NA | 1 | 0 | 0 | 0 | -1 | 0.718132 | 0.2847 | 1.9544 |
| 4 | C4 | 18 | A7 | UN | NA | 1 | 0 | 0 | 0 | -1 | 0.715786 | 0.1604 | 1.9211 |
| 5 | C5 | 6 | C6 | UN | NA | 0.7507 | 0.2493 | 0 | 0.1247 | -1 | 0.722404 | 1 | 2.9397 |
| 5 | C5 | 7 | C7 | UN | NA | 1 | 0 | 0 | 0 | -1 | 0.686387 | 0.6513 | 2.0316 |
| 5 | C5 | 8 | C8 | UN | NA | 0 | 1 | 0 | 0.5 | -1 | 0.793787 | 1 | 465.4 |
| 5 | C5 | 9 | C9 | UN | NA | 1 | 0 | 0 | 0 | -1 | 0.690471 | 0.0242 | 1.8499 |
| 5 | C5 | 10 | B1 | UN | NA | 0.5228 | 0.4504 | 0.0267 | 0.252 | -1 | 0.769442 | 1 | 4.3676 |
| 5 | C5 | 11 | B2 | UN | NA | 0.3726 | 0.6274 | 0 | 0.3137 | -1 | 0.780782 | 1 | 7.0707 |
| 5 | C5 | 12 | A1 | UN | NA | 1 | 0 | 0 | 0 | -1 | 0.71454 | 0.4089 | 1.9814 |
| 5 | C5 | 13 | A2 | UN | NA | 1 | 0 | 0 | 0 | -1 | 0.710239 | 0.7336 | 2.0516 |
| 5 | C5 | 14 | A3 | UN | NA | 1 | 0 | 0 | 0 | -1 | 0.709573 | 0.169 | 1.9244 |
| 5 | C5 | 15 | A4 | UN | NA | 1 | 0 | 0 | 0 | -1 | 0.712585 | 0.3191 | 1.9626 |
| 5 | C5 | 16 | A5 | UN | NA | 1 | 0 | 0 | 0 | -1 | 0.714135 | 0.0081 | 1.8151 |
| 5 | C5 | 17 | A6 | UN | NA | 1 | 0 | 0 | 0 | -1 | 0.714135 | 0.2757 | 1.9524 |
| 5 | C5 | 18 | A7 | UN | NA | 1 | 0 | 0 | 0 | -1 | 0.712426 | 0.2346 | 1.9418 |
| 6 | C6 | 7 | C7 | UN | NA | 1 | 0 | 0 | 0 | -1 | 0.682766 | 0.9918 | 2.2018 |
| 6 | C6 | 8 | C8 | UN | NA | 0.8433 | 0.1567 | 0 | 0.0784 | -1 | 0.696408 | 1 | 2.5198 |
| 6 | C6 | 9 | C9 | UN | NA | 1 | 0 | 0 | 0 | -1 | 0.69441 | 0.999 | 2.2634 |
| 6 | C6 | 10 | B1 | UN | NA | 0.7932 | 0.2068 | 0 | 0.1034 | -1 | 0.716148 | 1 | 2.7156 |
| 6 | C6 | 11 | B2 | UN | NA | 0 | 1 | 0 | 0.5 | -1 | 0.798711 | 1 | 122.75 |
| 6 | C6 | 12 | A1 | UN | NA | 1 | 0 | 0 | 0 | -1 | 0.695105 | 0.5478 | 2.0096 |
| 6 | C6 | 13 | A2 | UN | NA | 1 | 0 | 0 | 0 | -1 | 0.690688 | 0.0004 | 1.7568 |
| 6 | C6 | 14 | A3 | UN | NA | 1 | 0 | 0 | 0 | -1 | 0.692426 | 0.1469 | 1.9186 |
| 6 | C6 | 15 | A4 | UN | NA | 1 | 0 | 0 | 0 | -1 | 0.694453 | 0.0558 | 1.8785 |
| 6 | C6 | 16 | A5 | UN | NA | 1 | 0 | 0 | 0 | -1 | 0.695221 | 0.0174 | 1.8408 |
| 6 | C6 | 17 | A6 | UN | NA | 1 | 0 | 0 | 0 | -1 | 0.694265 | 0.2436 | 1.9453 |
| 6 | C6 | 18 | A7 | UN | NA | 1 | 0 | 0 | 0 | -1 | 0.695163 | 0.0294 | 1.8573 |
| 7 | C7 | 8 | C8 | UN | NA | 0.4734 | 0.5266 | 0 | 0.2633 | -1 | 0.758117 | 1 | 5.4037 |
| 7 | C7 | 9 | C9 | UN | NA | 0.4517 | 0.5371 | 0.0112 | 0.2797 | -1 | 0.774337 | 1 | 6.3592 |
| 7 | C7 | 10 | B1 | UN | NA | 1 | 0 | 0 | 0 | -1 | 0.690847 | 0.8598 | 2.0888 |
| 7 | C7 | 11 | B2 | UN | NA | 1 | 0 | 0 | 0 | -1 | 0.685938 | 0.85 | 2.085 |
| 7 | C7 | 12 | A1 | UN | NA | 1 | 0 | 0 | 0 | -1 | 0.691093 | 0.5214 | 2.0043 |
| 7 | C7 | 13 | A2 | UN | NA | 1 | 0 | 0 | 0 | -1 | 0.687864 | 0.666 | 2.0349 |
| 7 | C7 | 14 | A3 | UN | NA | 1 | 0 | 0 | 0 | -1 | 0.687632 | 0.1134 | 1.9067 |
| 7 | C7 | 15 | A4 | UN | NA | 1 | 0 | 0 | 0 | -1 | 0.691108 | 0.626 | 2.026 |
| 7 | C7 | 16 | A5 | UN | NA | 1 | 0 | 0 | 0 | -1 | 0.686604 | 0.0193 | 1.8422 |
| 7 | C7 | 17 | A6 | UN | NA | 1 | 0 | 0 | 0 | -1 | 0.690138 | 0.5481 | 2.0097 |
| 7 | C7 | 18 | A7 | UN | NA | 1 | 0 | 0 | 0 | -1 | 0.687734 | 0.3637 | 1.9722 |
| 8 | C8 | 9 | C9 | UN | NA | 0.4708 | 0.5292 | 0 | 0.2646 | -1 | 0.759247 | 1 | 5.216 |
| 8 | C8 | 10 | B1 | UN | NA | 0.6379 | 0.3621 | 0 | 0.1811 | -1 | 0.722129 | 1 | 3.1287 |
| 8 | C8 | 11 | B2 | UN | NA | 0.6039 | 0.3961 | 0 | 0.198 | -1 | 0.725069 | 1 | 3.4111 |
| 8 | C8 | 12 | A1 | UN | NA | 1 | 0 | 0 | 0 | -1 | 0.691151 | 0.1875 | 1.9301 |
| 8 | C8 | 13 | A2 | UN | NA | 1 | 0 | 0 | 0 | -1 | 0.691513 | 0.0035 | 1.7978 |
| 8 | C8 | 14 | A3 | UN | NA | 1 | 0 | 0 | 0 | -1 | 0.68908 | 0.2073 | 1.9359 |
| 8 | C8 | 15 | A4 | UN | NA | 1 | 0 | 0 | 0 | -1 | 0.690731 | 0.0526 | 1.8753 |
| 8 | C8 | 16 | A5 | UN | NA | 1 | 0 | 0 | 0 | -1 | 0.690138 | 0.0029 | 1.7925 |
| 8 | C8 | 17 | A6 | UN | NA | 1 | 0 | 0 | 0 | -1 | 0.689037 | 0.1823 | 1.9281 |
| 8 | C8 | 18 | A7 | UN | NA | 1 | 0 | 0 | 0 | -1 | 0.692223 | 0.1919 | 1.9316 |
| 9 | C9 | 10 | B1 | UN | NA | 1 | 0 | 0 | 0 | -1 | 0.690181 | 0.3843 | 1.9765 |
| 9 | C9 | 11 | B2 | UN | NA | 1 | 0 | 0 | 0 | -1 | 0.692368 | 0.7745 | 2.061 |
| 9 | C9 | 12 | A1 | UN | NA | 1 | 0 | 0 | 0 | -1 | 0.697147 | 0.5638 | 2.013 |
| 9 | C9 | 13 | A2 | UN | NA | 1 | 0 | 0 | 0 | -1 | 0.694815 | 0.295 | 1.9571 |
| 9 | C9 | 14 | A3 | UN | NA | 1 | 0 | 0 | 0 | -1 | 0.694381 | 0.2193 | 1.9392 |
| 9 | C9 | 15 | A4 | UN | NA | 1 | 0 | 0 | 0 | -1 | 0.696293 | 0.14 | 1.916 |
| 9 | C9 | 16 | A5 | UN | NA | 1 | 0 | 0 | 0 | -1 | 0.688689 | 0.0037 | 1.7969 |
| 9 | C9 | 17 | A6 | UN | NA | 1 | 0 | 0 | 0 | -1 | 0.695119 | 0.0668 | 1.8841 |
| 9 | C9 | 18 | A7 | UN | NA | 1 | 0 | 0 | 0 | -1 | 0.692629 | 0.1846 | 1.9295 |
| 10 | B1 | 11 | B2 | UN | NA | 0.4987 | 0.5013 | 0 | 0.2506 | -1 | 0.761173 | 1 | 4.4754 |
| 10 | B1 | 12 | A1 | UN | NA | 1 | 0 | 0 | 0 | -1 | 0.716075 | 0.9071 | 2.1111 |
| 10 | B1 | 13 | A2 | UN | NA | 1 | 0 | 0 | 0 | -1 | 0.707343 | 0.0103 | 1.8232 |
| 10 | B1 | 14 | A3 | UN | NA | 1 | 0 | 0 | 0 | -1 | 0.708009 | 0.1574 | 1.9203 |
| 10 | B1 | 15 | A4 | UN | NA | 1 | 0 | 0 | 0 | -1 | 0.709833 | 0.0024 | 1.788 |
| 10 | B1 | 16 | A5 | UN | NA | 1 | 0 | 0 | 0 | -1 | 0.711702 | 0.0074 | 1.8135 |
| 10 | B1 | 17 | A6 | UN | NA | 1 | 0 | 0 | 0 | -1 | 0.709529 | 0.1928 | 1.931 |
| 10 | B1 | 18 | A7 | UN | NA | 0.986 | 0 | 0.014 | 0.014 | -1 | 0.716017 | 0.8181 | 2.0757 |
| 11 | B2 | 12 | A1 | UN | NA | 1 | 0 | 0 | 0 | -1 | 0.707922 | 0.5161 | 2.0033 |
| 11 | B2 | 13 | A2 | UN | NA | 1 | 0 | 0 | 0 | -1 | 0.707038 | 0.0016 | 1.778 |
| 11 | B2 | 14 | A3 | UN | NA | 1 | 0 | 0 | 0 | -1 | 0.705735 | 0.0377 | 1.8629 |
| 11 | B2 | 15 | A4 | UN | NA | 1 | 0 | 0 | 0 | -1 | 0.709066 | 0.5054 | 2.0011 |
| 11 | B2 | 16 | A5 | UN | NA | 1 | 0 | 0 | 0 | -1 | 0.709515 | 0.0429 | 1.8658 |
| 11 | B2 | 17 | A6 | UN | NA | 1 | 0 | 0 | 0 | -1 | 0.711803 | 0.5807 | 2.0167 |
| 11 | B2 | 18 | A7 | UN | NA | 1 | 0 | 0 | 0 | -1 | 0.708125 | 0.473 | 1.9945 |
| 12 | A1 | 13 | A2 | UN | NA | 0.6814 | 0.3087 | 0.0099 | 0.1642 | -1 | 0.749298 | 1 | 3.0265 |
| 12 | A1 | 14 | A3 | UN | NA | 0.9675 | 0.0325 | 0 | 0.0163 | -1 | 0.716481 | 0.9817 | 2.1803 |
| 12 | A1 | 15 | A4 | UN | NA | 1 | 0 | 0 | 0 | -1 | 0.713613 | 0.1772 | 1.9262 |
| 12 | A1 | 16 | A5 | UN | NA | 0.5073 | 0.4585 | 0.0342 | 0.2635 | -1 | 0.772455 | 1 | 4.347 |
| 12 | A1 | 17 | A6 | UN | NA | 1 | 0 | 0 | 0 | -1 | 0.715916 | 0.2104 | 1.9355 |
| 12 | A1 | 18 | A7 | UN | NA | 0.5723 | 0.4171 | 0.0106 | 0.2192 | -1 | 0.761217 | 1 | 3.998 |
| 13 | A2 | 14 | A3 | UN | NA | 1 | 0 | 0 | 0 | -1 | 0.714642 | 0.0774 | 1.8876 |
| 13 | A2 | 15 | A4 | UN | NA | 1 | 0 | 0 | 0 | -1 | 0.717625 | 0.2401 | 1.9435 |
| 13 | A2 | 16 | A5 | UN | NA | 0.7121 | 0.2621 | 0.0258 | 0.1569 | -1 | 0.748834 | 1 | 2.8731 |
| 13 | A2 | 17 | A6 | UN | NA | 0.9639 | 0.0083 | 0.0278 | 0.0319 | -1 | 0.721984 | 0.7407 | 2.0541 |
| 13 | A2 | 18 | A7 | UN | NA | 0.6761 | 0.3128 | 0.0111 | 0.1675 | -1 | 0.75008 | 1 | 3.1573 |
| 14 | A3 | 15 | A4 | UN | NA | 1 | 0 | 0 | 0 | -1 | 0.714989 | 0.1999 | 1.9329 |
| 14 | A3 | 16 | A5 | UN | NA | 0.9683 | 0 | 0.0317 | 0.0317 | -1 | 0.719406 | 0.2664 | 1.9501 |
| 14 | A3 | 17 | A6 | UN | NA | 1 | 0 | 0 | 0 | -1 | 0.715293 | 0.0725 | 1.8854 |
| 14 | A3 | 18 | A7 | UN | NA | 0.9817 | 0 | 0.0183 | 0.0183 | -1 | 0.717871 | 0.6428 | 2.0301 |
| 15 | A4 | 16 | A5 | UN | NA | 1 | 0 | 0 | 0 | -1 | 0.713121 | 0.0007 | 1.7607 |
| 15 | A4 | 17 | A6 | UN | NA | 1 | 0 | 0 | 0 | -1 | 0.717321 | 0.0985 | 1.8984 |
| 15 | A4 | 18 | A7 | UN | NA | 0.9697 | 0 | 0.0303 | 0.0303 | -1 | 0.719059 | 0.337 | 1.9661 |
| 16 | A5 | 17 | A6 | UN | NA | 1 | 0 | 0 | 0 | -1 | 0.716322 | 0.0146 | 1.8309 |
| 16 | A5 | 18 | A7 | UN | NA | 0.5126 | 0.4701 | 0.0173 | 0.2524 | -1 | 0.768863 | 1 | 4.1805 |
| 17 | A6 | 18 | A7 | UN | NA | 1 | 0 | 0 | 0 | -1 | 0.716235 | 0.0749 | 1.886 |
