# Supplementary material for: A case of forensic genomics in Uganda reveals animal ownership and low exotic genetic introgression in indigenous cattle
Source: Vet Med Sci. 2023 Sep 19;9(6):2844–51. doi: 10.1002/vms3.1272 (PMC10650367; doi:10.1002/vms3.1272)
Supplement: Supplementary file 4 — Table S4: Runs of homozygosity identified in the Ugandan indigenous cattle. [file VMS3-9-2844-s002.docx]

**Supplementary File 4**

**Table S4**: Runs of homozygosity identified in the Ugandan indigenous cattle

| **FID** | **IID** | **Chromosome** | **SNP1** | **SNP2** | **POS1** | **POS2** | **KB** | **NSNP** | **DENSITY** | **PHOM** | **PHET** |
| --- | --- | --- | --- | --- | --- | --- | --- | --- | --- | --- | --- |
| 14 | A3 | 2 | ARS-BFGL-NGS-114498 | ARS-BFGL-NGS-65317 | 74130414 | 79414331 | 5283.918 | 107 | 49.382 | 0.991 | 0.009 |
| 17 | A6 | 2 | ARS-BFGL-NGS-117340 | ARS-BFGL-NGS-117203 | 116142693 | 136697236 | 20554.54 | 478 | 43.001 | 0.998 | 0.002 |
| 17 | A6 | 3 | Hapmap27668-BTA-67346 | ARS-BFGL-NGS-19686 | 30629487 | 34869507 | 4240.021 | 170 | 24.941 | 1 | 0 |
| 14 | A3 | 3 | BTB-02075948 | Hapmap53351-ss46526783 | 32112753 | 33514564 | 1401.812 | 105 | 13.351 | 0.99 | 0.01 |
| 18 | A7 | 4 | ARS-BFGL-NGS-31713 | ARS-BFGL-NGS-1024 | 8251640 | 27842356 | 19590.72 | 394 | 49.723 | 0.997 | 0.003 |
| 2 | C2 | 4 | BTB-00170116 | UA-IFASA-7669 | 25373005 | 31759057 | 6386.053 | 121 | 52.777 | 0.983 | 0.017 |
| 9 | C9 | 4 | ARS-BFGL-NGS-105499 | BTB-01007462 | 31988357 | 37122228 | 5133.872 | 103 | 49.843 | 0.99 | 0.01 |
| 15 | A4 | 4 | BTB-00178249 | BTA-110861-no-rs | 38235868 | 47788307 | 9552.44 | 191 | 50.013 | 0.99 | 0.01 |
| 16 | A5 | 4 | BTB-00191572 | Hapmap49722-BTA-72000 | 61729628 | 104197508 | 42467.88 | 822 | 51.664 | 0.998 | 0.002 |
| 9 | C9 | 5 | ARS-BFGL-NGS-16895 | Hapmap51299-BTA-73473 | 37369462 | 43799282 | 6429.821 | 108 | 59.535 | 0.991 | 0.009 |
| 2 | C2 | 5 | Hapmap43741-BTA-100726 | ARS-USMARC-614 | 40170383 | 45722126 | 5551.744 | 100 | 55.517 | 0.99 | 0.01 |
| 10 | B1 | 5 | Hapmap50523-BTA-98407 | ARS-BFGL-NGS-21434 | 46741275 | 57305230 | 10563.96 | 203 | 52.039 | 0.985 | 0.015 |
| 13 | A2 | 5 | BTA-98399-no-rs | BTA-87617-no-rs | 46968644 | 58847022 | 11878.38 | 218 | 54.488 | 0.995 | 0.005 |
| 7 | C7 | 5 | BovineHD0500016037 | BTA-87617-no-rs | 56462859 | 58847022 | 2384.164 | 101 | 23.606 | 0.99 | 0.01 |
| 17 | A6 | 5 | BTA-75235-no-rs | ARS-BFGL-NGS-104960 | 116252285 | 121175859 | 4923.575 | 113 | 43.571 | 0.991 | 0.009 |
| 13 | A2 | 6 | BTB-01317228 | ARS-BFGL-NGS-45046 | 13012206 | 18750621 | 5738.416 | 144 | 39.85 | 0.986 | 0.014 |
| 13 | A2 | 6 | BTA-00906-rs29014057 | BTB-00274797 | 96260709 | 101054800 | 4794.092 | 109 | 43.982 | 0.991 | 0.009 |
| 13 | A2 | 8 | ARS-BFGL-NGS-70430 | ARS-BFGL-NGS-104344 | 30809624 | 37136374 | 6326.751 | 107 | 59.129 | 0.991 | 0.009 |
| 4 | C4 | 8 | BTB-00348451 | Hapmap54905-rs29018794 | 52280365 | 57531155 | 5250.791 | 107 | 49.073 | 0.963 | 0.037 |
| 2 | C2 | 8 | Hapmap54905-rs29018794 | BTB-00353359 | 57531155 | 63931756 | 6400.602 | 142 | 45.075 | 0.986 | 0.014 |
| 4 | C4 | 8 | Hapmap47748-BTA-81265 | Hapmap39528-BTA-03210 | 57756381 | 63220492 | 5464.112 | 121 | 45.158 | 0.967 | 0.033 |
| 16 | A5 | 8 | Hapmap50244-BTA-116790 | BTB-01515843 | 96780259 | 109514971 | 12734.71 | 284 | 44.841 | 0.993 | 0.007 |
| 16 | A5 | 9 | ARS-BFGL-NGS-19735 | ARS-BFGL-NGS-14740 | 7992599 | 14436985 | 6444.387 | 183 | 35.215 | 1 | 0 |
| 3 | C3 | 9 | BTB-01352726 | ARS-BFGL-NGS-36972 | 49487154 | 61665807 | 12178.65 | 311 | 39.16 | 1 | 0 |
| 5 | C5 | 9 | BTB-01352726 | ARS-BFGL-NGS-36972 | 49487154 | 61665807 | 12178.65 | 311 | 39.16 | 1 | 0 |
| 8 | C8 | 10 | BTB-00416705 | ARS-BFGL-NGS-102487 | 31574360 | 42060020 | 10485.66 | 185 | 56.679 | 0.995 | 0.005 |
| 18 | A7 | 10 | Hapmap43507-BTA-20079 | ARS-BFGL-NGS-117876 | 42830195 | 48502416 | 5672.222 | 119 | 47.666 | 0.983 | 0.017 |
| 13 | A2 | 12 | ARS-BFGL-NGS-103078 | Hapmap27733-BTA-123403 | 25412732 | 31237239 | 5824.508 | 106 | 54.948 | 0.981 | 0.019 |
| 3 | C3 | 13 | UA-IFASA-4272 | ARS-BFGL-NGS-14463 | 45925987 | 51212055 | 5286.069 | 102 | 51.824 | 0.971 | 0.029 |
| 12 | A1 | 14 | BTB-00556813 | ARS-BFGL-NGS-117404 | 23384687 | 30498375 | 7113.689 | 146 | 48.724 | 0.993 | 0.007 |
| 14 | A3 | 15 | ARS-BFGL-NGS-110461 | ARS-BFGL-NGS-103106 | 53072568 | 68288320 | 15215.75 | 308 | 49.402 | 0.994 | 0.006 |
| 17 | A6 | 15 | ARS-BFGL-NGS-3040 | BTB-00611237 | 61378465 | 66082534 | 4704.07 | 104 | 45.231 | 0.99 | 0.01 |
| 16 | A5 | 16 | BTA-39939-no-rs | ARS-BFGL-NGS-103911 | 75522046 | 81322588 | 5800.543 | 141 | 41.139 | 0.993 | 0.007 |
| 18 | A7 | 17 | ARS-BFGL-NGS-40882 | ARS-BFGL-NGS-85197 | 33098572 | 39109225 | 6010.654 | 149 | 40.34 | 0.993 | 0.007 |
| 18 | A7 | 17 | Hapmap30543-BTA-141219 | BTB-02001746 | 39681407 | 44532671 | 4851.265 | 104 | 46.647 | 0.99 | 0.01 |
| 17 | A6 | 18 | ARS-BFGL-NGS-12731 | ARS-BFGL-NGS-111067 | 59417854 | 65978584 | 6560.731 | 139 | 47.2 | 1 | 0 |
| 3 | C3 | 20 | ARS-BFGL-NGS-107554 | ARS-BFGL-NGS-34264 | 999863 | 5392124 | 4392.262 | 100 | 43.923 | 0.98 | 0.02 |
| 14 | A3 | 20 | BTA-27853-no-rs | ARS-BFGL-NGS-105013 | 2239940 | 6445208 | 4205.269 | 101 | 41.636 | 0.98 | 0.02 |
| 5 | C5 | 20 | Hapmap54326-rs29009836 | ARS-BFGL-NGS-17077 | 21160226 | 22907088 | 1746.863 | 126 | 13.864 | 0.984 | 0.016 |
| 8 | C8 | 20 | ARS-BFGL-NGS-28884 | Hapmap31141-BTA-150972 | 21764593 | 24031442 | 2266.85 | 132 | 17.173 | 0.992 | 0.008 |
| 14 | A3 | 20 | BovineHD2000006617 | ARS-BFGL-NGS-5131 | 22065122 | 23085736 | 1020.615 | 100 | 10.206 | 0.98 | 0.02 |
| 16 | A5 | 21 | Hapmap41010-BTA-52085 | ARS-BFGL-NGS-111163 | 34121218 | 54056137 | 19934.92 | 375 | 53.16 | 0.995 | 0.005 |
| 5 | C5 | 22 | ARS-BFGL-NGS-81589 | Hapmap43880-BTA-54826 | 46691404 | 52355321 | 5663.918 | 108 | 52.444 | 0.981 | 0.019 |
| 16 | A5 | 23 | ARS-BFGL-BAC-31346 | BTA-55821-no-rs | 9570626 | 27944066 | 18373.44 | 379 | 48.479 | 0.992 | 0.008 |
| 8 | C8 | 23 | ARS-BFGL-NGS-12818 | Hapmap57845-rs29014813 | 25472436 | 32998188 | 7525.753 | 155 | 48.553 | 0.987 | 0.013 |
| 17 | A6 | 25 | Hapmap48238-BTA-18211 | ARS-BFGL-BAC-37178 | 24431072 | 30062438 | 5631.367 | 104 | 54.148 | 0.99 | 0.01 |

SNP1 = SNP at the start of the region; SNP2 = SNP at the end of the region; POS1 = Physical position (bp) of SNP1; POS2 = Physical position (bp) of SNP2; KB = Length of the region (kb); NSNP = Number of SNPs in the run; DENSITY = Average SNP density (1 SNP per 60 kb); PHOM = Proportion of homozygous sites and PHET = Proportion of sites heterozygous.
