# Supplementary material for: A case of forensic genomics in Uganda reveals animal ownership and low exotic genetic introgression in indigenous cattle
Source: Vet Med Sci. 2023 Sep 19;9(6):2844–51. doi: 10.1002/vms3.1272 (PMC10650367; doi:10.1002/vms3.1272)
Supplement: Supplementary file 5 — Table S5: Candidate genes within the identified runs of homozygosity regions. [file VMS3-9-2844-s001.docx]

**Supplementary file 5**

**Table S5:** Candidate genes within the identified runs of homozygosity regions

| **Gene stable ID** | **Chromosome** | **Gene end (bp)** | **Gene start (bp)** | **Strand** | **Gene name** |
| --- | --- | --- | --- | --- | --- |
| ENSBTAG00000043636 | 10 | 31737078 | 31736947 | 1 | U2 |
| ENSBTAG00000050159 | 10 | 32041701 | 32040688 | -1 |  |
| ENSBTAG00000042615 | 10 | 32145204 | 32145001 | -1 | SNORA74 |
| ENSBTAG00000002179 | 10 | 32473979 | 32243797 | 1 | CDIN1 |
| ENSBTAG00000003172 | 10 | 32770681 | 32547550 | -1 | MEIS2 |
| ENSBTAG00000043625 | 10 | 32624833 | 32624758 | 1 | U4 |
| ENSBTAG00000027162 | 10 | 33580261 | 33565746 | 1 | TMCO5A |
| ENSBTAG00000052190 | 10 | 33731139 | 33731081 | -1 | bta-mir-2285dl-1 |
| ENSBTAG00000032518 | 10 | 33966943 | 33847959 | 1 | SPRED1 |
| ENSBTAG00000043481 | 10 | 33868354 | 33868221 | -1 | SNORA70 |
| ENSBTAG00000053989 | 10 | 34050961 | 34050855 | -1 | U6 |
| ENSBTAG00000021870 | 10 | 34092922 | 34059936 | 1 | FAM98B |
| ENSBTAG00000021872 | 10 | 34179132 | 34100135 | -1 | RASGRP1 |
| ENSBTAG00000052941 | 10 | 34200133 | 34194106 | -1 |  |
| ENSBTAG00000051091 | 10 | 34331506 | 34283930 | 1 |  |
| ENSBTAG00000002006 | 10 | 35224867 | 35209595 | 1 | THBS1 |
| ENSBTAG00000012015 | 10 | 35436219 | 35227930 | -1 | FSIP1 |
| ENSBTAG00000016826 | 10 | 35579809 | 35455221 | -1 | GPR176 |
| ENSBTAG00000054650 | 10 | 35512185 | 35511041 | 1 |  |
| ENSBTAG00000014351 | 10 | 35687969 | 35590812 | 1 | EIF2AK4 |
| ENSBTAG00000043106 | 10 | 35610138 | 35610034 | -1 | U6 |
| ENSBTAG00000014353 | 10 | 35691505 | 35688239 | -1 | SRP14 |
| ENSBTAG00000012180 | 10 | 35757288 | 35737731 | -1 | BMF |
| ENSBTAG00000007237 | 10 | 35841256 | 35786200 | 1 | BUB1B |
| ENSBTAG00000054965 | 10 | 35853157 | 35842418 | -1 |  |
| ENSBTAG00000019076 | 10 | 35895687 | 35857579 | 1 | PAK6 |
| ENSBTAG00000046052 | 10 | 35900705 | 35899557 | -1 | ANKRD63 |
| ENSBTAG00000019079 | 10 | 35927228 | 35905190 | -1 | PLCB2 |
| ENSBTAG00000051305 | 10 | 35940782 | 35940324 | 1 | INAFM2 |
| ENSBTAG00000013774 | 10 | 35956754 | 35947853 | -1 | CCDC9B |
| ENSBTAG00000049093 | 10 | 35973540 | 35969942 | 1 | PHGR1 |
| ENSBTAG00000000552 | 10 | 35985917 | 35975164 | 1 | DISP2 |
| ENSBTAG00000032340 | 10 | 36010049 | 36000138 | 1 | KNSTRN |
| ENSBTAG00000004409 | 10 | 36034673 | 36022720 | 1 | IVD |
| ENSBTAG00000004420 | 10 | 36074743 | 36050714 | 1 | BAHD1 |
| ENSBTAG00000004421 | 10 | 36079398 | 36077444 | 1 | CHST14 |
| ENSBTAG00000025593 | 10 | 36160950 | 36139789 | -1 | CCDC32 |
| ENSBTAG00000003193 | 10 | 36174903 | 36167613 | 1 | RPUSD2 |
| ENSBTAG00000045360 | 10 | 36175381 | 36175266 | -1 |  |
| ENSBTAG00000053398 | 10 | 36240615 | 36179163 | 1 | KNL1 |
| ENSBTAG00000048559 | 10 | 36232046 | 36230985 | -1 |  |
| ENSBTAG00000002918 | 10 | 36291194 | 36255232 | 1 | RAD51 |
| ENSBTAG00000002921 | 10 | 36306626 | 36291370 | -1 | RMDN3 |
| ENSBTAG00000013477 | 10 | 36316540 | 36312775 | 1 | GCHFR |
| ENSBTAG00000013480 | 10 | 36347349 | 36316703 | -1 | DNAJC17 |
| ENSBTAG00000047281 | 10 | 36320976 | 36319056 | 1 | C10H15orf62 |
| ENSBTAG00000013485 | 10 | 36354616 | 36346936 | 1 | ZFYVE19 |
| ENSBTAG00000013488 | 10 | 36378044 | 36354678 | -1 | PPP1R14D |
| ENSBTAG00000004086 | 10 | 36391629 | 36377742 | 1 | SPINT1 |
| ENSBTAG00000053721 | 10 | 36412885 | 36410171 | -1 | RHOV |
| ENSBTAG00000011567 | 10 | 36442758 | 36433611 | 1 | VPS18 |
| ENSBTAG00000010361 | 10 | 36473693 | 36464520 | 1 | DLL4 |
| ENSBTAG00000010371 | 10 | 36488431 | 36485672 | 1 | CHAC1 |
| ENSBTAG00000010380 | 10 | 36628891 | 36505672 | -1 | INO80 |
| ENSBTAG00000038438 | 10 | 36675444 | 36644180 | -1 |  |
| ENSBTAG00000008363 | 10 | 36704017 | 36677942 | -1 | EXD1 |
| ENSBTAG00000027173 | 10 | 36755883 | 36719642 | 1 | CHP1 |
| ENSBTAG00000044604 | 10 | 36746912 | 36746722 | 1 |  |
| ENSBTAG00000010766 | 10 | 36789336 | 36780762 | -1 | OIP5 |
| ENSBTAG00000010774 | 10 | 36819852 | 36788934 | 1 | NUSAP1 |
| ENSBTAG00000010778 | 10 | 36837237 | 36820290 | -1 | NDUFAF1 |
| ENSBTAG00000001671 | 10 | 36909769 | 36864199 | 1 | RTF1 |
| ENSBTAG00000001675 | 10 | 36927140 | 36918794 | 1 | ITPKA |
| ENSBTAG00000001693 | 10 | 36955679 | 36938808 | -1 | RPAP1 |
| ENSBTAG00000001694 | 10 | 36979416 | 36960794 | 1 | TYRO3 |
| ENSBTAG00000053676 | 10 | 36998934 | 36997879 | -1 |  |
| ENSBTAG00000051865 | 10 | 37023532 | 37023426 | -1 | U6 |
| ENSBTAG00000015138 | 10 | 37166996 | 37075885 | 1 | MGA |
| ENSBTAG00000053146 | 10 | 37152748 | 37152687 | -1 | bta-mir-2285cr-1 |
| ENSBTAG00000015142 | 10 | 37219738 | 37171212 | 1 | MAPKBP1 |
| ENSBTAG00000046607 | 10 | 37228629 | 37222172 | 1 | JMJD7 |
| ENSBTAG00000006784 | 10 | 37241029 | 37229548 | 1 | PLA2G4B |
| ENSBTAG00000048410 | 10 | 37243484 | 37241617 | -1 |  |
| ENSBTAG00000006790 | 10 | 37288411 | 37243193 | -1 | SPTBN5 |
| ENSBTAG00000006792 | 10 | 37379567 | 37292701 | -1 | EHD4 |
| ENSBTAG00000021924 | 10 | 37428653 | 37390030 | -1 | PLA2G4E |
| ENSBTAG00000005268 | 10 | 37507804 | 37485317 | -1 | PLA2G4D |
| ENSBTAG00000032068 | 10 | 37547704 | 37533181 | -1 | PLA2G4F |
| ENSBTAG00000032055 | 10 | 37596971 | 37550085 | -1 | VPS39 |
| ENSBTAG00000008863 | 10 | 37637423 | 37598861 | -1 | TMEM87A |
| ENSBTAG00000008866 | 10 | 37700215 | 37638467 | 1 | GANC |
| ENSBTAG00000008868 | 10 | 37766813 | 37711578 | 1 | CAPN3 |
| ENSBTAG00000004079 | 10 | 37821807 | 37769390 | -1 | ZNF106 |
| ENSBTAG00000005661 | 10 | 37866625 | 37826017 | 1 | SNAP23 |
| ENSBTAG00000031981 | 10 | 37875056 | 37869708 | -1 | LRRC57 |
| ENSBTAG00000005663 | 10 | 37888393 | 37875148 | 1 | HAUS2 |
| ENSBTAG00000048958 | 10 | 38016333 | 37950148 | 1 |  |
| ENSBTAG00000005751 | 10 | 38032350 | 38020205 | -1 | CDAN1 |
| ENSBTAG00000006470 | 10 | 38129334 | 38039980 | -1 | TTBK2 |
| ENSBTAG00000021087 | 10 | 38333647 | 38181492 | -1 | UBR1 |
| ENSBTAG00000013031 | 10 | 38393156 | 38358798 | 1 | TMEM62 |
| ENSBTAG00000013032 | 10 | 38403862 | 38393529 | 1 | CCNDBP1 |
| ENSBTAG00000011439 | 10 | 38424273 | 38404680 | -1 | EPB42 |
| ENSBTAG00000046363 | 10 | 38738472 | 38738161 | -1 |  |
| ENSBTAG00000004692 | 10 | 38834018 | 38833674 | -1 |  |
| ENSBTAG00000050225 | 10 | 39333061 | 39332898 | 1 | U1 |
| ENSBTAG00000024582 | 10 | 39648473 | 39647709 | -1 | RPL10L |
| ENSBTAG00000054557 | 10 | 39872006 | 39826061 | -1 |  |
| ENSBTAG00000005697 | 10 | 40143903 | 40090850 | -1 | MDGA2 |
| ENSBTAG00000055000 | 10 | 40451405 | 40451037 | 1 |  |
| ENSBTAG00000034580 | 10 | 40681282 | 40681124 | -1 |  |
| ENSBTAG00000051213 | 10 | 42834569 | 42834490 | -1 |  |
| ENSBTAG00000050180 | 10 | 42834792 | 42834493 | -1 | Metazoa_SRP |
| ENSBTAG00000045980 | 10 | 42859396 | 42858869 | 1 | ARF6 |
| ENSBTAG00000054867 | 10 | 42952813 | 42952719 | -1 | bta-mir-10171 |
| ENSBTAG00000005547 | 10 | 43051985 | 43046520 | -1 | VCPKMT |
| ENSBTAG00000005550 | 10 | 43157640 | 43053808 | -1 | SOS2 |
| ENSBTAG00000043035 | 10 | 43147367 | 43147261 | -1 | U6 |
| ENSBTAG00000015200 | 10 | 43219854 | 43166419 | -1 | L2HGDH |
| ENSBTAG00000029512 | 10 | 43166773 | 43166613 | -1 | U1 |
| ENSBTAG00000015202 | 10 | 43235333 | 43219972 | 1 | DMAC2L |
| ENSBTAG00000004780 | 10 | 43284821 | 43233495 | -1 | CDKL1 |
| ENSBTAG00000014792 | 10 | 43438638 | 43312765 | -1 | MAP4K5 |
| ENSBTAG00000014312 | 10 | 43524337 | 43448785 | 1 | ATL1 |
| ENSBTAG00000001539 | 10 | 43555390 | 43523556 | -1 | SAV1 |
| ENSBTAG00000054530 | 10 | 43567905 | 43566883 | -1 |  |
| ENSBTAG00000020281 | 10 | 43700027 | 43594044 | -1 | NIN |
| ENSBTAG00000044049 | 10 | 43756260 | 43738868 | 1 | ABHD12B |
| ENSBTAG00000011494 | 10 | 43797321 | 43742881 | -1 | PYGL |
| ENSBTAG00000048395 | 10 | 43830892 | 43826423 | -1 |  |
| ENSBTAG00000010103 | 10 | 43944784 | 43826973 | -1 | TRIM9 |
| ENSBTAG00000053552 | 10 | 43892020 | 43891033 | -1 |  |
| ENSBTAG00000011225 | 10 | 44070733 | 44057645 | 1 | TMX1 |
| ENSBTAG00000031707 | 10 | 44564023 | 44297021 | 1 | FRMD6 |
| ENSBTAG00000003043 | 10 | 44794168 | 44659979 | 1 | GNG2 |
| ENSBTAG00000021944 | 10 | 44846457 | 44831891 | 1 | RTRAF |
| ENSBTAG00000021945 | 10 | 44939145 | 44846568 | -1 | NID2 |
| ENSBTAG00000001423 | 10 | 45125230 | 45121371 | -1 |  |
| ENSBTAG00000006703 | 10 | 45165480 | 45157991 | 1 | PTGDR |
| ENSBTAG00000008411 | 10 | 45256216 | 45233901 | -1 | PLEKHO2 |
| ENSBTAG00000007638 | 10 | 45277456 | 45270353 | 1 | PIF1 |
| ENSBTAG00000011037 | 10 | 45339402 | 45313259 | 1 | RBPMS2 |
| ENSBTAG00000015806 | 10 | 45379381 | 45366522 | 1 | OAZ2 |
| ENSBTAG00000015808 | 10 | 45582446 | 45385273 | -1 | ZNF609 |
| ENSBTAG00000043656 | 10 | 45415857 | 45415730 | -1 |  |
| ENSBTAG00000014493 | 10 | 45639746 | 45556247 | -1 | TRIP4 |
| ENSBTAG00000049725 | 10 | 45569995 | 45563494 | 1 |  |
| ENSBTAG00000039462 | 10 | 45653956 | 45645746 | 1 | PCLAF |
| ENSBTAG00000039462 | 10 | 45653956 | 45645746 | 1 | PCLAF |
| ENSBTAG00000016823 | 10 | 45811429 | 45660900 | 1 | CSNK1G1 |
| ENSBTAG00000049412 | 10 | 45806528 | 45806201 | 1 |  |
| ENSBTAG00000016822 | 10 | 45824619 | 45818677 | 1 | PPIB |
| ENSBTAG00000016821 | 10 | 45830579 | 45824595 | -1 | SNX22 |
| ENSBTAG00000002014 | 10 | 45880562 | 45843188 | -1 | SNX1 |
| ENSBTAG00000050918 | 10 | 45874262 | 45874156 | -1 | U6 |
| ENSBTAG00000002012 | 10 | 45895812 | 45880414 | 1 | CIAO2A |
| ENSBTAG00000011820 | 10 | 46088593 | 45948472 | 1 | DAPK2 |
| ENSBTAG00000053677 | 10 | 46254294 | 46185094 | 1 | HERC1 |
| ENSBTAG00000052707 | 10 | 46319176 | 46308841 | 1 |  |
| ENSBTAG00000051076 | 10 | 46328457 | 46323963 | 1 |  |
| ENSBTAG00000054388 | 10 | 46365224 | 46356404 | 1 |  |
| ENSBTAG00000050908 | 10 | 46371736 | 46370706 | -1 |  |
| ENSBTAG00000019474 | 10 | 46394622 | 46372672 | 1 |  |
| ENSBTAG00000008807 | 10 | 46408047 | 46401956 | -1 | FBXL22 |
| ENSBTAG00000008805 | 10 | 46513245 | 46413154 | -1 | USP3 |
| ENSBTAG00000019390 | 10 | 46851817 | 46649404 | 1 | CA12 |
| ENSBTAG00000010641 | 10 | 46772271 | 46654444 | -1 | APH1B |
| ENSBTAG00000011146 | 10 | 46850532 | 46780500 | -1 | RAB8B |
| ENSBTAG00000012898 | 10 | 46892617 | 46889250 | 1 | RPS27L |
| ENSBTAG00000017373 | 10 | 46923194 | 46905306 | -1 | LACTB |
| ENSBTAG00000053715 | 10 | 46991217 | 46983028 | 1 |  |
| ENSBTAG00000005373 | 10 | 47022836 | 46993261 | -1 | TPM1 |
| ENSBTAG00000040590 | 10 | 47009992 | 47008040 | -1 |  |
| ENSBTAG00000003667 | 10 | 47742906 | 47248374 | -1 | TLN2 |
| ENSBTAG00000030049 | 10 | 47265642 | 47265558 | -1 | bta-mir-190a |
| ENSBTAG00000050590 | 10 | 47889406 | 47883828 | 1 |  |
| ENSBTAG00000020814 | 10 | 47904131 | 47902447 | 1 | C2CD4B |
| ENSBTAG00000020814 | 10 | 47904131 | 47902447 | 1 | C2CD4B |
| ENSBTAG00000020814 | 10 | 47904131 | 47902447 | 1 | C2CD4B |
| ENSBTAG00000042813 | 10 | 47954729 | 47954598 | 1 |  |
| ENSBTAG00000042731 | 10 | 47971233 | 47971129 | 1 | U6 |
| ENSBTAG00000010952 | 10 | 48034891 | 48017835 | -1 |  |
| ENSBTAG00000038920 | 10 | 48214587 | 48035067 | 1 | VPS13C |
| ENSBTAG00000050502 | 10 | 48490763 | 48304676 | 1 |  |
| ENSBTAG00000053984 | 12 | 25593295 | 25346719 | 1 |  |
| ENSBTAG00000038495 | 12 | 25689221 | 25608293 | 1 |  |
| ENSBTAG00000022991 | 12 | 26441648 | 25769802 | -1 | NBEA |
| ENSBTAG00000034069 | 12 | 25979327 | 25976904 | 1 | MAB21L1 |
| ENSBTAG00000053773 | 12 | 26452522 | 26441669 | 1 |  |
| ENSBTAG00000049023 | 12 | 26707511 | 26625368 | 1 |  |
| ENSBTAG00000049236 | 12 | 27098493 | 26987535 | 1 |  |
| ENSBTAG00000053193 | 12 | 27362596 | 27324614 | 1 |  |
| ENSBTAG00000010787 | 12 | 27396278 | 27378900 | -1 | RFC3 |
| ENSBTAG00000021815 | 12 | 28012399 | 27785642 | 1 | STARD13 |
| ENSBTAG00000013984 | 12 | 28088332 | 28051025 | -1 | KL |
| ENSBTAG00000052284 | 12 | 28170831 | 28170664 | 1 |  |
| ENSBTAG00000054876 | 12 | 28171246 | 28170791 | -1 |  |
| ENSBTAG00000052447 | 12 | 28171409 | 28171074 | 1 |  |
| ENSBTAG00000011597 | 12 | 28495424 | 28312936 | -1 | PDS5B |
| ENSBTAG00000023279 | 12 | 28586101 | 28524407 | 1 | N4BP2L2 |
| ENSBTAG00000000993 | 12 | 28614349 | 28591476 | 1 | N4BP2L1 |
| ENSBTAG00000000988 | 12 | 28667820 | 28616747 | -1 | BRCA2 |
| ENSBTAG00000026384 | 12 | 28679944 | 28670476 | 1 | ZAR1L |
| ENSBTAG00000006771 | 12 | 28997274 | 28747721 | -1 | FRY |
| ENSBTAG00000015132 | 12 | 29274747 | 29212314 | -1 | RXFP2 |
| ENSBTAG00000053332 | 12 | 29577177 | 29575199 | 1 |  |
| ENSBTAG00000044236 | 12 | 29635346 | 29635268 | -1 | bta-mir-2299 |
| ENSBTAG00000033412 | 12 | 29752743 | 29636372 | -1 | B3GLCT |
| ENSBTAG00000005012 | 12 | 29819628 | 29796159 | 1 | HSPH1 |
| ENSBTAG00000016052 | 12 | 29877617 | 29841921 | -1 |  |
| ENSBTAG00000053517 | 12 | 29911215 | 29896450 | -1 |  |
| ENSBTAG00000033386 | 12 | 30001111 | 29967343 | -1 | TEX26 |
| ENSBTAG00000008271 | 12 | 30014621 | 29997123 | -1 | MEDAG |
| ENSBTAG00000013201 | 12 | 30138259 | 30108987 | -1 | ALOX5AP |
| ENSBTAG00000001178 | 12 | 30206285 | 30183487 | -1 | USPL1 |
| ENSBTAG00000018103 | 12 | 30333011 | 30327227 | 1 | HMGB1 |
| ENSBTAG00000053560 | 12 | 30345069 | 30343824 | -1 |  |
| ENSBTAG00000009340 | 12 | 30533274 | 30478919 | 1 | KATNAL1 |
| ENSBTAG00000045239 | 12 | 30562253 | 30562148 | -1 | SNORA70 |
| ENSBTAG00000052411 | 12 | 30596761 | 30595481 | -1 |  |
| ENSBTAG00000012170 | 12 | 30854060 | 30806229 | 1 | UBL3 |
| ENSBTAG00000018577 | 12 | 30991889 | 30934105 | 1 | SLC7A1 |
| ENSBTAG00000001094 | 12 | 31388275 | 31001535 | -1 | MTUS2 |
| ENSBTAG00000014194 | 13 | 46276680 | 46039122 | 1 | ADARB2 |
| ENSBTAG00000054346 | 13 | 46282629 | 46282318 | -1 |  |
| ENSBTAG00000000683 | 13 | 46322491 | 46289405 | -1 | WDR37 |
| ENSBTAG00000004075 | 13 | 46328399 | 46325275 | 1 | IDI1 |
| ENSBTAG00000014917 | 13 | 46346321 | 46331627 | -1 | GTPBP4 |
| ENSBTAG00000045453 | 13 | 46379550 | 46379461 | -1 | U6 |
| ENSBTAG00000013899 | 13 | 46478656 | 46389229 | 1 | LARP4B |
| ENSBTAG00000051962 | 13 | 46454850 | 46454449 | -1 |  |
| ENSBTAG00000006531 | 13 | 46818194 | 46527612 | 1 | DIP2C |
| ENSBTAG00000053954 | 13 | 46729149 | 46729069 | -1 | bta-mir-2285m-2 |
| ENSBTAG00000048436 | 13 | 46756166 | 46756060 | -1 | U6 |
| ENSBTAG00000002578 | 13 | 46913156 | 46836953 | -1 | ZMYND11 |
| ENSBTAG00000048903 | 13 | 47058759 | 47039699 | 1 | PRNP |
| ENSBTAG00000050866 | 13 | 47056465 | 47056079 | -1 |  |
| ENSBTAG00000011010 | 13 | 47091045 | 47086007 | 1 | PRND |
| ENSBTAG00000017346 | 13 | 47157586 | 47134626 | -1 | RASSF2 |
| ENSBTAG00000049515 | 13 | 47195743 | 47195637 | -1 | U6 |
| ENSBTAG00000051124 | 13 | 47202587 | 47201583 | 1 |  |
| ENSBTAG00000032366 | 13 | 47348038 | 47209404 | -1 | SLC23A2 |
| ENSBTAG00000006063 | 13 | 47429786 | 47421389 | -1 | TMEM230 |
| ENSBTAG00000006063 | 13 | 47429786 | 47421389 | -1 | TMEM230 |
| ENSBTAG00000006065 | 13 | 47436478 | 47430406 | -1 | PCNA |
| ENSBTAG00000006066 | 13 | 47498715 | 47441800 | 1 | CDS2 |
| ENSBTAG00000042644 | 13 | 47487891 | 47487786 | 1 |  |
| ENSBTAG00000020668 | 13 | 47547502 | 47546444 | -1 |  |
| ENSBTAG00000015872 | 13 | 47558426 | 47550275 | -1 | PROKR2 |
| ENSBTAG00000008293 | 13 | 47778950 | 47720768 | -1 | GPCPD1 |
| ENSBTAG00000054005 | 13 | 47808405 | 47807353 | -1 |  |
| ENSBTAG00000051557 | 13 | 47866134 | 47827734 | 1 |  |
| ENSBTAG00000014048 | 13 | 48028742 | 47916971 | 1 | SHLD1 |
| ENSBTAG00000011782 | 13 | 48084157 | 48069187 | 1 | CHGB |
| ENSBTAG00000001314 | 13 | 48120755 | 48110221 | -1 | TRMT6 |
| ENSBTAG00000014623 | 13 | 48166976 | 48123958 | 1 | MCM8 |
| ENSBTAG00000000065 | 13 | 48209791 | 48186875 | 1 | CRLS1 |
| ENSBTAG00000000066 | 13 | 48233309 | 48213047 | -1 | LRRN4 |
| ENSBTAG00000020465 | 13 | 48312017 | 48251375 | -1 | FERMT1 |
| ENSBTAG00000005111 | 13 | 49185579 | 49151441 | 1 | BMP2 |
| ENSBTAG00000048604 | 13 | 49473233 | 49447109 | 1 |  |
| ENSBTAG00000007199 | 13 | 50548392 | 50547036 | -1 |  |
| ENSBTAG00000019811 | 13 | 50715147 | 50564351 | -1 | HAO1 |
| ENSBTAG00000019504 | 13 | 50932861 | 50910836 | 1 | ADRA1D |
| ENSBTAG00000001112 | 13 | 50985484 | 50950179 | -1 | SMOX |
| ENSBTAG00000054153 | 14 | 23397050 | 23395823 | -1 |  |
| ENSBTAG00000018570 | 14 | 23441042 | 23428404 | -1 | SDR16C5 |
| ENSBTAG00000040321 | 14 | 23502005 | 23475942 | -1 | SDR16C6 |
| ENSBTAG00000004924 | 14 | 23546868 | 23542370 | -1 | PENK |
| ENSBTAG00000043923 | 14 | 23815599 | 23815505 | 1 | U6 |
| ENSBTAG00000015637 | 14 | 23883121 | 23867153 | -1 | BPNT2 |
| ENSBTAG00000050550 | 14 | 24432635 | 24365744 | 1 | FAM110B |
| ENSBTAG00000047136 | 14 | 24496930 | 24496775 | 1 |  |
| ENSBTAG00000051748 | 14 | 24587122 | 24577095 | -1 |  |
| ENSBTAG00000009138 | 14 | 24624435 | 24587138 | 1 | UBXN2B |
| ENSBTAG00000005287 | 14 | 24675169 | 24664833 | -1 | CYP7A1 |
| ENSBTAG00000033622 | 14 | 24697033 | 24696870 | -1 | U1 |
| ENSBTAG00000019910 | 14 | 24763264 | 24728895 | 1 | SDCBP |
| ENSBTAG00000008958 | 14 | 24830983 | 24765312 | -1 | NSMAF |
| ENSBTAG00000004954 | 14 | 25258596 | 24946881 | -1 | TOX |
| ENSBTAG00000054521 | 14 | 25805643 | 25804210 | -1 |  |
| ENSBTAG00000017529 | 14 | 26038333 | 25956319 | -1 | CA8 |
| ENSBTAG00000000948 | 14 | 26253265 | 26181071 | 1 | RAB2A |
| ENSBTAG00000050813 | 14 | 26290277 | 26282271 | -1 |  |
| ENSBTAG00000021841 | 14 | 26487977 | 26361178 | 1 | CHD7 |
| ENSBTAG00000053382 | 14 | 26545427 | 26539963 | 1 |  |
| ENSBTAG00000052540 | 14 | 26751787 | 26751681 | -1 | U6 |
| ENSBTAG00000043978 | 14 | 27012590 | 26854867 | 1 | CLVS1 |
| ENSBTAG00000026283 | 14 | 27203495 | 27016060 | -1 | ASPH |
| ENSBTAG00000044415 | 14 | 27389328 | 27389218 | 1 | 5S_rRNA |
| ENSBTAG00000054400 | 14 | 27998050 | 27737786 | 1 | NKAIN3 |
| ENSBTAG00000045031 | 14 | 28027733 | 28027608 | 1 | SNORA70 |
| ENSBTAG00000007534 | 14 | 28070375 | 28047727 | -1 | GGH |
| ENSBTAG00000022471 | 14 | 28104548 | 28074682 | -1 | TTPA |
| ENSBTAG00000018557 | 14 | 28204989 | 28141155 | 1 | YTHDF3 |
| ENSBTAG00000055090 | 14 | 29116036 | 29113789 | -1 |  |
| ENSBTAG00000036421 | 14 | 29124466 | 29124356 | 1 | bta-mir-124a-2 |
| ENSBTAG00000001299 | 14 | 29460150 | 29199622 | -1 | CYP7B1 |
| ENSBTAG00000033173 | 14 | 29275673 | 29274588 | 1 | BHLHE22 |
| ENSBTAG00000044542 | 14 | 29580322 | 29580067 | 1 | 7SK |
| ENSBTAG00000054289 | 14 | 29809528 | 29809422 | -1 | U6 |
| ENSBTAG00000015901 | 14 | 30024834 | 30000050 | -1 | ARMC1 |
| ENSBTAG00000008629 | 14 | 30098890 | 30039506 | 1 | MTFR1 |
| ENSBTAG00000011614 | 14 | 30127822 | 30111614 | -1 | PDE7A |
| ENSBTAG00000054430 | 14 | 30141418 | 30141287 | -1 | SNORA72 |
| ENSBTAG00000021821 | 14 | 30145431 | 30144524 | -1 |  |
| ENSBTAG00000055026 | 14 | 30162307 | 30146796 | -1 |  |
| ENSBTAG00000053011 | 14 | 30213886 | 30211580 | -1 |  |
| ENSBTAG00000015229 | 14 | 30448182 | 30355171 | 1 | DNAJC5B |
| ENSBTAG00000021009 | 14 | 30495868 | 30469248 | 1 | TRIM55 |
| ENSBTAG00000016497 | 15 | 53086176 | 52890052 | -1 | FAM168A |
| ENSBTAG00000009830 | 15 | 53136353 | 53117190 | 1 | PLEKHB1 |
| ENSBTAG00000000025 | 15 | 53241816 | 53150637 | -1 | RAB6A |
| ENSBTAG00000042173 | 15 | 53258034 | 53257927 | -1 | U6 |
| ENSBTAG00000008347 | 15 | 53333980 | 53278739 | 1 | MRPL48 |
| ENSBTAG00000054199 | 15 | 53344586 | 53342076 | -1 | COA4 |
| ENSBTAG00000003679 | 15 | 53393878 | 53344671 | 1 | PAAF1 |
| ENSBTAG00000003682 | 15 | 53406519 | 53393791 | 1 | DNAJB13 |
| ENSBTAG00000003692 | 15 | 53416245 | 53409782 | -1 | UCP2 |
| ENSBTAG00000005259 | 15 | 53451256 | 53440626 | -1 | UCP3 |
| ENSBTAG00000002907 | 15 | 53526389 | 53454345 | -1 | C2CD3 |
| ENSBTAG00000051952 | 15 | 53484226 | 53484129 | -1 | 5S_rRNA |
| ENSBTAG00000054229 | 15 | 53579820 | 53552619 | -1 | C2CD3 |
| ENSBTAG00000049655 | 15 | 53555073 | 53554001 | -1 |  |
| ENSBTAG00000027612 | 15 | 53629168 | 53580198 | 1 | PPME1 |
| ENSBTAG00000006579 | 15 | 53681795 | 53640849 | -1 | P4HA3 |
| ENSBTAG00000027610 | 15 | 53690661 | 53690341 | 1 |  |
| ENSBTAG00000000770 | 15 | 53759583 | 53707096 | -1 | PGM2L1 |
| ENSBTAG00000054292 | 15 | 53734263 | 53734157 | 1 | U6 |
| ENSBTAG00000011873 | 15 | 53809595 | 53809296 | -1 | KCNE3 |
| ENSBTAG00000008227 | 15 | 53839967 | 53838026 | -1 | LIPT2 |
| ENSBTAG00000016869 | 15 | 53953518 | 53909173 | 1 | POLD3 |
| ENSBTAG00000021306 | 15 | 54042826 | 54008949 | -1 | CHRDL2 |
| ENSBTAG00000000727 | 15 | 54147686 | 54063709 | 1 | RNF169 |
| ENSBTAG00000052876 | 15 | 54067858 | 54067752 | -1 | U6 |
| ENSBTAG00000054207 | 15 | 54077414 | 54076404 | -1 |  |
| ENSBTAG00000042319 | 15 | 54113515 | 54113379 | 1 |  |
| ENSBTAG00000006610 | 15 | 54223146 | 54155681 | -1 | XRRA1 |
| ENSBTAG00000006644 | 15 | 54247190 | 54223258 | 1 | SPCS2 |
| ENSBTAG00000025931 | 15 | 54267975 | 54252856 | 1 | NEU3 |
| ENSBTAG00000047344 | 15 | 54314615 | 54313650 | -1 | OR2AT2C |
| ENSBTAG00000048088 | 15 | 54360992 | 54359456 | -1 |  |
| ENSBTAG00000015596 | 15 | 54427957 | 54375514 | 1 | SLCO2B1 |
| ENSBTAG00000019622 | 15 | 54463034 | 54461865 | 1 | TPBGL |
| ENSBTAG00000020485 | 15 | 54553148 | 54480057 | -1 | ARRB1 |
| ENSBTAG00000029833 | 15 | 54537364 | 54537263 | -1 | bta-mir-326 |
| ENSBTAG00000005620 | 15 | 54598584 | 54593621 | 1 | RPS3 |
| ENSBTAG00000042649 | 15 | 54594386 | 54594237 | 1 | SNORD15 |
| ENSBTAG00000042916 | 15 | 54597855 | 54597712 | 1 | SNORD15 |
| ENSBTAG00000015087 | 15 | 54610635 | 54604155 | -1 | KLHL35 |
| ENSBTAG00000031777 | 15 | 54706765 | 54615540 | -1 | GDPD5 |
| ENSBTAG00000001027 | 15 | 54748417 | 54737997 | 1 | SERPINH1 |
| ENSBTAG00000001028 | 15 | 54846909 | 54763503 | -1 | MAP6 |
| ENSBTAG00000049519 | 15 | 54897038 | 54884911 | 1 | MOGAT2 |
| ENSBTAG00000053794 | 15 | 54898516 | 54896121 | 1 |  |
| ENSBTAG00000047111 | 15 | 54955595 | 54940726 | 1 |  |
| ENSBTAG00000017443 | 15 | 54997831 | 54984012 | 1 |  |
| ENSBTAG00000015091 | 15 | 55048724 | 55030396 | 1 |  |
| ENSBTAG00000052936 | 15 | 55075615 | 55068987 | -1 |  |
| ENSBTAG00000037483 | 15 | 55124658 | 55112737 | 1 | MOGAT2 |
| ENSBTAG00000016355 | 15 | 55539167 | 55160620 | 1 | UVRAG |
| ENSBTAG00000010820 | 15 | 55624948 | 55605299 | -1 | WNT11 |
| ENSBTAG00000050371 | 15 | 55627955 | 55625742 | 1 |  |
| ENSBTAG00000051011 | 15 | 55652571 | 55649776 | -1 |  |
| ENSBTAG00000031609 | 15 | 55791234 | 55762482 | -1 | THAP12 |
| ENSBTAG00000052297 | 15 | 55837032 | 55791610 | 1 | GVQW3 |
| ENSBTAG00000010826 | 15 | 55933677 | 55854695 | 1 | EMSY |
| ENSBTAG00000048805 | 15 | 56025709 | 56020063 | -1 |  |
| ENSBTAG00000001301 | 15 | 56057707 | 56045705 | -1 | LRRC32 |
| ENSBTAG00000051627 | 15 | 56080761 | 56074822 | -1 |  |
| ENSBTAG00000001537 | 15 | 56193003 | 56179611 | 1 | TSKU |
| ENSBTAG00000017218 | 15 | 56403904 | 56246885 | 1 | ACER3 |
| ENSBTAG00000052260 | 15 | 56340221 | 56336602 | -1 |  |
| ENSBTAG00000008557 | 15 | 56340810 | 56338774 | -1 |  |
| ENSBTAG00000054600 | 15 | 56426088 | 56425386 | -1 |  |
| ENSBTAG00000047129 | 15 | 56455145 | 56449007 | 1 | B3GNT6 |
| ENSBTAG00000005034 | 15 | 56530219 | 56472805 | 1 | CAPN5 |
| ENSBTAG00000003955 | 15 | 56637170 | 56532192 | 1 | MYO7A |
| ENSBTAG00000021855 | 15 | 57352721 | 56896688 | 1 | ANO3 |
| ENSBTAG00000047903 | 15 | 56943156 | 56943021 | 1 |  |
| ENSBTAG00000027126 | 15 | 57284289 | 57270887 | -1 | MUC15 |
| ENSBTAG00000021858 | 15 | 57415989 | 57364699 | -1 | SLC5A12 |
| ENSBTAG00000006676 | 15 | 57707587 | 57703872 | 1 | FIBIN |
| ENSBTAG00000014847 | 15 | 57839046 | 57758972 | 1 | BBOX1 |
| ENSBTAG00000043814 | 15 | 57799394 | 57799281 | -1 | 5S_rRNA |
| ENSBTAG00000021346 | 15 | 58088286 | 58043890 | -1 | CCDC34 |
| ENSBTAG00000002606 | 15 | 58197387 | 58091974 | -1 | LGR4 |
| ENSBTAG00000001460 | 15 | 58224553 | 58214580 | -1 | LIN7C |
| ENSBTAG00000008134 | 15 | 58422131 | 58385523 | -1 | BDNF |
| ENSBTAG00000002117 | 15 | 58806311 | 58720346 | -1 | KIF18A |
| ENSBTAG00000003361 | 15 | 59021213 | 58806394 | 1 | METTL15 |
| ENSBTAG00000049088 | 15 | 59208354 | 59208159 | -1 |  |
| ENSBTAG00000044677 | 15 | 60094215 | 60093944 | -1 | 7SK |
| ENSBTAG00000045944 | 15 | 60635325 | 60635206 | -1 | 5S_rRNA |
| ENSBTAG00000020793 | 15 | 60736043 | 60728577 | -1 | KCNA4 |
| ENSBTAG00000010889 | 15 | 60980910 | 60976952 | 1 | FSHB |
| ENSBTAG00000012417 | 15 | 61061504 | 61047198 | 1 | ARL14EP |
| ENSBTAG00000020956 | 15 | 61371379 | 61161268 | -1 | MPPED2 |
| ENSBTAG00000006060 | 15 | 62145149 | 61692631 | -1 | DCDC1 |
| ENSBTAG00000050888 | 15 | 62220968 | 62144539 | 1 | DNAJC24 |
| ENSBTAG00000000475 | 15 | 62296459 | 62222483 | -1 | IMMP1L |
| ENSBTAG00000023831 | 15 | 62553952 | 62296511 | 1 | ELP4 |
| ENSBTAG00000004561 | 15 | 62588024 | 62560118 | -1 | PAX6 |
| ENSBTAG00000053505 | 15 | 62824377 | 62765428 | -1 |  |
| ENSBTAG00000052762 | 15 | 62863425 | 62854355 | -1 |  |
| ENSBTAG00000047362 | 15 | 62867520 | 62854463 | 1 | RCN1 |
| ENSBTAG00000052796 | 15 | 63067012 | 62873613 | -1 |  |
| ENSBTAG00000047268 | 15 | 63168835 | 63118358 | -1 | WT1 |
| ENSBTAG00000052383 | 15 | 63169060 | 63168951 | 1 |  |
| ENSBTAG00000052550 | 15 | 63169639 | 63169511 | 1 |  |
| ENSBTAG00000053465 | 15 | 63170193 | 63169962 | 1 |  |
| ENSBTAG00000054308 | 15 | 63170357 | 63170236 | 1 |  |
| ENSBTAG00000052824 | 15 | 63172371 | 63172250 | 1 |  |
| ENSBTAG00000050170 | 15 | 63173125 | 63172838 | 1 |  |
| ENSBTAG00000052000 | 15 | 63173496 | 63173225 | 1 |  |
| ENSBTAG00000005338 | 15 | 63316294 | 63298794 | 1 | EIF3M |
| ENSBTAG00000049128 | 15 | 63331135 | 63330758 | -1 |  |
| ENSBTAG00000050304 | 15 | 63358589 | 63342193 | -1 |  |
| ENSBTAG00000054030 | 15 | 63432268 | 63389114 | -1 |  |
| ENSBTAG00000050844 | 15 | 63515243 | 63510596 | -1 |  |
| ENSBTAG00000020731 | 15 | 63530238 | 63515385 | 1 | PRRG4 |
| ENSBTAG00000014399 | 15 | 63634185 | 63556795 | 1 | QSER1 |
| ENSBTAG00000021809 | 15 | 63688683 | 63659355 | 1 | DEPDC7 |
| ENSBTAG00000008613 | 15 | 63716137 | 63686064 | 1 | TCP11L1 |
| ENSBTAG00000044774 | 15 | 63700388 | 63700296 | 1 | U6 |
| ENSBTAG00000008617 | 15 | 63789775 | 63722373 | -1 | CSTF3 |
| ENSBTAG00000042111 | 15 | 63855088 | 63854986 | -1 | U6 |
| ENSBTAG00000014884 | 15 | 63962613 | 63908514 | 1 | HIPK3 |
| ENSBTAG00000012846 | 15 | 64306981 | 63989009 | 1 | KIAA1549L |
| ENSBTAG00000027577 | 15 | 64334453 | 64332509 | -1 | C15H11orf91 |
| ENSBTAG00000002302 | 15 | 64368108 | 64346582 | -1 | CD59 |
| ENSBTAG00000010071 | 15 | 64408465 | 64374161 | -1 | FBXO3 |
| ENSBTAG00000006951 | 15 | 64485114 | 64472394 | -1 | LMO2 |
| ENSBTAG00000016744 | 15 | 64697021 | 64661600 | 1 | CAPRIN1 |
| ENSBTAG00000016747 | 15 | 64740844 | 64702013 | 1 | NAT10 |
| ENSBTAG00000005923 | 15 | 64932891 | 64746521 | -1 | ABTB2 |
| ENSBTAG00000020980 | 15 | 65011223 | 64975271 | 1 | CAT |
| ENSBTAG00000012460 | 15 | 65067265 | 65019976 | -1 | ELF5 |
| ENSBTAG00000017150 | 15 | 65227768 | 65186293 | 1 | EHF |
| ENSBTAG00000018257 | 15 | 65427122 | 65403011 | -1 | APIP |
| ENSBTAG00000018261 | 15 | 65502624 | 65427261 | 1 | PDHX |
| ENSBTAG00000044322 | 15 | 65460366 | 65460265 | 1 |  |
| ENSBTAG00000011578 | 15 | 65737462 | 65649433 | 1 | CD44 |
| ENSBTAG00000012628 | 15 | 65935522 | 65776406 | -1 | SLC1A2 |
| ENSBTAG00000012630 | 15 | 66028384 | 65950563 | -1 | PAMR1 |
| ENSBTAG00000047083 | 15 | 66126201 | 66124888 | 1 | FJX1 |
| ENSBTAG00000037389 | 15 | 66291995 | 66181718 | 1 | TRIM44 |
| ENSBTAG00000044158 | 15 | 66702598 | 66504294 | 1 | LDLRAD3 |
| ENSBTAG00000019045 | 15 | 66755759 | 66742629 | -1 | COMMD9 |
| ENSBTAG00000021029 | 15 | 66918831 | 66763111 | 1 | PRR5L |
| ENSBTAG00000036009 | 15 | 66967540 | 66946444 | -1 | TRAF6 |
| ENSBTAG00000040293 | 15 | 67008473 | 67005342 | 1 | RAG1 |
| ENSBTAG00000031309 | 15 | 67027721 | 67019957 | -1 | RAG2 |
| ENSBTAG00000021611 | 15 | 67089356 | 67022721 | 1 | IFTAP |
| ENSBTAG00000044849 | 15 | 67052977 | 67052906 | -1 | U6 |
| ENSBTAG00000051625 | 16 | 75557325 | 75556041 | 1 |  |
| ENSBTAG00000049747 | 16 | 75573334 | 75572249 | -1 |  |
| ENSBTAG00000051785 | 16 | 75577131 | 75577042 | 1 | bta-mir-29b-2 |
| ENSBTAG00000052678 | 16 | 75577693 | 75577606 | 1 | bta-mir-29c |
| ENSBTAG00000005397 | 16 | 75621057 | 75582021 | -1 | CD46 |
| ENSBTAG00000052620 | 16 | 75615244 | 75614111 | 1 |  |
| ENSBTAG00000051920 | 16 | 75660459 | 75660370 | 1 | bta-mir-29b-2 |
| ENSBTAG00000051791 | 16 | 75661000 | 75660913 | 1 | bta-mir-29d |
| ENSBTAG00000052901 | 16 | 75736094 | 75690225 | -1 |  |
| ENSBTAG00000050070 | 16 | 75810353 | 75768942 | -1 |  |
| ENSBTAG00000053010 | 16 | 75841153 | 75825896 | -1 |  |
| ENSBTAG00000030910 | 16 | 75887017 | 75850895 | -1 |  |
| ENSBTAG00000048618 | 16 | 75928905 | 75921020 | 1 |  |
| ENSBTAG00000000070 | 16 | 75966734 | 75944878 | 1 | F13B |
| ENSBTAG00000038171 | 16 | 76010930 | 75977678 | 1 | CFHR5 |
| ENSBTAG00000007860 | 16 | 76082066 | 76021939 | -1 | ASPM |
| ENSBTAG00000002291 | 16 | 76135565 | 76102444 | -1 | ZBTB41 |
| ENSBTAG00000008944 | 16 | 76401805 | 76188124 | 1 | CRB1 |
| ENSBTAG00000044926 | 16 | 76424685 | 76424616 | -1 | bta-mir-2284n |
| ENSBTAG00000012433 | 16 | 76747186 | 76482363 | -1 | DENND1B |
| ENSBTAG00000051004 | 16 | 76853904 | 76847270 | 1 | C16H1orf53 |
| ENSBTAG00000013499 | 16 | 76878665 | 76858671 | 1 | LHX9 |
| ENSBTAG00000016943 | 16 | 77212053 | 77074385 | 1 | NEK7 |
| ENSBTAG00000049638 | 16 | 77228438 | 77225125 | 1 |  |
| ENSBTAG00000019890 | 16 | 77427160 | 77406070 | -1 | ATP6V1G3 |
| ENSBTAG00000054124 | 16 | 77520229 | 77516694 | 1 |  |
| ENSBTAG00000049945 | 16 | 77597927 | 77540164 | 1 |  |
| ENSBTAG00000023144 | 16 | 77669271 | 77600496 | 1 | PTPRC |
| ENSBTAG00000049692 | 16 | 77626782 | 77626676 | -1 | U6 |
| ENSBTAG00000046345 | 16 | 77634326 | 77633793 | -1 |  |
| ENSBTAG00000029993 | 16 | 77762773 | 77762664 | -1 | bta-mir-181b-1 |
| ENSBTAG00000029849 | 16 | 77762945 | 77762836 | -1 | bta-mir-181a-1 |
| ENSBTAG00000050713 | 16 | 77848960 | 77762906 | -1 |  |
| ENSBTAG00000055055 | 16 | 78409150 | 78409091 | 1 | bta-mir-2285ay |
| ENSBTAG00000009265 | 16 | 78957232 | 78828931 | 1 | NR5A2 |
| ENSBTAG00000052560 | 16 | 79092533 | 79090731 | 1 |  |
| ENSBTAG00000047428 | 16 | 79121031 | 79118410 | -1 | ZNF281 |
| ENSBTAG00000055184 | 16 | 79140977 | 79121510 | 1 |  |
| ENSBTAG00000052684 | 16 | 79142756 | 79141150 | 1 |  |
| ENSBTAG00000052549 | 16 | 79212340 | 79212234 | 1 | U6 |
| ENSBTAG00000021874 | 16 | 79278728 | 79237466 | -1 | KIF14 |
| ENSBTAG00000048732 | 16 | 79238643 | 79238537 | 1 | U6 |
| ENSBTAG00000020563 | 16 | 79317794 | 79300366 | -1 | DDX59 |
| ENSBTAG00000008153 | 16 | 79473155 | 79375975 | 1 | CAMSAP2 |
| ENSBTAG00000053928 | 16 | 79481332 | 79480208 | 1 | GPR25 |
| ENSBTAG00000047972 | 16 | 79515409 | 79497446 | 1 | INAVA |
| ENSBTAG00000053707 | 16 | 79540935 | 79521258 | 1 |  |
| ENSBTAG00000047274 | 16 | 79581778 | 79548497 | -1 | KIF21B |
| ENSBTAG00000047491 | 16 | 79660665 | 79599108 | -1 | CACNA1S |
| ENSBTAG00000051448 | 16 | 79688109 | 79675900 | -1 | TMEM9 |
| ENSBTAG00000046177 | 16 | 79750062 | 79714751 | 1 | IGFN1 |
| ENSBTAG00000046350 | 16 | 79824967 | 79787223 | 1 | PKP1 |
| ENSBTAG00000019931 | 16 | 79853936 | 79851015 | 1 |  |
| ENSBTAG00000019927 | 16 | 79865626 | 79860458 | 1 | CYB5R1 |
| ENSBTAG00000009727 | 16 | 79883989 | 79870488 | 1 | ADIPOR1 |
| ENSBTAG00000009726 | 16 | 79911514 | 79889001 | 1 | KLHL12 |
| ENSBTAG00000013560 | 16 | 79917702 | 79913789 | 1 | RABIF |
| ENSBTAG00000016189 | 16 | 79940606 | 79938517 | -1 |  |
| ENSBTAG00000006175 | 16 | 80022792 | 79949862 | 1 | KDM5B |
| ENSBTAG00000051303 | 16 | 80025651 | 80025558 | 1 | bta-mir-10180 |
| ENSBTAG00000047648 | 16 | 80130039 | 80036572 | 1 | SYT2 |
| ENSBTAG00000049657 | 16 | 80134176 | 80130060 | -1 |  |
| ENSBTAG00000011772 | 16 | 80301780 | 80138744 | -1 | PPP1R12B |
| ENSBTAG00000033994 | 16 | 80270426 | 80270236 | 1 | U2 |
| ENSBTAG00000004790 | 16 | 80309745 | 80305103 | 1 | UBE2T |
| ENSBTAG00000004789 | 16 | 80399065 | 80312915 | -1 | LGR6 |
| ENSBTAG00000047073 | 16 | 80405323 | 80402498 | -1 |  |
| ENSBTAG00000003016 | 16 | 80428748 | 80420337 | 1 | PTPN7 |
| ENSBTAG00000006574 | 16 | 80436465 | 80429842 | 1 | ARL8A |
| ENSBTAG00000014992 | 16 | 80444291 | 80440023 | -1 | GPR37L1 |
| ENSBTAG00000052377 | 16 | 80448567 | 80448310 | -1 |  |
| ENSBTAG00000049594 | 16 | 80455108 | 80452399 | -1 |  |
| ENSBTAG00000008756 | 16 | 80507964 | 80502933 | -1 | ELF3 |
| ENSBTAG00000013726 | 16 | 80522913 | 80509611 | -1 | RNPEP |
| ENSBTAG00000047059 | 16 | 80543813 | 80532691 | -1 | TIMM17A |
| ENSBTAG00000021576 | 16 | 80576112 | 80548214 | 1 | LMOD1 |
| ENSBTAG00000050933 | 16 | 80581443 | 80578402 | -1 | SHISA4 |
| ENSBTAG00000054740 | 16 | 80586083 | 80585034 | 1 |  |
| ENSBTAG00000004452 | 16 | 80629377 | 80589408 | -1 | IPO9 |
| ENSBTAG00000054727 | 16 | 80634826 | 80633603 | -1 |  |
| ENSBTAG00000021919 | 16 | 80688965 | 80636726 | -1 | NAV1 |
| ENSBTAG00000049173 | 16 | 80757039 | 80752893 | 1 |  |
| ENSBTAG00000050642 | 16 | 80784882 | 80782869 | -1 |  |
| ENSBTAG00000016057 | 16 | 80867559 | 80847188 | 1 | CSRP1 |
| ENSBTAG00000053086 | 16 | 80877463 | 80877092 | 1 | PHLDA3 |
| ENSBTAG00000047231 | 16 | 80906695 | 80897787 | 1 | TNNI1 |
| ENSBTAG00000024663 | 16 | 80929327 | 80916091 | 1 | LAD1 |
| ENSBTAG00000045789 | 16 | 80942857 | 80934811 | 1 | TNNT2 |
| ENSBTAG00000001117 | 17 | 33168247 | 33093747 | 1 | ANKRD50 |
| ENSBTAG00000052376 | 17 | 34106725 | 33991881 | -1 |  |
| ENSBTAG00000021245 | 17 | 34357245 | 34353125 | -1 | SPRY1 |
| ENSBTAG00000052988 | 17 | 34358736 | 34358675 | -1 | bta-mir-12036 |
| ENSBTAG00000000125 | 17 | 34747076 | 34422664 | -1 | SPATA5 |
| ENSBTAG00000042522 | 17 | 34697537 | 34697409 | -1 |  |
| ENSBTAG00000005695 | 17 | 34807159 | 34747124 | 1 | NUDT6 |
| ENSBTAG00000043841 | 17 | 34771997 | 34771860 | 1 | U4 |
| ENSBTAG00000005691 | 17 | 34860849 | 34801330 | -1 | FGF2 |
| ENSBTAG00000007564 | 17 | 34938688 | 34936426 | -1 | BBS12 |
| ENSBTAG00000016147 | 17 | 34953270 | 34949127 | 1 | CETN4 |
| ENSBTAG00000012368 | 17 | 35070765 | 35062970 | 1 | IL21 |
| ENSBTAG00000020883 | 17 | 35222747 | 35217965 | 1 | IL2 |
| ENSBTAG00000046515 | 17 | 35266174 | 35266061 | -1 | U6 |
| ENSBTAG00000046191 | 17 | 35307547 | 35298887 | -1 | ADAD1 |
| ENSBTAG00000048798 | 17 | 35476298 | 35321748 | -1 |  |
| ENSBTAG00000051886 | 17 | 35505549 | 35478947 | -1 |  |
| ENSBTAG00000019021 | 17 | 35800211 | 35728366 | 1 | TRPC3 |
| ENSBTAG00000047595 | 17 | 37189201 | 36260758 | 1 | FSTL5 |
| ENSBTAG00000043467 | 17 | 36425507 | 36425393 | -1 | SNORA72 |
| ENSBTAG00000016059 | 17 | 37485360 | 37484575 | -1 |  |
| ENSBTAG00000048198 | 17 | 38240376 | 38240258 | 1 | 5S_rRNA |
| ENSBTAG00000012450 | 17 | 39902881 | 39633239 | -1 | RAPGEF2 |
| ENSBTAG00000029127 | 17 | 39977317 | 39977206 | -1 | 5S_rRNA |
| ENSBTAG00000033547 | 17 | 40116757 | 39983282 | 1 | C17H4orf45 |
| ENSBTAG00000011970 | 17 | 40236065 | 40112134 | -1 | FNIP2 |
| ENSBTAG00000016680 | 17 | 40292370 | 40279436 | 1 | PPID |
| ENSBTAG00000016679 | 17 | 40337562 | 40285083 | -1 | ETFDH |
| ENSBTAG00000053834 | 17 | 40321615 | 40321022 | 1 |  |
| ENSBTAG00000033486 | 17 | 40340549 | 40337328 | 1 | C17H4orf46 |
| ENSBTAG00000010306 | 17 | 40475237 | 40348027 | -1 | RXFP1 |
| ENSBTAG00000052985 | 17 | 40686515 | 40683568 | -1 |  |
| ENSBTAG00000019229 | 17 | 40745537 | 40697162 | -1 | TMEM144 |
| ENSBTAG00000055144 | 17 | 40721841 | 40721416 | 1 |  |
| ENSBTAG00000052509 | 17 | 40794750 | 40783167 | -1 |  |
| ENSBTAG00000017069 | 17 | 40871776 | 40795189 | 1 | GASK1B |
| ENSBTAG00000043594 | 17 | 40844944 | 40844833 | -1 | 5S_rRNA |
| ENSBTAG00000004775 | 17 | 41971774 | 41785259 | -1 | GRIA2 |
| ENSBTAG00000021764 | 17 | 42110395 | 42021088 | -1 | GLRB |
| ENSBTAG00000043959 | 17 | 42478276 | 42227069 | 1 | PDGFC |
| ENSBTAG00000054886 | 17 | 42587383 | 42581950 | 1 |  |
| ENSBTAG00000049769 | 17 | 42589090 | 42588038 | 1 |  |
| ENSBTAG00000011063 | 17 | 43381605 | 43364999 | 1 | CTSO |
| ENSBTAG00000042385 | 17 | 43381209 | 43381106 | 1 | U6 |
| ENSBTAG00000011062 | 17 | 43403747 | 43386894 | -1 | TDO2 |
| ENSBTAG00000044109 | 17 | 43491735 | 43437239 | 1 | ASIC5 |
| ENSBTAG00000003840 | 17 | 43577750 | 43514978 | -1 | GUCY1B1 |
| ENSBTAG00000051096 | 17 | 43530307 | 43528091 | 1 |  |
| ENSBTAG00000014576 | 17 | 43673434 | 43601257 | -1 | GUCY1A1 |
| ENSBTAG00000049458 | 17 | 43797868 | 43726286 | 1 |  |
| ENSBTAG00000005240 | 17 | 43977394 | 43972110 | -1 | ZNF605 |
| ENSBTAG00000053385 | 17 | 44001483 | 43996363 | -1 |  |
| ENSBTAG00000054451 | 17 | 44008029 | 44000826 | 1 |  |
| ENSBTAG00000053029 | 17 | 44029448 | 44023784 | 1 | ZNF26 |
| ENSBTAG00000013001 | 17 | 44068639 | 44044076 | 1 | ZNF84 |
| ENSBTAG00000040072 | 17 | 44121402 | 44110583 | 1 | ZNF140 |
| ENSBTAG00000054305 | 17 | 44117247 | 44111747 | 1 |  |
| ENSBTAG00000038806 | 17 | 44147730 | 44146467 | -1 | ZNF891 |
| ENSBTAG00000039242 | 17 | 44169024 | 44156961 | 1 | ZNF10 |
| ENSBTAG00000040336 | 17 | 44221154 | 44195034 | 1 | ZNF268 |
| ENSBTAG00000039316 | 17 | 44226983 | 44224575 | 1 | ZNF268 |
| ENSBTAG00000006753 | 17 | 44259224 | 44257597 | 1 | MBD3L1 |
| ENSBTAG00000006758 | 17 | 44272435 | 44271152 | -1 |  |
| ENSBTAG00000026626 | 17 | 44291931 | 44283822 | -1 | ANHX |
| ENSBTAG00000013951 | 17 | 44411465 | 44386512 | 1 | CHFR |
| ENSBTAG00000009755 | 17 | 44470280 | 44423383 | 1 | GOLGA3 |
| ENSBTAG00000033339 | 17 | 44499871 | 44477204 | 1 | ANKLE2 |
| ENSBTAG00000007513 | 17 | 44510533 | 44502890 | -1 | PGAM5 |
| ENSBTAG00000054166 | 17 | 44510673 | 44506135 | 1 |  |
| ENSBTAG00000007510 | 17 | 44522964 | 44512664 | -1 | PXMP2 |
| ENSBTAG00000054055 | 17 | 44518591 | 44515473 | 1 |  |
| ENSBTAG00000000590 | 17 | 44583195 | 44523016 | 1 | POLE |
| ENSBTAG00000053884 | 17 | 44525002 | 44524896 | -1 | U6 |
| ENSBTAG00000054322 | 18 | 59447261 | 59444868 | -1 |  |
| ENSBTAG00000050562 | 18 | 59526105 | 59523031 | -1 |  |
| ENSBTAG00000052631 | 18 | 59530147 | 59528963 | -1 |  |
| ENSBTAG00000049645 | 18 | 59558120 | 59556687 | -1 |  |
| ENSBTAG00000021433 | 18 | 59606597 | 59604000 | -1 |  |
| ENSBTAG00000054418 | 18 | 59918042 | 59915850 | -1 |  |
| ENSBTAG00000054088 | 18 | 59949659 | 59920134 | -1 |  |
| ENSBTAG00000025023 | 18 | 59964069 | 59963092 | 1 |  |
| ENSBTAG00000045985 | 18 | 59970062 | 59968692 | 1 |  |
| ENSBTAG00000051883 | 18 | 59995640 | 59994045 | 1 |  |
| ENSBTAG00000004925 | 18 | 60019726 | 60018479 | 1 |  |
| ENSBTAG00000055311 | 18 | 60043497 | 60041848 | 1 |  |
| ENSBTAG00000038674 | 18 | 60116948 | 60074952 | 1 | ZNF677 |
| ENSBTAG00000053759 | 18 | 60259210 | 60094269 | -1 |  |
| ENSBTAG00000047301 | 18 | 60112775 | 60111587 | -1 |  |
| ENSBTAG00000030440 | 18 | 60160418 | 60158850 | 1 |  |
| ENSBTAG00000030444 | 18 | 60201208 | 60198992 | -1 |  |
| ENSBTAG00000037699 | 18 | 60231052 | 60229001 | -1 |  |
| ENSBTAG00000034090 | 18 | 60333737 | 60268945 | -1 |  |
| ENSBTAG00000053237 | 18 | 60557911 | 60344098 | -1 |  |
| ENSBTAG00000053293 | 18 | 60391208 | 60353332 | -1 |  |
| ENSBTAG00000045581 | 18 | 60389389 | 60378959 | -1 |  |
| ENSBTAG00000053330 | 18 | 60410948 | 60410091 | -1 |  |
| ENSBTAG00000038702 | 18 | 60425791 | 60418125 | -1 |  |
| ENSBTAG00000033642 | 18 | 60653111 | 60479470 | -1 |  |
| ENSBTAG00000048990 | 18 | 60500528 | 60486740 | -1 |  |
| ENSBTAG00000051088 | 18 | 60543068 | 60528443 | -1 |  |
| ENSBTAG00000038755 | 18 | 60608616 | 60530988 | -1 |  |
| ENSBTAG00000011441 | 18 | 60656231 | 60655738 | -1 |  |
| ENSBTAG00000039849 | 18 | 60684957 | 60682643 | -1 |  |
| ENSBTAG00000016513 | 18 | 60749312 | 60741854 | -1 | ZNF331 |
| ENSBTAG00000049523 | 18 | 60754774 | 60751023 | -1 |  |
| ENSBTAG00000002290 | 18 | 60768024 | 60759305 | -1 |  |
| ENSBTAG00000040392 | 18 | 60866029 | 60856348 | -1 | MGC139164 |
| ENSBTAG00000036392 | 18 | 60903249 | 60903171 | 1 | bta-mir-371 |
| ENSBTAG00000038149 | 18 | 60925131 | 60905906 | -1 | NLRP12 |
| ENSBTAG00000036224 | 18 | 61018636 | 61009582 | 1 | MGC157082 |
| ENSBTAG00000014953 | 18 | 61083999 | 61067997 | -1 |  |
| ENSBTAG00000000336 | 18 | 61171888 | 61158271 | 1 |  |
| ENSBTAG00000009171 | 18 | 61189526 | 61180951 | 1 |  |
| ENSBTAG00000015061 | 18 | 61224598 | 61209482 | 1 |  |
| ENSBTAG00000014328 | 18 | 61255678 | 61238819 | 1 | MGC138914 |
| ENSBTAG00000054918 | 18 | 61273471 | 61272049 | 1 |  |
| ENSBTAG00000013345 | 18 | 61290145 | 61284316 | 1 |  |
| ENSBTAG00000009364 | 18 | 61306285 | 61300943 | 1 |  |
| ENSBTAG00000015987 | 18 | 61333880 | 61322356 | 1 |  |
| ENSBTAG00000050073 | 18 | 61335095 | 61335031 | -1 | bta-mir-11977 |
| ENSBTAG00000051856 | 18 | 61418491 | 61410874 | 1 |  |
| ENSBTAG00000046961 | 18 | 61478161 | 61467834 | 1 |  |
| ENSBTAG00000051149 | 18 | 61484576 | 61483464 | -1 |  |
| ENSBTAG00000030416 | 18 | 61488199 | 61486796 | 1 |  |
| ENSBTAG00000015139 | 18 | 61507130 | 61501761 | 1 |  |
| ENSBTAG00000018152 | 18 | 61579533 | 61573948 | 1 | MYADM |
| ENSBTAG00000013550 | 18 | 61602873 | 61583328 | 1 | PRKCG |
| ENSBTAG00000007506 | 18 | 61622688 | 61607183 | 1 | CACNG7 |
| ENSBTAG00000007509 | 18 | 61651931 | 61637425 | 1 | CACNG8 |
| ENSBTAG00000037338 | 18 | 61651485 | 61651395 | 1 | bta-mir-935 |
| ENSBTAG00000021657 | 18 | 61675273 | 61659470 | 1 | CACNG6 |
| ENSBTAG00000002596 | 18 | 61717831 | 61698117 | -1 | VSTM1 |
| ENSBTAG00000051657 | 18 | 61736415 | 61733133 | -1 |  |
| ENSBTAG00000052278 | 18 | 61747381 | 61746452 | -1 |  |
| ENSBTAG00000004725 | 18 | 61796493 | 61771664 | -1 | NLRP9 |
| ENSBTAG00000053115 | 18 | 61812137 | 61811229 | 1 |  |
| ENSBTAG00000049631 | 18 | 61826626 | 61820707 | 1 |  |
| ENSBTAG00000049020 | 18 | 61851746 | 61844759 | 1 |  |
| ENSBTAG00000046996 | 18 | 61878669 | 61867768 | -1 | EPN1 |
| ENSBTAG00000002830 | 18 | 61892127 | 61879206 | -1 | U2AF2 |
| ENSBTAG00000011517 | 18 | 61897283 | 61893703 | -1 | CCDC106 |
| ENSBTAG00000045864 | 18 | 61899895 | 61899299 | -1 | ZNF581 |
| ENSBTAG00000047508 | 18 | 61901500 | 61900982 | -1 | ZNF580 |
| ENSBTAG00000012333 | 18 | 61960163 | 61915345 | -1 | ZNF524 |
| ENSBTAG00000016299 | 18 | 61924820 | 61921781 | 1 | ZNF784 |
| ENSBTAG00000030403 | 18 | 61947502 | 61941890 | 1 | FIZ1 |
| ENSBTAG00000051917 | 18 | 61959077 | 61954479 | 1 | ZNF579 |
| ENSBTAG00000006121 | 18 | 61990216 | 61982147 | 1 | SBK2 |
| ENSBTAG00000030885 | 18 | 62021041 | 61999364 | -1 | SSC5D |
| ENSBTAG00000043361 | 18 | 62002193 | 62002090 | -1 | U6 |
| ENSBTAG00000019051 | 18 | 62025035 | 62021731 | -1 | NAT14 |
| ENSBTAG00000038034 | 18 | 62031537 | 62025056 | -1 | ZNF628 |
| ENSBTAG00000051978 | 18 | 62051276 | 62042308 | 1 | C19orf85 |
| ENSBTAG00000050011 | 18 | 62052842 | 62048799 | -1 |  |
| ENSBTAG00000015950 | 18 | 62060249 | 62053844 | 1 | ISOC2 |
| ENSBTAG00000008932 | 18 | 62073582 | 62066756 | 1 | SHISA7 |
| ENSBTAG00000009211 | 18 | 62093134 | 62089250 | 1 | UBE2S |
| ENSBTAG00000023343 | 18 | 62101918 | 62099120 | -1 | RPL28 |
| ENSBTAG00000021057 | 18 | 62110115 | 62106899 | -1 | TMEM190 |
| ENSBTAG00000044157 | 18 | 62122389 | 62118907 | 1 |  |
| ENSBTAG00000007200 | 18 | 62126264 | 62124767 | 1 | COX6B2 |
| ENSBTAG00000014087 | 18 | 62138720 | 62131439 | -1 | KMT5C |
| ENSBTAG00000022813 | 18 | 62167311 | 62159120 | 1 | TMEM150B |
| ENSBTAG00000015226 | 18 | 62190012 | 62167350 | -1 | BRSK1 |
| ENSBTAG00000051071 | 18 | 62191434 | 62190246 | -1 |  |
| ENSBTAG00000003644 | 18 | 62205672 | 62195226 | 1 | HSPBP1 |
| ENSBTAG00000049905 | 18 | 62212564 | 62206129 | -1 |  |
| ENSBTAG00000021157 | 18 | 62228726 | 62216820 | 1 | PPP6R1 |
| ENSBTAG00000052531 | 18 | 62222960 | 62222901 | 1 | bta-mir-7865 |
| ENSBTAG00000051212 | 18 | 62233357 | 62229686 | 1 | TMEM86B |
| ENSBTAG00000040559 | 18 | 62251375 | 62242461 | 1 | PTPRH |
| ENSBTAG00000002522 | 18 | 62259163 | 62252338 | 1 | SYT5 |
| ENSBTAG00000002515 | 18 | 62273071 | 62260692 | 1 | TNNI3 |
| ENSBTAG00000006419 | 18 | 62285833 | 62274658 | 1 | TNNT1 |
| ENSBTAG00000017847 | 18 | 62309936 | 62291434 | 1 | PPP1R12C |
| ENSBTAG00000009512 | 18 | 62323676 | 62310784 | -1 | EPS8L1 |
| ENSBTAG00000030393 | 18 | 62355536 | 62337525 | 1 | RDH13 |
| ENSBTAG00000050944 | 18 | 62370641 | 62363963 | 1 |  |
| ENSBTAG00000047175 | 18 | 62405845 | 62393610 | 1 |  |
| ENSBTAG00000047953 | 18 | 62438024 | 62409111 | -1 | NLRP2 |
| ENSBTAG00000046383 | 18 | 62454894 | 62453964 | -1 |  |
| ENSBTAG00000045854 | 18 | 62476548 | 62462232 | -1 |  |
| ENSBTAG00000047473 | 18 | 62504128 | 62490163 | 1 |  |
| ENSBTAG00000045529 | 18 | 62516410 | 62512088 | -1 | NCR1 |
| ENSBTAG00000021647 | 18 | 62537893 | 62527485 | -1 | FCAR |
| ENSBTAG00000039215 | 18 | 62552522 | 62542301 | -1 | KIR2DL5A |
| ENSBTAG00000039215 | 18 | 62552522 | 62542301 | -1 | KIR2DL5A |
| ENSBTAG00000054741 | 18 | 62561908 | 62554596 | -1 | KIR3DL1 |
| ENSBTAG00000022807 | 18 | 62594465 | 62587896 | -1 | KIR3DS1 |
| ENSBTAG00000045951 | 18 | 62636766 | 62625845 | -1 |  |
| ENSBTAG00000052880 | 18 | 62653396 | 62645561 | -1 |  |
| ENSBTAG00000054818 | 18 | 62662605 | 62655625 | -1 | KIR3DL2 |
| ENSBTAG00000054818 | 18 | 62662605 | 62655625 | -1 | KIR3DL2 |
| ENSBTAG00000054818 | 18 | 62662605 | 62655625 | -1 | KIR3DL2 |
| ENSBTAG00000049820 | 18 | 62699050 | 62671269 | -1 |  |
| ENSBTAG00000047270 | 18 | 62722641 | 62714565 | -1 |  |
| ENSBTAG00000045795 | 18 | 62761536 | 62754722 | -1 | KIR2DS1 |
| ENSBTAG00000045795 | 18 | 62761536 | 62754722 | -1 | KIR2DS1 |
| ENSBTAG00000039413 | 18 | 62798262 | 62793883 | -1 |  |
| ENSBTAG00000039086 | 18 | 62823366 | 62802729 | -1 |  |
| ENSBTAG00000026080 | 18 | 62859813 | 62817836 | -1 | LAIR1 |
| ENSBTAG00000003470 | 18 | 62875689 | 62862296 | 1 | TTYH1 |
| ENSBTAG00000011689 | 18 | 62888679 | 62878755 | 1 | LENG8 |
| ENSBTAG00000011693 | 18 | 62890331 | 62888724 | -1 | LENG9 |
| ENSBTAG00000045989 | 18 | 62891788 | 62891327 | -1 | CDC42EP5 |
| ENSBTAG00000052741 | 18 | 62897574 | 62892627 | -1 |  |
| ENSBTAG00000050536 | 18 | 62924121 | 62917599 | 1 |  |
| ENSBTAG00000048420 | 18 | 62942083 | 62937680 | 1 |  |
| ENSBTAG00000004043 | 18 | 62948849 | 62945785 | 1 |  |
| ENSBTAG00000038797 | 18 | 62974443 | 62970281 | 1 |  |
| ENSBTAG00000049178 | 18 | 63168770 | 63165062 | 1 |  |
| ENSBTAG00000006487 | 18 | 63204349 | 63196980 | -1 | RPS9 |
| ENSBTAG00000006486 | 18 | 63213014 | 63209077 | -1 | TSEN34 |
| ENSBTAG00000015908 | 18 | 63227739 | 63213542 | 1 | MBOAT7 |
| ENSBTAG00000020062 | 18 | 63238070 | 63227989 | 1 | TMC4 |
| ENSBTAG00000023333 | 18 | 63241746 | 63238408 | 1 | LENG1 |
| ENSBTAG00000014900 | 18 | 63257591 | 63241718 | -1 | CNOT3 |
| ENSBTAG00000007757 | 18 | 63271920 | 63260500 | -1 | PRPF31 |
| ENSBTAG00000007756 | 18 | 63279516 | 63271873 | 1 | TFPT |
| ENSBTAG00000007754 | 18 | 63282901 | 63279554 | -1 | NDUFA3 |
| ENSBTAG00000001051 | 18 | 63290397 | 63283496 | 1 | OSCAR |
| ENSBTAG00000045861 | 18 | 63303051 | 63294632 | 1 | TARM1 |
| ENSBTAG00000050838 | 18 | 63316280 | 63312581 | 1 |  |
| ENSBTAG00000054608 | 18 | 63328973 | 63327601 | 1 |  |
| ENSBTAG00000054213 | 18 | 63336857 | 63335943 | 1 |  |
| ENSBTAG00000051005 | 18 | 63361931 | 63361023 | 1 |  |
| ENSBTAG00000048343 | 18 | 63374447 | 63373080 | 1 |  |
| ENSBTAG00000050175 | 18 | 63382287 | 63381373 | 1 |  |
| ENSBTAG00000052498 | 18 | 63419332 | 63403065 | 1 |  |
| ENSBTAG00000006961 | 18 | 63449560 | 63423255 | -1 | NLRP13 |
| ENSBTAG00000006965 | 18 | 63475264 | 63455458 | 1 | NLRP8 |
| ENSBTAG00000013247 | 18 | 63493232 | 63478435 | 1 | NLRP5 |
| ENSBTAG00000002904 | 18 | 63512692 | 63496846 | -1 | ZNF787 |
| ENSBTAG00000046111 | 18 | 63535355 | 63526743 | 1 | ZNF444 |
| ENSBTAG00000055190 | 18 | 63551901 | 63548091 | 1 |  |
| ENSBTAG00000049186 | 18 | 63562726 | 63558439 | -1 |  |
| ENSBTAG00000038022 | 18 | 63576528 | 63573782 | 1 |  |
| ENSBTAG00000055035 | 18 | 63589948 | 63586494 | -1 |  |
| ENSBTAG00000050868 | 18 | 63599260 | 63598922 | -1 |  |
| ENSBTAG00000048593 | 18 | 63606851 | 63606513 | 1 |  |
| ENSBTAG00000048735 | 18 | 63655674 | 63655336 | -1 |  |
| ENSBTAG00000053137 | 18 | 63660253 | 63659915 | 1 |  |
| ENSBTAG00000050885 | 18 | 63678934 | 63678596 | 1 |  |
| ENSBTAG00000000803 | 18 | 63741526 | 63719243 | 1 | ZNF667 |
| ENSBTAG00000000801 | 18 | 63757996 | 63750840 | -1 | ZNF583 |
| ENSBTAG00000030348 | 18 | 63785937 | 63773648 | 1 | ZNF582 |
| ENSBTAG00000050395 | 18 | 63806654 | 63800079 | 1 |  |
| ENSBTAG00000054887 | 18 | 63819766 | 63806826 | 1 | ZNF471 |
| ENSBTAG00000053375 | 18 | 63812183 | 63811457 | 1 |  |
| ENSBTAG00000054544 | 18 | 63835510 | 63822637 | -1 | SMIM17 |
| ENSBTAG00000051066 | 18 | 63851730 | 63846562 | -1 | ZNF71 |
| ENSBTAG00000046101 | 18 | 63872963 | 63864852 | -1 | ZNF470 |
| ENSBTAG00000002055 | 18 | 63907053 | 63880404 | -1 | ZFP28 |
| ENSBTAG00000050453 | 18 | 63913508 | 63907938 | -1 |  |
| ENSBTAG00000048927 | 18 | 63951090 | 63950125 | 1 |  |
| ENSBTAG00000054360 | 18 | 63964139 | 63960651 | 1 | OR6Z9 |
| ENSBTAG00000051081 | 18 | 63969960 | 63969022 | -1 | OR6Z3 |
| ENSBTAG00000011664 | 18 | 64019858 | 64011118 | -1 | ZIM2 |
| ENSBTAG00000038326 | 18 | 64043302 | 64034413 | -1 |  |
| ENSBTAG00000048005 | 18 | 64061205 | 64048790 | -1 | MGC157368 |
| ENSBTAG00000054518 | 18 | 64076678 | 64066454 | -1 |  |
| ENSBTAG00000055216 | 18 | 64092010 | 64085063 | -1 |  |
| ENSBTAG00000054572 | 18 | 64118980 | 64099930 | -1 | PEG3 |
| ENSBTAG00000047251 | 18 | 64108823 | 64108770 | -1 | bta-mir-2900 |
| ENSBTAG00000050637 | 18 | 64123028 | 64122952 | 1 |  |
| ENSBTAG00000051123 | 18 | 64130114 | 64129995 | 1 |  |
| ENSBTAG00000044457 | 18 | 64313234 | 64313137 | -1 | U6 |
| ENSBTAG00000052336 | 18 | 64378543 | 64374169 | -1 | ZIM3 |
| ENSBTAG00000050946 | 18 | 64400322 | 64398336 | -1 |  |
| ENSBTAG00000048466 | 18 | 64435396 | 64424836 | 1 |  |
| ENSBTAG00000003261 | 18 | 64458059 | 64453980 | 1 | AURKC |
| ENSBTAG00000038926 | 18 | 64475319 | 64463752 | 1 | ZNF805 |
| ENSBTAG00000049557 | 18 | 64500782 | 64499682 | 1 |  |
| ENSBTAG00000037721 | 18 | 64540693 | 64505119 | -1 |  |
| ENSBTAG00000017872 | 18 | 64525539 | 64520900 | 1 | ZNF304 |
| ENSBTAG00000039341 | 18 | 64572891 | 64571839 | -1 |  |
| ENSBTAG00000037375 | 18 | 64595246 | 64587441 | -1 |  |
| ENSBTAG00000048682 | 18 | 64619591 | 64609623 | -1 | ZNF548 |
| ENSBTAG00000000195 | 18 | 64640989 | 64632169 | 1 |  |
| ENSBTAG00000054041 | 18 | 64653892 | 64651877 | -1 |  |
| ENSBTAG00000054074 | 18 | 64664583 | 64656232 | -1 |  |
| ENSBTAG00000053810 | 18 | 64724504 | 64660529 | -1 |  |
| ENSBTAG00000040358 | 18 | 64718827 | 64684152 | -1 |  |
| ENSBTAG00000017613 | 18 | 64705250 | 64698070 | 1 | ZNF419 |
| ENSBTAG00000051941 | 18 | 64741530 | 64734866 | -1 |  |
| ENSBTAG00000009460 | 18 | 64764864 | 64754096 | -1 | ZNF550 |
| ENSBTAG00000048481 | 18 | 64780421 | 64775206 | 1 |  |
| ENSBTAG00000054988 | 18 | 64782049 | 64780517 | 1 |  |
| ENSBTAG00000054538 | 18 | 64792383 | 64789351 | -1 |  |
| ENSBTAG00000037981 | 18 | 64808625 | 64803007 | -1 |  |
| ENSBTAG00000048261 | 18 | 64817427 | 64816465 | -1 |  |
| ENSBTAG00000051980 | 18 | 64842776 | 64842670 | 1 | U6 |
| ENSBTAG00000049688 | 18 | 64850447 | 64848219 | 1 |  |
| ENSBTAG00000055070 | 18 | 64870921 | 64856105 | 1 |  |
| ENSBTAG00000038240 | 18 | 64876602 | 64875301 | 1 |  |
| ENSBTAG00000050241 | 18 | 64880535 | 64880429 | 1 | U6 |
| ENSBTAG00000048765 | 18 | 64891245 | 64890300 | -1 |  |
| ENSBTAG00000047568 | 18 | 64905966 | 64903999 | 1 |  |
| ENSBTAG00000053290 | 18 | 64926031 | 64924829 | -1 |  |
| ENSBTAG00000049011 | 18 | 64927590 | 64927243 | 1 |  |
| ENSBTAG00000049069 | 18 | 64943595 | 64940798 | -1 |  |
| ENSBTAG00000038635 | 18 | 65182761 | 64948170 | -1 |  |
| ENSBTAG00000040169 | 18 | 65037998 | 65024314 | 1 |  |
| ENSBTAG00000015866 | 18 | 65050896 | 65047407 | -1 |  |
| ENSBTAG00000054731 | 18 | 65089344 | 65079731 | 1 |  |
| ENSBTAG00000038947 | 18 | 65103788 | 65102323 | 1 | ZSCAN4 |
| ENSBTAG00000055250 | 18 | 65121511 | 65112478 | 1 |  |
| ENSBTAG00000003447 | 18 | 65143289 | 65142027 | 1 |  |
| ENSBTAG00000053246 | 18 | 65172754 | 65170465 | -1 |  |
| ENSBTAG00000038088 | 18 | 65254845 | 65252212 | -1 |  |
| ENSBTAG00000038715 | 18 | 65328279 | 65314357 | 1 |  |
| ENSBTAG00000052402 | 18 | 65323594 | 65322290 | -1 |  |
| ENSBTAG00000049127 | 18 | 65347515 | 65344217 | 1 |  |
| ENSBTAG00000054370 | 18 | 65362907 | 65356584 | 1 |  |
| ENSBTAG00000040442 | 18 | 65367229 | 65365400 | 1 |  |
| ENSBTAG00000039946 | 18 | 65405941 | 65375097 | 1 | ZNF814 |
| ENSBTAG00000043658 | 18 | 65422949 | 65422830 | 1 | 5S_rRNA |
| ENSBTAG00000027787 | 18 | 65442602 | 65442182 | 1 |  |
| ENSBTAG00000003456 | 18 | 65468871 | 65466424 | -1 | ZNF606 |
| ENSBTAG00000043433 | 18 | 65477693 | 65477589 | 1 | U6 |
| ENSBTAG00000051756 | 18 | 65486952 | 65483993 | -1 |  |
| ENSBTAG00000003465 | 18 | 65528663 | 65485031 | -1 | ZNF329 |
| ENSBTAG00000003462 | 18 | 65508164 | 65499669 | 1 | ZNF135 |
| ENSBTAG00000013353 | 18 | 65568827 | 65546714 | 1 | ZNF274 |
| ENSBTAG00000044508 | 18 | 65553683 | 65553582 | 1 | U6 |
| ENSBTAG00000049556 | 18 | 65599864 | 65579823 | 1 |  |
| ENSBTAG00000052906 | 18 | 65617689 | 65600904 | 1 | ZNF8 |
| ENSBTAG00000009735 | 18 | 65646101 | 65641089 | -1 | A1BG |
| ENSBTAG00000015989 | 18 | 65665846 | 65660240 | 1 | RPS5 |
| ENSBTAG00000037882 | 18 | 65691647 | 65680940 | 1 | ZNF584 |
| ENSBTAG00000003267 | 18 | 65701693 | 65692531 | -1 | ZNF132 |
| ENSBTAG00000052922 | 18 | 65710756 | 65702278 | 1 |  |
| ENSBTAG00000039453 | 18 | 65718607 | 65712918 | 1 |  |
| ENSBTAG00000044536 | 18 | 65713930 | 65713783 | 1 |  |
| ENSBTAG00000038534 | 18 | 65722871 | 65720236 | 1 | ZNF446 |
| ENSBTAG00000052100 | 18 | 65729032 | 65724499 | 1 |  |
| ENSBTAG00000015164 | 18 | 65741718 | 65732865 | -1 | SLC27A5 |
| ENSBTAG00000051830 | 18 | 65745802 | 65743443 | -1 | ZBTB45 |
| ENSBTAG00000006422 | 18 | 65775447 | 65769086 | 1 | TRIM28 |
| ENSBTAG00000013882 | 18 | 65778680 | 65775983 | -1 | CHMP2A |
| ENSBTAG00000012744 | 18 | 65782460 | 65779277 | -1 | UBE2M |
| ENSBTAG00000037581 | 18 | 65796086 | 65786615 | -1 | MZF1 |
| ENSBTAG00000054839 | 18 | 65805308 | 65801135 | 1 |  |
| ENSBTAG00000049866 | 18 | 65813823 | 65811961 | 1 |  |
| ENSBTAG00000021328 | 2 | 1.16E+08 | 1.16E+08 | -1 | SPHKAP |
| ENSBTAG00000037640 | 2 | 1.17E+08 | 1.17E+08 | -1 | PID1 |
| ENSBTAG00000044908 | 2 | 1.17E+08 | 1.17E+08 | -1 | 7SK |
| ENSBTAG00000016063 | 2 | 1.18E+08 | 1.17E+08 | -1 | DNER |
| ENSBTAG00000049334 | 2 | 1.17E+08 | 1.17E+08 | 1 | U6 |
| ENSBTAG00000045470 | 2 | 1.17E+08 | 1.17E+08 | -1 | bta-mir-2284m |
| ENSBTAG00000049805 | 2 | 1.18E+08 | 1.18E+08 | -1 |  |
| ENSBTAG00000039046 | 2 | 1.18E+08 | 1.18E+08 | 1 | CD24 |
| ENSBTAG00000021653 | 2 | 1.18E+08 | 1.18E+08 | -1 | TRIP12 |
| ENSBTAG00000010338 | 2 | 1.18E+08 | 1.18E+08 | 1 | FBXO36 |
| ENSBTAG00000054843 | 2 | 1.18E+08 | 1.18E+08 | 1 | U6 |
| ENSBTAG00000053519 | 2 | 1.18E+08 | 1.18E+08 | -1 | U6 |
| ENSBTAG00000010344 | 2 | 1.18E+08 | 1.18E+08 | -1 | SLC16A14 |
| ENSBTAG00000007280 | 2 | 1.18E+08 | 1.18E+08 | 1 |  |
| ENSBTAG00000005584 | 2 | 1.18E+08 | 1.18E+08 | 1 |  |
| ENSBTAG00000015752 | 2 | 1.18E+08 | 1.18E+08 | -1 | SP110 |
| ENSBTAG00000009664 | 2 | 1.18E+08 | 1.18E+08 | 1 |  |
| ENSBTAG00000044419 | 2 | 1.18E+08 | 1.18E+08 | -1 | 5S_rRNA |
| ENSBTAG00000047133 | 2 | 1.18E+08 | 1.18E+08 | 1 |  |
| ENSBTAG00000054636 | 2 | 1.18E+08 | 1.18E+08 | 1 |  |
| ENSBTAG00000037702 | 2 | 1.18E+08 | 1.18E+08 | 1 | SP140L |
| ENSBTAG00000040055 | 2 | 1.19E+08 | 1.19E+08 | 1 | CAB39 |
| ENSBTAG00000017855 | 2 | 1.19E+08 | 1.19E+08 | 1 | ITM2C |
| ENSBTAG00000049476 | 2 | 1.19E+08 | 1.19E+08 | 1 | U6 |
| ENSBTAG00000017431 | 2 | 1.19E+08 | 1.19E+08 | -1 | GPR55 |
| ENSBTAG00000053516 | 2 | 1.19E+08 | 1.19E+08 | -1 |  |
| ENSBTAG00000030718 | 2 | 1.19E+08 | 1.19E+08 | 1 | SPATA3 |
| ENSBTAG00000054626 | 2 | 1.19E+08 | 1.19E+08 | 1 |  |
| ENSBTAG00000026710 | 2 | 1.19E+08 | 1.19E+08 | 1 | C2H2orf72 |
| ENSBTAG00000005119 | 2 | 1.19E+08 | 1.19E+08 | 1 | PSMD1 |
| ENSBTAG00000039772 | 2 | 1.19E+08 | 1.19E+08 | -1 | HTR2B |
| ENSBTAG00000018502 | 2 | 1.19E+08 | 1.19E+08 | 1 | ARMC9 |
| ENSBTAG00000053975 | 2 | 1.19E+08 | 1.19E+08 | 1 | U6 |
| ENSBTAG00000001359 | 2 | 1.19E+08 | 1.19E+08 | 1 | B3GNT7 |
| ENSBTAG00000050689 | 2 | 1.19E+08 | 1.19E+08 | 1 |  |
| ENSBTAG00000016174 | 2 | 1.19E+08 | 1.19E+08 | -1 | NCL |
| ENSBTAG00000042435 | 2 | 1.19E+08 | 1.19E+08 | -1 | SNORA75 |
| ENSBTAG00000043297 | 2 | 1.19E+08 | 1.19E+08 | -1 | SNORD20 |
| ENSBTAG00000017684 | 2 | 1.19E+08 | 1.19E+08 | -1 | NMUR1 |
| ENSBTAG00000054422 | 2 | 1.19E+08 | 1.19E+08 | -1 |  |
| ENSBTAG00000050503 | 2 | 1.19E+08 | 1.19E+08 | 1 |  |
| ENSBTAG00000051671 | 2 | 1.19E+08 | 1.19E+08 | 1 |  |
| ENSBTAG00000002549 | 2 | 1.19E+08 | 1.19E+08 | 1 | PTMA |
| ENSBTAG00000019480 | 2 | 1.19E+08 | 1.19E+08 | -1 | PDE6D |
| ENSBTAG00000014988 | 2 | 1.19E+08 | 1.19E+08 | 1 |  |
| ENSBTAG00000005207 | 2 | 1.2E+08 | 1.2E+08 | 1 | COPS7B |
| ENSBTAG00000003253 | 2 | 1.2E+08 | 1.2E+08 | -1 | NPPC |
| ENSBTAG00000011964 | 2 | 1.2E+08 | 1.2E+08 | 1 | DIS3L2 |
| ENSBTAG00000043514 | 2 | 1.2E+08 | 1.2E+08 | 1 | SNORA62 |
| ENSBTAG00000053448 | 2 | 1.2E+08 | 1.2E+08 | 1 |  |
| ENSBTAG00000016748 | 2 | 1.2E+08 | 1.2E+08 | 1 |  |
| ENSBTAG00000051665 | 2 | 1.2E+08 | 1.2E+08 | 1 |  |
| ENSBTAG00000039346 | 2 | 1.2E+08 | 1.2E+08 | 1 |  |
| ENSBTAG00000004519 | 2 | 1.2E+08 | 1.2E+08 | 1 | ALPI |
| ENSBTAG00000012363 | 2 | 1.2E+08 | 1.2E+08 | 1 |  |
| ENSBTAG00000021050 | 2 | 1.2E+08 | 1.2E+08 | -1 | ECEL1 |
| ENSBTAG00000050396 | 2 | 1.2E+08 | 1.2E+08 | 1 |  |
| ENSBTAG00000048397 | 2 | 1.2E+08 | 1.2E+08 | 1 | PRSS56 |
| ENSBTAG00000011390 | 2 | 1.2E+08 | 1.2E+08 | 1 | CHRND |
| ENSBTAG00000045943 | 2 | 1.2E+08 | 1.2E+08 | 1 | CHRNG |
| ENSBTAG00000010598 | 2 | 1.2E+08 | 1.2E+08 | 1 | EIF4E2 |
| ENSBTAG00000004603 | 2 | 1.2E+08 | 1.2E+08 | 1 | PHC2 |
| ENSBTAG00000054666 | 2 | 1.2E+08 | 1.2E+08 | 1 |  |
| ENSBTAG00000012629 | 2 | 1.2E+08 | 1.2E+08 | -1 | ZNF362 |
| ENSBTAG00000020671 | 2 | 1.21E+08 | 1.21E+08 | 1 | TRIM62 |
| ENSBTAG00000050972 | 2 | 1.21E+08 | 1.21E+08 | 1 |  |
| ENSBTAG00000020548 | 2 | 1.21E+08 | 1.21E+08 | -1 | AZIN2 |
| ENSBTAG00000017605 | 2 | 1.21E+08 | 1.21E+08 | 1 | AK2 |
| ENSBTAG00000044284 | 2 | 1.21E+08 | 1.21E+08 | -1 | bta-mir-2357 |
| ENSBTAG00000008330 | 2 | 1.21E+08 | 1.21E+08 | 1 | RNF19B |
| ENSBTAG00000008331 | 2 | 1.21E+08 | 1.21E+08 | 1 | TMEM54 |
| ENSBTAG00000008336 | 2 | 1.21E+08 | 1.21E+08 | -1 | HPCA |
| ENSBTAG00000051540 | 2 | 1.21E+08 | 1.21E+08 | 1 | FNDC5 |
| ENSBTAG00000018067 | 2 | 1.21E+08 | 1.21E+08 | -1 | S100PBP |
| ENSBTAG00000018065 | 2 | 1.21E+08 | 1.21E+08 | 1 | YARS1 |
| ENSBTAG00000018062 | 2 | 1.21E+08 | 1.21E+08 | -1 | KIAA1522 |
| ENSBTAG00000026684 | 2 | 1.21E+08 | 1.21E+08 | 1 | SYNC |
| ENSBTAG00000005904 | 2 | 1.21E+08 | 1.21E+08 | -1 | RBBP4 |
| ENSBTAG00000027159 | 2 | 1.21E+08 | 1.21E+08 | 1 | ZBTB8OS |
| ENSBTAG00000018199 | 2 | 1.21E+08 | 1.21E+08 | -1 | ZBTB8A |
| ENSBTAG00000046044 | 2 | 1.21E+08 | 1.21E+08 | -1 | ZBTB8B |
| ENSBTAG00000020655 | 2 | 1.21E+08 | 1.21E+08 | 1 | BSDC1 |
| ENSBTAG00000020652 | 2 | 1.21E+08 | 1.21E+08 | -1 | TSSK3 |
| ENSBTAG00000037902 | 2 | 1.21E+08 | 1.21E+08 | 1 | FAM229A |
| ENSBTAG00000046862 | 2 | 1.21E+08 | 1.21E+08 | 1 | MARCKSL1 |
| ENSBTAG00000012698 | 2 | 1.21E+08 | 1.21E+08 | -1 | HDAC1 |
| ENSBTAG00000012695 | 2 | 1.21E+08 | 1.21E+08 | -1 | LCK |
| ENSBTAG00000002027 | 2 | 1.21E+08 | 1.21E+08 | -1 | FAM167B |
| ENSBTAG00000014390 | 2 | 1.21E+08 | 1.21E+08 | 1 | MTMR9 |
| ENSBTAG00000014388 | 2 | 1.21E+08 | 1.21E+08 | -1 | EIF3I |
| ENSBTAG00000009163 | 2 | 1.21E+08 | 1.21E+08 | 1 | TMEM234 |
| ENSBTAG00000026696 | 2 | 1.21E+08 | 1.21E+08 | -1 | DCDC2B |
| ENSBTAG00000015103 | 2 | 1.21E+08 | 1.21E+08 | -1 | IQCC |
| ENSBTAG00000015104 | 2 | 1.21E+08 | 1.21E+08 | -1 | CCDC28B |
| ENSBTAG00000048869 | 2 | 1.21E+08 | 1.21E+08 | -1 |  |
| ENSBTAG00000046605 | 2 | 1.21E+08 | 1.21E+08 | -1 | U6 |
| ENSBTAG00000019486 | 2 | 1.21E+08 | 1.21E+08 | 1 | CLDN12 |
| ENSBTAG00000026788 | 2 | 1.21E+08 | 1.21E+08 | 1 | SHOX |
| ENSBTAG00000051364 | 2 | 1.22E+08 | 1.22E+08 | -1 |  |
| ENSBTAG00000054661 | 2 | 1.22E+08 | 1.22E+08 | -1 | bta-mir-2887-1 |
| ENSBTAG00000040518 | 2 | 1.22E+08 | 1.22E+08 | 1 |  |
| ENSBTAG00000039121 | 2 | 1.22E+08 | 1.22E+08 | 1 | PTP4A2 |
| ENSBTAG00000015715 | 2 | 1.22E+08 | 1.22E+08 | 1 | SPOCD1 |
| ENSBTAG00000010657 | 2 | 1.22E+08 | 1.22E+08 | 1 | ADGRB2 |
| ENSBTAG00000010050 | 2 | 1.22E+08 | 1.22E+08 | 1 | COL16A1 |
| ENSBTAG00000010378 | 2 | 1.22E+08 | 1.22E+08 | 1 | PEF1 |
| ENSBTAG00000045347 | 2 | 1.22E+08 | 1.22E+08 | 1 | SNORA70 |
| ENSBTAG00000010366 | 2 | 1.22E+08 | 1.22E+08 | -1 | HCRTR1 |
| ENSBTAG00000013235 | 2 | 1.22E+08 | 1.22E+08 | -1 | TINAGL1 |
| ENSBTAG00000039994 | 2 | 1.22E+08 | 1.22E+08 | -1 |  |
| ENSBTAG00000035081 | 2 | 1.22E+08 | 1.22E+08 | -1 | SERINC2 |
| ENSBTAG00000052286 | 2 | 1.22E+08 | 1.22E+08 | 1 |  |
| ENSBTAG00000016819 | 2 | 1.22E+08 | 1.22E+08 | 1 | FABP3 |
| ENSBTAG00000016818 | 2 | 1.22E+08 | 1.22E+08 | -1 | ZCCHC17 |
| ENSBTAG00000016817 | 2 | 1.22E+08 | 1.22E+08 | 1 | SNRNP40 |
| ENSBTAG00000020159 | 2 | 1.22E+08 | 1.22E+08 | 1 | NKAIN1 |
| ENSBTAG00000053976 | 2 | 1.22E+08 | 1.22E+08 | -1 |  |
| ENSBTAG00000018979 | 2 | 1.23E+08 | 1.23E+08 | 1 | PUM1 |
| ENSBTAG00000043864 | 2 | 1.23E+08 | 1.23E+08 | 1 |  |
| ENSBTAG00000042860 | 2 | 1.23E+08 | 1.23E+08 | 1 |  |
| ENSBTAG00000042142 | 2 | 1.23E+08 | 1.23E+08 | 1 |  |
| ENSBTAG00000050624 | 2 | 1.23E+08 | 1.23E+08 | 1 |  |
| ENSBTAG00000026676 | 2 | 1.23E+08 | 1.23E+08 | 1 | SDC3 |
| ENSBTAG00000005477 | 2 | 1.23E+08 | 1.23E+08 | 1 | LAPTM5 |
| ENSBTAG00000003479 | 2 | 1.23E+08 | 1.23E+08 | 1 | MATN1 |
| ENSBTAG00000053024 | 2 | 1.24E+08 | 1.24E+08 | 1 |  |
| ENSBTAG00000012848 | 2 | 1.24E+08 | 1.24E+08 | -1 | PTPRU |
| ENSBTAG00000017253 | 2 | 1.24E+08 | 1.24E+08 | 1 | MECR |
| ENSBTAG00000048793 | 2 | 1.24E+08 | 1.24E+08 | 1 | U6 |
| ENSBTAG00000013309 | 2 | 1.24E+08 | 1.24E+08 | 1 | SRSF4 |
| ENSBTAG00000051464 | 2 | 1.24E+08 | 1.24E+08 | 1 |  |
| ENSBTAG00000047524 | 2 | 1.24E+08 | 1.24E+08 | 1 | TMEM200B |
| ENSBTAG00000006667 | 2 | 1.25E+08 | 1.24E+08 | -1 | EPB41 |
| ENSBTAG00000003202 | 2 | 1.25E+08 | 1.25E+08 | -1 | OPRD1 |
| ENSBTAG00000015771 | 2 | 1.25E+08 | 1.25E+08 | -1 | YTHDF2 |
| ENSBTAG00000043380 | 2 | 1.25E+08 | 1.25E+08 | 1 | U6 |
| ENSBTAG00000001160 | 2 | 1.25E+08 | 1.25E+08 | -1 | GMEB1 |
| ENSBTAG00000042723 | 2 | 1.25E+08 | 1.25E+08 | -1 |  |
| ENSBTAG00000026660 | 2 | 1.25E+08 | 1.25E+08 | 1 | TAF12 |
| ENSBTAG00000021525 | 2 | 1.25E+08 | 1.25E+08 | -1 | RAB42 |
| ENSBTAG00000052614 | 2 | 1.25E+08 | 1.25E+08 | 1 |  |
| ENSBTAG00000048228 | 2 | 1.25E+08 | 1.25E+08 | 1 |  |
| ENSBTAG00000042986 | 2 | 1.25E+08 | 1.25E+08 | 1 |  |
| ENSBTAG00000042871 | 2 | 1.25E+08 | 1.25E+08 | 1 | SNORA44 |
| ENSBTAG00000043315 | 2 | 1.25E+08 | 1.25E+08 | 1 | SNORA61 |
| ENSBTAG00000042553 | 2 | 1.25E+08 | 1.25E+08 | 1 | SNORD99 |
| ENSBTAG00000010393 | 2 | 1.25E+08 | 1.25E+08 | -1 | TRNAU1AP |
| ENSBTAG00000003788 | 2 | 1.25E+08 | 1.25E+08 | -1 | RCC1 |
| ENSBTAG00000055165 | 2 | 1.25E+08 | 1.25E+08 | -1 | SNORA73 |
| ENSBTAG00000050344 | 2 | 1.25E+08 | 1.25E+08 | -1 | SNORA73 |
| ENSBTAG00000051053 | 2 | 1.25E+08 | 1.25E+08 | -1 | SNORA73 |
| ENSBTAG00000002727 | 2 | 1.25E+08 | 1.25E+08 | -1 | PHACTR4 |
| ENSBTAG00000003676 | 2 | 1.25E+08 | 1.25E+08 | 1 |  |
| ENSBTAG00000040210 | 2 | 1.25E+08 | 1.25E+08 | 1 |  |
| ENSBTAG00000045032 | 2 | 1.25E+08 | 1.25E+08 | 1 |  |
| ENSBTAG00000002678 | 2 | 1.25E+08 | 1.25E+08 | -1 | MED18 |
| ENSBTAG00000002363 | 2 | 1.25E+08 | 1.25E+08 | -1 | SESN2 |
| ENSBTAG00000006342 | 2 | 1.25E+08 | 1.25E+08 | -1 | ATP5IF1 |
| ENSBTAG00000018385 | 2 | 1.25E+08 | 1.25E+08 | 1 | DNAJC8 |
| ENSBTAG00000054543 | 2 | 1.25E+08 | 1.25E+08 | -1 |  |
| ENSBTAG00000027051 | 2 | 1.25E+08 | 1.25E+08 | 1 | PTAFR |
| ENSBTAG00000043989 | 2 | 1.25E+08 | 1.25E+08 | 1 | EYA3 |
| ENSBTAG00000008800 | 2 | 1.25E+08 | 1.25E+08 | -1 | XKR8 |
| ENSBTAG00000012997 | 2 | 1.25E+08 | 1.25E+08 | -1 | SMPDL3B |
| ENSBTAG00000006225 | 2 | 1.25E+08 | 1.25E+08 | 1 | RPA2 |
| ENSBTAG00000006223 | 2 | 1.25E+08 | 1.25E+08 | -1 | THEMIS2 |
| ENSBTAG00000003866 | 2 | 1.26E+08 | 1.26E+08 | -1 | PPP1R8 |
| ENSBTAG00000044441 | 2 | 1.26E+08 | 1.26E+08 | -1 | SCARNA1 |
| ENSBTAG00000001192 | 2 | 1.26E+08 | 1.26E+08 | -1 | STX12 |
| ENSBTAG00000019614 | 2 | 1.26E+08 | 1.26E+08 | -1 | FAM76A |
| ENSBTAG00000007554 | 2 | 1.26E+08 | 1.26E+08 | 1 | IFI6 |
| ENSBTAG00000011784 | 2 | 1.26E+08 | 1.26E+08 | 1 | FGR |
| ENSBTAG00000037456 | 2 | 1.26E+08 | 1.26E+08 | 1 | AHDC1 |
| ENSBTAG00000018374 | 2 | 1.26E+08 | 1.26E+08 | 1 | WASF2 |
| ENSBTAG00000052426 | 2 | 1.26E+08 | 1.26E+08 | -1 | U6 |
| ENSBTAG00000002505 | 2 | 1.26E+08 | 1.26E+08 | -1 | GPR3 |
| ENSBTAG00000019728 | 2 | 1.26E+08 | 1.26E+08 | 1 | CD164L2 |
| ENSBTAG00000010254 | 2 | 1.26E+08 | 1.26E+08 | 1 | MAP3K6 |
| ENSBTAG00000010253 | 2 | 1.26E+08 | 1.26E+08 | -1 | SYTL1 |
| ENSBTAG00000010249 | 2 | 1.26E+08 | 1.26E+08 | -1 | TMEM222 |
| ENSBTAG00000050795 | 2 | 1.26E+08 | 1.26E+08 | -1 |  |
| ENSBTAG00000053588 | 2 | 1.26E+08 | 1.26E+08 | -1 | WDTC1 |
| ENSBTAG00000008766 | 2 | 1.26E+08 | 1.26E+08 | 1 | SLC9A1 |
| ENSBTAG00000051649 | 2 | 1.26E+08 | 1.26E+08 | 1 |  |
| ENSBTAG00000051814 | 2 | 1.26E+08 | 1.26E+08 | 1 |  |
| ENSBTAG00000018413 | 2 | 1.26E+08 | 1.26E+08 | 1 | TENT5B |
| ENSBTAG00000054719 | 2 | 1.26E+08 | 1.26E+08 | -1 | TRNP1 |
| ENSBTAG00000019252 | 2 | 1.26E+08 | 1.26E+08 | 1 | KDF1 |
| ENSBTAG00000055176 | 2 | 1.26E+08 | 1.26E+08 | -1 |  |
| ENSBTAG00000004416 | 2 | 1.26E+08 | 1.26E+08 | -1 | NUDC |
| ENSBTAG00000014848 | 2 | 1.26E+08 | 1.26E+08 | 1 | NR0B2 |
| ENSBTAG00000014849 | 2 | 1.26E+08 | 1.26E+08 | 1 | GPATCH3 |
| ENSBTAG00000014850 | 2 | 1.26E+08 | 1.26E+08 | 1 | GPN2 |
| ENSBTAG00000009223 | 2 | 1.26E+08 | 1.26E+08 | -1 | SFN |
| ENSBTAG00000046672 | 2 | 1.26E+08 | 1.26E+08 | -1 | ZDHHC18 |
| ENSBTAG00000009796 | 2 | 1.26E+08 | 1.26E+08 | -1 | PIGV |
| ENSBTAG00000001024 | 2 | 1.26E+08 | 1.26E+08 | -1 | ARID1A |
| ENSBTAG00000014447 | 2 | 1.27E+08 | 1.27E+08 | -1 | RPS6KA1 |
| ENSBTAG00000003235 | 2 | 1.27E+08 | 1.27E+08 | -1 | DHDDS |
| ENSBTAG00000049812 | 2 | 1.27E+08 | 1.27E+08 | 1 |  |
| ENSBTAG00000040497 | 2 | 1.27E+08 | 1.27E+08 | -1 | LIN28A |
| ENSBTAG00000009631 | 2 | 1.27E+08 | 1.27E+08 | 1 | ZNF683 |
| ENSBTAG00000005629 | 2 | 1.27E+08 | 1.27E+08 | 1 | CRYBG2 |
| ENSBTAG00000005628 | 2 | 1.27E+08 | 1.27E+08 | -1 | CD52 |
| ENSBTAG00000009581 | 2 | 1.27E+08 | 1.27E+08 | 1 | UBXN11 |
| ENSBTAG00000009580 | 2 | 1.27E+08 | 1.27E+08 | -1 | SH3BGRL3 |
| ENSBTAG00000009579 | 2 | 1.27E+08 | 1.27E+08 | -1 | CEP85 |
| ENSBTAG00000026638 | 2 | 1.27E+08 | 1.27E+08 | -1 | CATSPER4 |
| ENSBTAG00000019399 | 2 | 1.27E+08 | 1.27E+08 | -1 | CNKSR1 |
| ENSBTAG00000050680 | 2 | 1.27E+08 | 1.27E+08 | 1 | ZNF593OS |
| ENSBTAG00000009562 | 2 | 1.27E+08 | 1.27E+08 | -1 | ZNF593 |
| ENSBTAG00000013131 | 2 | 1.27E+08 | 1.27E+08 | -1 | FAM110D |
| ENSBTAG00000001513 | 2 | 1.27E+08 | 1.27E+08 | -1 | PDIK1L |
| ENSBTAG00000005085 | 2 | 1.27E+08 | 1.27E+08 | 1 | TRIM63 |
| ENSBTAG00000006355 | 2 | 1.27E+08 | 1.27E+08 | 1 | SLC30A2 |
| ENSBTAG00000006349 | 2 | 1.27E+08 | 1.27E+08 | -1 | EXTL1 |
| ENSBTAG00000005105 | 2 | 1.27E+08 | 1.27E+08 | 1 | PAFAH2 |
| ENSBTAG00000013761 | 2 | 1.27E+08 | 1.27E+08 | 1 | STMN1 |
| ENSBTAG00000021787 | 2 | 1.27E+08 | 1.27E+08 | 1 | PAQR7 |
| ENSBTAG00000021786 | 2 | 1.27E+08 | 1.27E+08 | 1 | AUNIP |
| ENSBTAG00000021781 | 2 | 1.27E+08 | 1.27E+08 | -1 | MTFR1L |
| ENSBTAG00000021778 | 2 | 1.27E+08 | 1.27E+08 | -1 | SELENON |
| ENSBTAG00000052666 | 2 | 1.27E+08 | 1.27E+08 | -1 |  |
| ENSBTAG00000003069 | 2 | 1.27E+08 | 1.27E+08 | -1 | MAN1C1 |
| ENSBTAG00000001050 | 2 | 1.28E+08 | 1.27E+08 | -1 | LDLRAP1 |
| ENSBTAG00000002988 | 2 | 1.28E+08 | 1.28E+08 | -1 | MACO1 |
| ENSBTAG00000001297 | 2 | 1.28E+08 | 1.28E+08 | 1 |  |
| ENSBTAG00000052592 | 2 | 1.28E+08 | 1.28E+08 | -1 | U6 |
| ENSBTAG00000001296 | 2 | 1.28E+08 | 1.28E+08 | -1 | TMEM50A |
| ENSBTAG00000015222 | 2 | 1.28E+08 | 1.28E+08 | 1 | RSRP1 |
| ENSBTAG00000001651 | 2 | 1.28E+08 | 1.28E+08 | 1 | SYF2 |
| ENSBTAG00000050474 | 2 | 1.28E+08 | 1.28E+08 | 1 | RUNX3 |
| ENSBTAG00000009470 | 2 | 1.28E+08 | 1.28E+08 | -1 | CLIC4 |
| ENSBTAG00000054129 | 2 | 1.28E+08 | 1.28E+08 | -1 |  |
| ENSBTAG00000054645 | 2 | 1.28E+08 | 1.28E+08 | -1 |  |
| ENSBTAG00000013772 | 2 | 1.28E+08 | 1.28E+08 | -1 | SRRM1 |
| ENSBTAG00000012604 | 2 | 1.28E+08 | 1.28E+08 | -1 | NCMAP |
| ENSBTAG00000017452 | 2 | 1.28E+08 | 1.28E+08 | -1 | RCAN3 |
| ENSBTAG00000013048 | 2 | 1.28E+08 | 1.28E+08 | -1 | NIPAL3 |
| ENSBTAG00000010278 | 2 | 1.29E+08 | 1.29E+08 | 1 | STPG1 |
| ENSBTAG00000019791 | 2 | 1.29E+08 | 1.29E+08 | -1 | GRHL3 |
| ENSBTAG00000045407 | 2 | 1.29E+08 | 1.29E+08 | 1 | U6 |
| ENSBTAG00000001101 | 2 | 1.29E+08 | 1.29E+08 | 1 | IFNLR1 |
| ENSBTAG00000001100 | 2 | 1.29E+08 | 1.29E+08 | 1 | IL22RA1 |
| ENSBTAG00000014885 | 2 | 1.29E+08 | 1.29E+08 | 1 | MYOM3 |
| ENSBTAG00000042918 | 2 | 1.29E+08 | 1.29E+08 | -1 | U6 |
| ENSBTAG00000008072 | 2 | 1.29E+08 | 1.29E+08 | 1 | SRSF10 |
| ENSBTAG00000030435 | 2 | 1.29E+08 | 1.29E+08 | -1 | PNRC2 |
| ENSBTAG00000019371 | 2 | 1.29E+08 | 1.29E+08 | 1 | CNR2 |
| ENSBTAG00000030434 | 2 | 1.29E+08 | 1.29E+08 | 1 | FUCA1 |
| ENSBTAG00000053569 | 2 | 1.29E+08 | 1.29E+08 | 1 | U6 |
| ENSBTAG00000021832 | 2 | 1.29E+08 | 1.29E+08 | 1 | HMGCL |
| ENSBTAG00000005002 | 2 | 1.29E+08 | 1.29E+08 | 1 | GALE |
| ENSBTAG00000011625 | 2 | 1.29E+08 | 1.29E+08 | -1 | LYPLA2 |
| ENSBTAG00000021620 | 2 | 1.29E+08 | 1.29E+08 | -1 | PITHD1 |
| ENSBTAG00000049982 | 2 | 1.29E+08 | 1.29E+08 | -1 | bta-mir-10181 |
| ENSBTAG00000026585 | 2 | 1.29E+08 | 1.29E+08 | -1 | ELOA |
| ENSBTAG00000020905 | 2 | 1.29E+08 | 1.29E+08 | -1 | RPL11 |
| ENSBTAG00000052293 | 2 | 1.29E+08 | 1.29E+08 | 1 |  |
| ENSBTAG00000030425 | 2 | 1.29E+08 | 1.29E+08 | 1 | ID3 |
| ENSBTAG00000014400 | 2 | 1.29E+08 | 1.29E+08 | 1 | E2F2 |
| ENSBTAG00000012263 | 2 | 1.29E+08 | 1.29E+08 | 1 | ASAP3 |
| ENSBTAG00000048702 | 2 | 1.29E+08 | 1.29E+08 | -1 | U6 |
| ENSBTAG00000038865 | 2 | 1.29E+08 | 1.29E+08 | 1 | TCEA3 |
| ENSBTAG00000002594 | 2 | 1.29E+08 | 1.29E+08 | 1 | ZNF436 |
| ENSBTAG00000016578 | 2 | 1.3E+08 | 1.3E+08 | 1 | HNRNPR |
| ENSBTAG00000040329 | 2 | 1.3E+08 | 1.3E+08 | 1 | HTR1D |
| ENSBTAG00000009502 | 2 | 1.3E+08 | 1.3E+08 | 1 | LUZP1 |
| ENSBTAG00000009500 | 2 | 1.3E+08 | 1.3E+08 | -1 | KDM1A |
| ENSBTAG00000053631 | 2 | 1.3E+08 | 1.3E+08 | 1 | TEX46 |
| ENSBTAG00000030413 | 2 | 1.3E+08 | 1.3E+08 | 1 | LACTBL1 |
| ENSBTAG00000045902 | 2 | 1.3E+08 | 1.3E+08 | -1 | EPHB2 |
| ENSBTAG00000011196 | 2 | 1.3E+08 | 1.3E+08 | -1 | C1QB |
| ENSBTAG00000011195 | 2 | 1.3E+08 | 1.3E+08 | -1 |  |
| ENSBTAG00000011193 | 2 | 1.3E+08 | 1.3E+08 | -1 | C1QC |
| ENSBTAG00000007153 | 2 | 1.3E+08 | 1.3E+08 | -1 | C1QA |
| ENSBTAG00000020102 | 2 | 1.3E+08 | 1.3E+08 | -1 | EPHA8 |
| ENSBTAG00000016448 | 2 | 1.3E+08 | 1.3E+08 | -1 | ZBTB40 |
| ENSBTAG00000045330 | 2 | 1.3E+08 | 1.3E+08 | 1 | bta-mir-2284u |
| ENSBTAG00000051083 | 2 | 1.31E+08 | 1.31E+08 | 1 | WNT4 |
| ENSBTAG00000048494 | 2 | 1.31E+08 | 1.31E+08 | -1 | U6 |
| ENSBTAG00000001700 | 2 | 1.31E+08 | 1.31E+08 | -1 | CDC42 |
| ENSBTAG00000040602 | 2 | 1.31E+08 | 1.31E+08 | -1 |  |
| ENSBTAG00000013085 | 2 | 1.31E+08 | 1.31E+08 | -1 | CELA3B |
| ENSBTAG00000030269 | 2 | 1.31E+08 | 1.31E+08 | 1 |  |
| ENSBTAG00000017122 | 2 | 1.31E+08 | 1.31E+08 | 1 | HSPG2 |
| ENSBTAG00000006940 | 2 | 1.31E+08 | 1.31E+08 | 1 | USP48 |
| ENSBTAG00000008952 | 2 | 1.31E+08 | 1.31E+08 | 1 | RAP1GAP |
| ENSBTAG00000008951 | 2 | 1.31E+08 | 1.31E+08 | -1 | ALPL |
| ENSBTAG00000042825 | 2 | 1.31E+08 | 1.31E+08 | 1 | U6 |
| ENSBTAG00000002977 | 2 | 1.31E+08 | 1.31E+08 | 1 | ECE1 |
| ENSBTAG00000040215 | 2 | 1.32E+08 | 1.31E+08 | 1 | EIF4G3 |
| ENSBTAG00000018656 | 2 | 1.32E+08 | 1.32E+08 | 1 | HP1BP3 |
| ENSBTAG00000001082 | 2 | 1.32E+08 | 1.32E+08 | 1 | SH2D5 |
| ENSBTAG00000050449 | 2 | 1.32E+08 | 1.32E+08 | 1 |  |
| ENSBTAG00000017644 | 2 | 1.32E+08 | 1.32E+08 | 1 | KIF17 |
| ENSBTAG00000006085 | 2 | 1.32E+08 | 1.32E+08 | 1 | DDOST |
| ENSBTAG00000006084 | 2 | 1.32E+08 | 1.32E+08 | -1 | PINK1 |
| ENSBTAG00000052516 | 2 | 1.32E+08 | 1.32E+08 | -1 | CDA |
| ENSBTAG00000046988 | 2 | 1.32E+08 | 1.32E+08 | -1 | FAM43B |
| ENSBTAG00000049959 | 2 | 1.32E+08 | 1.32E+08 | 1 |  |
| ENSBTAG00000055116 | 2 | 1.32E+08 | 1.32E+08 | -1 |  |
| ENSBTAG00000020850 | 2 | 1.32E+08 | 1.32E+08 | 1 | MUL1 |
| ENSBTAG00000009156 | 2 | 1.32E+08 | 1.32E+08 | 1 | CAMK2N1 |
| ENSBTAG00000050271 | 2 | 1.32E+08 | 1.32E+08 | 1 |  |
| ENSBTAG00000013511 | 2 | 1.32E+08 | 1.32E+08 | -1 | VWA5B1 |
| ENSBTAG00000040547 | 2 | 1.32E+08 | 1.32E+08 | -1 | UBXN10 |
| ENSBTAG00000016877 | 2 | 1.32E+08 | 1.32E+08 | 1 | PLA2G2C |
| ENSBTAG00000050633 | 2 | 1.33E+08 | 1.32E+08 | -1 | PLA2G2F |
| ENSBTAG00000046977 | 2 | 1.33E+08 | 1.33E+08 | 1 | PLA2G2D4 |
| ENSBTAG00000020499 | 2 | 1.33E+08 | 1.33E+08 | 1 |  |
| ENSBTAG00000050719 | 2 | 1.33E+08 | 1.33E+08 | 1 | PLA2G2D1 |
| ENSBTAG00000050719 | 2 | 1.33E+08 | 1.33E+08 | 1 | PLA2G2D1 |
| ENSBTAG00000039122 | 2 | 1.33E+08 | 1.33E+08 | -1 | PLA2G5 |
| ENSBTAG00000002700 | 2 | 1.33E+08 | 1.33E+08 | 1 | PLA2G2A |
| ENSBTAG00000048919 | 2 | 1.33E+08 | 1.33E+08 | 1 |  |
| ENSBTAG00000005759 | 2 | 1.33E+08 | 1.33E+08 | 1 | PLA2G2A |
| ENSBTAG00000013039 | 2 | 1.33E+08 | 1.33E+08 | 1 |  |
| ENSBTAG00000004418 | 2 | 1.33E+08 | 1.33E+08 | 1 | PLA2G2E |
| ENSBTAG00000017108 | 2 | 1.33E+08 | 1.33E+08 | -1 | OTUD3 |
| ENSBTAG00000030367 | 2 | 1.33E+08 | 1.33E+08 | 1 | RNF186 |
| ENSBTAG00000007998 | 2 | 1.33E+08 | 1.33E+08 | 1 | TMCO4 |
| ENSBTAG00000007996 | 2 | 1.33E+08 | 1.33E+08 | -1 | HTR6 |
| ENSBTAG00000005140 | 2 | 1.33E+08 | 1.33E+08 | -1 | NBL1 |
| ENSBTAG00000050219 | 2 | 1.33E+08 | 1.33E+08 | -1 | MICOS10 |
| ENSBTAG00000044904 | 2 | 1.33E+08 | 1.33E+08 | -1 | U6 |
| ENSBTAG00000004554 | 2 | 1.33E+08 | 1.33E+08 | 1 | CAPZB |
| ENSBTAG00000013650 | 2 | 1.33E+08 | 1.33E+08 | -1 | SLC66A1 |
| ENSBTAG00000012289 | 2 | 1.33E+08 | 1.33E+08 | 1 | AKR7A2 |
| ENSBTAG00000026758 | 2 | 1.33E+08 | 1.33E+08 | -1 |  |
| ENSBTAG00000011951 | 2 | 1.33E+08 | 1.33E+08 | -1 | MRTO4 |
| ENSBTAG00000011950 | 2 | 1.33E+08 | 1.33E+08 | 1 | EMC1 |
| ENSBTAG00000052114 | 2 | 1.33E+08 | 1.33E+08 | 1 |  |
| ENSBTAG00000044375 | 2 | 1.33E+08 | 1.33E+08 | -1 | SNORA70 |
| ENSBTAG00000015240 | 2 | 1.34E+08 | 1.33E+08 | 1 | UBR4 |
| ENSBTAG00000028707 | 2 | 1.34E+08 | 1.34E+08 | 1 | U3 |
| ENSBTAG00000051731 | 2 | 1.34E+08 | 1.34E+08 | -1 | U1 |
| ENSBTAG00000030340 | 2 | 1.34E+08 | 1.34E+08 | 1 | IFFO2 |
| ENSBTAG00000030335 | 2 | 1.34E+08 | 1.34E+08 | 1 | ALDH4A1 |
| ENSBTAG00000015246 | 2 | 1.34E+08 | 1.34E+08 | 1 | TAS1R2 |
| ENSBTAG00000014967 | 2 | 1.34E+08 | 1.34E+08 | -1 | PAX7 |
| ENSBTAG00000030322 | 2 | 1.34E+08 | 1.34E+08 | -1 | KLHDC7A |
| ENSBTAG00000054138 | 2 | 1.34E+08 | 1.34E+08 | 1 |  |
| ENSBTAG00000053388 | 2 | 1.34E+08 | 1.34E+08 | -1 |  |
| ENSBTAG00000019172 | 2 | 1.34E+08 | 1.34E+08 | -1 | IGSF21 |
| ENSBTAG00000006438 | 2 | 1.35E+08 | 1.35E+08 | -1 | ACTL8 |
| ENSBTAG00000000684 | 2 | 1.35E+08 | 1.35E+08 | -1 | ARHGEF10L |
| ENSBTAG00000048830 | 2 | 1.35E+08 | 1.35E+08 | -1 |  |
| ENSBTAG00000008579 | 2 | 1.35E+08 | 1.35E+08 | 1 | RCC2 |
| ENSBTAG00000045172 | 2 | 1.35E+08 | 1.35E+08 | 1 | bta-mir-2358 |
| ENSBTAG00000038945 | 2 | 1.35E+08 | 1.35E+08 | -1 | PADI6 |
| ENSBTAG00000012052 | 2 | 1.35E+08 | 1.35E+08 | -1 | PADI4 |
| ENSBTAG00000012043 | 2 | 1.35E+08 | 1.35E+08 | -1 | PADI3 |
| ENSBTAG00000002138 | 2 | 1.35E+08 | 1.35E+08 | -1 | PADI1 |
| ENSBTAG00000043637 | 2 | 1.35E+08 | 1.35E+08 | 1 | 5S_rRNA |
| ENSBTAG00000003403 | 2 | 1.36E+08 | 1.35E+08 | 1 | PADI2 |
| ENSBTAG00000008314 | 2 | 1.36E+08 | 1.36E+08 | 1 | SDHB |
| ENSBTAG00000008309 | 2 | 1.36E+08 | 1.36E+08 | 1 | ATP13A2 |
| ENSBTAG00000003832 | 2 | 1.36E+08 | 1.36E+08 | 1 | MFAP2 |
| ENSBTAG00000003822 | 2 | 1.36E+08 | 1.36E+08 | -1 | CROCC |
| ENSBTAG00000013282 | 2 | 1.36E+08 | 1.36E+08 | -1 | NECAP2 |
| ENSBTAG00000013281 | 2 | 1.36E+08 | 1.36E+08 | 1 | SPATA21 |
| ENSBTAG00000009251 | 2 | 1.36E+08 | 1.36E+08 | -1 | SZRD1 |
| ENSBTAG00000051080 | 2 | 1.36E+08 | 1.36E+08 | 1 |  |
| ENSBTAG00000052646 | 2 | 1.36E+08 | 1.36E+08 | 1 |  |
| ENSBTAG00000008510 | 2 | 1.36E+08 | 1.36E+08 | 1 | FBXO42 |
| ENSBTAG00000014221 | 2 | 1.36E+08 | 1.36E+08 | 1 | CPLANE2 |
| ENSBTAG00000005670 | 2 | 1.36E+08 | 1.36E+08 | 1 | ARHGEF19 |
| ENSBTAG00000000815 | 2 | 1.36E+08 | 1.36E+08 | 1 | EPHA2 |
| ENSBTAG00000053870 | 2 | 1.36E+08 | 1.36E+08 | 1 | bta-mir-12026-1 |
| ENSBTAG00000034680 | 2 | 1.36E+08 | 1.36E+08 | 1 | FAM131C |
| ENSBTAG00000048882 | 2 | 1.36E+08 | 1.36E+08 | -1 | U6 |
| ENSBTAG00000034674 | 2 | 1.36E+08 | 1.36E+08 | -1 |  |
| ENSBTAG00000055045 | 2 | 1.36E+08 | 1.36E+08 | 1 | HSPB7 |
| ENSBTAG00000010938 | 2 | 1.36E+08 | 1.36E+08 | -1 | SRARP |
| ENSBTAG00000048513 | 2 | 1.36E+08 | 1.36E+08 | 1 |  |
| ENSBTAG00000031918 | 2 | 74228632 | 74228030 | -1 |  |
| ENSBTAG00000050710 | 2 | 75113317 | 75111884 | -1 |  |
| ENSBTAG00000054992 | 2 | 75747947 | 75745327 | 1 |  |
| ENSBTAG00000031898 | 2 | 76921405 | 76113318 | 1 | CNTNAP5 |
| ENSBTAG00000049994 | 2 | 77049170 | 77047344 | 1 |  |
| ENSBTAG00000051403 | 2 | 77263098 | 77262460 | 1 |  |
| ENSBTAG00000044835 | 2 | 78876535 | 78876480 | -1 |  |
| ENSBTAG00000014863 | 2 | 78934052 | 78885394 | 1 | GYPC |
| ENSBTAG00000046468 | 20 | 21799446 | 21798316 | 1 | ACTBL2 |
| ENSBTAG00000051426 | 20 | 21823478 | 21823390 | 1 | bta-mir-2285f-2 |
| ENSBTAG00000042330 | 20 | 21869861 | 21869775 | 1 |  |
| ENSBTAG00000026505 | 20 | 21932086 | 21931661 | 1 |  |
| ENSBTAG00000012124 | 20 | 22080506 | 22017006 | -1 | GPBP1 |
| ENSBTAG00000053164 | 20 | 22122022 | 22121630 | 1 |  |
| ENSBTAG00000014248 | 20 | 22305654 | 22279228 | 1 | MIER3 |
| ENSBTAG00000013426 | 20 | 22323346 | 22314474 | -1 | SETD9 |
| ENSBTAG00000036766 | 20 | 22331024 | 22330873 | 1 | U1 |
| ENSBTAG00000013790 | 20 | 22417428 | 22340163 | -1 | MAP3K1 |
| ENSBTAG00000049279 | 20 | 22454400 | 22451775 | -1 |  |
| ENSBTAG00000051954 | 20 | 22600229 | 22561143 | 1 |  |
| ENSBTAG00000043083 | 20 | 22781295 | 22781192 | -1 | U6 |
| ENSBTAG00000054651 | 20 | 22868589 | 22858616 | -1 |  |
| ENSBTAG00000001001 | 20 | 23096396 | 22986548 | 1 | ANKRD55 |
| ENSBTAG00000051655 | 20 | 23113536 | 23104056 | 1 |  |
| ENSBTAG00000017745 | 20 | 23250938 | 23191785 | 1 | IL6ST |
| ENSBTAG00000052935 | 20 | 23198922 | 23198844 | 1 | bta-mir-2359 |
| ENSBTAG00000004351 | 20 | 23352421 | 23285000 | -1 | IL31RA |
| ENSBTAG00000008871 | 20 | 23424574 | 23358630 | -1 | DDX4 |
| ENSBTAG00000033313 | 20 | 23542895 | 23440943 | 1 | SLC38A9 |
| ENSBTAG00000044567 | 20 | 23518094 | 23518008 | 1 |  |
| ENSBTAG00000010526 | 20 | 23738916 | 23636355 | 1 | PLPP1 |
| ENSBTAG00000010518 | 20 | 23830250 | 23738862 | -1 | MTREX |
| ENSBTAG00000010510 | 20 | 23877461 | 23830149 | 1 | DHX29 |
| ENSBTAG00000048821 | 20 | 23879254 | 23878055 | -1 |  |
| ENSBTAG00000047594 | 20 | 23903855 | 23901697 | 1 | CCNO |
| ENSBTAG00000047087 | 20 | 23913997 | 23907747 | 1 | MCIDAS |
| ENSBTAG00000048418 | 20 | 23937332 | 23921650 | -1 |  |
| ENSBTAG00000021959 | 20 | 24005261 | 23940884 | 1 | CDC20B |
| ENSBTAG00000030051 | 20 | 23941804 | 23941700 | 1 | bta-mir-449c |
| ENSBTAG00000030027 | 20 | 23943289 | 23943192 | 1 | bta-mir-449b |
| ENSBTAG00000030121 | 20 | 23943403 | 23943316 | 1 | bta-mir-449a |
| ENSBTAG00000021960 | 20 | 23956371 | 23951563 | -1 | GPX8 |
| ENSBTAG00000021958 | 20 | 24018787 | 24009814 | -1 | GZMA |
| ENSBTAG00000027865 | 20 | 24035304 | 24024507 | -1 | gzmA |
| ENSBTAG00000017746 | 20 | 1163533 | 447373 | -1 | SLIT3 |
| ENSBTAG00000008180 | 20 | 1510229 | 1477149 | 1 | SPDL1 |
| ENSBTAG00000014612 | 20 | 2001666 | 1562432 | 1 | DOCK2 |
| ENSBTAG00000002134 | 20 | 1792301 | 1768224 | -1 | INSYN2B |
| ENSBTAG00000014620 | 20 | 2016056 | 2012271 | 1 | FOXI1 |
| ENSBTAG00000043132 | 20 | 2032365 | 2032260 | -1 | U6 |
| ENSBTAG00000009381 | 20 | 2179242 | 2132959 | -1 | LCP2 |
| ENSBTAG00000002985 | 20 | 2270188 | 2255766 | -1 | KCNMB1 |
| ENSBTAG00000053195 | 20 | 2373165 | 2373109 | 1 | bta-mir-12032 |
| ENSBTAG00000002865 | 20 | 2641564 | 2381094 | 1 | KCNIP1 |
| ENSBTAG00000002779 | 20 | 2707201 | 2680474 | 1 | GABRP |
| ENSBTAG00000024801 | 20 | 3149145 | 2765576 | 1 | RANBP17 |
| ENSBTAG00000010003 | 20 | 3161648 | 3158996 | 1 | TLX3 |
| ENSBTAG00000015316 | 20 | 3215788 | 3205593 | 1 | NPM1 |
| ENSBTAG00000000128 | 20 | 3263206 | 3226532 | 1 | FGF18 |
| ENSBTAG00000054615 | 20 | 3252756 | 3252364 | -1 |  |
| ENSBTAG00000042429 | 20 | 3339972 | 3339868 | 1 | U6 |
| ENSBTAG00000053610 | 20 | 3632978 | 3630355 | -1 |  |
| ENSBTAG00000015376 | 20 | 3809572 | 3678207 | -1 | FBXW11 |
| ENSBTAG00000017457 | 20 | 3975388 | 3852216 | -1 | STK10 |
| ENSBTAG00000008876 | 20 | 3992285 | 3986718 | 1 | EFCAB9 |
| ENSBTAG00000034659 | 20 | 4064029 | 3997984 | -1 | UBTD2 |
| ENSBTAG00000009019 | 20 | 4223391 | 4104991 | -1 | SH3PXD2B |
| ENSBTAG00000001429 | 20 | 4448471 | 4408001 | 1 | NEURL1B |
| ENSBTAG00000013863 | 20 | 4543611 | 4540518 | -1 | DUSP1 |
| ENSBTAG00000015955 | 20 | 4709455 | 4596395 | 1 | ERGIC1 |
| ENSBTAG00000015099 | 20 | 4723653 | 4716985 | 1 | RPL26L1 |
| ENSBTAG00000015100 | 20 | 4762537 | 4732524 | 1 | ATP6V0E1 |
| ENSBTAG00000002020 | 20 | 4831767 | 4776513 | 1 | CREBRF |
| ENSBTAG00000002021 | 20 | 4854725 | 4841533 | 1 | BNIP1 |
| ENSBTAG00000050983 | 20 | 4898205 | 4898087 | 1 | 5S_rRNA |
| ENSBTAG00000020568 | 20 | 4913786 | 4910166 | -1 | NKX2-5 |
| ENSBTAG00000048827 | 20 | 4924449 | 4923319 | 1 |  |
| ENSBTAG00000001823 | 20 | 5011125 | 4999725 | -1 | STC2 |
| ENSBTAG00000034598 | 20 | 5331743 | 5319518 | -1 | BOD1 |
| ENSBTAG00000049048 | 20 | 5380258 | 5332424 | 1 |  |
| ENSBTAG00000050409 | 20 | 5390985 | 5388843 | 1 |  |
| ENSBTAG00000049683 | 20 | 5493229 | 5490442 | 1 |  |
| ENSBTAG00000009995 | 20 | 5678507 | 5606065 | 1 | CPEB4 |
| ENSBTAG00000049650 | 20 | 5628553 | 5628141 | -1 |  |
| ENSBTAG00000054459 | 20 | 5720066 | 5702887 | 1 | C20H5orf47 |
| ENSBTAG00000000564 | 20 | 5822147 | 5750258 | 1 | NSG2 |
| ENSBTAG00000048786 | 20 | 5765704 | 5763714 | -1 |  |
| ENSBTAG00000030064 | 20 | 6324167 | 6324092 | -1 | bta-mir-584-6 |
| ENSBTAG00000013873 | 20 | 6449312 | 6444184 | 1 | MSX2 |
| ENSBTAG00000011828 | 21 | 34131883 | 34083678 | -1 | ARID3B |
| ENSBTAG00000049104 | 21 | 34181985 | 34181656 | 1 |  |
| ENSBTAG00000000010 | 21 | 34223394 | 34209956 | 1 | UBL7 |
| ENSBTAG00000011446 | 21 | 34258160 | 34234871 | 1 | SEMA7A |
| ENSBTAG00000006934 | 21 | 34342900 | 34328403 | 1 | CYP11A1 |
| ENSBTAG00000006934 | 21 | 34342900 | 34328403 | 1 | CYP11A1 |
| ENSBTAG00000006934 | 21 | 34342900 | 34328403 | 1 | CYP11A1 |
| ENSBTAG00000006934 | 21 | 34342900 | 34328403 | 1 | CYP11A1 |
| ENSBTAG00000006912 | 21 | 34440515 | 34401527 | -1 |  |
| ENSBTAG00000050030 | 21 | 34473017 | 34464938 | -1 |  |
| ENSBTAG00000007348 | 21 | 34507708 | 34477952 | 1 | STRA6 |
| ENSBTAG00000006024 | 21 | 34512801 | 34509672 | -1 | ISLR |
| ENSBTAG00000002661 | 21 | 34560183 | 34556000 | -1 | ISLR2 |
| ENSBTAG00000051314 | 21 | 34563739 | 34561706 | 1 |  |
| ENSBTAG00000015779 | 21 | 34630988 | 34575149 | -1 | PML |
| ENSBTAG00000015772 | 21 | 34641526 | 34633182 | 1 | STOML1 |
| ENSBTAG00000009086 | 21 | 34687592 | 34663151 | -1 | LOXL1 |
| ENSBTAG00000052449 | 21 | 34720610 | 34717625 | -1 |  |
| ENSBTAG00000050871 | 21 | 34730647 | 34727438 | 1 |  |
| ENSBTAG00000053015 | 21 | 34743331 | 34740311 | 1 | GZMB |
| ENSBTAG00000053015 | 21 | 34743331 | 34740311 | 1 | GZMB |
| ENSBTAG00000039813 | 21 | 34754746 | 34746523 | 1 |  |
| ENSBTAG00000038159 | 21 | 34758193 | 34755350 | 1 |  |
| ENSBTAG00000050353 | 21 | 34763098 | 34761185 | 1 |  |
| ENSBTAG00000040134 | 21 | 34776915 | 34774233 | 1 |  |
| ENSBTAG00000010828 | 21 | 34814493 | 34811403 | -1 | GZMH |
| ENSBTAG00000013055 | 21 | 34818847 | 34816886 | -1 |  |
| ENSBTAG00000038080 | 21 | 34837188 | 34835231 | -1 |  |
| ENSBTAG00000010057 | 21 | 34880233 | 34876603 | -1 | GZMB |
| ENSBTAG00000021291 | 21 | 35267297 | 35027776 | -1 | STXBP6 |
| ENSBTAG00000054263 | 21 | 36161541 | 36012637 | -1 |  |
| ENSBTAG00000002170 | 21 | 36879180 | 36714178 | -1 | NOVA1 |
| ENSBTAG00000045428 | 21 | 37728838 | 37728737 | -1 | U6 |
| ENSBTAG00000031558 | 21 | 37876419 | 37872895 | -1 |  |
| ENSBTAG00000011113 | 21 | 39242646 | 39241174 | 1 | FOXG1 |
| ENSBTAG00000012789 | 21 | 40460921 | 40114486 | -1 | PRKD1 |
| ENSBTAG00000045322 | 21 | 40190081 | 40189964 | 1 | 5S_rRNA |
| ENSBTAG00000001617 | 21 | 41262640 | 41190632 | 1 | G2E3 |
| ENSBTAG00000017565 | 21 | 41381665 | 41268558 | 1 | SCFD1 |
| ENSBTAG00000021844 | 21 | 41540316 | 41526381 | 1 | COCH |
| ENSBTAG00000021845 | 21 | 41650684 | 41543030 | -1 | STRN3 |
| ENSBTAG00000017201 | 21 | 41697665 | 41679370 | 1 | AP4S1 |
| ENSBTAG00000032477 | 21 | 41776463 | 41701589 | -1 | HECTD1 |
| ENSBTAG00000022847 | 21 | 41936150 | 41836061 | -1 | HEATR5A |
| ENSBTAG00000042753 | 21 | 41875823 | 41875675 | -1 | SNORA62 |
| ENSBTAG00000038020 | 21 | 41968785 | 41959757 | -1 |  |
| ENSBTAG00000010536 | 21 | 41986030 | 41985008 | -1 | GPR33 |
| ENSBTAG00000044121 | 21 | 42376482 | 42056643 | 1 | NUBPL |
| ENSBTAG00000028324 | 21 | 42138066 | 42137944 | -1 | 5S_rRNA |
| ENSBTAG00000050424 | 21 | 42393726 | 42380070 | 1 |  |
| ENSBTAG00000055201 | 21 | 42435867 | 42435451 | 1 |  |
| ENSBTAG00000027024 | 21 | 42581786 | 42505542 | 1 | ARHGAP5 |
| ENSBTAG00000050552 | 21 | 42615125 | 42615019 | -1 | U6 |
| ENSBTAG00000044894 | 21 | 42624577 | 42624484 | -1 | Vault |
| ENSBTAG00000017719 | 21 | 43212928 | 42714751 | 1 | AKAP6 |
| ENSBTAG00000054217 | 21 | 42798277 | 42798171 | -1 | U6 |
| ENSBTAG00000050350 | 21 | 43392502 | 43392429 | 1 | bta-mir-6522 |
| ENSBTAG00000004462 | 21 | 44285742 | 44020190 | 1 | NPAS3 |
| ENSBTAG00000008172 | 21 | 44416154 | 44387137 | -1 | EGLN3 |
| ENSBTAG00000050690 | 21 | 44581301 | 44430980 | -1 |  |
| ENSBTAG00000054203 | 21 | 44480383 | 44480324 | -1 | bta-mir-2285cy |
| ENSBTAG00000051703 | 21 | 44671226 | 44652884 | 1 |  |
| ENSBTAG00000013981 | 21 | 44901774 | 44882558 | -1 | SPTSSA |
| ENSBTAG00000053208 | 21 | 44931313 | 44930950 | -1 |  |
| ENSBTAG00000003419 | 21 | 44951180 | 44934146 | -1 | EAPP |
| ENSBTAG00000051054 | 21 | 44955335 | 44955172 | -1 | U1 |
| ENSBTAG00000049660 | 21 | 44959261 | 44959098 | 1 | U1 |
| ENSBTAG00000037013 | 21 | 44968121 | 44967957 | 1 | U1 |
| ENSBTAG00000028419 | 21 | 44981500 | 44981342 | -1 | U1 |
| ENSBTAG00000055182 | 21 | 44987947 | 44987784 | -1 | U1 |
| ENSBTAG00000051603 | 21 | 44996271 | 44996108 | 1 | U1 |
| ENSBTAG00000012873 | 21 | 45046871 | 44998853 | -1 | SNX6 |
| ENSBTAG00000037179 | 21 | 45017330 | 45017108 | -1 | U3 |
| ENSBTAG00000015053 | 21 | 45101864 | 45099213 | -1 | CFL2 |
| ENSBTAG00000020164 | 21 | 45202855 | 45116769 | -1 | BAZ1A |
| ENSBTAG00000037938 | 21 | 45301018 | 45271631 | -1 |  |
| ENSBTAG00000019085 | 21 | 45337119 | 45308844 | 1 | SRP54 |
| ENSBTAG00000000425 | 21 | 45342812 | 45341894 | 1 |  |
| ENSBTAG00000009680 | 21 | 45368962 | 45352820 | 1 | FAM177A1 |
| ENSBTAG00000009681 | 21 | 45395367 | 45369633 | -1 | PPP2R3C |
| ENSBTAG00000009682 | 21 | 45527537 | 45395271 | 1 | PRORP |
| ENSBTAG00000043216 | 21 | 45476293 | 45476187 | -1 | U6 |
| ENSBTAG00000049349 | 21 | 45491594 | 45490576 | -1 |  |
| ENSBTAG00000009683 | 21 | 45572660 | 45548878 | 1 | PSMA6 |
| ENSBTAG00000016683 | 21 | 45643131 | 45639133 | -1 | NFKBIA |
| ENSBTAG00000042175 | 21 | 45652233 | 45652127 | -1 | U6 |
| ENSBTAG00000027013 | 21 | 45773604 | 45771940 | 1 | INSM2 |
| ENSBTAG00000001282 | 21 | 45986337 | 45775690 | -1 | RALGAPA1 |
| ENSBTAG00000049307 | 21 | 45975372 | 45975266 | 1 | U6 |
| ENSBTAG00000049674 | 21 | 45986743 | 45986113 | 1 |  |
| ENSBTAG00000018400 | 21 | 46058395 | 46014364 | 1 | BRMS1L |
| ENSBTAG00000009941 | 21 | 46507168 | 46488245 | -1 | MBIP |
| ENSBTAG00000049414 | 21 | 46698732 | 46671142 | -1 |  |
| ENSBTAG00000000541 | 21 | 46705898 | 46701290 | -1 | NKX2-1 |
| ENSBTAG00000013352 | 21 | 46769486 | 46767484 | -1 | NKX2-8 |
| ENSBTAG00000009186 | 21 | 46877480 | 46860164 | 1 | PAX9 |
| ENSBTAG00000019350 | 21 | 47400026 | 46881642 | -1 | SLC25A21 |
| ENSBTAG00000054035 | 21 | 47253538 | 46889946 | 1 |  |
| ENSBTAG00000000655 | 21 | 47734138 | 47398496 | 1 | MIPOL1 |
| ENSBTAG00000013888 | 21 | 47782907 | 47777670 | -1 | FOXA1 |
| ENSBTAG00000015095 | 21 | 47998810 | 47810137 | 1 | TTC6 |
| ENSBTAG00000006582 | 21 | 48351072 | 48348493 | 1 | SSTR1 |
| ENSBTAG00000006130 | 21 | 48395926 | 48394033 | -1 | CLEC14A |
| ENSBTAG00000003708 | 21 | 49103406 | 49038721 | -1 | SEC23A |
| ENSBTAG00000051859 | 21 | 49078735 | 49074122 | 1 |  |
| ENSBTAG00000020930 | 21 | 49140569 | 49119054 | 1 | GEMIN2 |
| ENSBTAG00000054660 | 21 | 49139052 | 49137418 | -1 |  |
| ENSBTAG00000015023 | 21 | 49156067 | 49145029 | -1 | TRAPPC6B |
| ENSBTAG00000026995 | 21 | 49166919 | 49159484 | 1 | PNN |
| ENSBTAG00000006819 | 21 | 49281283 | 49184308 | 1 |  |
| ENSBTAG00000006836 | 21 | 49310143 | 49289714 | -1 | FBXO33 |
| ENSBTAG00000055122 | 21 | 49839399 | 49839321 | 1 | bta-mir-2365 |
| ENSBTAG00000003684 | 21 | 51648277 | 51329040 | 1 | LRFN5 |
| ENSBTAG00000043018 | 21 | 51620482 | 51620343 | 1 | U4 |
| ENSBTAG00000006363 | 21 | 52287484 | 52283866 | -1 |  |
| ENSBTAG00000054715 | 21 | 52789035 | 52788688 | -1 |  |
| ENSBTAG00000048948 | 21 | 52870614 | 52870542 | -1 |  |
| ENSBTAG00000051424 | 21 | 53534944 | 53534525 | -1 |  |
| ENSBTAG00000013117 | 22 | 46818511 | 45924535 | -1 | CACNA2D3 |
| ENSBTAG00000051702 | 22 | 46706548 | 46706442 | -1 | U6 |
| ENSBTAG00000044233 | 22 | 46847933 | 46847840 | -1 | U6 |
| ENSBTAG00000015149 | 22 | 46920844 | 46919070 | 1 |  |
| ENSBTAG00000032374 | 22 | 47031572 | 47024359 | 1 | SELENOK |
| ENSBTAG00000011180 | 22 | 47056658 | 47032971 | 1 | ACTR8 |
| ENSBTAG00000011178 | 22 | 47066805 | 47051292 | -1 | IL17RB |
| ENSBTAG00000010027 | 22 | 47158681 | 47133914 | 1 | CHDH |
| ENSBTAG00000010026 | 22 | 47508559 | 47162857 | -1 | CACNA1D |
| ENSBTAG00000032347 | 22 | 47589179 | 47588803 | 1 |  |
| ENSBTAG00000053819 | 22 | 47616393 | 47616203 | 1 | U2 |
| ENSBTAG00000010863 | 22 | 47690569 | 47646509 | 1 | DCP1A |
| ENSBTAG00000052138 | 22 | 47694387 | 47693377 | -1 |  |
| ENSBTAG00000003758 | 22 | 47731710 | 47707405 | 1 | TKT |
| ENSBTAG00000008719 | 22 | 47780548 | 47749990 | -1 | PRKCD |
| ENSBTAG00000020973 | 22 | 47852306 | 47812822 | 1 | RFT1 |
| ENSBTAG00000011345 | 22 | 47992605 | 47877481 | 1 | SFMBT1 |
| ENSBTAG00000011344 | 22 | 48051428 | 47999883 | 1 | TMEM110 |
| ENSBTAG00000032531 | 22 | 48057235 | 48056089 | 1 | MUSTN1 |
| ENSBTAG00000007850 | 22 | 48079899 | 48059508 | 1 | ITIH4 |
| ENSBTAG00000007846 | 22 | 48091969 | 48078023 | -1 | ITIH3 |
| ENSBTAG00000007843 | 22 | 48113488 | 48099219 | -1 | ITIH1 |
| ENSBTAG00000004749 | 22 | 48152515 | 48124642 | 1 | NEK4 |
| ENSBTAG00000043291 | 22 | 48133273 | 48133148 | -1 |  |
| ENSBTAG00000053918 | 22 | 48157947 | 48155389 | -1 | SPCS1 |
| ENSBTAG00000014550 | 22 | 48169414 | 48157167 | 1 | GLT8D1 |
| ENSBTAG00000014549 | 22 | 48175878 | 48169659 | -1 | GNL3 |
| ENSBTAG00000043140 | 22 | 48171234 | 48171158 | -1 | SNORD69 |
| ENSBTAG00000043914 | 22 | 48171845 | 48171770 | -1 | SNORD19C |
| ENSBTAG00000044972 | 22 | 48172301 | 48172220 | -1 | SNORD19B |
| ENSBTAG00000042117 | 22 | 48173104 | 48173028 | -1 | SNORD19 |
| ENSBTAG00000014786 | 22 | 48283003 | 48176132 | 1 | PBRM1 |
| ENSBTAG00000044079 | 22 | 48289693 | 48286485 | -1 | SMIM4 |
| ENSBTAG00000014784 | 22 | 48343007 | 48291599 | 1 | NT5DC2 |
| ENSBTAG00000014782 | 22 | 48368328 | 48343002 | -1 | STAB1 |
| ENSBTAG00000014779 | 22 | 48432781 | 48370379 | -1 | NISCH |
| ENSBTAG00000045757 | 22 | 48437765 | 48434632 | 1 | TNNC1 |
| ENSBTAG00000046924 | 22 | 48455607 | 48445745 | 1 | SEMA3G |
| ENSBTAG00000047658 | 22 | 48479025 | 48466090 | -1 | PHF7 |
| ENSBTAG00000045817 | 22 | 48487708 | 48479063 | 1 | BAP1 |
| ENSBTAG00000047794 | 22 | 48533380 | 48488285 | -1 | DNAH1 |
| ENSBTAG00000054920 | 22 | 48561179 | 48534894 | -1 |  |
| ENSBTAG00000029825 | 22 | 48588990 | 48588901 | 1 | bta-mir-135a-1 |
| ENSBTAG00000021695 | 22 | 48595068 | 48589696 | -1 | GLYCTK |
| ENSBTAG00000002960 | 22 | 48648118 | 48629513 | 1 | WDR82 |
| ENSBTAG00000029861 | 22 | 48636497 | 48636391 | 1 | MIRLET7G |
| ENSBTAG00000002962 | 22 | 48658495 | 48649439 | -1 | PPM1M |
| ENSBTAG00000054131 | 22 | 48674430 | 48665158 | 1 | TWF2 |
| ENSBTAG00000018198 | 22 | 48680938 | 48676660 | 1 | TLR9 |
| ENSBTAG00000004118 | 22 | 48704126 | 48689674 | -1 | ALAS1 |
| ENSBTAG00000001471 | 22 | 48805972 | 48705195 | 1 | POC1A |
| ENSBTAG00000021912 | 22 | 48832342 | 48825120 | 1 | DUSP7 |
| ENSBTAG00000018628 | 22 | 48947321 | 48945144 | 1 | RPL29 |
| ENSBTAG00000032304 | 22 | 48953164 | 48948303 | -1 | ACY1 |
| ENSBTAG00000053854 | 22 | 48957705 | 48954795 | -1 | ABHD14A |
| ENSBTAG00000037377 | 22 | 48965810 | 48962168 | 1 | ABHD14B |
| ENSBTAG00000018622 | 22 | 48976850 | 48966805 | 1 | PCBP4 |
| ENSBTAG00000013442 | 22 | 48978925 | 48976816 | -1 | GPR62 |
| ENSBTAG00000000671 | 22 | 48989507 | 48984160 | -1 | PARP3 |
| ENSBTAG00000000670 | 22 | 48998979 | 48990198 | 1 | RRP9 |
| ENSBTAG00000032279 | 22 | 49029107 | 48997519 | -1 | IQCF2 |
| ENSBTAG00000000805 | 22 | 49006034 | 49003339 | 1 | IQCF1 |
| ENSBTAG00000040574 | 22 | 49013783 | 49011889 | 1 | IQCF5 |
| ENSBTAG00000054379 | 22 | 49041106 | 49034196 | -1 |  |
| ENSBTAG00000037410 | 22 | 49052860 | 49048876 | -1 |  |
| ENSBTAG00000045942 | 22 | 49079635 | 49075129 | 1 | IQCF6 |
| ENSBTAG00000039664 | 22 | 49105932 | 49105357 | 1 |  |
| ENSBTAG00000001067 | 22 | 49127374 | 49118416 | -1 | GRM2 |
| ENSBTAG00000034796 | 22 | 49162180 | 49131857 | -1 | TEX264 |
| ENSBTAG00000032253 | 22 | 49277563 | 49169587 | -1 | RAD54L2 |
| ENSBTAG00000006501 | 22 | 49370178 | 49282339 | 1 | DCAF1 |
| ENSBTAG00000006500 | 22 | 49377717 | 49374567 | -1 | RBM15B |
| ENSBTAG00000031797 | 22 | 49383627 | 49379095 | -1 | MANF |
| ENSBTAG00000001874 | 22 | 49668868 | 49387098 | -1 | DOCK3 |
| ENSBTAG00000047517 | 22 | 49688248 | 49688187 | -1 |  |
| ENSBTAG00000016532 | 22 | 49740232 | 49711610 | -1 | MAPKAPK3 |
| ENSBTAG00000022622 | 22 | 49755490 | 49749512 | 1 | CISH |
| ENSBTAG00000004120 | 22 | 49789516 | 49767036 | -1 | HEMK1 |
| ENSBTAG00000010165 | 22 | 49799038 | 49790596 | 1 | C22H3orf18 |
| ENSBTAG00000009489 | 22 | 49986489 | 49872343 | 1 | CACNA2D2 |
| ENSBTAG00000019161 | 22 | 50001031 | 49988945 | 1 | NPRL2 |
| ENSBTAG00000019163 | 22 | 49997571 | 49994656 | -1 | CYB561D2 |
| ENSBTAG00000020965 | 22 | 50006742 | 50002503 | 1 | ZMYND10 |
| ENSBTAG00000020963 | 22 | 50015926 | 50006693 | 1 | RASSF1 |
| ENSBTAG00000000484 | 22 | 50026835 | 50016339 | 1 | HYAL2 |
| ENSBTAG00000000483 | 22 | 50032903 | 50029859 | 1 | HYAL1 |
| ENSBTAG00000052388 | 22 | 50039436 | 50034120 | 1 | HYAL3 |
| ENSBTAG00000038084 | 22 | 50036349 | 50035332 | 1 | NAA80 |
| ENSBTAG00000000480 | 22 | 50066774 | 50039840 | 1 | IFRD2 |
| ENSBTAG00000000478 | 22 | 50069527 | 50067283 | -1 | LSMEM2 |
| ENSBTAG00000010138 | 22 | 50093377 | 50084781 | -1 | SEMA3B |
| ENSBTAG00000020645 | 22 | 50120260 | 50099960 | -1 | GNAI2 |
| ENSBTAG00000008509 | 22 | 50146728 | 50131869 | -1 | SLC38A3 |
| ENSBTAG00000018020 | 22 | 50157613 | 50152601 | -1 | GNAT1 |
| ENSBTAG00000018307 | 22 | 50190175 | 50161374 | -1 | SEMA3F |
| ENSBTAG00000006330 | 22 | 50242674 | 50218344 | -1 | RBM5 |
| ENSBTAG00000042160 | 22 | 50244533 | 50244428 | 1 | U6 |
| ENSBTAG00000006328 | 22 | 50336220 | 50246619 | -1 | RBM6 |
| ENSBTAG00000053287 | 22 | 50274445 | 50272871 | 1 |  |
| ENSBTAG00000054602 | 22 | 50335875 | 50335610 | -1 |  |
| ENSBTAG00000020047 | 22 | 50353722 | 50341652 | 1 | MON1A |
| ENSBTAG00000015046 | 22 | 50370270 | 50357168 | 1 | MST1R |
| ENSBTAG00000040083 | 22 | 50382736 | 50379122 | 1 | ACTL11 |
| ENSBTAG00000004878 | 22 | 50397870 | 50386354 | 1 | CAMKV |
| ENSBTAG00000004877 | 22 | 50417063 | 50399202 | 1 | TRAIP |
| ENSBTAG00000012335 | 22 | 50430092 | 50420590 | 1 | UBA7 |
| ENSBTAG00000012322 | 22 | 50432582 | 50430296 | -1 | INKA1 |
| ENSBTAG00000005728 | 22 | 50441745 | 50434292 | 1 | CDHR4 |
| ENSBTAG00000011595 | 22 | 50481306 | 50443820 | 1 | IP6K1 |
| ENSBTAG00000032026 | 22 | 50484009 | 50481628 | 1 | GMPPB |
| ENSBTAG00000011588 | 22 | 50507304 | 50484030 | -1 | RNF123 |
| ENSBTAG00000045547 | 22 | 50487389 | 50485830 | 1 | AMIGO3 |
| ENSBTAG00000011585 | 22 | 50514786 | 50509885 | 1 | MST1 |
| ENSBTAG00000011583 | 22 | 50525786 | 50515197 | -1 | APEH |
| ENSBTAG00000011581 | 22 | 50568056 | 50533323 | -1 | BSN |
| ENSBTAG00000011580 | 22 | 50663265 | 50616593 | -1 | DAG1 |
| ENSBTAG00000000387 | 22 | 50692789 | 50688543 | 1 | NICN1 |
| ENSBTAG00000002321 | 22 | 50697601 | 50693259 | 1 | AMT |
| ENSBTAG00000022632 | 22 | 50700780 | 50697897 | -1 | TCTA |
| ENSBTAG00000004279 | 22 | 50751132 | 50701226 | 1 | RHOA |
| ENSBTAG00000054195 | 22 | 50753719 | 50752499 | 1 | GPX1 |
| ENSBTAG00000044873 | 22 | 50755854 | 50755752 | -1 | U6 |
| ENSBTAG00000011899 | 22 | 50803467 | 50762625 | 1 | USP4 |
| ENSBTAG00000011894 | 22 | 50809723 | 50803150 | 1 | C22H3orf62 |
| ENSBTAG00000009371 | 22 | 50840894 | 50812881 | -1 | IHO1 |
| ENSBTAG00000000982 | 22 | 50854259 | 50846267 | 1 | C22H3orf84 |
| ENSBTAG00000000981 | 22 | 50859766 | 50854957 | -1 | KLHDC8B |
| ENSBTAG00000013932 | 22 | 50866898 | 50863214 | 1 | CCDC71 |
| ENSBTAG00000055290 | 22 | 50882602 | 50867110 | 1 |  |
| ENSBTAG00000022635 | 22 | 50897890 | 50883645 | 1 | LAMB2 |
| ENSBTAG00000018921 | 22 | 50909096 | 50899782 | 1 | USP19 |
| ENSBTAG00000049609 | 22 | 50908557 | 50908307 | 1 |  |
| ENSBTAG00000018928 | 22 | 50919173 | 50911689 | 1 | QARS1 |
| ENSBTAG00000018924 | 22 | 50962568 | 50920376 | 1 | QRICH1 |
| ENSBTAG00000031837 | 22 | 50967685 | 50962854 | 1 | IMPDH2 |
| ENSBTAG00000018918 | 22 | 50969630 | 50968509 | -1 | NDUFAF3 |
| ENSBTAG00000029957 | 22 | 50971083 | 50970980 | 1 | MIR191 |
| ENSBTAG00000029913 | 22 | 50971548 | 50971462 | 1 | bta-mir-425 |
| ENSBTAG00000018913 | 22 | 50975586 | 50972627 | 1 | DALRD3 |
| ENSBTAG00000018910 | 22 | 50983657 | 50975112 | -1 | WDR6 |
| ENSBTAG00000005490 | 22 | 50997485 | 50983772 | -1 | P4HTM |
| ENSBTAG00000005488 | 22 | 51038025 | 51000461 | -1 | ARIH2 |
| ENSBTAG00000000191 | 22 | 51080116 | 51045061 | 1 | SLC25A20 |
| ENSBTAG00000014205 | 22 | 51155609 | 51085591 | 1 | PRKAR2A |
| ENSBTAG00000021853 | 22 | 51199445 | 51175603 | 1 | IP6K2 |
| ENSBTAG00000021850 | 22 | 51213764 | 51201255 | 1 | NCKIPSD |
| ENSBTAG00000021846 | 22 | 51248173 | 51220718 | 1 | CELSR3 |
| ENSBTAG00000038381 | 22 | 51259943 | 51249161 | 1 | SLC26A6 |
| ENSBTAG00000021390 | 22 | 51264015 | 51261961 | 1 | TMEM89 |
| ENSBTAG00000019096 | 22 | 51283826 | 51275073 | 1 | UQCRC1 |
| ENSBTAG00000019081 | 22 | 51317102 | 51287420 | 1 | COL7A1 |
| ENSBTAG00000049917 | 22 | 51297669 | 51296725 | 1 |  |
| ENSBTAG00000052088 | 22 | 51303302 | 51302648 | 1 |  |
| ENSBTAG00000049028 | 22 | 51310614 | 51309396 | 1 |  |
| ENSBTAG00000019078 | 22 | 51319066 | 51318728 | 1 | UCN2 |
| ENSBTAG00000006752 | 22 | 51363442 | 51322004 | 1 | PFKFB4 |
| ENSBTAG00000009183 | 22 | 51399168 | 51371186 | 1 | SHISA5 |
| ENSBTAG00000048942 | 22 | 51391480 | 51389456 | -1 |  |
| ENSBTAG00000008406 | 22 | 51401262 | 51399388 | -1 | TREX1 |
| ENSBTAG00000008399 | 22 | 51419086 | 51401422 | -1 | ATRIP |
| ENSBTAG00000053755 | 22 | 51423482 | 51421537 | -1 | TMA7 |
| ENSBTAG00000013413 | 22 | 51433253 | 51422933 | 1 | CCDC51 |
| ENSBTAG00000013407 | 22 | 51463440 | 51439234 | 1 | PLXNB1 |
| ENSBTAG00000053231 | 22 | 51517679 | 51513513 | 1 |  |
| ENSBTAG00000003025 | 22 | 51564695 | 51557935 | 1 | NME6 |
| ENSBTAG00000003022 | 22 | 51576697 | 51574749 | -1 | CAMP |
| ENSBTAG00000013356 | 22 | 51621314 | 51579579 | -1 | CATHL3 |
| ENSBTAG00000013356 | 22 | 51621314 | 51579579 | -1 | CATHL3 |
| ENSBTAG00000013356 | 22 | 51621314 | 51579579 | -1 | CATHL3 |
| ENSBTAG00000013356 | 22 | 51621314 | 51579579 | -1 | CATHL3 |
| ENSBTAG00000052903 | 22 | 51587500 | 51586120 | -1 |  |
| ENSBTAG00000053016 | 22 | 51594193 | 51592817 | -1 | CATHL4 |
| ENSBTAG00000039879 | 22 | 51608515 | 51607354 | -1 |  |
| ENSBTAG00000016153 | 22 | 51632260 | 51631052 | -1 |  |
| ENSBTAG00000024852 | 22 | 51642757 | 51641063 | -1 | CATHL2 |
| ENSBTAG00000031594 | 22 | 51655076 | 51653980 | -1 |  |
| ENSBTAG00000049629 | 22 | 51665452 | 51662801 | -1 | CATHL6 |
| ENSBTAG00000020076 | 22 | 51673833 | 51671499 | -1 | CATHL5 |
| ENSBTAG00000009586 | 22 | 51717133 | 51693533 | 1 | CDC25A |
| ENSBTAG00000044607 | 22 | 51701030 | 51700922 | 1 | 5S_rRNA |
| ENSBTAG00000054831 | 22 | 51724720 | 51723115 | 1 |  |
| ENSBTAG00000015839 | 22 | 51907742 | 51814681 | 1 | MAP4 |
| ENSBTAG00000015833 | 22 | 51929087 | 51910393 | -1 | DHX30 |
| ENSBTAG00000052616 | 22 | 51930266 | 51930143 | -1 |  |
| ENSBTAG00000031567 | 22 | 52083698 | 51958135 | 1 | SMARCC1 |
| ENSBTAG00000002309 | 22 | 52102048 | 52089753 | 1 | CSPG5 |
| ENSBTAG00000011478 | 22 | 52149780 | 52133185 | 1 | ELP6 |
| ENSBTAG00000015782 | 22 | 52264116 | 52163455 | 1 | SCAP |
| ENSBTAG00000042921 | 22 | 52202301 | 52202199 | 1 | U6 |
| ENSBTAG00000051878 | 22 | 52252575 | 52252195 | -1 |  |
| ENSBTAG00000002774 | 22 | 52284441 | 52263501 | -1 | PTPN23 |
| ENSBTAG00000031519 | 22 | 52301963 | 52297736 | 1 | NGP |
| ENSBTAG00000055088 | 22 | 52321362 | 52298693 | -1 |  |
| ENSBTAG00000006573 | 22 | 52361884 | 52310680 | -1 | KLHL18 |
| ENSBTAG00000049258 | 23 | 10882587 | 10881334 | 1 |  |
| ENSBTAG00000014172 | 23 | 10942796 | 10916507 | 1 | FGD2 |
| ENSBTAG00000048345 | 23 | 31542624 | 31538865 | -1 |  |
| ENSBTAG00000053665 | 23 | 31542108 | 31542058 | -1 |  |
| ENSBTAG00000031747 | 23 | 31573268 | 31565290 | -1 | HMGN4 |
| ENSBTAG00000031753 | 23 | 31591478 | 31585191 | -1 | BTN1A1 |
| ENSBTAG00000048838 | 23 | 11011176 | 10963673 | -1 |  |
| ENSBTAG00000051300 | 23 | 10995572 | 10989275 | 1 |  |
| ENSBTAG00000000396 | 23 | 11071948 | 11066555 | 1 | PIM1 |
| ENSBTAG00000045936 | 23 | 11109870 | 11108189 | -1 |  |
| ENSBTAG00000002369 | 23 | 11148488 | 11125128 | -1 | TMEM217 |
| ENSBTAG00000014253 | 23 | 11223026 | 11148615 | 1 | TBC1D22B |
| ENSBTAG00000010852 | 23 | 31615067 | 31608469 | -1 |  |
| ENSBTAG00000054314 | 23 | 31655614 | 31644576 | -1 |  |
| ENSBTAG00000049949 | 23 | 31708940 | 31696654 | 1 | H4C8 |
| ENSBTAG00000050080 | 23 | 31725473 | 31700317 | -1 |  |
| ENSBTAG00000052895 | 23 | 31702220 | 31701454 | 1 | H2AC10 |
| ENSBTAG00000048666 | 23 | 31714207 | 31713797 | 1 | H3C7 |
| ENSBTAG00000008321 | 23 | 11282020 | 11244735 | 1 | RNF8 |
| ENSBTAG00000049555 | 23 | 31736801 | 31719142 | 1 | H4C7 |
| ENSBTAG00000048478 | 23 | 31737657 | 31737247 | -1 | H3C6 |
| ENSBTAG00000017091 | 23 | 11372125 | 11318736 | 1 | CMTR1 |
| ENSBTAG00000031767 | 23 | 31757391 | 31756982 | -1 | H3C5P |
| ENSBTAG00000048743 | 23 | 31758683 | 31758291 | -1 | H2AC8 |
| ENSBTAG00000031769 | 23 | 31759531 | 31758964 | 1 | H2BC8 |
| ENSBTAG00000049598 | 23 | 31766025 | 31765645 | -1 | H2BC7 |
| ENSBTAG00000053792 | 23 | 31766740 | 31766348 | 1 | H2AC7 |
| ENSBTAG00000031774 | 23 | 31771607 | 31770009 | -1 | H1-12 |
| ENSBTAG00000050102 | 23 | 31770707 | 31770297 | 1 | H3C4 |
| ENSBTAG00000040277 | 23 | 31775288 | 31774497 | 1 | H4C4 |
| ENSBTAG00000031776 | 23 | 31779815 | 31779435 | -1 | H2BC6 |
| ENSBTAG00000031778 | 23 | 31795097 | 31794124 | -1 | H2BC5 |
| ENSBTAG00000047206 | 23 | 31796757 | 31796098 | -1 | H1-4 |
| ENSBTAG00000039657 | 23 | 31828988 | 31818805 | -1 | H2AC6 |
| ENSBTAG00000050874 | 23 | 11356309 | 11354870 | -1 |  |
| ENSBTAG00000012069 | 23 | 11389074 | 11373453 | -1 | CCDC167 |
| ENSBTAG00000053033 | 23 | 31829004 | 31828921 | -1 | bta-mir-6531 |
| ENSBTAG00000052382 | 23 | 31829648 | 31829268 | 1 | H2BC4 |
| ENSBTAG00000005888 | 23 | 11611239 | 11546127 | -1 | MDGA1 |
| ENSBTAG00000011671 | 23 | 31849982 | 31844641 | 1 | H1-6 |
| ENSBTAG00000052609 | 23 | 31847554 | 31847243 | -1 | H4C3 |
| ENSBTAG00000000527 | 23 | 12057180 | 11729171 | 1 | ZFAND3 |
| ENSBTAG00000013065 | 23 | 31864562 | 31855234 | -1 | HFE |
| ENSBTAG00000011677 | 23 | 31878150 | 31876303 | 1 | H1-2 |
| ENSBTAG00000050477 | 23 | 31883136 | 31882726 | -1 | H3C3 |
| ENSBTAG00000007286 | 23 | 31884488 | 31884099 | -1 | H2AC5P |
| ENSBTAG00000048567 | 23 | 31885225 | 31884845 | 1 | H2BC3 |
| ENSBTAG00000050614 | 23 | 31891734 | 31891342 | 1 | H2AC4 |
| ENSBTAG00000000529 | 23 | 12436221 | 12062334 | -1 | BTBD9 |
| ENSBTAG00000050209 | 23 | 31893082 | 31892672 | 1 | H3C2 |
| ENSBTAG00000054933 | 23 | 31915463 | 31904227 | 1 | H4C2 |
| ENSBTAG00000053034 | 23 | 31908601 | 31908290 | -1 | H4C1 |
| ENSBTAG00000054582 | 23 | 31909768 | 31909358 | -1 | H3C1 |
| ENSBTAG00000012703 | 23 | 12519916 | 12494154 | -1 | GLO1 |
| ENSBTAG00000050149 | 23 | 31925267 | 31920734 | -1 |  |
| ENSBTAG00000050998 | 23 | 31927357 | 31926269 | 1 |  |
| ENSBTAG00000018523 | 23 | 31947272 | 31932362 | -1 | TRIM38 |
| ENSBTAG00000050414 | 23 | 31950693 | 31949508 | 1 |  |
| ENSBTAG00000002642 | 23 | 31977850 | 31959620 | 1 | SLC17A2 |
| ENSBTAG00000014063 | 23 | 12888737 | 12560570 | 1 | DNAH8 |
| ENSBTAG00000003797 | 23 | 32017253 | 31999597 | 1 | SLC17A3 |
| ENSBTAG00000037917 | 23 | 32068359 | 32034987 | 1 | SLC17A1 |
| ENSBTAG00000037738 | 23 | 32103739 | 32080668 | -1 | SLC17A4 |
| ENSBTAG00000052046 | 23 | 32153222 | 32153019 | 1 | H2BC2P |
| ENSBTAG00000050285 | 23 | 32156183 | 32155980 | -1 | H2BC1 |
| ENSBTAG00000046992 | 23 | 32157131 | 32156742 | 1 | H2AC1 |
| ENSBTAG00000019926 | 23 | 32242064 | 32189851 | -1 | SCGN |
| ENSBTAG00000016549 | 23 | 32573502 | 32267215 | -1 | CARMIL1 |
| ENSBTAG00000008491 | 23 | 32622371 | 32615138 | 1 |  |
| ENSBTAG00000003892 | 23 | 32731972 | 32672258 | 1 |  |
| ENSBTAG00000018165 | 23 | 32996550 | 32786389 | 1 | RIPOR2 |
| ENSBTAG00000054806 | 23 | 32933715 | 32933609 | 1 | U6 |
| ENSBTAG00000018224 | 23 | 12946096 | 12914475 | 1 | GLP1R |
| ENSBTAG00000001074 | 23 | 12976488 | 12969790 | -1 | SAYSD1 |
| ENSBTAG00000011112 | 23 | 13101280 | 13060754 | -1 | KCNK5 |
| ENSBTAG00000044708 | 23 | 13087888 | 13087820 | -1 | bta-mir-2377 |
| ENSBTAG00000010328 | 23 | 13179213 | 13166550 | -1 | KCNK17 |
| ENSBTAG00000010352 | 23 | 13187815 | 13180877 | -1 | KCNK16 |
| ENSBTAG00000027197 | 23 | 13617580 | 13196359 | -1 | KIF6 |
| ENSBTAG00000021381 | 23 | 13832799 | 13699642 | 1 | DAAM2 |
| ENSBTAG00000010449 | 23 | 13889103 | 13836254 | -1 | MOCS1 |
| ENSBTAG00000043518 | 23 | 13904665 | 13904558 | -1 | U6 |
| ENSBTAG00000048842 | 23 | 14288368 | 14286405 | 1 |  |
| ENSBTAG00000017971 | 23 | 14502729 | 14301055 | -1 | LRFN2 |
| ENSBTAG00000054029 | 23 | 14318219 | 14318113 | 1 | U6 |
| ENSBTAG00000002919 | 23 | 14963734 | 14951493 | -1 | UNC5CL |
| ENSBTAG00000005974 | 23 | 14990620 | 14967227 | 1 | APOBEC2 |
| ENSBTAG00000005975 | 23 | 14998169 | 14992681 | -1 | OARD1 |
| ENSBTAG00000009905 | 23 | 15022870 | 14998801 | 1 | NFYA |
| ENSBTAG00000021359 | 23 | 15039091 | 15025399 | 1 |  |
| ENSBTAG00000006485 | 23 | 15078758 | 15074058 | -1 | TREML1 |
| ENSBTAG00000007275 | 23 | 15105534 | 15082501 | -1 | TREM2 |
| ENSBTAG00000015707 | 23 | 15125755 | 15118374 | -1 | TREML2 |
| ENSBTAG00000017593 | 23 | 15155609 | 15140484 | -1 | TREM1 |
| ENSBTAG00000050887 | 23 | 15222948 | 15218055 | -1 |  |
| ENSBTAG00000014211 | 23 | 15417429 | 15366276 | 1 | FOXP4 |
| ENSBTAG00000019343 | 23 | 15474705 | 15457459 | 1 | MDFI |
| ENSBTAG00000012384 | 23 | 15547643 | 15499140 | -1 | TFEB |
| ENSBTAG00000003818 | 23 | 15559909 | 15548833 | -1 | PGC |
| ENSBTAG00000009861 | 23 | 15599481 | 15583411 | -1 | FRS3 |
| ENSBTAG00000038916 | 23 | 15600022 | 15593409 | 1 | PRICKLE4 |
| ENSBTAG00000008977 | 23 | 15601893 | 15600547 | 1 | TOMM6 |
| ENSBTAG00000008981 | 23 | 15669922 | 15607998 | -1 | USP49 |
| ENSBTAG00000010100 | 23 | 15687902 | 15642765 | -1 | MED20 |
| ENSBTAG00000010101 | 23 | 15698295 | 15687963 | 1 | BYSL |
| ENSBTAG00000010106 | 23 | 15705476 | 15698661 | -1 | CCND3 |
| ENSBTAG00000011339 | 23 | 15814397 | 15794648 | 1 | TAF8 |
| ENSBTAG00000054479 | 23 | 15820718 | 15817994 | -1 |  |
| ENSBTAG00000051116 | 23 | 15865906 | 15831094 | -1 | C23H6orf132 |
| ENSBTAG00000012822 | 23 | 15884246 | 15865137 | 1 | MGC137036 |
| ENSBTAG00000051956 | 23 | 15899850 | 15892199 | 1 | GUCA1A |
| ENSBTAG00000012439 | 23 | 15911894 | 15900394 | -1 | GUCA1B |
| ENSBTAG00000015301 | 23 | 15932613 | 15920024 | -1 | MRPS10 |
| ENSBTAG00000053124 | 23 | 15931149 | 15929494 | 1 |  |
| ENSBTAG00000015321 | 23 | 16145761 | 15939656 | -1 | TRERF1 |
| ENSBTAG00000043404 | 23 | 16186251 | 16186145 | 1 | U6 |
| ENSBTAG00000005967 | 23 | 16363361 | 16255756 | 1 | UBR2 |
| ENSBTAG00000005971 | 23 | 16382111 | 16367966 | -1 | PRPH2 |
| ENSBTAG00000029173 | 23 | 16377963 | 16377849 | 1 | 5S_rRNA |
| ENSBTAG00000000940 | 23 | 16399349 | 16396728 | -1 | TBCC |
| ENSBTAG00000014814 | 23 | 16506078 | 16428773 | 1 | BICRAL |
| ENSBTAG00000018478 | 23 | 16519583 | 16512932 | 1 | RPL7L1 |
| ENSBTAG00000052790 | 23 | 16534799 | 16531639 | -1 |  |
| ENSBTAG00000017097 | 23 | 16552636 | 16547283 | 1 | PTCRA |
| ENSBTAG00000015900 | 23 | 16563856 | 16555474 | 1 | CNPY3 |
| ENSBTAG00000051534 | 23 | 16575153 | 16571910 | 1 | GNMT |
| ENSBTAG00000051935 | 23 | 16598738 | 16594837 | 1 |  |
| ENSBTAG00000051409 | 23 | 16610447 | 16607203 | 1 |  |
| ENSBTAG00000005532 | 23 | 16625004 | 16610717 | -1 | PEX6 |
| ENSBTAG00000005533 | 23 | 16653065 | 16629759 | 1 | PPP2R5D |
| ENSBTAG00000005536 | 23 | 16657240 | 16652951 | -1 | MEA1 |
| ENSBTAG00000050696 | 23 | 16661311 | 16654986 | 1 | KLHDC3 |
| ENSBTAG00000053873 | 23 | 16665996 | 16661624 | 1 | RRP36 |
| ENSBTAG00000012749 | 23 | 16684135 | 16668580 | -1 | CUL7 |
| ENSBTAG00000012752 | 23 | 16688088 | 16683878 | -1 | MRPL2 |
| ENSBTAG00000012755 | 23 | 16702483 | 16689176 | 1 | KLC4 |
| ENSBTAG00000012761 | 23 | 16767981 | 16703732 | 1 | PTK7 |
| ENSBTAG00000012777 | 23 | 16783881 | 16775130 | 1 | SRF |
| ENSBTAG00000019908 | 23 | 16822127 | 16786304 | 1 | CUL9 |
| ENSBTAG00000019913 | 23 | 16824968 | 16822621 | -1 | DNPH1 |
| ENSBTAG00000046107 | 23 | 16888037 | 16856085 | 1 | TTBK1 |
| ENSBTAG00000006350 | 23 | 16899654 | 16893160 | 1 | SLC22A7 |
| ENSBTAG00000006357 | 23 | 16902449 | 16899607 | -1 | CRIP3 |
| ENSBTAG00000051172 | 23 | 16903613 | 16903507 | -1 | U6 |
| ENSBTAG00000010217 | 23 | 16955126 | 16929147 | -1 | ZNF318 |
| ENSBTAG00000005842 | 23 | 17008684 | 16988512 | 1 | ABCC10 |
| ENSBTAG00000005850 | 23 | 17013149 | 17008895 | -1 | DLK2 |
| ENSBTAG00000011189 | 23 | 17052411 | 17028383 | 1 | TJAP1 |
| ENSBTAG00000025554 | 23 | 17055851 | 17053108 | -1 | LRRC73 |
| ENSBTAG00000018967 | 23 | 17062278 | 17057485 | -1 | YIPF3 |
| ENSBTAG00000018969 | 23 | 17069399 | 17062532 | 1 | POLR1C |
| ENSBTAG00000018970 | 23 | 17118839 | 17071458 | -1 | XPO5 |
| ENSBTAG00000006015 | 23 | 17143658 | 17114387 | 1 | POLH |
| ENSBTAG00000006016 | 23 | 17153545 | 17144373 | -1 | GTPBP2 |
| ENSBTAG00000009548 | 23 | 17162867 | 17158204 | 1 | MAD2L1BP |
| ENSBTAG00000009549 | 23 | 17177978 | 17164825 | 1 | RSPH9 |
| ENSBTAG00000025571 | 23 | 17194684 | 17178373 | -1 | MRPS18A |
| ENSBTAG00000050989 | 23 | 17277526 | 17250818 | 1 |  |
| ENSBTAG00000005339 | 23 | 17297193 | 17281915 | 1 | VEGFA |
| ENSBTAG00000049965 | 23 | 17546983 | 17535072 | 1 |  |
| ENSBTAG00000047995 | 23 | 17551186 | 17551082 | -1 | U6 |
| ENSBTAG00000050249 | 23 | 17658450 | 17639869 | -1 |  |
| ENSBTAG00000032680 | 23 | 17683153 | 17658530 | 1 | TMEM63B |
| ENSBTAG00000021066 | 23 | 17695277 | 17689202 | 1 | CAPN11 |
| ENSBTAG00000050117 | 23 | 17692329 | 17692108 | -1 |  |
| ENSBTAG00000053551 | 23 | 17702528 | 17698748 | 1 |  |
| ENSBTAG00000049369 | 23 | 17727024 | 17726770 | 1 | MYMX |
| ENSBTAG00000053897 | 23 | 17729678 | 17727915 | 1 |  |
| ENSBTAG00000015131 | 23 | 17741105 | 17730856 | 1 | SLC29A1 |
| ENSBTAG00000000778 | 23 | 17758156 | 17752495 | 1 | HSP90AB1 |
| ENSBTAG00000008076 | 23 | 17763259 | 17759581 | -1 | SLC35B2 |
| ENSBTAG00000013705 | 23 | 17771918 | 17764635 | -1 | NFKBIE |
| ENSBTAG00000050799 | 23 | 17783660 | 17776596 | 1 | TMEM151B |
| ENSBTAG00000050049 | 23 | 17788901 | 17784315 | -1 |  |
| ENSBTAG00000013722 | 23 | 17813188 | 17801845 | -1 | AARS2 |
| ENSBTAG00000053813 | 23 | 17863033 | 17838739 | 1 | SPATS1 |
| ENSBTAG00000020012 | 23 | 17907737 | 17866548 | 1 | CDC5L |
| ENSBTAG00000032887 | 23 | 18641106 | 18239634 | -1 | SUPT3H |
| ENSBTAG00000045355 | 23 | 18350650 | 18350519 | 1 | 5S_rRNA |
| ENSBTAG00000004104 | 23 | 18945527 | 18594098 | 1 | RUNX2 |
| ENSBTAG00000010244 | 23 | 19351221 | 19185150 | -1 | CLIC5 |
| ENSBTAG00000044482 | 23 | 19298641 | 19298535 | 1 | U6 |
| ENSBTAG00000003499 | 23 | 19401592 | 19386682 | 1 | ENPP4 |
| ENSBTAG00000015829 | 23 | 19415720 | 19405349 | -1 | ENPP5 |
| ENSBTAG00000009302 | 23 | 19685336 | 19452882 | -1 | RCAN2 |
| ENSBTAG00000003632 | 23 | 19878413 | 19743358 | -1 | CYP39A1 |
| ENSBTAG00000052219 | 23 | 19841295 | 19841189 | 1 | U6 |
| ENSBTAG00000016302 | 23 | 19900772 | 19878544 | 1 | SLC25A27 |
| ENSBTAG00000009978 | 23 | 19922053 | 19910664 | 1 | TDRD6 |
| ENSBTAG00000019315 | 23 | 19963342 | 19924777 | -1 | PLA2G7 |
| ENSBTAG00000047009 | 23 | 19984806 | 19970227 | 1 | ANKRD66 |
| ENSBTAG00000004345 | 23 | 20049421 | 20023407 | 1 | MEP1A |
| ENSBTAG00000004347 | 23 | 20136992 | 20061552 | -1 | ADGRF5 |
| ENSBTAG00000021609 | 23 | 20258614 | 20213988 | -1 | ADGRF1 |
| ENSBTAG00000020054 | 23 | 20482057 | 20418222 | -1 | TNFRSF21 |
| ENSBTAG00000000322 | 23 | 20689496 | 20602899 | 1 | CD2AP |
| ENSBTAG00000037687 | 23 | 20740185 | 20706772 | 1 | ADGRF2 |
| ENSBTAG00000002934 | 23 | 20764795 | 20752000 | 1 | ADGRF4 |
| ENSBTAG00000016499 | 23 | 20839674 | 20807288 | 1 | OPN5 |
| ENSBTAG00000021415 | 23 | 20900663 | 20899056 | -1 |  |
| ENSBTAG00000051749 | 23 | 21093942 | 21093163 | -1 | PTCHD4 |
| ENSBTAG00000053275 | 23 | 21610363 | 21609428 | -1 | OR9G1 |
| ENSBTAG00000047485 | 23 | 21701280 | 21697125 | 1 | OR5M10C |
| ENSBTAG00000038677 | 23 | 21745130 | 21739991 | 1 |  |
| ENSBTAG00000051050 | 23 | 21752076 | 21751958 | -1 | 5S_rRNA |
| ENSBTAG00000054235 | 23 | 21777951 | 21776616 | 1 |  |
| ENSBTAG00000046237 | 23 | 22123107 | 22122697 | 1 |  |
| ENSBTAG00000014272 | 23 | 22263733 | 22196643 | -1 | MMUT |
| ENSBTAG00000020710 | 23 | 22280742 | 22263107 | 1 | CENPQ |
| ENSBTAG00000025535 | 23 | 22307981 | 22288147 | 1 | GLYATL3 |
| ENSBTAG00000047591 | 23 | 22327665 | 22323297 | 1 | C23H6orf141 |
| ENSBTAG00000045363 | 23 | 22367810 | 22367716 | -1 | U6 |
| ENSBTAG00000011300 | 23 | 22392473 | 22371557 | -1 | RHAG |
| ENSBTAG00000002166 | 23 | 22439206 | 22413744 | -1 | CRISP2 |
| ENSBTAG00000012918 | 23 | 22484653 | 22446157 | -1 | CRISP3 |
| ENSBTAG00000053808 | 23 | 22458975 | 22457553 | -1 |  |
| ENSBTAG00000042716 | 23 | 22474283 | 22473968 | -1 | 7SK |
| ENSBTAG00000050850 | 23 | 22504879 | 22503240 | -1 |  |
| ENSBTAG00000008085 | 23 | 22544257 | 22527786 | -1 | CRISP1 |
| ENSBTAG00000054219 | 23 | 22615351 | 22612852 | -1 | DEFB114 |
| ENSBTAG00000054396 | 23 | 22634981 | 22633444 | -1 | DEFB113 |
| ENSBTAG00000051453 | 23 | 22656537 | 22651567 | -1 | DEFB110 |
| ENSBTAG00000046711 | 23 | 22669740 | 22663626 | -1 |  |
| ENSBTAG00000020425 | 23 | 23337345 | 23277603 | 1 | TFAP2D |
| ENSBTAG00000020426 | 23 | 23406742 | 23375185 | 1 | TFAP2B |
| ENSBTAG00000053324 | 23 | 23464859 | 23460005 | 1 |  |
| ENSBTAG00000011237 | 23 | 24509308 | 24075070 | -1 | PKHD1 |
| ENSBTAG00000050626 | 23 | 24573333 | 24559298 | 1 |  |
| ENSBTAG00000036400 | 23 | 24567289 | 24567204 | 1 | bta-mir-206 |
| ENSBTAG00000029826 | 23 | 24571404 | 24571300 | 1 | MIR133B |
| ENSBTAG00000002150 | 23 | 24609483 | 24605962 | 1 | IL17A |
| ENSBTAG00000016835 | 23 | 24653192 | 24645643 | -1 | IL17F |
| ENSBTAG00000010721 | 23 | 24712140 | 24694544 | -1 | MCM3 |
| ENSBTAG00000025494 | 23 | 24826623 | 24783271 | 1 | PAQR8 |
| ENSBTAG00000017810 | 23 | 24883070 | 24842515 | 1 | EFHC1 |
| ENSBTAG00000009112 | 23 | 24957939 | 24893081 | -1 | TRAM2 |
| ENSBTAG00000055192 | 23 | 24900614 | 24896961 | -1 |  |
| ENSBTAG00000005206 | 23 | 25066519 | 25056028 | 1 | TMEM14A |
| ENSBTAG00000006546 | 23 | 25088616 | 25074938 | -1 | GSTA2 |
| ENSBTAG00000025485 | 23 | 25113151 | 25101816 | -1 |  |
| ENSBTAG00000021516 | 23 | 25137232 | 25122359 | -1 | GSTA1 |
| ENSBTAG00000037949 | 23 | 25162882 | 25141525 | -1 | GSTA5 |
| ENSBTAG00000032642 | 23 | 25204322 | 25182280 | -1 | GSTA3 |
| ENSBTAG00000004288 | 23 | 25224154 | 25209814 | -1 | GSTA4 |
| ENSBTAG00000043250 | 23 | 25224585 | 25224255 | 1 | 7SK |
| ENSBTAG00000015590 | 23 | 25272669 | 25229377 | -1 | CILK1 |
| ENSBTAG00000015083 | 23 | 25297277 | 25275775 | 1 | FBXO9 |
| ENSBTAG00000008148 | 23 | 25332932 | 25311634 | -1 | GCM1 |
| ENSBTAG00000048357 | 23 | 25325947 | 25325829 | -1 | 5S_rRNA |
| ENSBTAG00000003359 | 23 | 25474020 | 25400733 | -1 | ELOVL5 |
| ENSBTAG00000045034 | 23 | 25485519 | 25485248 | 1 |  |
| ENSBTAG00000009656 | 23 | 25589209 | 25583083 | -1 |  |
| ENSBTAG00000021077 | 23 | 25622150 | 25607502 | 1 | BOLA-DQB |
| ENSBTAG00000038128 | 23 | 25643878 | 25636255 | -1 | BOLA-DQA5 |
| ENSBTAG00000019588 | 23 | 25680506 | 25672686 | 1 | BLA-DQB |
| ENSBTAG00000019588 | 23 | 25680506 | 25672686 | 1 | BLA-DQB |
| ENSBTAG00000019588 | 23 | 25680506 | 25672686 | 1 | BLA-DQB |
| ENSBTAG00000019588 | 23 | 25680506 | 25672686 | 1 | BLA-DQB |
| ENSBTAG00000019588 | 23 | 25680506 | 25672686 | 1 | BLA-DQB |
| ENSBTAG00000037605 | 23 | 25695296 | 25691259 | -1 |  |
| ENSBTAG00000013919 | 23 | 25735337 | 25723715 | 1 |  |
| ENSBTAG00000048364 | 23 | 25781520 | 25770710 | 1 |  |
| ENSBTAG00000038397 | 23 | 25809471 | 25795675 | 1 |  |
| ENSBTAG00000015565 | 23 | 25828791 | 25825528 | 1 |  |
| ENSBTAG00000010645 | 23 | 25843155 | 25837353 | -1 | BOLA-DRA |
| ENSBTAG00000034939 | 23 | 25883331 | 25858687 | 1 | BTNL2 |
| ENSBTAG00000034945 | 23 | 25894260 | 25885521 | -1 |  |
| ENSBTAG00000007618 | 23 | 25907363 | 25899578 | 1 |  |
| ENSBTAG00000026163 | 23 | 26113857 | 25969346 | 1 |  |
| ENSBTAG00000050817 | 23 | 26168271 | 26167597 | 1 |  |
| ENSBTAG00000023541 | 23 | 26992543 | 26990784 | 1 |  |
| ENSBTAG00000048304 | 23 | 27125633 | 27112294 | -1 |  |
| ENSBTAG00000023563 | 23 | 27146479 | 27139269 | -1 |  |
| ENSBTAG00000025424 | 23 | 27196528 | 27171388 | 1 | NOTCH4 |
| ENSBTAG00000054438 | 23 | 27199347 | 27197567 | 1 | GPSM3 |
| ENSBTAG00000014421 | 23 | 27203174 | 27199606 | 1 | PBX2 |
| ENSBTAG00000014420 | 23 | 27209354 | 27206155 | 1 | AGER |
| ENSBTAG00000025413 | 23 | 27212136 | 27209390 | -1 | RNF5 |
| ENSBTAG00000004442 | 23 | 27221612 | 27214132 | 1 | AGPAT1 |
| ENSBTAG00000025410 | 23 | 27224272 | 27221530 | -1 | EGFL8 |
| ENSBTAG00000004436 | 23 | 27233488 | 27225234 | -1 | PPT2 |
| ENSBTAG00000038904 | 23 | 27238717 | 27235239 | 1 | PRRT1 |
| ENSBTAG00000037781 | 23 | 27254424 | 27252845 | 1 | FKBPL |
| ENSBTAG00000008794 | 23 | 27264842 | 27254964 | 1 | ATF6B |
| ENSBTAG00000001444 | 23 | 27326827 | 27279346 | 1 | TNXB |
| ENSBTAG00000047039 | 23 | 27330230 | 27326531 | -1 | CYP21 |
| ENSBTAG00000006864 | 23 | 27348134 | 27333830 | -1 |  |
| ENSBTAG00000052577 | 23 | 27365735 | 27352864 | 1 |  |
| ENSBTAG00000001443 | 23 | 27368844 | 27365917 | -1 |  |
| ENSBTAG00000037533 | 23 | 27386883 | 27372094 | -1 | C4A |
| ENSBTAG00000005589 | 23 | 27396606 | 27387692 | -1 | STK19 |
| ENSBTAG00000005588 | 23 | 27402108 | 27396902 | 1 | DXO |
| ENSBTAG00000005587 | 23 | 27409242 | 27399292 | -1 | SKIV2L |
| ENSBTAG00000007453 | 23 | 27415203 | 27409462 | 1 | NELFE |
| ENSBTAG00000046158 | 23 | 27421377 | 27415355 | -1 | CFB |
| ENSBTAG00000007450 | 23 | 27433479 | 27421810 | -1 | C2 |
| ENSBTAG00000032031 | 23 | 27465020 | 27463641 | 1 | ZBTB12 |
| ENSBTAG00000005676 | 23 | 27480388 | 27467178 | 1 | EHMT2 |
| ENSBTAG00000005675 | 23 | 27494225 | 27480743 | 1 | SLC44A4 |
| ENSBTAG00000005674 | 23 | 27498491 | 27494530 | 1 | NEU1 |
| ENSBTAG00000053399 | 23 | 27514505 | 27507452 | 1 |  |
| ENSBTAG00000042989 | 23 | 27514184 | 27514118 | -1 | SNORD52 |
| ENSBTAG00000043151 | 23 | 27514688 | 27514626 | -1 | SNORD48 |
| ENSBTAG00000025441 | 23 | 27522790 | 27520317 | -1 | HSPA1A |
| ENSBTAG00000025441 | 23 | 27522790 | 27520317 | -1 | HSPA1A |
| ENSBTAG00000025441 | 23 | 27522790 | 27520317 | -1 | HSPA1A |
| ENSBTAG00000025441 | 23 | 27522790 | 27520317 | -1 | HSPA1A |
| ENSBTAG00000025441 | 23 | 27522790 | 27520317 | -1 | HSPA1A |
| ENSBTAG00000025441 | 23 | 27522790 | 27520317 | -1 | HSPA1A |
| ENSBTAG00000025442 | 23 | 27527209 | 27523225 | 1 | HSPA1L |
| ENSBTAG00000005634 | 23 | 27533357 | 27528402 | 1 | LSM2 |
| ENSBTAG00000005631 | 23 | 27546157 | 27533912 | 1 | VARS1 |
| ENSBTAG00000005630 | 23 | 27555831 | 27546267 | 1 | VWA7 |
| ENSBTAG00000039620 | 23 | 27558048 | 27555744 | -1 | SAPCD1 |
| ENSBTAG00000019790 | 23 | 27578112 | 27558157 | -1 | MSH5 |
| ENSBTAG00000013533 | 23 | 27581255 | 27575514 | 1 | CLIC1 |
| ENSBTAG00000013530 | 23 | 27585128 | 27581609 | 1 | DDAH2 |
| ENSBTAG00000013525 | 23 | 27588663 | 27585431 | -1 | MPIG6B |
| ENSBTAG00000000585 | 23 | 27592606 | 27589914 | 1 | LY6G6C |
| ENSBTAG00000053561 | 23 | 27595228 | 27592579 | -1 | LY6G6D |
| ENSBTAG00000000582 | 23 | 27598654 | 27595986 | 1 | LY6G6E |
| ENSBTAG00000000580 | 23 | 27602913 | 27599221 | -1 | LY6G6F |
| ENSBTAG00000000580 | 23 | 27602913 | 27599221 | -1 | LY6G6F |
| ENSBTAG00000000578 | 23 | 27618039 | 27599348 | 1 | ABHD16A |
| ENSBTAG00000025449 | 23 | 27625133 | 27622767 | 1 | LY6G5C |
| ENSBTAG00000039740 | 23 | 27628964 | 27627266 | -1 | LY6G5B |
| ENSBTAG00000008837 | 23 | 27633927 | 27629798 | -1 | CSNK2B |
| ENSBTAG00000008835 | 23 | 27637429 | 27634037 | 1 | GPANK1 |
| ENSBTAG00000023628 | 23 | 27641450 | 27639809 | 1 | C23H6orf47 |
| ENSBTAG00000008833 | 23 | 27643685 | 27641580 | -1 | APOM |
| ENSBTAG00000019685 | 23 | 27656958 | 27645741 | 1 | BAG6 |
| ENSBTAG00000019682 | 23 | 27671044 | 27657894 | -1 | PRRC2A |
| ENSBTAG00000042984 | 23 | 27670683 | 27670553 | -1 | SNORA38 |
| ENSBTAG00000020554 | 23 | 27687723 | 27685449 | -1 | AIF1 |
| ENSBTAG00000018343 | 23 | 27706071 | 27703728 | 1 | NCR3 |
| ENSBTAG00000053179 | 23 | 27707827 | 27706740 | -1 | LST1 |
| ENSBTAG00000020674 | 23 | 27713962 | 27712125 | 1 | LTB |
| ENSBTAG00000025471 | 23 | 27719047 | 27716168 | -1 | TNF |
| ENSBTAG00000000016 | 23 | 27721739 | 27720176 | -1 | LTA |
| ENSBTAG00000014492 | 23 | 27742916 | 27732075 | -1 | NFKBIL1 |
| ENSBTAG00000014491 | 23 | 27746134 | 27743849 | 1 | ATP6V1G2 |
| ENSBTAG00000014490 | 23 | 27761676 | 27748035 | 1 | DDX39B |
| ENSBTAG00000045577 | 23 | 27760332 | 27756575 | -1 | MCCD1 |
| ENSBTAG00000031913 | 23 | 27797556 | 27796195 | 1 |  |
| ENSBTAG00000031905 | 23 | 27829325 | 27828888 | 1 |  |
| ENSBTAG00000005182 | 23 | 27836725 | 27829737 | 1 | BoLA |
| ENSBTAG00000005182 | 23 | 27836725 | 27829737 | 1 | BoLA |
| ENSBTAG00000054754 | 23 | 27830699 | 27830623 | 1 | bta-mir-10167 |
| ENSBTAG00000054342 | 23 | 27913198 | 27841095 | -1 | MIC1 |
| ENSBTAG00000054342 | 23 | 27913198 | 27841095 | -1 | MIC1 |
| ENSBTAG00000010166 | 23 | 27849003 | 27842117 | -1 | MIC2 |
| ENSBTAG00000012208 | 23 | 27875056 | 27871206 | 1 |  |
| ENSBTAG00000051047 | 23 | 27944257 | 27894420 | -1 |  |
| ENSBTAG00000001476 | 23 | 27905422 | 27896902 | 1 |  |
| ENSBTAG00000007075 | 23 | 28059869 | 27943564 | 1 |  |
| ENSBTAG00000053433 | 23 | 27961282 | 27953265 | -1 |  |
| ENSBTAG00000021111 | 23 | 27987297 | 27982798 | 1 | POU5F1 |
| ENSBTAG00000021111 | 23 | 27987297 | 27982798 | 1 | POU5F1 |
| ENSBTAG00000021111 | 23 | 27987297 | 27982798 | 1 | POU5F1 |
| ENSBTAG00000014435 | 23 | 27992408 | 27988581 | -1 | TCF19 |
| ENSBTAG00000014434 | 23 | 28004146 | 27992754 | 1 | CCHCR1 |
| ENSBTAG00000025531 | 23 | 28007631 | 28006091 | 1 | PSORS1C2 |
| ENSBTAG00000021721 | 23 | 28025613 | 28021400 | 1 | CDSN |
| ENSBTAG00000004539 | 23 | 28030328 | 28029202 | 1 | C23H6orf15 |
| ENSBTAG00000054001 | 23 | 28050216 | 28050110 | -1 | U6 |
| ENSBTAG00000038810 | 23 | 28143467 | 28142385 | 1 | SFTA2 |
| ENSBTAG00000049699 | 23 | 28144355 | 28144249 | 1 | U6 |
| ENSBTAG00000010698 | 23 | 28159087 | 28146081 | -1 | VARS2 |
| ENSBTAG00000046757 | 23 | 28166226 | 28159277 | -1 | GTF2H4 |
| ENSBTAG00000010682 | 23 | 28219346 | 28172747 | -1 | DDR1 |
| ENSBTAG00000011358 | 23 | 28286183 | 28284912 | 1 | IER3 |
| ENSBTAG00000009960 | 23 | 28296570 | 28286486 | 1 | FLOT1 |
| ENSBTAG00000006969 | 23 | 28302018 | 28298359 | -1 | TUBB |
| ENSBTAG00000025526 | 23 | 28316822 | 28304399 | 1 | MDC1 |
| ENSBTAG00000006966 | 23 | 28323011 | 28319981 | 1 | NRM |
| ENSBTAG00000039695 | 23 | 28333411 | 28324719 | 1 | PPP1R18 |
| ENSBTAG00000006960 | 23 | 28348888 | 28336294 | 1 | DHX16 |
| ENSBTAG00000006958 | 23 | 28353973 | 28349045 | -1 | C23H6orf136 |
| ENSBTAG00000006941 | 23 | 28366874 | 28353038 | -1 | ATAT1 |
| ENSBTAG00000006936 | 23 | 28373216 | 28366299 | -1 | MRPS18B |
| ENSBTAG00000006933 | 23 | 28389370 | 28373727 | 1 | PPP1R10 |
| ENSBTAG00000006927 | 23 | 28408133 | 28394981 | -1 | ABCF1 |
| ENSBTAG00000044647 | 23 | 28399243 | 28399166 | -1 | bta-mir-2378 |
| ENSBTAG00000037337 | 23 | 28399640 | 28399539 | -1 | bta-mir-877 |
| ENSBTAG00000006914 | 23 | 28420086 | 28415008 | -1 | PRR3 |
| ENSBTAG00000025516 | 23 | 28428768 | 28420981 | 1 | GNL1 |
| ENSBTAG00000050907 | 23 | 28453766 | 28451481 | -1 | RPP21 |
| ENSBTAG00000007643 | 23 | 28465409 | 28456600 | -1 | TRIM39 |
| ENSBTAG00000001477 | 23 | 28492531 | 28492098 | -1 |  |
| ENSBTAG00000038619 | 23 | 28505219 | 28493346 | 1 |  |
| ENSBTAG00000053664 | 23 | 28524677 | 28516280 | -1 |  |
| ENSBTAG00000005146 | 23 | 28527983 | 28525598 | -1 |  |
| ENSBTAG00000019386 | 23 | 28553089 | 28548781 | -1 | BOLA-NC1 |
| ENSBTAG00000019386 | 23 | 28553089 | 28548781 | -1 | BOLA-NC1 |
| ENSBTAG00000020116 | 23 | 28667379 | 28663713 | -1 | JSP.1 |
| ENSBTAG00000037421 | 23 | 28686666 | 28677524 | -1 |  |
| ENSBTAG00000054323 | 23 | 28720305 | 28720245 | -1 | bta-mir-11979 |
| ENSBTAG00000002069 | 23 | 28724399 | 28720501 | -1 | BOLA |
| ENSBTAG00000002069 | 23 | 28724399 | 28720501 | -1 | BOLA |
| ENSBTAG00000002069 | 23 | 28724399 | 28720501 | -1 | BOLA |
| ENSBTAG00000002069 | 23 | 28724399 | 28720501 | -1 | BOLA |
| ENSBTAG00000002069 | 23 | 28724399 | 28720501 | -1 | BOLA |
| ENSBTAG00000051089 | 23 | 28750116 | 28741064 | -1 |  |
| ENSBTAG00000035744 | 23 | 28787696 | 28777770 | 1 | TRIM26 |
| ENSBTAG00000007532 | 23 | 28813526 | 28803215 | -1 | TRIM15 |
| ENSBTAG00000007530 | 23 | 28825181 | 28816417 | 1 | TRIM10 |
| ENSBTAG00000037381 | 23 | 28840652 | 28828979 | -1 | TRIM40 |
| ENSBTAG00000004490 | 23 | 28879147 | 28868629 | 1 | TRIM31 |
| ENSBTAG00000054439 | 23 | 28888228 | 28886762 | 1 |  |
| ENSBTAG00000052499 | 23 | 28908861 | 28904289 | 1 | RNF39 |
| ENSBTAG00000032247 | 23 | 28913055 | 28909903 | -1 | PPP1R11 |
| ENSBTAG00000031792 | 23 | 28920535 | 28917109 | -1 | POLR1H |
| ENSBTAG00000054419 | 23 | 28925030 | 28924955 | -1 |  |
| ENSBTAG00000054588 | 23 | 28926246 | 28925617 | 1 |  |
| ENSBTAG00000017836 | 23 | 28943319 | 28938566 | 1 | ZFP57 |
| ENSBTAG00000017818 | 23 | 28953585 | 28941968 | -1 | MOG |
| ENSBTAG00000051539 | 23 | 28967670 | 28962193 | 1 |  |
| ENSBTAG00000017239 | 23 | 29003173 | 28974598 | 1 | GABBR1 |
| ENSBTAG00000031825 | 23 | 29014232 | 29013585 | -1 |  |
| ENSBTAG00000025398 | 23 | 29074861 | 29027762 | -1 |  |
| ENSBTAG00000052703 | 23 | 29069618 | 29054653 | -1 |  |
| ENSBTAG00000003250 | 23 | 29089402 | 29088449 | -1 | OR2H1D |
| ENSBTAG00000043759 | 23 | 29092651 | 29092546 | 1 | U6 |
| ENSBTAG00000027245 | 23 | 29103164 | 29102226 | -1 | OR2H1 |
| ENSBTAG00000052587 | 23 | 29115601 | 29115535 | -1 | bta-mir-12033 |
| ENSBTAG00000027246 | 23 | 29120016 | 29117026 | 1 | UBD |
| ENSBTAG00000017227 | 23 | 29122128 | 29121175 | -1 | OR2I1P |
| ENSBTAG00000045586 | 23 | 29150230 | 29149292 | -1 | OR10C1 |
| ENSBTAG00000012956 | 23 | 29155828 | 29154869 | -1 | OR11W1 |
| ENSBTAG00000048946 | 23 | 29168701 | 29167187 | 1 | OR11A12 |
| ENSBTAG00000053227 | 23 | 29184643 | 29183684 | 1 | OR11A1 |
| ENSBTAG00000031832 | 23 | 29192289 | 29191351 | -1 | OR1O8 |
| ENSBTAG00000049031 | 23 | 29204751 | 29203825 | -1 | OR12D2G |
| ENSBTAG00000050146 | 23 | 29218926 | 29218012 | -1 |  |
| ENSBTAG00000049352 | 23 | 29226003 | 29222731 | -1 | OR1O5P |
| ENSBTAG00000053370 | 23 | 29232531 | 29231605 | -1 | OR12D2 |
| ENSBTAG00000031835 | 23 | 29238908 | 29237952 | -1 | OR1O9 |
| ENSBTAG00000055092 | 23 | 29250424 | 29248740 | -1 |  |
| ENSBTAG00000050879 | 23 | 29261963 | 29261037 | -1 | OR12D2F |
| ENSBTAG00000052867 | 23 | 29271956 | 29271030 | -1 | OR12D23 |
| ENSBTAG00000051434 | 23 | 29279049 | 29278135 | -1 | OR12D18 |
| ENSBTAG00000052416 | 23 | 29286137 | 29282850 | -1 | OR1O6P |
| ENSBTAG00000049292 | 23 | 29292713 | 29291787 | -1 | OR12D2H |
| ENSBTAG00000040188 | 23 | 29309785 | 29305933 | -1 | OR12D2E |
| ENSBTAG00000052547 | 23 | 29322505 | 29321579 | -1 | OR12D2D |
| ENSBTAG00000039116 | 23 | 29345285 | 29344338 | 1 | OR5V2 |
| ENSBTAG00000039562 | 23 | 29351712 | 29350762 | 1 | OR12D3 |
| ENSBTAG00000048441 | 23 | 29359398 | 29358442 | 1 | OR12D21 |
| ENSBTAG00000052463 | 23 | 29372087 | 29368353 | 1 | OR12D20 |
| ENSBTAG00000039901 | 23 | 29378423 | 29377500 | 1 | OR5V1C |
| ENSBTAG00000038472 | 23 | 29388677 | 29387724 | 1 | OR5V1 |
| ENSBTAG00000013654 | 23 | 29408693 | 29407731 | -1 | OR14J12 |
| ENSBTAG00000054679 | 23 | 29414404 | 29413295 | -1 | OR14J1 |
| ENSBTAG00000031843 | 23 | 29425560 | 29424616 | -1 | OR2B4 |
| ENSBTAG00000039534 | 23 | 29443488 | 29442553 | -1 | OR2G1 |
| ENSBTAG00000047086 | 23 | 29470207 | 29469275 | 1 | OR2H18 |
| ENSBTAG00000046777 | 23 | 29483772 | 29481565 | 1 | OR2B37 |
| ENSBTAG00000052969 | 23 | 29494154 | 29491367 | 1 | OR2H20 |
| ENSBTAG00000046023 | 23 | 29501680 | 29500748 | 1 | OR2H19 |
| ENSBTAG00000038608 | 23 | 29516566 | 29515616 | 1 | OR2G7B |
| ENSBTAG00000000228 | 23 | 29528871 | 29527909 | -1 | OR2Y3B |
| ENSBTAG00000037628 | 23 | 29562523 | 29561576 | 1 | OR2U5 |
| ENSBTAG00000050455 | 23 | 29569939 | 29568995 | -1 |  |
| ENSBTAG00000054760 | 23 | 29589378 | 29588437 | 1 | OR2B3 |
| ENSBTAG00000006339 | 23 | 29605106 | 29604281 | 1 |  |
| ENSBTAG00000027279 | 23 | 29613169 | 29612534 | -1 |  |
| ENSBTAG00000050351 | 23 | 29616214 | 29615075 | -1 |  |
| ENSBTAG00000049353 | 23 | 29651822 | 29650881 | 1 | OR2B3B |
| ENSBTAG00000040582 | 23 | 29663150 | 29661663 | 1 | OR2P2 |
| ENSBTAG00000053092 | 23 | 29680334 | 29679396 | 1 | OR2N1 |
| ENSBTAG00000051716 | 23 | 29703129 | 29702200 | -1 | OR2J3 |
| ENSBTAG00000051060 | 23 | 29718152 | 29717196 | 1 | OR10AL42 |
| ENSBTAG00000054689 | 23 | 29728406 | 29725370 | -1 |  |
| ENSBTAG00000052149 | 23 | 29740592 | 29739360 | -1 | OR2J3D |
| ENSBTAG00000054399 | 23 | 29752528 | 29751566 | 1 | OR10AL41 |
| ENSBTAG00000053389 | 23 | 29772080 | 29770766 | 1 | OR2N1O |
| ENSBTAG00000049302 | 23 | 29781070 | 29780120 | 1 | OR10AL43 |
| ENSBTAG00000049750 | 23 | 29792882 | 29791959 | -1 | OR2J3B |
| ENSBTAG00000051617 | 23 | 29821721 | 29820771 | 1 | OR10AL40 |
| ENSBTAG00000050941 | 23 | 29832180 | 29831244 | 1 |  |
| ENSBTAG00000051052 | 23 | 29836146 | 29835220 | -1 |  |
| ENSBTAG00000054401 | 23 | 29861913 | 29859536 | -1 | OR2J1 |
| ENSBTAG00000040457 | 23 | 29877890 | 29876937 | 1 | OR2W1D |
| ENSBTAG00000015175 | 23 | 29896566 | 29895604 | 1 | OR2W1 |
| ENSBTAG00000000214 | 23 | 29911257 | 29910286 | 1 | OR2AD1 |
| ENSBTAG00000047585 | 23 | 29931333 | 29930410 | 1 | OR2AD1B |
| ENSBTAG00000008349 | 23 | 30007987 | 29996426 | 1 | ZNF311 |
| ENSBTAG00000047789 | 23 | 30019307 | 30019010 | -1 | Metazoa_SRP |
| ENSBTAG00000052294 | 23 | 30031573 | 30026121 | -1 |  |
| ENSBTAG00000000871 | 23 | 30061046 | 30047159 | 1 | TRIM27 |
| ENSBTAG00000045477 | 23 | 30069235 | 30069139 | 1 | U6 |
| ENSBTAG00000053198 | 23 | 30103049 | 30101631 | -1 |  |
| ENSBTAG00000046381 | 23 | 30131269 | 30131163 | -1 | U6 |
| ENSBTAG00000052835 | 23 | 30132772 | 30132666 | 1 | U6 |
| ENSBTAG00000051232 | 23 | 30318807 | 30198394 | 1 |  |
| ENSBTAG00000051628 | 23 | 30214092 | 30210124 | 1 |  |
| ENSBTAG00000050752 | 23 | 30245150 | 30236196 | -1 |  |
| ENSBTAG00000049252 | 23 | 30275730 | 30269444 | 1 |  |
| ENSBTAG00000008943 | 23 | 30347409 | 30316678 | 1 | ZSCAN12 |
| ENSBTAG00000048996 | 23 | 30358682 | 30355323 | 1 |  |
| ENSBTAG00000037988 | 23 | 30379817 | 30377190 | 1 | ZSCAN31 |
| ENSBTAG00000046573 | 23 | 30412787 | 30390348 | -1 | PGBD1 |
| ENSBTAG00000045403 | 23 | 30401566 | 30401490 | -1 |  |
| ENSBTAG00000031869 | 23 | 30427406 | 30416140 | -1 | ZSCAN26 |
| ENSBTAG00000031871 | 23 | 30435000 | 30431383 | -1 | NKAPL |
| ENSBTAG00000003575 | 23 | 30450225 | 30441464 | -1 | ZKSCAN4 |
| ENSBTAG00000040168 | 23 | 30467794 | 30467132 | -1 | ZSCAN9 |
| ENSBTAG00000048989 | 23 | 30494674 | 30493485 | -1 |  |
| ENSBTAG00000051292 | 23 | 30513892 | 30510284 | -1 |  |
| ENSBTAG00000051500 | 23 | 30525304 | 30520977 | -1 | ZNF389 |
| ENSBTAG00000007074 | 23 | 30550488 | 30534992 | -1 | ZKSCAN8 |
| ENSBTAG00000031873 | 23 | 30568915 | 30561557 | -1 | ZSCAN16 |
| ENSBTAG00000049545 | 23 | 30581248 | 30575870 | -1 |  |
| ENSBTAG00000031874 | 23 | 30599254 | 30587543 | -1 | ZNF165 |
| ENSBTAG00000013384 | 23 | 30606276 | 30605335 | -1 | OR1F12 |
| ENSBTAG00000039079 | 23 | 30632830 | 30631701 | 1 |  |
| ENSBTAG00000054480 | 23 | 30653957 | 30652837 | 1 | OR2B8K |
| ENSBTAG00000055279 | 23 | 30670288 | 30669159 | 1 |  |
| ENSBTAG00000055288 | 23 | 30679780 | 30678836 | -1 | OR2B7C |
| ENSBTAG00000054581 | 23 | 30714609 | 30713671 | 1 | OR2B8M |
| ENSBTAG00000050507 | 23 | 30724682 | 30723738 | -1 | OR2B7 |
| ENSBTAG00000049012 | 23 | 30740584 | 30739646 | 1 |  |
| ENSBTAG00000054795 | 23 | 30753838 | 30752711 | 1 |  |
| ENSBTAG00000050213 | 23 | 30764233 | 30763289 | -1 | OR2B7B |
| ENSBTAG00000040307 | 23 | 30789874 | 30788954 | -1 | OR2W2 |
| ENSBTAG00000005885 | 23 | 30821817 | 30820412 | -1 | OR2B2 |
| ENSBTAG00000038597 | 23 | 30843453 | 30842512 | 1 | OR2W6 |
| ENSBTAG00000039677 | 23 | 30856456 | 30855515 | 1 | OR2B6 |
| ENSBTAG00000039145 | 23 | 30882438 | 30881509 | 1 | OR2W4 |
| ENSBTAG00000039274 | 23 | 30931686 | 30930742 | 1 | OR2B2D |
| ENSBTAG00000050532 | 23 | 30995670 | 30948090 | -1 | H2BC14 |
| ENSBTAG00000051969 | 23 | 30996569 | 30948887 | 1 | H2AC14 |
| ENSBTAG00000054340 | 23 | 30974615 | 30959667 | 1 | H4C13 |
| ENSBTAG00000031886 | 23 | 30976581 | 30972348 | -1 | H2BC15 |
| ENSBTAG00000053627 | 23 | 30992426 | 30975866 | -1 | H4C11 |
| ENSBTAG00000053621 | 23 | 30981632 | 30976809 | 1 | H2AC15 |
| ENSBTAG00000054176 | 23 | 31002313 | 30982648 | 1 | H4C12 |
| ENSBTAG00000046373 | 23 | 31002183 | 31001731 | -1 | H2AC13 |
| ENSBTAG00000052713 | 23 | 31002870 | 31002490 | 1 | H2BC13 |
| ENSBTAG00000054889 | 23 | 31035381 | 31009401 | -1 |  |
| ENSBTAG00000045181 | 23 | 31023071 | 31022994 | -1 | bta-mir-2379 |
| ENSBTAG00000054556 | 23 | 31119516 | 31119451 | 1 | bta-mir-7857-1 |
| ENSBTAG00000050370 | 23 | 31129314 | 31129228 | -1 | bta-mir-7857-2 |
| ENSBTAG00000051743 | 23 | 31166680 | 31164801 | 1 |  |
| ENSBTAG00000055174 | 23 | 31171190 | 31170000 | -1 |  |
| ENSBTAG00000011498 | 23 | 31184917 | 31172787 | 1 | ZNF184 |
| ENSBTAG00000009152 | 23 | 31208024 | 31199273 | -1 | ZNF391 |
| ENSBTAG00000024169 | 23 | 31240259 | 31236803 | 1 | POM121L2 |
| ENSBTAG00000012461 | 23 | 31315668 | 31308822 | -1 | PRSS16 |
| ENSBTAG00000054548 | 23 | 31354325 | 31352111 | -1 | H2AC12 |
| ENSBTAG00000038173 | 23 | 31355275 | 31354601 | 1 | H2BC12 |
| ENSBTAG00000046096 | 23 | 31355570 | 31355274 | -1 | H4C9 |
| ENSBTAG00000048599 | 23 | 31363051 | 31362659 | -1 | H2AC11 |
| ENSBTAG00000054812 | 23 | 31363753 | 31363373 | 1 | H2BC11 |
| ENSBTAG00000054654 | 23 | 31417835 | 31409272 | 1 |  |
| ENSBTAG00000031741 | 23 | 31458492 | 31448749 | 1 | ZNF322 |
| ENSBTAG00000054898 | 23 | 31482890 | 31477974 | 1 | OR2M17 |
| ENSBTAG00000048661 | 23 | 31494679 | 31492889 | 1 | OR2M14 |
| ENSBTAG00000010784 | 23 | 31523923 | 31520971 | -1 | ABT1 |
| ENSBTAG00000047502 | 23 | 9672159 | 9555619 | -1 | FKBP5 |
| ENSBTAG00000010877 | 23 | 9728648 | 9716949 | 1 | ARMC12 |
| ENSBTAG00000048447 | 23 | 9748669 | 9746303 | 1 | CLPSL2 |
| ENSBTAG00000016833 | 23 | 9760669 | 9757684 | -1 | CLPS |
| ENSBTAG00000016834 | 23 | 9783035 | 9767607 | 1 | LHFPL5 |
| ENSBTAG00000016838 | 23 | 9824986 | 9787871 | -1 | SRPK1 |
| ENSBTAG00000046180 | 23 | 9817368 | 9813955 | 1 |  |
| ENSBTAG00000017251 | 23 | 9967961 | 9878961 | -1 | SLC26A8 |
| ENSBTAG00000053805 | 23 | 9898626 | 9898520 | -1 | U6 |
| ENSBTAG00000020783 | 23 | 10044336 | 9969009 | 1 | MAPK14 |
| ENSBTAG00000010007 | 23 | 10067089 | 10058481 | 1 | MAPK13 |
| ENSBTAG00000013316 | 23 | 10154265 | 10118401 | 1 | BRPF3 |
| ENSBTAG00000015118 | 23 | 10245704 | 10187681 | 1 | PNPLA1 |
| ENSBTAG00000017617 | 23 | 10269825 | 10250135 | -1 | BNIP5 |
| ENSBTAG00000052568 | 23 | 10305748 | 10303272 | -1 |  |
| ENSBTAG00000053750 | 23 | 10360192 | 10341272 | -1 | PXT1 |
| ENSBTAG00000020595 | 23 | 10413105 | 10377039 | 1 | KCTD20 |
| ENSBTAG00000011126 | 23 | 10458886 | 10417325 | -1 | STK38 |
| ENSBTAG00000004219 | 23 | 10468845 | 10468465 | 1 |  |
| ENSBTAG00000040006 | 23 | 10509269 | 10502815 | 1 | SRSF3 |
| ENSBTAG00000025617 | 23 | 10565808 | 10550062 | -1 | RPS4Y1 |
| ENSBTAG00000008353 | 23 | 10590589 | 10582308 | 1 | CDKN1A |
| ENSBTAG00000045551 | 23 | 10598507 | 10598199 | 1 |  |
| ENSBTAG00000043057 | 23 | 10603456 | 10603351 | -1 | U6 |
| ENSBTAG00000050445 | 23 | 10643542 | 10616635 | 1 | RAB44 |
| ENSBTAG00000007514 | 23 | 10757196 | 10653672 | -1 | CPNE5 |
| ENSBTAG00000002376 | 23 | 10791508 | 10771195 | -1 | PPIL1 |
| ENSBTAG00000002393 | 23 | 10831744 | 10795467 | 1 | C23H6orf89 |
| ENSBTAG00000002092 | 23 | 10873149 | 10860919 | 1 | PI16 |
| ENSBTAG00000033453 | 23 | 10898204 | 10878590 | -1 | MTCH1 |
| ENSBTAG00000020051 | 25 | 24673861 | 24671805 | 1 | C25H16orf82 |
| ENSBTAG00000019530 | 25 | 24800600 | 24778510 | 1 | KDM8 |
| ENSBTAG00000002176 | 25 | 24839867 | 24801771 | -1 | NSMCE1 |
| ENSBTAG00000001602 | 25 | 24922650 | 24872629 | 1 | IL4R |
| ENSBTAG00000019567 | 25 | 25002153 | 24941958 | 1 | IL21R |
| ENSBTAG00000006126 | 25 | 25089814 | 25009630 | -1 | GTF3C1 |
| ENSBTAG00000006129 | 25 | 25327424 | 25087968 | 1 | KATNIP |
| ENSBTAG00000004607 | 25 | 25591807 | 25339151 | -1 | GSG1L |
| ENSBTAG00000010309 | 25 | 25727702 | 25620580 | -1 | XPO6 |
| ENSBTAG00000032140 | 25 | 25820460 | 25816381 | 1 | SBK1 |
| ENSBTAG00000051235 | 25 | 25838223 | 25837678 | 1 |  |
| ENSBTAG00000032136 | 25 | 25841294 | 25840532 | -1 |  |
| ENSBTAG00000021249 | 25 | 25866924 | 25862056 | -1 | LAT |
| ENSBTAG00000014653 | 25 | 25876113 | 25867538 | -1 | SPNS1 |
| ENSBTAG00000014650 | 25 | 25897161 | 25885086 | -1 | NFATC2IP |
| ENSBTAG00000053850 | 25 | 25903444 | 25899988 | -1 |  |
| ENSBTAG00000032122 | 25 | 25911990 | 25905403 | -1 | CD19 |
| ENSBTAG00000006542 | 25 | 25929296 | 25917049 | 1 | RABEP2 |
| ENSBTAG00000006541 | 25 | 25946430 | 25929240 | -1 | ATP2A1 |
| ENSBTAG00000019219 | 25 | 25956976 | 25950650 | -1 | SH2B1 |
| ENSBTAG00000019216 | 25 | 25971740 | 25967782 | 1 | TUFM |
| ENSBTAG00000032087 | 25 | 25985608 | 25974051 | -1 | ATXN2L |
| ENSBTAG00000006543 | 25 | 26028899 | 26008727 | -1 | EIF3CL |
| ENSBTAG00000006543 | 25 | 26028899 | 26008727 | -1 | EIF3CL |
| ENSBTAG00000006543 | 25 | 26028899 | 26008727 | -1 | EIF3CL |
| ENSBTAG00000018000 | 25 | 26056357 | 26043254 | -1 | CLN3 |
| ENSBTAG00000050361 | 25 | 26060529 | 26056941 | 1 |  |
| ENSBTAG00000018015 | 25 | 26066562 | 26061018 | -1 | IL27 |
| ENSBTAG00000018016 | 25 | 26083376 | 26082047 | -1 | NUPR1 |
| ENSBTAG00000016883 | 25 | 26123366 | 26092599 | 1 | SGF29 |
| ENSBTAG00000008635 | 25 | 26127457 | 26123609 | -1 | SULT1A1 |
| ENSBTAG00000008633 | 25 | 26131638 | 26126027 | -1 | SLX1A |
| ENSBTAG00000008633 | 25 | 26131638 | 26126027 | -1 | SLX1A |
| ENSBTAG00000008632 | 25 | 26132468 | 26131819 | 1 |  |
| ENSBTAG00000008631 | 25 | 26140301 | 26134784 | -1 | CORO1A |
| ENSBTAG00000016156 | 25 | 26186174 | 26178468 | 1 | MAPK3 |
| ENSBTAG00000016155 | 25 | 26191906 | 26186549 | 1 | GDPD3 |
| ENSBTAG00000009047 | 25 | 26196515 | 26193135 | 1 | YPEL3 |
| ENSBTAG00000009046 | 25 | 26201774 | 26197449 | 1 | TBX6 |
| ENSBTAG00000012928 | 25 | 26210403 | 26202157 | -1 | PPP4C |
| ENSBTAG00000012927 | 25 | 26218476 | 26212553 | -1 | ALDOA |
| ENSBTAG00000052420 | 25 | 26215721 | 26215663 | -1 | bta-mir-12060 |
| ENSBTAG00000050743 | 25 | 26225036 | 26219037 | -1 |  |
| ENSBTAG00000048352 | 25 | 26234841 | 26230298 | 1 |  |
| ENSBTAG00000005033 | 25 | 26247628 | 26240206 | 1 | TLCD3B |
| ENSBTAG00000048797 | 25 | 26248379 | 26247838 | -1 | C16orf92 |
| ENSBTAG00000005031 | 25 | 26260480 | 26253986 | 1 | DOC2A |
| ENSBTAG00000005030 | 25 | 26268621 | 26260151 | -1 | INO80E |
| ENSBTAG00000005028 | 25 | 26271508 | 26267918 | 1 | HIRIP3 |
| ENSBTAG00000017422 | 25 | 26289860 | 26272135 | -1 | TAOK2 |
| ENSBTAG00000021785 | 25 | 26307986 | 26290036 | -1 | TMEM219 |
| ENSBTAG00000045841 | 25 | 26302015 | 26300132 | 1 |  |
| ENSBTAG00000015982 | 25 | 26356561 | 26343322 | 1 | KCTD13 |
| ENSBTAG00000026319 | 25 | 26360506 | 26356699 | -1 | ASPHD1 |
| ENSBTAG00000007955 | 25 | 26382375 | 26362158 | 1 | SEZ6L2 |
| ENSBTAG00000053919 | 25 | 26388296 | 26384220 | -1 |  |
| ENSBTAG00000007954 | 25 | 26391768 | 26388343 | 1 | CDIPT |
| ENSBTAG00000007952 | 25 | 26423373 | 26403797 | -1 | MVP |
| ENSBTAG00000010904 | 25 | 26431502 | 26423976 | -1 | PAGR1 |
| ENSBTAG00000046752 | 25 | 26430899 | 26427870 | -1 | PRRT2 |
| ENSBTAG00000052323 | 25 | 26437695 | 26433443 | -1 | MAZ |
| ENSBTAG00000044880 | 25 | 26434763 | 26434689 | -1 | bta-mir-2385 |
| ENSBTAG00000013669 | 25 | 26453320 | 26438296 | -1 | KIF22 |
| ENSBTAG00000049417 | 25 | 26469701 | 26469595 | -1 | U6 |
| ENSBTAG00000047448 | 25 | 26480893 | 26478204 | 1 | C25H16orf54 |
| ENSBTAG00000018082 | 25 | 26506201 | 26490747 | -1 | QPRT |
| ENSBTAG00000026326 | 25 | 26513471 | 26509758 | -1 | SPN |
| ENSBTAG00000054160 | 25 | 26522553 | 26522447 | -1 | U6 |
| ENSBTAG00000015486 | 25 | 26542953 | 26540572 | -1 | CD2BP2 |
| ENSBTAG00000031852 | 25 | 26555450 | 26544203 | -1 | TBC1D10B |
| ENSBTAG00000021218 | 25 | 26561866 | 26558869 | 1 | MYL11 |
| ENSBTAG00000021219 | 25 | 26565656 | 26562037 | -1 | SEPTIN1 |
| ENSBTAG00000031806 | 25 | 26579631 | 26575277 | 1 | ZNF48 |
| ENSBTAG00000021222 | 25 | 26592180 | 26583806 | 1 | ZNF771 |
| ENSBTAG00000039962 | 25 | 26599199 | 26595524 | -1 | DCTPP1 |
| ENSBTAG00000007103 | 25 | 26703485 | 26654119 | 1 | ITGAL |
| ENSBTAG00000048731 | 25 | 26660456 | 26658783 | -1 |  |
| ENSBTAG00000052789 | 25 | 26685032 | 26683758 | -1 |  |
| ENSBTAG00000043974 | 25 | 26712376 | 26709871 | -1 |  |
| ENSBTAG00000051451 | 25 | 26724280 | 26717521 | -1 |  |
| ENSBTAG00000019357 | 25 | 26737926 | 26735144 | -1 | ZNF688 |
| ENSBTAG00000018094 | 25 | 26760550 | 26756299 | -1 | ZNF689 |
| ENSBTAG00000009099 | 25 | 26781350 | 26776309 | 1 | PRR14 |
| ENSBTAG00000031789 | 25 | 26794861 | 26783860 | 1 | FBRS |
| ENSBTAG00000013917 | 25 | 26844133 | 26832768 | 1 |  |
| ENSBTAG00000042276 | 25 | 26838416 | 26838286 | 1 |  |
| ENSBTAG00000054329 | 25 | 26860416 | 26859105 | 1 |  |
| ENSBTAG00000050825 | 25 | 26864354 | 26861212 | 1 | TMEM265 |
| ENSBTAG00000043199 | 25 | 26867479 | 26867375 | -1 | U6 |
| ENSBTAG00000003417 | 25 | 26878577 | 26869325 | 1 | PHKG2 |
| ENSBTAG00000031765 | 25 | 26882751 | 26876842 | -1 | CFAP119 |
| ENSBTAG00000003421 | 25 | 26894901 | 26883214 | 1 | RNF40 |
| ENSBTAG00000049596 | 25 | 26896444 | 26893401 | -1 |  |
| ENSBTAG00000026307 | 25 | 26924098 | 26901426 | -1 | ZNF629 |
| ENSBTAG00000008412 | 25 | 26969724 | 26938951 | -1 | BCL7C |
| ENSBTAG00000017697 | 25 | 26976313 | 26971595 | 1 | CTF1 |
| ENSBTAG00000053298 | 25 | 26980456 | 26977191 | -1 |  |
| ENSBTAG00000040555 | 25 | 27086008 | 27002887 | 1 | FBXL19 |
| ENSBTAG00000048857 | 25 | 27010937 | 27007831 | -1 |  |
| ENSBTAG00000050574 | 25 | 27046838 | 27040934 | -1 | CTF2 |
| ENSBTAG00000002344 | 25 | 27092751 | 27087303 | 1 | ORAI3 |
| ENSBTAG00000002345 | 25 | 27122818 | 27095052 | 1 | SETD1A |
| ENSBTAG00000002346 | 25 | 27122802 | 27119323 | 1 | HSD3B7 |
| ENSBTAG00000002349 | 25 | 27141974 | 27125156 | -1 | STX1B |
| ENSBTAG00000007523 | 25 | 27163281 | 27156826 | 1 | STX4 |
| ENSBTAG00000006650 | 25 | 27184380 | 27175346 | -1 | ZNF668 |
| ENSBTAG00000051572 | 25 | 27193576 | 27184092 | 1 | ZNF646 |
| ENSBTAG00000031551 | 25 | 27199629 | 27194320 | -1 | PRSS53 |
| ENSBTAG00000000405 | 25 | 27204251 | 27201063 | -1 | VKORC1 |
| ENSBTAG00000010524 | 25 | 27216927 | 27212130 | 1 | BCKDK |
| ENSBTAG00000010511 | 25 | 27228735 | 27217760 | 1 | KAT8 |
| ENSBTAG00000016973 | 25 | 27233122 | 27228651 | -1 | PRSS8 |
| ENSBTAG00000037397 | 25 | 27243964 | 27236563 | -1 | PRSS36 |
| ENSBTAG00000005757 | 25 | 27277781 | 27267596 | 1 | FUS |
| ENSBTAG00000020535 | 25 | 27286399 | 27285067 | -1 | PYCARD |
| ENSBTAG00000001503 | 25 | 27305411 | 27295750 | 1 | TRIM72 |
| ENSBTAG00000047238 | 25 | 27381713 | 27344004 | 1 | ITGAM |
| ENSBTAG00000054461 | 25 | 27417799 | 27398592 | 1 |  |
| ENSBTAG00000019524 | 25 | 27480928 | 27451624 | 1 | ITGAD |
| ENSBTAG00000019521 | 25 | 27481274 | 27479986 | -1 | COX6A2 |
| ENSBTAG00000002374 | 25 | 27500855 | 27493317 | 1 | ARMC5 |
| ENSBTAG00000002391 | 25 | 27510659 | 27503118 | 1 | TGFB1I1 |
| ENSBTAG00000010096 | 25 | 27523749 | 27513955 | 1 | SLC5A2 |
| ENSBTAG00000047734 | 25 | 27537894 | 27523808 | -1 | RUSF1 |
| ENSBTAG00000006457 | 25 | 27544133 | 27543234 | 1 | AHSP |
| ENSBTAG00000040319 | 25 | 27554945 | 27553977 | -1 | OR7A53 |
| ENSBTAG00000037462 | 25 | 27580110 | 27579124 | 1 | OR7A153 |
| ENSBTAG00000009327 | 25 | 27611278 | 27581047 | -1 | SEPTIN14 |
| ENSBTAG00000008175 | 25 | 27637076 | 27624063 | 1 | ZNF713 |
| ENSBTAG00000007934 | 25 | 27658120 | 27653220 | 1 | MRPS17 |
| ENSBTAG00000031598 | 25 | 27688636 | 27661422 | 1 | NIPSNAP2 |
| ENSBTAG00000013081 | 25 | 27719287 | 27697184 | -1 | PSPH |
| ENSBTAG00000008184 | 25 | 27738084 | 27727918 | 1 | CCT6A |
| ENSBTAG00000042366 | 25 | 27730811 | 27730678 | 1 |  |
| ENSBTAG00000046466 | 25 | 27734668 | 27734528 | 1 |  |
| ENSBTAG00000008190 | 25 | 27754221 | 27738682 | 1 | SUMF2 |
| ENSBTAG00000008195 | 25 | 27763437 | 27749099 | -1 | PHKG1 |
| ENSBTAG00000040295 | 25 | 27766328 | 27760604 | -1 | CHCHD2 |
| ENSBTAG00000031583 | 25 | 27772376 | 27771475 | -1 | NUPR2 |
| ENSBTAG00000031582 | 25 | 27778361 | 27778005 | -1 |  |
| ENSBTAG00000014448 | 25 | 27901668 | 27845210 | 1 | VKORC1L1 |
| ENSBTAG00000000704 | 25 | 27918749 | 27898147 | -1 | GUSB |
| ENSBTAG00000055247 | 25 | 27950669 | 27949314 | 1 |  |
| ENSBTAG00000052611 | 25 | 27978745 | 27966980 | 1 |  |
| ENSBTAG00000015314 | 25 | 27998697 | 27989110 | -1 | ASL |
| ENSBTAG00000037489 | 25 | 28036980 | 27997740 | 1 | CRCP |
| ENSBTAG00000000390 | 25 | 28188134 | 28081759 | 1 | TPST1 |
| ENSBTAG00000017435 | 25 | 28215217 | 28204182 | 1 | KCTD7 |
| ENSBTAG00000003842 | 25 | 28275671 | 28224570 | 1 | RABGEF1 |
| ENSBTAG00000046622 | 25 | 28248477 | 28248165 | -1 | Metazoa_SRP |
| ENSBTAG00000002935 | 25 | 28319832 | 28293459 | 1 | TMEM248 |
| ENSBTAG00000004051 | 25 | 28377073 | 28371598 | -1 | SBDS |
| ENSBTAG00000008446 | 25 | 28522373 | 28378765 | 1 | TYW1 |
| ENSBTAG00000043969 | 25 | 29090972 | 28636197 | 1 | CALN1 |
| ENSBTAG00000008718 | 25 | 29563785 | 29133705 | -1 | GALNT17 |
| ENSBTAG00000048714 | 25 | 29242291 | 29242217 | 1 | bta-mir-2386 |
| ENSBTAG00000002083 | 25 | 29817161 | 29782669 | -1 | AUTS2 |
| ENSBTAG00000039738 | 3 | 31829058 | 31745649 | 1 | TMIGD3 |
| ENSBTAG00000052822 | 3 | 31836548 | 31834096 | -1 | C3H1orf162 |
| ENSBTAG00000006441 | 3 | 31863330 | 31846429 | -1 | ATP5PB |
| ENSBTAG00000014102 | 3 | 31873115 | 31863581 | 1 | WDR77 |
| ENSBTAG00000005973 | 3 | 31901999 | 31890059 | 1 | OVGP1 |
| ENSBTAG00000034841 | 3 | 31923925 | 31915199 | 1 |  |
| ENSBTAG00000002489 | 3 | 31958001 | 31952318 | -1 |  |
| ENSBTAG00000024849 | 3 | 31984308 | 31974420 | 1 |  |
| ENSBTAG00000053654 | 3 | 31984640 | 31982659 | -1 |  |
| ENSBTAG00000023535 | 3 | 32051900 | 32034788 | 1 |  |
| ENSBTAG00000050402 | 3 | 32057649 | 32049915 | -1 |  |
| ENSBTAG00000048596 | 3 | 32060390 | 32058825 | 1 |  |
| ENSBTAG00000000259 | 3 | 32090853 | 32071080 | -1 | CHIA |
| ENSBTAG00000013578 | 3 | 32192735 | 32155439 | -1 | CHI3L2 |
| ENSBTAG00000020172 | 3 | 32224615 | 32204153 | 1 | DENND2D |
| ENSBTAG00000050725 | 3 | 32205128 | 32204829 | -1 |  |
| ENSBTAG00000020169 | 3 | 32272255 | 32227187 | -1 | CEPT1 |
| ENSBTAG00000050534 | 3 | 32264893 | 32261528 | 1 |  |
| ENSBTAG00000009888 | 3 | 32300821 | 32272397 | 1 | DRAM2 |
| ENSBTAG00000012659 | 3 | 32428000 | 32408226 | 1 | LRIF1 |
| ENSBTAG00000053209 | 3 | 32472098 | 32471098 | -1 |  |
| ENSBTAG00000042091 | 3 | 32485129 | 32485026 | -1 | U6 |
| ENSBTAG00000006466 | 3 | 32533031 | 32494132 | -1 | CD53 |
| ENSBTAG00000047879 | 3 | 32747031 | 32745301 | 1 | KCNA3 |
| ENSBTAG00000015459 | 3 | 32834145 | 32830656 | 1 | KCNA2 |
| ENSBTAG00000008322 | 3 | 32923604 | 32922069 | 1 | KCNA10 |
| ENSBTAG00000010565 | 3 | 32954911 | 32944105 | -1 | CYM |
| ENSBTAG00000010560 | 3 | 32974969 | 32969972 | -1 | PROK1 |
| ENSBTAG00000014970 | 3 | 33031126 | 33026800 | 1 | LAMTOR5 |
| ENSBTAG00000015380 | 3 | 33074902 | 33044290 | 1 | SLC16A4 |
| ENSBTAG00000012555 | 3 | 33095448 | 33088523 | -1 | RBM15 |
| ENSBTAG00000007352 | 3 | 33216772 | 33193740 | -1 | KCNC4 |
| ENSBTAG00000001981 | 3 | 33281966 | 33226163 | -1 | SLC6A17 |
| ENSBTAG00000053616 | 3 | 33245734 | 33245673 | -1 | bta-mir-2285as-3 |
| ENSBTAG00000016389 | 3 | 33317748 | 33316136 | -1 | UBL4B |
| ENSBTAG00000020966 | 3 | 33370572 | 33360536 | 1 | ALX3 |
| ENSBTAG00000020964 | 3 | 33393926 | 33374163 | -1 | STRIP1 |
| ENSBTAG00000018893 | 3 | 33444827 | 33403800 | -1 | AHCYL1 |
| ENSBTAG00000000283 | 3 | 33509479 | 33488957 | -1 | CSF1 |
| ENSBTAG00000044953 | 3 | 33588455 | 33588377 | -1 | bta-mir-2413 |
| ENSBTAG00000001845 | 3 | 33649761 | 33634806 | 1 | EPS8L3 |
| ENSBTAG00000001842 | 3 | 33655778 | 33652466 | 1 | GSTM3 |
| ENSBTAG00000017765 | 3 | 33720169 | 33667202 | -1 | GSTM1 |
| ENSBTAG00000012692 | 3 | 33731268 | 33690892 | -1 |  |
| ENSBTAG00000037673 | 3 | 33765942 | 33759204 | -1 | GSTM1 |
| ENSBTAG00000013018 | 3 | 33830235 | 33818280 | -1 | AMPD2 |
| ENSBTAG00000013017 | 3 | 33846790 | 33837406 | 1 | GNAT2 |
| ENSBTAG00000029921 | 3 | 33850375 | 33850268 | -1 | MIR197 |
| ENSBTAG00000013016 | 3 | 33899237 | 33854858 | -1 | GNAI3 |
| ENSBTAG00000013014 | 3 | 33905313 | 33901936 | -1 | GPR61 |
| ENSBTAG00000014083 | 3 | 33945726 | 33940232 | 1 | AMIGO1 |
| ENSBTAG00000016643 | 3 | 33954930 | 33946014 | -1 | CYB561D1 |
| ENSBTAG00000009042 | 3 | 33965040 | 33956341 | -1 | ATXN7L2 |
| ENSBTAG00000015470 | 3 | 33981459 | 33968205 | -1 | SYPL2 |
| ENSBTAG00000020641 | 3 | 34033681 | 34012638 | 1 | PSMA5 |
| ENSBTAG00000008849 | 3 | 34100367 | 34036271 | 1 | SORT1 |
| ENSBTAG00000018807 | 3 | 34122165 | 34107374 | 1 | MYBPHL |
| ENSBTAG00000018806 | 3 | 34129175 | 34122368 | 1 | PSRC1 |
| ENSBTAG00000018804 | 3 | 34157519 | 34131464 | -1 | CELSR2 |
| ENSBTAG00000012962 | 3 | 34190011 | 34170143 | -1 | SARS1 |
| ENSBTAG00000001136 | 3 | 34215659 | 34200677 | -1 | ELAPOR1 |
| ENSBTAG00000051851 | 3 | 34239641 | 34219155 | -1 |  |
| ENSBTAG00000001135 | 3 | 34280108 | 34274613 | 1 | CFAP276 |
| ENSBTAG00000045530 | 3 | 34281597 | 34281165 | -1 | SCARNA2 |
| ENSBTAG00000038979 | 3 | 34291820 | 34286939 | -1 | TMEM167B |
| ENSBTAG00000049309 | 3 | 34311597 | 34304171 | 1 | TAF13 |
| ENSBTAG00000031655 | 3 | 34324753 | 34324532 | 1 |  |
| ENSBTAG00000020384 | 3 | 34394375 | 34331946 | 1 | WDR47 |
| ENSBTAG00000017129 | 3 | 34430982 | 34397588 | 1 | CLCC1 |
| ENSBTAG00000017124 | 3 | 34485343 | 34431970 | -1 | GPSM2 |
| ENSBTAG00000044600 | 3 | 34456164 | 34455829 | -1 | RNaseP_nuc |
| ENSBTAG00000044430 | 3 | 34458755 | 34458623 | -1 | SNORA70 |
| ENSBTAG00000007333 | 3 | 34521119 | 34517748 | 1 |  |
| ENSBTAG00000051792 | 3 | 34560535 | 34557009 | 1 |  |
| ENSBTAG00000007330 | 3 | 34622399 | 34568247 | -1 | STXBP3 |
| ENSBTAG00000050538 | 3 | 34595637 | 34594141 | 1 |  |
| ENSBTAG00000046771 | 3 | 34658968 | 34633213 | -1 | FNDC7 |
| ENSBTAG00000047874 | 3 | 34681037 | 34673537 | -1 | PRPF38B |
| ENSBTAG00000008467 | 3 | 34714161 | 34702129 | 1 | HENMT1 |
| ENSBTAG00000005439 | 3 | 34805116 | 34726393 | -1 | FAM102B |
| ENSBTAG00000054598 | 3 | 34907441 | 34844369 | 1 |  |
| ENSBTAG00000014297 | 3 | 30652245 | 30624734 | -1 | MOV10 |
| ENSBTAG00000014295 | 3 | 30701981 | 30657802 | -1 | CAPZA1 |
| ENSBTAG00000014294 | 3 | 30844632 | 30702703 | 1 | ST7L |
| ENSBTAG00000014291 | 3 | 30805442 | 30790883 | -1 | WNT2B |
| ENSBTAG00000021602 | 3 | 30885081 | 30834932 | -1 | CTTNBP2NL |
| ENSBTAG00000050797 | 3 | 30858980 | 30858577 | 1 |  |
| ENSBTAG00000031654 | 3 | 31530748 | 31311513 | 1 | KCND3 |
| ENSBTAG00000018717 | 3 | 31550254 | 31539743 | -1 | DDX20 |
| ENSBTAG00000022580 | 3 | 31578746 | 31565399 | 1 | INKA2 |
| ENSBTAG00000050904 | 3 | 31676077 | 31588106 | -1 | RAP1A |
| ENSBTAG00000018645 | 4 | 14408479 | 14404123 | -1 | DLX5 |
| ENSBTAG00000054132 | 4 | 14466823 | 14463630 | -1 |  |
| ENSBTAG00000015925 | 4 | 14567618 | 14476805 | 1 | SDHAF3 |
| ENSBTAG00000015356 | 4 | 15023433 | 15014498 | 1 | TAC1 |
| ENSBTAG00000003222 | 4 | 15276513 | 15128694 | -1 | ASNS |
| ENSBTAG00000044673 | 4 | 15184681 | 15184577 | 1 |  |
| ENSBTAG00000004995 | 4 | 15320627 | 15276632 | 1 | C1GALT1 |
| ENSBTAG00000024420 | 4 | 15603392 | 15425637 | -1 | COL28A1 |
| ENSBTAG00000013781 | 4 | 15672063 | 15634972 | 1 | MIOS |
| ENSBTAG00000050627 | 4 | 15670201 | 15669853 | -1 |  |
| ENSBTAG00000013168 | 4 | 15695690 | 15692488 | -1 | RPA3 |
| ENSBTAG00000047004 | 4 | 15991563 | 15734909 | 1 | UMAD1 |
| ENSBTAG00000044405 | 4 | 15853005 | 15852947 | -1 | U6 |
| ENSBTAG00000043805 | 4 | 15943556 | 15943455 | -1 | 5S_rRNA |
| ENSBTAG00000008533 | 4 | 16156180 | 16051521 | 1 | GLCCI1 |
| ENSBTAG00000009823 | 4 | 86627801 | 86627249 | -1 |  |
| ENSBTAG00000000799 | 4 | 16355628 | 16192292 | -1 | ICA1 |
| ENSBTAG00000005103 | 4 | 86718421 | 86715415 | -1 | FEZF1 |
| ENSBTAG00000005110 | 4 | 87314496 | 86734600 | -1 | CADPS2 |
| ENSBTAG00000055236 | 4 | 16376679 | 16371841 | -1 |  |
| ENSBTAG00000025604 | 4 | 16376872 | 16376195 | 1 |  |
| ENSBTAG00000045078 | 4 | 16476057 | 16475905 | -1 | U1 |
| ENSBTAG00000045937 | 4 | 16781333 | 16779419 | 1 |  |
| ENSBTAG00000054892 | 4 | 16779803 | 16779758 | 1 |  |
| ENSBTAG00000006924 | 4 | 16890150 | 16889144 | 1 | NXPH1 |
| ENSBTAG00000032049 | 4 | 87408316 | 87407411 | -1 | TAS2R16 |
| ENSBTAG00000052259 | 4 | 18381579 | 18381040 | 1 |  |
| ENSBTAG00000006674 | 4 | 87568389 | 87464059 | -1 | SLC13A1 |
| ENSBTAG00000049682 | 4 | 18476123 | 18475535 | 1 |  |
| ENSBTAG00000043043 | 4 | 18984623 | 18984474 | -1 | SNORA62 |
| ENSBTAG00000038527 | 4 | 19018355 | 19017935 | -1 |  |
| ENSBTAG00000011145 | 4 | 19049196 | 19041321 | -1 | NDUFA4 |
| ENSBTAG00000004405 | 4 | 87862973 | 87783954 | -1 | IQUB |
| ENSBTAG00000044664 | 4 | 87837289 | 87837227 | 1 | U7 |
| ENSBTAG00000009334 | 4 | 87887305 | 87871092 | -1 | NDUFA5 |
| ENSBTAG00000011819 | 4 | 19077110 | 19065677 | 1 | PHF14 |
| ENSBTAG00000002151 | 4 | 87942478 | 87910057 | 1 | ASB15 |
| ENSBTAG00000053922 | 4 | 19196937 | 19160155 | 1 |  |
| ENSBTAG00000002157 | 4 | 87968300 | 87959332 | 1 | LMOD2 |
| ENSBTAG00000004643 | 4 | 88069929 | 87988365 | -1 | WASL |
| ENSBTAG00000007680 | 4 | 19990379 | 19514202 | -1 | THSD7A |
| ENSBTAG00000039304 | 4 | 88142636 | 88142432 | -1 | U3 |
| ENSBTAG00000054421 | 4 | 88162095 | 88160770 | -1 |  |
| ENSBTAG00000019750 | 4 | 20388726 | 20366146 | 1 | TMEM106B |
| ENSBTAG00000005548 | 4 | 88190862 | 88178402 | 1 | HYAL4 |
| ENSBTAG00000004640 | 4 | 88222320 | 88202846 | 1 | SPAM1 |
| ENSBTAG00000028457 | 4 | 88220045 | 88219942 | -1 | 5S_rRNA |
| ENSBTAG00000040349 | 4 | 88284027 | 88267317 | 1 |  |
| ENSBTAG00000051912 | 4 | 20557546 | 20556638 | 1 |  |
| ENSBTAG00000009786 | 4 | 20703669 | 20624426 | 1 | SCIN |
| ENSBTAG00000031958 | 4 | 88377510 | 88370599 | 1 |  |
| ENSBTAG00000036646 | 4 | 88375393 | 88375270 | -1 | 5S_rRNA |
| ENSBTAG00000022880 | 4 | 88431290 | 88429070 | -1 |  |
| ENSBTAG00000049382 | 4 | 88452025 | 88450928 | -1 | TMEM229A |
| ENSBTAG00000042864 | 4 | 88928798 | 88928692 | 1 | U6 |
| ENSBTAG00000013732 | 4 | 89037983 | 89019866 | -1 | GPR37 |
| ENSBTAG00000053466 | 4 | 20757134 | 20756719 | 1 |  |
| ENSBTAG00000002869 | 4 | 20766198 | 20764660 | 1 | ARL4A |
| ENSBTAG00000013180 | 4 | 89183193 | 89093139 | -1 | POT1 |
| ENSBTAG00000043674 | 4 | 21594980 | 21594863 | -1 | 5S_rRNA |
| ENSBTAG00000042514 | 4 | 21914164 | 21914043 | 1 |  |
| ENSBTAG00000015981 | 4 | 22195687 | 22099728 | -1 | ETV1 |
| ENSBTAG00000054974 | 4 | 91510132 | 90671659 | -1 | GRM8 |
| ENSBTAG00000044941 | 4 | 90931205 | 90931081 | -1 |  |
| ENSBTAG00000030030 | 4 | 91320695 | 91320600 | -1 | bta-mir-592 |
| ENSBTAG00000020004 | 4 | 91638634 | 91589029 | -1 | ZNF800 |
| ENSBTAG00000043830 | 4 | 91685745 | 91685454 | -1 | 7SK |
| ENSBTAG00000003102 | 4 | 91815933 | 91811881 | -1 | GCC1 |
| ENSBTAG00000009366 | 4 | 91822148 | 91819098 | 1 | ARF5 |
| ENSBTAG00000009365 | 4 | 91828161 | 91824134 | 1 | FSCN3 |
| ENSBTAG00000000410 | 4 | 91840881 | 91836115 | -1 | PAX4 |
| ENSBTAG00000010692 | 4 | 92296106 | 91873756 | 1 | SND1 |
| ENSBTAG00000010704 | 4 | 92233611 | 92143302 | -1 | LRRC4 |
| ENSBTAG00000029915 | 4 | 92408020 | 92407937 | 1 | MIR129-1 |
| ENSBTAG00000014911 | 4 | 92453653 | 92436922 | 1 | LEP |
| ENSBTAG00000017001 | 4 | 92531680 | 92499373 | -1 | RBM28 |
| ENSBTAG00000021905 | 4 | 23262263 | 22389767 | -1 | DGKB |
| ENSBTAG00000043411 | 4 | 23304904 | 23304802 | -1 | U6 |
| ENSBTAG00000047482 | 4 | 92545269 | 92537251 | -1 | PRRT4 |
| ENSBTAG00000031866 | 4 | 92569356 | 92565558 | 1 |  |
| ENSBTAG00000000296 | 4 | 92586808 | 92569423 | -1 | IMPDH1 |
| ENSBTAG00000024291 | 4 | 92674005 | 92647910 | 1 | GARIN1A |
| ENSBTAG00000009867 | 4 | 92711399 | 92695804 | 1 | GARIN1B |
| ENSBTAG00000016481 | 4 | 92744873 | 92719265 | 1 | CALU |
| ENSBTAG00000016485 | 4 | 92748928 | 92745939 | -1 | OPN1SW |
| ENSBTAG00000011002 | 4 | 92792188 | 92763331 | 1 | CCDC136 |
| ENSBTAG00000054772 | 4 | 23818155 | 23549707 | 1 |  |
| ENSBTAG00000006253 | 4 | 92825735 | 92797675 | 1 | FLNC |
| ENSBTAG00000034154 | 4 | 23947179 | 23557610 | -1 | AGMO |
| ENSBTAG00000003238 | 4 | 24075104 | 23999242 | -1 | MEOX2 |
| ENSBTAG00000015073 | 4 | 24921062 | 24500692 | -1 | CRPPA |
| ENSBTAG00000005377 | 4 | 24966045 | 24961801 | -1 | SOSTDC1 |
| ENSBTAG00000034091 | 4 | 25084046 | 25025019 | 1 | LRRC72 |
| ENSBTAG00000045109 | 4 | 92808181 | 92808114 | 1 | bta-mir-2422 |
| ENSBTAG00000007572 | 4 | 92831704 | 92828149 | 1 | ATP6V1F |
| ENSBTAG00000010980 | 4 | 25130458 | 25090913 | -1 | ANKMY2 |
| ENSBTAG00000045645 | 4 | 92834051 | 92831983 | 1 | ATP6V1FNB |
| ENSBTAG00000052301 | 4 | 92839050 | 92834495 | -1 |  |
| ENSBTAG00000003449 | 4 | 92873142 | 92843936 | -1 | KCP |
| ENSBTAG00000014262 | 4 | 25193513 | 25130690 | 1 | BZW2 |
| ENSBTAG00000012805 | 4 | 25283286 | 25245149 | 1 | TSPAN13 |
| ENSBTAG00000024406 | 4 | 25304186 | 25290663 | -1 | AGR2 |
| ENSBTAG00000045578 | 4 | 25366158 | 25349573 | -1 | AGR3 |
| ENSBTAG00000054646 | 4 | 25591857 | 25591651 | -1 |  |
| ENSBTAG00000007746 | 4 | 25855901 | 25805796 | 1 | AHR |
| ENSBTAG00000043006 | 4 | 25846152 | 25846087 | -1 | SNORD31 |
| ENSBTAG00000042327 | 4 | 26118654 | 26118550 | -1 | U6 |
| ENSBTAG00000014074 | 4 | 26469689 | 26324012 | -1 | SNX13 |
| ENSBTAG00000004989 | 4 | 92905297 | 92892127 | 1 | IRF5 |
| ENSBTAG00000033954 | 4 | 26563847 | 26562725 | -1 | PRPS1L1 |
| ENSBTAG00000051137 | 4 | 26746600 | 26624419 | 1 |  |
| ENSBTAG00000044780 | 4 | 26744611 | 26744491 | -1 |  |
| ENSBTAG00000004992 | 4 | 92987982 | 92909747 | -1 | TNPO3 |
| ENSBTAG00000003808 | 4 | 27667961 | 27093041 | 1 | HDAC9 |
| ENSBTAG00000044868 | 4 | 92988191 | 92988123 | -1 | bta-mir-1843 |
| ENSBTAG00000007253 | 4 | 93088762 | 93068851 | 1 | TSPAN33 |
| ENSBTAG00000013287 | 4 | 93132945 | 93108630 | 1 | SMO |
| ENSBTAG00000046922 | 4 | 27819874 | 27817880 | -1 | TWIST1 |
| ENSBTAG00000039955 | 4 | 27854966 | 27854472 | -1 | FERD3L |
| ENSBTAG00000006721 | 4 | 28410479 | 28399526 | -1 | POLR1F |
| ENSBTAG00000001754 | 4 | 93333822 | 93142664 | 1 | AHCYL2 |
| ENSBTAG00000050341 | 4 | 28421508 | 28417813 | -1 |  |
| ENSBTAG00000044012 | 4 | 28496683 | 28422955 | -1 | TMEM196 |
| ENSBTAG00000051697 | 4 | 93190297 | 93188917 | 1 |  |
| ENSBTAG00000003751 | 4 | 28886703 | 28790424 | -1 | MACC1 |
| ENSBTAG00000007206 | 4 | 93401401 | 93338888 | 1 | STRIP2 |
| ENSBTAG00000014273 | 4 | 93576616 | 93449966 | 1 | NRF1 |
| ENSBTAG00000003709 | 4 | 29115760 | 29024090 | 1 | ITGB8 |
| ENSBTAG00000030118 | 4 | 93591534 | 93591424 | -1 | bta-mir-182 |
| ENSBTAG00000029977 | 4 | 93596022 | 93595920 | -1 | bta-mir-96 |
| ENSBTAG00000029762 | 4 | 93596248 | 93596139 | -1 | MIR183 |
| ENSBTAG00000047845 | 4 | 93616147 | 93615932 | -1 |  |
| ENSBTAG00000000911 | 4 | 93748886 | 93649542 | -1 | UBE2H |
| ENSBTAG00000011247 | 4 | 93835784 | 93819542 | -1 | ZC3HC1 |
| ENSBTAG00000002595 | 4 | 29474876 | 29354689 | 1 | ABCB5 |
| ENSBTAG00000000548 | 4 | 93922707 | 93862090 | 1 | KLHDC10 |
| ENSBTAG00000050404 | 4 | 93935256 | 93935150 | 1 | U6 |
| ENSBTAG00000015588 | 4 | 93983043 | 93952779 | -1 | TMEM209 |
| ENSBTAG00000055228 | 4 | 93966040 | 93964466 | 1 |  |
| ENSBTAG00000013473 | 4 | 93995180 | 93985493 | 1 | SSMEM1 |
| ENSBTAG00000046648 | 4 | 94070532 | 94046269 | 1 | CPA4 |
| ENSBTAG00000013476 | 4 | 94118110 | 94063807 | 1 | CPA5 |
| ENSBTAG00000046169 | 4 | 94135232 | 94128586 | 1 | CPA1 |
| ENSBTAG00000007413 | 4 | 94192546 | 94144405 | -1 | CEP41 |
| ENSBTAG00000054642 | 4 | 94246745 | 94246647 | 1 |  |
| ENSBTAG00000052730 | 4 | 94247349 | 94247106 | 1 |  |
| ENSBTAG00000048995 | 4 | 94248710 | 94248612 | 1 |  |
| ENSBTAG00000017223 | 4 | 94263796 | 94250156 | 1 | MEST |
| ENSBTAG00000029800 | 4 | 94255174 | 94255083 | 1 | bta-mir-335 |
| ENSBTAG00000017245 | 4 | 94443596 | 94264008 | -1 | COPG2 |
| ENSBTAG00000020183 | 4 | 94468216 | 94443863 | -1 | TSGA13 |
| ENSBTAG00000052251 | 4 | 94524428 | 94523445 | -1 | KLF14 |
| ENSBTAG00000042721 | 4 | 94600697 | 94600564 | -1 |  |
| ENSBTAG00000029884 | 4 | 94636537 | 94636455 | -1 | MIR29A |
| ENSBTAG00000029980 | 4 | 94636925 | 94636840 | -1 | bta-mir-29b-1 |
| ENSBTAG00000049855 | 4 | 94848101 | 94734002 | -1 |  |
| ENSBTAG00000051415 | 4 | 94848328 | 94848122 | -1 |  |
| ENSBTAG00000051746 | 4 | 94940528 | 94849946 | 1 |  |
| ENSBTAG00000049664 | 4 | 94982345 | 94961149 | -1 |  |
| ENSBTAG00000050548 | 4 | 95025975 | 95023570 | -1 |  |
| ENSBTAG00000010437 | 4 | 95232557 | 95025992 | 1 | MKLN1 |
| ENSBTAG00000010452 | 4 | 95294639 | 95248660 | -1 | PODXL |
| ENSBTAG00000054979 | 4 | 95485100 | 95480727 | -1 |  |
| ENSBTAG00000045386 | 4 | 95631921 | 95631850 | -1 | bta-mir-320b |
| ENSBTAG00000014543 | 4 | 95958170 | 95795104 | -1 | PLXNA4 |
| ENSBTAG00000002604 | 4 | 29495564 | 29493047 | -1 | SP8 |
| ENSBTAG00000049946 | 4 | 96270788 | 96089025 | -1 |  |
| ENSBTAG00000045673 | 4 | 30029833 | 30029423 | 1 |  |
| ENSBTAG00000014389 | 4 | 30305513 | 30225647 | 1 | SP4 |
| ENSBTAG00000024723 | 4 | 96761348 | 96468817 | -1 | CHCHD3 |
| ENSBTAG00000014112 | 4 | 97744748 | 96940092 | 1 | EXOC4 |
| ENSBTAG00000044499 | 4 | 97217497 | 97217425 | 1 | bta-mir-2423 |
| ENSBTAG00000015773 | 4 | 97890975 | 97789937 | 1 | LRGUK |
| ENSBTAG00000003372 | 4 | 97975400 | 97939642 | -1 | SLC35B4 |
| ENSBTAG00000009902 | 4 | 98132148 | 98115288 | -1 | AKR1B1 |
| ENSBTAG00000001582 | 4 | 98239457 | 98217455 | 1 | AKR1B10 |
| ENSBTAG00000008895 | 4 | 98353157 | 98321779 | 1 | BPGM |
| ENSBTAG00000052204 | 4 | 98549793 | 98476141 | 1 |  |
| ENSBTAG00000013953 | 4 | 98679000 | 98573632 | 1 | CALD1 |
| ENSBTAG00000013078 | 4 | 30693265 | 30324683 | 1 | DNAH11 |
| ENSBTAG00000052279 | 4 | 98658750 | 98655797 | -1 |  |
| ENSBTAG00000013976 | 4 | 98789432 | 98689853 | 1 | AGBL3 |
| ENSBTAG00000042841 | 4 | 98792227 | 98792121 | 1 | U6 |
| ENSBTAG00000001576 | 4 | 98812609 | 98798511 | 1 | TMEM140 |
| ENSBTAG00000001773 | 4 | 98814946 | 98812496 | -1 | CYREN |
| ENSBTAG00000017905 | 4 | 98849268 | 98822560 | -1 | WDR91 |
| ENSBTAG00000036475 | 4 | 98829754 | 98829636 | 1 | 5S_rRNA |
| ENSBTAG00000001346 | 4 | 98885072 | 98862169 | 1 | STRA8 |
| ENSBTAG00000031548 | 4 | 98969970 | 98884574 | -1 |  |
| ENSBTAG00000049834 | 4 | 98912513 | 98909679 | 1 |  |
| ENSBTAG00000047605 | 4 | 99055474 | 98980494 | -1 |  |
| ENSBTAG00000052991 | 4 | 99014713 | 99012599 | 1 |  |
| ENSBTAG00000054978 | 4 | 99088132 | 99079895 | -1 |  |
| ENSBTAG00000047088 | 4 | 99135175 | 99091792 | -1 |  |
| ENSBTAG00000017919 | 4 | 99335717 | 99183547 | -1 | CNOT4 |
| ENSBTAG00000011127 | 4 | 99461007 | 99335497 | 1 | NUP205 |
| ENSBTAG00000051809 | 4 | 99477987 | 99462650 | 1 | STMP1 |
| ENSBTAG00000015551 | 4 | 99482705 | 99482020 | -1 |  |
| ENSBTAG00000003864 | 4 | 99526129 | 99483644 | -1 | SLC13A4 |
| ENSBTAG00000017427 | 4 | 99546135 | 99532570 | -1 | FAM180A |
| ENSBTAG00000007806 | 4 | 99827165 | 99750823 | -1 | MTPN |
| ENSBTAG00000031458 | 4 | 1E+08 | 1E+08 | 1 |  |
| ENSBTAG00000029965 | 4 | 1.01E+08 | 1.01E+08 | 1 | bta-mir-490 |
| ENSBTAG00000014674 | 4 | 1.01E+08 | 1.01E+08 | 1 | CHRM2 |
| ENSBTAG00000002317 | 4 | 1.01E+08 | 1.01E+08 | -1 | PTN |
| ENSBTAG00000019980 | 4 | 1.02E+08 | 1.01E+08 | -1 | DGKI |
| ENSBTAG00000015802 | 4 | 1.02E+08 | 1.02E+08 | -1 | CREB3L2 |
| ENSBTAG00000050521 | 4 | 1.02E+08 | 1.02E+08 | -1 | bta-mir-12013 |
| ENSBTAG00000043638 | 4 | 1.02E+08 | 1.02E+08 | 1 | 7SK |
| ENSBTAG00000001658 | 4 | 1.02E+08 | 1.02E+08 | 1 | AKR1D1 |
| ENSBTAG00000002431 | 4 | 1.02E+08 | 1.02E+08 | 1 | TRIM24 |
| ENSBTAG00000050451 | 4 | 1.02E+08 | 1.02E+08 | -1 |  |
| ENSBTAG00000031265 | 4 | 1.02E+08 | 1.02E+08 | -1 | SVOPL |
| ENSBTAG00000042817 | 4 | 1.02E+08 | 1.02E+08 | -1 | Y_RNA |
| ENSBTAG00000004263 | 4 | 1.02E+08 | 1.02E+08 | -1 | ATP6V0A4 |
| ENSBTAG00000001527 | 4 | 1.02E+08 | 1.02E+08 | 1 | TMEM213 |
| ENSBTAG00000021073 | 4 | 1.03E+08 | 1.02E+08 | -1 | KIAA1549 |
| ENSBTAG00000014250 | 4 | 1.03E+08 | 1.03E+08 | -1 | ZC3HAV1L |
| ENSBTAG00000021617 | 4 | 1.03E+08 | 1.03E+08 | -1 | ZC3HAV1 |
| ENSBTAG00000016373 | 4 | 1.03E+08 | 1.03E+08 | 1 | TTC26 |
| ENSBTAG00000014021 | 4 | 1.03E+08 | 1.03E+08 | 1 | UBN2 |
| ENSBTAG00000053406 | 4 | 1.03E+08 | 1.03E+08 | 1 | FMC1 |
| ENSBTAG00000017770 | 4 | 1.03E+08 | 1.03E+08 | 1 | LUC7L2 |
| ENSBTAG00000017793 | 4 | 1.03E+08 | 1.03E+08 | -1 | KLRG2 |
| ENSBTAG00000038854 | 4 | 1.03E+08 | 1.03E+08 | 1 | CLEC2L |
| ENSBTAG00000017860 | 4 | 1.03E+08 | 1.03E+08 | -1 | HIPK2 |
| ENSBTAG00000020225 | 4 | 1.03E+08 | 1.03E+08 | 1 | TBXAS1 |
| ENSBTAG00000048477 | 4 | 1.03E+08 | 1.03E+08 | -1 |  |
| ENSBTAG00000052089 | 4 | 1.03E+08 | 1.03E+08 | -1 | U3 |
| ENSBTAG00000016546 | 4 | 1.04E+08 | 1.03E+08 | -1 | PARP12 |
| ENSBTAG00000003495 | 4 | 1.04E+08 | 1.04E+08 | -1 | KDM7A |
| ENSBTAG00000012239 | 4 | 1.04E+08 | 1.04E+08 | -1 | SLC37A3 |
| ENSBTAG00000012261 | 4 | 1.04E+08 | 1.04E+08 | 1 | RAB19 |
| ENSBTAG00000008306 | 4 | 1.04E+08 | 1.04E+08 | -1 | MKRN1 |
| ENSBTAG00000004799 | 4 | 1.04E+08 | 1.04E+08 | -1 | DENND2A |
| ENSBTAG00000020484 | 4 | 1.04E+08 | 1.04E+08 | 1 | ADCK2 |
| ENSBTAG00000021759 | 4 | 1.04E+08 | 1.04E+08 | 1 | NDUFB2 |
| ENSBTAG00000021761 | 4 | 1.04E+08 | 1.04E+08 | -1 | BRAF |
| ENSBTAG00000052905 | 4 | 1.04E+08 | 1.04E+08 | -1 | 5S_rRNA |
| ENSBTAG00000004976 | 4 | 30904166 | 30692956 | -1 | CDCA7L |
| ENSBTAG00000015539 | 4 | 31143356 | 30909069 | -1 | RAPGEF5 |
| ENSBTAG00000014921 | 4 | 31459131 | 31454749 | 1 | IL6 |
| ENSBTAG00000051150 | 4 | 31527812 | 31520444 | -1 | TOMM7 |
| ENSBTAG00000053905 | 4 | 31554228 | 31551687 | 1 |  |
| ENSBTAG00000043519 | 4 | 31553778 | 31553706 | 1 | SNORD93 |
| ENSBTAG00000033806 | 4 | 31570769 | 31570207 | 1 |  |
| ENSBTAG00000004575 | 4 | 31751438 | 31618071 | -1 | FAM126A |
| ENSBTAG00000019406 | 4 | 32088119 | 31943617 | -1 | IGF2BP3 |
| ENSBTAG00000012622 | 4 | 32135318 | 32116515 | -1 | TRA2A |
| ENSBTAG00000013010 | 4 | 32259874 | 32217367 | 1 | CCDC126 |
| ENSBTAG00000052932 | 4 | 32272807 | 32272205 | 1 |  |
| ENSBTAG00000014711 | 4 | 32315089 | 32287899 | -1 | DBF4 |
| ENSBTAG00000054987 | 4 | 32394916 | 32315217 | 1 | SLC25A40 |
| ENSBTAG00000052947 | 4 | 32419402 | 32400488 | -1 |  |
| ENSBTAG00000015666 | 4 | 32536675 | 32435032 | -1 |  |
| ENSBTAG00000054875 | 4 | 32539062 | 32538061 | -1 |  |
| ENSBTAG00000021313 | 4 | 32731459 | 32564174 | 1 | RUNDC3B |
| ENSBTAG00000033449 | 4 | 32783267 | 32735267 | -1 |  |
| ENSBTAG00000052401 | 4 | 32783234 | 32781463 | -1 |  |
| ENSBTAG00000005997 | 4 | 32982260 | 32880212 | 1 |  |
| ENSBTAG00000048664 | 4 | 8616503 | 7937943 | 1 | CDK14 |
| ENSBTAG00000002107 | 4 | 8670064 | 8665915 | 1 | FZD1 |
| ENSBTAG00000027319 | 4 | 8740694 | 8735637 | 1 |  |
| ENSBTAG00000048337 | 4 | 8811475 | 8769961 | 1 |  |
| ENSBTAG00000052341 | 4 | 9144825 | 9008324 | -1 |  |
| ENSBTAG00000045617 | 4 | 9254862 | 9247873 | -1 | MTERF1 |
| ENSBTAG00000007442 | 4 | 9455572 | 9333128 | 1 | AKAP9 |
| ENSBTAG00000001992 | 4 | 9476713 | 9459764 | -1 | CYP51A1 |
| ENSBTAG00000001571 | 4 | 9504398 | 9488265 | -1 | LRRD1 |
| ENSBTAG00000002750 | 4 | 9567814 | 9528416 | -1 | KRIT1 |
| ENSBTAG00000034936 | 4 | 9725826 | 9624288 | 1 | ANKIB1 |
| ENSBTAG00000051645 | 4 | 9666083 | 9664779 | -1 |  |
| ENSBTAG00000048169 | 4 | 9704457 | 9704065 | -1 |  |
| ENSBTAG00000034905 | 4 | 9765541 | 9735071 | -1 | TMBIM7 |
| ENSBTAG00000030095 | 4 | 9736948 | 9736854 | -1 | bta-mir-584-5 |
| ENSBTAG00000021996 | 4 | 9772218 | 9764439 | 1 | GATAD1 |
| ENSBTAG00000024431 | 4 | 9870626 | 9789227 | -1 | PEX1 |
| ENSBTAG00000018363 | 4 | 9877234 | 9870503 | 1 | RBM48 |
| ENSBTAG00000003810 | 4 | 9895506 | 9877592 | -1 |  |
| ENSBTAG00000050705 | 4 | 9926242 | 9909855 | -1 | FAM133B |
| ENSBTAG00000044023 | 4 | 10188536 | 9939646 | -1 | CDK6 |
| ENSBTAG00000051416 | 4 | 10206518 | 10203048 | 1 |  |
| ENSBTAG00000042409 | 4 | 10414194 | 10414083 | -1 | U6 |
| ENSBTAG00000053050 | 4 | 10468917 | 10449812 | -1 | SAMD9 |
| ENSBTAG00000012456 | 4 | 10595196 | 10541020 | -1 | HEPACAM2 |
| ENSBTAG00000014255 | 4 | 10732873 | 10600800 | 1 | VPS50 |
| ENSBTAG00000017458 | 4 | 10893236 | 10781954 | -1 | CALCR |
| ENSBTAG00000036366 | 4 | 10822290 | 10822201 | -1 | bta-mir-653 |
| ENSBTAG00000044929 | 4 | 10822951 | 10822870 | -1 | bta-mir-489 |
| ENSBTAG00000053622 | 4 | 10884030 | 10883955 | 1 | bta-mir-378-2 |
| ENSBTAG00000015844 | 4 | 11208760 | 11204075 | -1 | TFPI2 |
| ENSBTAG00000002674 | 4 | 11235219 | 11210209 | 1 | GNGT1 |
| ENSBTAG00000000820 | 4 | 11247552 | 11242533 | 1 | GNG11 |
| ENSBTAG00000003424 | 4 | 11345143 | 11330484 | -1 | BET1 |
| ENSBTAG00000013472 | 4 | 11812997 | 11776554 | 1 | COL1A2 |
| ENSBTAG00000009109 | 4 | 11939978 | 11886419 | 1 | CASD1 |
| ENSBTAG00000021282 | 4 | 12063687 | 11992415 | -1 | SGCE |
| ENSBTAG00000053735 | 4 | 12076656 | 12063954 | 1 | PEG10 |
| ENSBTAG00000043945 | 4 | 12093745 | 12093642 | 1 | U6 |
| ENSBTAG00000024426 | 4 | 12534305 | 12191129 | 1 | PPP1R9A |
| ENSBTAG00000015654 | 4 | 12576328 | 12542354 | -1 | PON1 |
| ENSBTAG00000034645 | 4 | 12631421 | 12593909 | -1 | PON3 |
| ENSBTAG00000008361 | 4 | 12673193 | 12645430 | -1 | PON2 |
| ENSBTAG00000018185 | 4 | 12843879 | 12750329 | 1 | ASB4 |
| ENSBTAG00000047555 | 4 | 12853294 | 12852914 | 1 |  |
| ENSBTAG00000014069 | 4 | 12895362 | 12881889 | -1 | PDK4 |
| ENSBTAG00000045214 | 4 | 12925914 | 12925810 | 1 | U6 |
| ENSBTAG00000027134 | 4 | 13440805 | 13061840 | 1 | DYNC1I1 |
| ENSBTAG00000003773 | 4 | 13692192 | 13462646 | -1 | SLC25A13 |
| ENSBTAG00000044544 | 4 | 13670098 | 13669967 | 1 | SNORA70 |
| ENSBTAG00000035660 | 4 | 13707455 | 13707135 | 1 |  |
| ENSBTAG00000052508 | 4 | 14079935 | 14054960 | -1 | SEM1 |
| ENSBTAG00000020685 | 4 | 14117046 | 14114994 | 1 |  |
| ENSBTAG00000051143 | 4 | 14350592 | 14350413 | 1 |  |
| ENSBTAG00000053796 | 4 | 14351035 | 14350830 | 1 |  |
| ENSBTAG00000053173 | 4 | 14388049 | 14386701 | -1 |  |
| ENSBTAG00000021458 | 4 | 14394726 | 14390022 | 1 | DLX6 |
| ENSBTAG00000054386 | 4 | 14397713 | 14397572 | -1 |  |
| ENSBTAG00000005653 | 4 | 33080056 | 33006669 | 1 | ABCB4 |
| ENSBTAG00000021535 | 4 | 33144192 | 33083035 | -1 | CROT |
| ENSBTAG00000049907 | 4 | 33144705 | 33144607 | -1 |  |
| ENSBTAG00000054504 | 4 | 33144957 | 33144801 | -1 |  |
| ENSBTAG00000035379 | 4 | 33213237 | 33212389 | 1 |  |
| ENSBTAG00000038283 | 4 | 33248624 | 33226967 | 1 | TMEM243 |
| ENSBTAG00000020878 | 4 | 33286857 | 33249637 | -1 | DMTF1 |
| ENSBTAG00000053479 | 4 | 33258782 | 33257813 | -1 |  |
| ENSBTAG00000049818 | 4 | 33263192 | 33263022 | -1 |  |
| ENSBTAG00000019276 | 4 | 33388681 | 33387397 | 1 |  |
| ENSBTAG00000004023 | 4 | 33619877 | 33398732 | 1 | ELAPOR2 |
| ENSBTAG00000018989 | 4 | 33877961 | 33626562 | -1 | GRM3 |
| ENSBTAG00000050375 | 4 | 34689864 | 34689758 | 1 | U6 |
| ENSBTAG00000024394 | 4 | 35616774 | 35390232 | 1 | SEMA3D |
| ENSBTAG00000043038 | 4 | 35512390 | 35512286 | -1 | U6 |
| ENSBTAG00000018133 | 4 | 36631147 | 36254736 | 1 | SEMA3A |
| ENSBTAG00000053333 | 4 | 36711110 | 36705851 | 1 |  |
| ENSBTAG00000014920 | 4 | 37237011 | 36966817 | 1 | SEMA3E |
| ENSBTAG00000020569 | 4 | 38673177 | 38152730 | 1 | CACNA2D1 |
| ENSBTAG00000043671 | 4 | 38303062 | 38302740 | 1 | 7SK |
| ENSBTAG00000017664 | 4 | 39010999 | 38929386 | 1 | HGF |
| ENSBTAG00000055213 | 4 | 39326200 | 39325997 | 1 |  |
| ENSBTAG00000044967 | 4 | 39552169 | 39552074 | 1 | U6 |
| ENSBTAG00000006138 | 4 | 40145312 | 39939436 | 1 | SEMA3C |
| ENSBTAG00000047646 | 4 | 40262214 | 40232708 | -1 |  |
| ENSBTAG00000054922 | 4 | 40291133 | 40288475 | -1 |  |
| ENSBTAG00000014220 | 4 | 40360899 | 40319707 | -1 |  |
| ENSBTAG00000017866 | 4 | 40443336 | 40380301 | -1 | CD36 |
| ENSBTAG00000017866 | 4 | 40443336 | 40380301 | -1 | CD36 |
| ENSBTAG00000017866 | 4 | 40443336 | 40380301 | -1 | CD36 |
| ENSBTAG00000049549 | 4 | 40475971 | 40473212 | -1 |  |
| ENSBTAG00000008641 | 4 | 40629320 | 40574285 | 1 | GNAT3 |
| ENSBTAG00000029292 | 4 | 40685829 | 40685712 | -1 | 5S_rRNA |
| ENSBTAG00000047294 | 4 | 40864160 | 40861857 | 1 |  |
| ENSBTAG00000002714 | 4 | 41062151 | 40956253 | -1 | GNAI1 |
| ENSBTAG00000044831 | 4 | 41566762 | 41566637 | 1 | 5S_rRNA |
| ENSBTAG00000053714 | 4 | 41794763 | 41775571 | -1 |  |
| ENSBTAG00000053328 | 4 | 42293599 | 42292576 | 1 |  |
| ENSBTAG00000051262 | 4 | 43002303 | 42761355 | 1 |  |
| ENSBTAG00000022498 | 4 | 43148895 | 43148370 | -1 |  |
| ENSBTAG00000005102 | 4 | 43449891 | 43312172 | -1 | PHTF2 |
| ENSBTAG00000033290 | 4 | 43454105 | 43450254 | 1 | TMEM60 |
| ENSBTAG00000015753 | 4 | 43548241 | 43464283 | -1 | RSBN1L |
| ENSBTAG00000052433 | 4 | 43490674 | 43488782 | 1 |  |
| ENSBTAG00000043602 | 4 | 43562734 | 43562420 | 1 | 7SK |
| ENSBTAG00000048468 | 4 | 43571609 | 43571206 | 1 |  |
| ENSBTAG00000043904 | 4 | 43585651 | 43585507 | 1 |  |
| ENSBTAG00000003825 | 4 | 43690863 | 43598679 | -1 | PTPN12 |
| ENSBTAG00000033269 | 4 | 43752487 | 43752324 | -1 | U1 |
| ENSBTAG00000015461 | 4 | 43758983 | 43758669 | -1 |  |
| ENSBTAG00000053154 | 4 | 43767198 | 43764414 | 1 |  |
| ENSBTAG00000018430 | 4 | 43913447 | 43815426 | 1 | GSAP |
| ENSBTAG00000054348 | 4 | 43853697 | 43852384 | -1 |  |
| ENSBTAG00000009341 | 4 | 44057818 | 43916446 | -1 | CCDC146 |
| ENSBTAG00000009717 | 4 | 44004848 | 44000211 | 1 | FGL2 |
| ENSBTAG00000044264 | 4 | 44016018 | 44015903 | 1 | 5S_rRNA |
| ENSBTAG00000014879 | 4 | 44133423 | 44057955 | 1 | FAM185A |
| ENSBTAG00000016324 | 4 | 44363428 | 44146960 | -1 | FBXL13 |
| ENSBTAG00000024379 | 4 | 44279731 | 44155991 | 1 | LRRC17 |
| ENSBTAG00000014161 | 4 | 44428499 | 44363508 | 1 | ARMC10 |
| ENSBTAG00000014171 | 4 | 44517650 | 44427825 | -1 | NAPEPLD |
| ENSBTAG00000048633 | 4 | 44457869 | 44457763 | 1 | U6 |
| ENSBTAG00000006876 | 4 | 44531440 | 44516585 | 1 | PMPCB |
| ENSBTAG00000003784 | 4 | 44565511 | 44531524 | -1 | DNAJC2 |
| ENSBTAG00000046712 | 4 | 44582371 | 44569083 | 1 | PSMC2 |
| ENSBTAG00000009296 | 4 | 44624862 | 44588731 | -1 | SLC26A5 |
| ENSBTAG00000003658 | 4 | 45211015 | 44653832 | -1 | RELN |
| ENSBTAG00000052629 | 4 | 45161520 | 45156056 | -1 |  |
| ENSBTAG00000040058 | 4 | 45418109 | 45330008 | -1 | ORC5 |
| ENSBTAG00000000189 | 4 | 46117403 | 45537952 | 1 | LHFPL3 |
| ENSBTAG00000048818 | 4 | 45979959 | 45576207 | -1 |  |
| ENSBTAG00000009207 | 4 | 46292635 | 46202241 | 1 | KMT2E |
| ENSBTAG00000002175 | 4 | 46536298 | 46296970 | -1 | SRPK2 |
| ENSBTAG00000047286 | 4 | 46340362 | 46337363 | -1 |  |
| ENSBTAG00000053913 | 4 | 46384898 | 46384653 | 1 |  |
| ENSBTAG00000007743 | 4 | 46640231 | 46586617 | -1 | PUS7 |
| ENSBTAG00000008302 | 4 | 46670543 | 46646922 | 1 | RINT1 |
| ENSBTAG00000053355 | 4 | 46685149 | 46670649 | -1 | EFCAB10 |
| ENSBTAG00000002037 | 4 | 46757682 | 46703819 | -1 | ATXN7L1 |
| ENSBTAG00000049733 | 4 | 46955175 | 46833351 | -1 | ATXN7L1 |
| ENSBTAG00000021365 | 4 | 47099902 | 47031326 | 1 | CDHR3 |
| ENSBTAG00000044548 | 4 | 47094286 | 47094216 | -1 | bta-mir-2284b |
| ENSBTAG00000019794 | 4 | 47180223 | 47151949 | -1 | SYPL1 |
| ENSBTAG00000042539 | 4 | 47209960 | 47209854 | -1 | U6 |
| ENSBTAG00000019824 | 4 | 47235256 | 47234827 | -1 |  |
| ENSBTAG00000015509 | 4 | 47361906 | 47321231 | -1 | NAMPT |
| ENSBTAG00000017541 | 4 | 61832723 | 61789173 | 1 | TBX20 |
| ENSBTAG00000032650 | 4 | 61940801 | 61850902 | 1 | DPY19L2 |
| ENSBTAG00000005785 | 4 | 62068881 | 61976304 | 1 | DPY19L1 |
| ENSBTAG00000042960 | 4 | 62083600 | 62083270 | 1 | 7SK |
| ENSBTAG00000013768 | 4 | 62274639 | 62120709 | -1 | NPSR1 |
| ENSBTAG00000050014 | 4 | 62144295 | 62144189 | -1 | U6 |
| ENSBTAG00000053990 | 4 | 62601967 | 62600534 | 1 |  |
| ENSBTAG00000010866 | 4 | 62917005 | 62664288 | -1 | BMPER |
| ENSBTAG00000051549 | 4 | 62797117 | 62797060 | -1 | bta-mir-2285bu-2 |
| ENSBTAG00000044585 | 4 | 63059273 | 63059199 | 1 | bta-mir-1814c |
| ENSBTAG00000006528 | 4 | 63708251 | 63255358 | -1 | BBS9 |
| ENSBTAG00000051837 | 4 | 63310268 | 63310162 | 1 | U6 |
| ENSBTAG00000044971 | 4 | 63624249 | 63624165 | -1 |  |
| ENSBTAG00000055285 | 4 | 63732922 | 63730458 | 1 |  |
| ENSBTAG00000032598 | 4 | 63746286 | 63733826 | 1 | RP9 |
| ENSBTAG00000052846 | 4 | 63768770 | 63764945 | 1 |  |
| ENSBTAG00000016709 | 4 | 63807500 | 63788270 | 1 | NT5C3A |
| ENSBTAG00000016707 | 4 | 63854573 | 63812248 | -1 | FKBP9 |
| ENSBTAG00000003115 | 4 | 63925721 | 63912710 | 1 | KBTBD2 |
| ENSBTAG00000003114 | 4 | 64010228 | 63956366 | -1 | AVL9 |
| ENSBTAG00000002332 | 4 | 64018569 | 64014862 | 1 | LSM5 |
| ENSBTAG00000002739 | 4 | 64715670 | 64554173 | 1 | PDE1C |
| ENSBTAG00000001976 | 4 | 64779923 | 64759541 | -1 | PPP1R17 |
| ENSBTAG00000009254 | 4 | 64906352 | 64817186 | -1 | ITPRID1 |
| ENSBTAG00000000558 | 4 | 65093899 | 65090702 | 1 | NEUROD6 |
| ENSBTAG00000020247 | 4 | 65377107 | 65266775 | -1 | ADCYAP1R1 |
| ENSBTAG00000047599 | 4 | 65411689 | 65395787 | -1 | GHRHR |
| ENSBTAG00000000745 | 4 | 65447871 | 65433649 | -1 | AQP1 |
| ENSBTAG00000009390 | 4 | 65585947 | 65467521 | -1 | MINDY4 |
| ENSBTAG00000001448 | 4 | 65608037 | 65603906 | 1 | INMT |
| ENSBTAG00000014381 | 4 | 65705053 | 65647996 | 1 | CRHR2 |
| ENSBTAG00000018972 | 4 | 65758270 | 65704797 | -1 | GARS1 |
| ENSBTAG00000051818 | 4 | 65805893 | 65785076 | 1 |  |
| ENSBTAG00000010597 | 4 | 65816128 | 65806682 | 1 | GGCT |
| ENSBTAG00000038235 | 4 | 65911847 | 65822126 | 1 | NOD1 |
| ENSBTAG00000045223 | 4 | 65945945 | 65945870 | -1 | bta-mir-2419 |
| ENSBTAG00000000260 | 4 | 66053326 | 65961977 | -1 | ZNRF2 |
| ENSBTAG00000052317 | 4 | 66214450 | 66186175 | -1 | MTURN |
| ENSBTAG00000000521 | 4 | 66328519 | 66266959 | -1 | PLEKHA8 |
| ENSBTAG00000007870 | 4 | 66340716 | 66328677 | 1 | FKBP14 |
| ENSBTAG00000016223 | 4 | 66421169 | 66355254 | 1 | SCRN1 |
| ENSBTAG00000016220 | 4 | 66443623 | 66423128 | -1 | WIPF3 |
| ENSBTAG00000045567 | 4 | 66610438 | 66608858 | -1 | PRR15 |
| ENSBTAG00000020931 | 4 | 66970909 | 66634935 | -1 | CHN2 |
| ENSBTAG00000054573 | 4 | 67002936 | 67001833 | 1 |  |
| ENSBTAG00000007146 | 4 | 67157458 | 67029848 | 1 | CPVL |
| ENSBTAG00000024340 | 4 | 67207092 | 67204660 | 1 | TRIL |
| ENSBTAG00000018909 | 4 | 67752296 | 67314290 | -1 | CREB5 |
| ENSBTAG00000044617 | 4 | 67468840 | 67468735 | -1 | 5S_rRNA |
| ENSBTAG00000019024 | 4 | 68321145 | 67992971 | 1 | JAZF1 |
| ENSBTAG00000019020 | 4 | 68407181 | 68323940 | -1 | TAX1BP1 |
| ENSBTAG00000001036 | 4 | 68614954 | 68473794 | 1 | HIBADH |
| ENSBTAG00000043287 | 4 | 68490521 | 68490451 | 1 |  |
| ENSBTAG00000020919 | 4 | 68801018 | 68797645 | -1 | EVX1 |
| ENSBTAG00000053691 | 4 | 68836391 | 68836226 | -1 |  |
| ENSBTAG00000048820 | 4 | 68840222 | 68839861 | -1 |  |
| ENSBTAG00000055166 | 4 | 68840692 | 68840319 | -1 |  |
| ENSBTAG00000053894 | 4 | 68842077 | 68842022 | -1 |  |
| ENSBTAG00000014735 | 4 | 68844385 | 68842500 | 1 | HOXA13 |
| ENSBTAG00000054389 | 4 | 68853862 | 68853684 | -1 |  |
| ENSBTAG00000051476 | 4 | 68854828 | 68854619 | -1 |  |
| ENSBTAG00000054854 | 4 | 68855753 | 68855523 | -1 |  |
| ENSBTAG00000051971 | 4 | 68856437 | 68856340 | -1 |  |
| ENSBTAG00000051125 | 4 | 68856766 | 68856581 | -1 |  |
| ENSBTAG00000048773 | 4 | 68857151 | 68857056 | -1 |  |
| ENSBTAG00000014738 | 4 | 68861055 | 68857431 | 1 | HOXA11 |
| ENSBTAG00000040082 | 4 | 68872086 | 68862439 | 1 | HOXA10 |
| ENSBTAG00000048562 | 4 | 68869395 | 68868473 | 1 |  |
| ENSBTAG00000029970 | 4 | 68873238 | 68873154 | 1 | bta-mir-196b |
| ENSBTAG00000053595 | 4 | 68880322 | 68877182 | 1 | HOXA9 |
| ENSBTAG00000001455 | 4 | 68887923 | 68885186 | 1 | HOXA7 |
| ENSBTAG00000024341 | 4 | 68898550 | 68894951 | 1 | HOXA6 |
| ENSBTAG00000012211 | 4 | 68901681 | 68898726 | 1 | HOXA5 |
| ENSBTAG00000008139 | 4 | 68934981 | 68902556 | 1 | HOXA3 |
| ENSBTAG00000001063 | 4 | 68914205 | 68911991 | 1 | HOXA4 |
| ENSBTAG00000008138 | 4 | 68941279 | 68939317 | 1 | HOXA2 |
| ENSBTAG00000052264 | 4 | 68942034 | 68941892 | -1 |  |
| ENSBTAG00000050085 | 4 | 68942145 | 68942044 | -1 |  |
| ENSBTAG00000048509 | 4 | 68942222 | 68942165 | -1 |  |
| ENSBTAG00000049679 | 4 | 68943112 | 68942896 | -1 |  |
| ENSBTAG00000052517 | 4 | 68945863 | 68945736 | -1 |  |
| ENSBTAG00000013263 | 4 | 68948264 | 68945977 | 1 | HOXA1 |
| ENSBTAG00000047863 | 4 | 69003498 | 69002744 | -1 |  |
| ENSBTAG00000005650 | 4 | 69297030 | 69125496 | 1 | SKAP2 |
| ENSBTAG00000043804 | 4 | 69383863 | 69383582 | -1 | 7SK |
| ENSBTAG00000001822 | 4 | 69655430 | 69587812 | -1 | SNX10 |
| ENSBTAG00000049437 | 4 | 69734069 | 69722997 | -1 | CBX3 |
| ENSBTAG00000005726 | 4 | 69744610 | 69735135 | 1 | HNRNPA2B1 |
| ENSBTAG00000004136 | 4 | 69782790 | 69748359 | -1 | NFE2L3 |
| ENSBTAG00000029945 | 4 | 69949550 | 69949466 | 1 | MIR148A |
| ENSBTAG00000043517 | 4 | 70119089 | 70118983 | -1 | U6 |
| ENSBTAG00000050039 | 4 | 70162251 | 70153381 | -1 |  |
| ENSBTAG00000019447 | 4 | 70670595 | 70667240 | 1 | NPVF |
| ENSBTAG00000019445 | 4 | 70727802 | 70699194 | 1 | C4H7orf31 |
| ENSBTAG00000022613 | 4 | 70738238 | 70735436 | 1 | CYCS |
| ENSBTAG00000019342 | 4 | 71041101 | 70844945 | 1 | OSBPL3 |
| ENSBTAG00000043109 | 4 | 70963845 | 70963720 | 1 |  |
| ENSBTAG00000046586 | 4 | 71063807 | 71063232 | 1 |  |
| ENSBTAG00000015326 | 4 | 71192446 | 71088326 | 1 | GSDME |
| ENSBTAG00000015303 | 4 | 71303589 | 71173805 | -1 | PALS2 |
| ENSBTAG00000004503 | 4 | 71623983 | 71616842 | -1 | NPY |
| ENSBTAG00000010609 | 4 | 72031056 | 71959160 | -1 | STK31 |
| ENSBTAG00000018064 | 4 | 72059949 | 72036169 | -1 | FAM221A |
| ENSBTAG00000007136 | 4 | 72308387 | 72076430 | 1 | ADAM22 |
| ENSBTAG00000024334 | 4 | 72118502 | 72118023 | -1 |  |
| ENSBTAG00000010390 | 4 | 72342353 | 72319442 | -1 | SRI |
| ENSBTAG00000002340 | 4 | 72407341 | 72378038 | -1 | STEAP4 |
| ENSBTAG00000052069 | 4 | 72487900 | 72487794 | 1 | U6 |
| ENSBTAG00000050357 | 4 | 72862341 | 72859093 | 1 |  |
| ENSBTAG00000032393 | 4 | 72902435 | 72900781 | -1 | TEX47 |
| ENSBTAG00000042914 | 4 | 73110941 | 73110833 | -1 | U6 |
| ENSBTAG00000046430 | 4 | 73427125 | 73324012 | 1 | ZNF804B |
| ENSBTAG00000051144 | 4 | 73576575 | 73576457 | -1 | 5S_rRNA |
| ENSBTAG00000015749 | 4 | 74368429 | 74355342 | 1 | STEAP1 |
| ENSBTAG00000003506 | 4 | 74430946 | 74405139 | 1 | STEAP2 |
| ENSBTAG00000003508 | 4 | 74502981 | 74443217 | 1 | CFAP69 |
| ENSBTAG00000052637 | 4 | 74512770 | 74512360 | -1 | FAM237B |
| ENSBTAG00000032166 | 4 | 74559891 | 74533033 | 1 | GTPBP10 |
| ENSBTAG00000013226 | 4 | 74587244 | 74573944 | 1 | HUS1 |
| ENSBTAG00000044309 | 4 | 74773235 | 74773098 | -1 |  |
| ENSBTAG00000009655 | 4 | 74996283 | 74779686 | 1 | TNS3 |
| ENSBTAG00000048877 | 4 | 75161472 | 75151604 | -1 |  |
| ENSBTAG00000051057 | 4 | 75188292 | 75186128 | 1 |  |
| ENSBTAG00000044867 | 4 | 76017538 | 76017461 | -1 | bta-mir-2420 |
| ENSBTAG00000003994 | 4 | 76124835 | 76116349 | 1 | IGFBP3 |
| ENSBTAG00000046768 | 4 | 76136846 | 76131287 | -1 | IGFBP1 |
| ENSBTAG00000053893 | 4 | 76157434 | 76154665 | 1 |  |
| ENSBTAG00000009520 | 4 | 76273756 | 76174089 | -1 | ADCY1 |
| ENSBTAG00000020704 | 4 | 76493685 | 76444976 | -1 | RAMP3 |
| ENSBTAG00000046334 | 4 | 76513580 | 76511048 | 1 |  |
| ENSBTAG00000010504 | 4 | 76531904 | 76522744 | 1 | TBRG4 |
| ENSBTAG00000043398 | 4 | 76527322 | 76527187 | 1 | SNORA5C |
| ENSBTAG00000043541 | 4 | 76527844 | 76527710 | 1 | SNORA5A |
| ENSBTAG00000051248 | 4 | 76548692 | 76537712 | 1 |  |
| ENSBTAG00000008090 | 4 | 76603468 | 76550183 | -1 | CCM2 |
| ENSBTAG00000052466 | 4 | 76614443 | 76611350 | 1 |  |
| ENSBTAG00000042440 | 4 | 76612390 | 76612259 | 1 | SNORA9 |
| ENSBTAG00000006377 | 4 | 76633185 | 76617184 | 1 | MYO1G |
| ENSBTAG00000048806 | 4 | 76653725 | 76653619 | -1 | U6 |
| ENSBTAG00000052945 | 4 | 76669082 | 76665960 | 1 | PURB |
| ENSBTAG00000046529 | 4 | 76669579 | 76669524 | 1 | bta-mir-4657 |
| ENSBTAG00000016975 | 4 | 76701169 | 76689344 | 1 | H2AZ2 |
| ENSBTAG00000012003 | 4 | 76713230 | 76709520 | -1 | PPIA |
| ENSBTAG00000011997 | 4 | 76748161 | 76730454 | -1 | ZMIZ2 |
| ENSBTAG00000006029 | 4 | 76844759 | 76758582 | -1 | OGDH |
| ENSBTAG00000010612 | 4 | 76862453 | 76859613 | 1 | TMED4 |
| ENSBTAG00000010602 | 4 | 76874946 | 76865589 | 1 | DDX56 |
| ENSBTAG00000044146 | 4 | 76912254 | 76889504 | 1 | NPC1L1 |
| ENSBTAG00000006325 | 4 | 76988105 | 76917098 | 1 | NUDCD3 |
| ENSBTAG00000012653 | 4 | 77117686 | 77025265 | 1 | CAMK2B |
| ENSBTAG00000000274 | 4 | 77129830 | 77120960 | -1 | YKT6 |
| ENSBTAG00000032288 | 4 | 77175906 | 77139555 | 1 | GCK |
| ENSBTAG00000002066 | 4 | 77181080 | 77178578 | 1 | MYL7 |
| ENSBTAG00000012241 | 4 | 77200482 | 77190290 | 1 | POLD2 |
| ENSBTAG00000012237 | 4 | 77209981 | 77200840 | -1 | AEBP1 |
| ENSBTAG00000049936 | 4 | 77215166 | 77210122 | -1 |  |
| ENSBTAG00000007796 | 4 | 77236566 | 77228350 | 1 | POLM |
| ENSBTAG00000053416 | 4 | 77267194 | 77267088 | -1 | U6 |
| ENSBTAG00000013414 | 4 | 77335575 | 77284046 | -1 | BLVRA |
| ENSBTAG00000012968 | 4 | 77465906 | 77371216 | 1 | COA1 |
| ENSBTAG00000008143 | 4 | 77515252 | 77478669 | -1 | STK17A |
| ENSBTAG00000021216 | 4 | 77804239 | 77539351 | -1 | HECW1 |
| ENSBTAG00000000991 | 4 | 78126851 | 78121755 | -1 | MRPL32 |
| ENSBTAG00000000990 | 4 | 78137712 | 78127061 | 1 | PSMA2 |
| ENSBTAG00000009472 | 4 | 78147222 | 78136885 | 1 | C4H7orf25 |
| ENSBTAG00000054524 | 4 | 78368350 | 78345465 | 1 |  |
| ENSBTAG00000052436 | 4 | 78743839 | 78733138 | -1 |  |
| ENSBTAG00000010671 | 4 | 79063150 | 78761983 | 1 | GLI3 |
| ENSBTAG00000050936 | 4 | 78857029 | 78856976 | 1 |  |
| ENSBTAG00000051960 | 4 | 79219655 | 79150090 | -1 |  |
| ENSBTAG00000051408 | 4 | 79285525 | 79279928 | 1 |  |
| ENSBTAG00000048508 | 4 | 79302445 | 79288772 | 1 | INHBA |
| ENSBTAG00000049628 | 4 | 79289953 | 79289904 | -1 | bta-mir-11996 |
| ENSBTAG00000053253 | 4 | 79873179 | 79872130 | 1 |  |
| ENSBTAG00000032121 | 4 | 80928936 | 80159127 | -1 | SUGCT |
| ENSBTAG00000018715 | 4 | 80931409 | 80929262 | 1 | MPLKIP |
| ENSBTAG00000001528 | 4 | 81075991 | 80952446 | -1 | CDK13 |
| ENSBTAG00000049322 | 4 | 81106637 | 81104811 | 1 |  |
| ENSBTAG00000054825 | 4 | 81177099 | 81171526 | -1 |  |
| ENSBTAG00000006661 | 4 | 81244481 | 81187581 | -1 | RALA |
| ENSBTAG00000001892 | 4 | 81291402 | 81286079 | -1 | YAE1 |
| ENSBTAG00000013648 | 4 | 81759991 | 81370877 | -1 | POU6F2 |
| ENSBTAG00000042795 | 4 | 81939393 | 81939266 | 1 |  |
| ENSBTAG00000007305 | 4 | 82156974 | 81966837 | 1 | VPS41 |
| ENSBTAG00000031967 | 4 | 82507550 | 82295679 | 1 | AMPH |
| ENSBTAG00000000144 | 4 | 82573389 | 82533901 | 1 |  |
| ENSBTAG00000053474 | 4 | 82641187 | 82638575 | 1 |  |
| ENSBTAG00000055254 | 4 | 82710398 | 82692176 | 1 |  |
| ENSBTAG00000002859 | 4 | 82744666 | 82720173 | 1 |  |
| ENSBTAG00000049399 | 4 | 82745786 | 82745643 | 1 |  |
| ENSBTAG00000012649 | 4 | 82793813 | 82786342 | 1 | LSM8 |
| ENSBTAG00000036476 | 4 | 83846882 | 83846765 | 1 | 5S_rRNA |
| ENSBTAG00000006152 | 4 | 83911797 | 83911360 | -1 |  |
| ENSBTAG00000031464 | 4 | 83946737 | 83946063 | 1 |  |
| ENSBTAG00000032077 | 4 | 85251322 | 84688361 | 1 | KCND2 |
| ENSBTAG00000015337 | 4 | 85355349 | 85288736 | -1 | TSPAN12 |
| ENSBTAG00000052759 | 4 | 85380893 | 85374622 | 1 |  |
| ENSBTAG00000016332 | 4 | 85480394 | 85453414 | 1 | ING3 |
| ENSBTAG00000002938 | 4 | 85814161 | 85491227 | 1 | CPED1 |
| ENSBTAG00000002940 | 4 | 85850836 | 85839505 | 1 | WNT16 |
| ENSBTAG00000007976 | 4 | 85918137 | 85865619 | -1 | FAM3C |
| ENSBTAG00000044925 | 4 | 86166290 | 86165950 | 1 | 7SK |
| ENSBTAG00000012119 | 4 | 86510240 | 86301179 | 1 | PTPRZ1 |
| ENSBTAG00000012128 | 4 | 86594679 | 86516905 | -1 | AASS |
| ENSBTAG00000013315 | 5 | 56795018 | 56789495 | 1 | ATP5F1B |
| ENSBTAG00000043048 | 5 | 56790575 | 56790501 | 1 | SNORD59A |
| ENSBTAG00000042677 | 5 | 56791646 | 56791576 | 1 | bta-mir-677 |
| ENSBTAG00000049930 | 5 | 56791642 | 56791577 | 1 | bta-mir-677 |
| ENSBTAG00000017840 | 5 | 56831748 | 56796775 | 1 | BAZ2A |
| ENSBTAG00000017830 | 5 | 56890516 | 56836859 | -1 | RBMS2 |
| ENSBTAG00000009281 | 5 | 56925355 | 56893433 | -1 | SPRYD4 |
| ENSBTAG00000009284 | 5 | 56923321 | 56910794 | 1 | GLS2 |
| ENSBTAG00000010127 | 5 | 56938031 | 56934066 | 1 | MIP |
| ENSBTAG00000020459 | 5 | 56964713 | 56937139 | 1 | TIMELESS |
| ENSBTAG00000004384 | 5 | 56994574 | 56979557 | 1 | APOF |
| ENSBTAG00000045969 | 5 | 56980940 | 56979557 | 1 | APON |
| ENSBTAG00000004380 | 5 | 57009477 | 56997911 | 1 | STAT2 |
| ENSBTAG00000004378 | 5 | 57011422 | 57009388 | -1 | IL23A |
| ENSBTAG00000044879 | 5 | 57013835 | 57013765 | -1 | bta-mir-2432 |
| ENSBTAG00000004376 | 5 | 57028863 | 57015019 | 1 | PAN2 |
| ENSBTAG00000004374 | 5 | 57034101 | 57031093 | 1 | CNPY2 |
| ENSBTAG00000052591 | 5 | 57031292 | 57031227 | 1 | bta-mir-12054 |
| ENSBTAG00000004371 | 5 | 57065001 | 57042171 | 1 | CS |
| ENSBTAG00000004367 | 5 | 57069211 | 57065720 | -1 | COQ10A |
| ENSBTAG00000011762 | 5 | 57086731 | 57074995 | 1 | ANKRD52 |
| ENSBTAG00000044339 | 5 | 57075682 | 57075606 | -1 | bta-mir-2433 |
| ENSBTAG00000052361 | 5 | 57091294 | 57090764 | 1 |  |
| ENSBTAG00000002799 | 5 | 57097226 | 57091706 | -1 | SLC39A5 |
| ENSBTAG00000002798 | 5 | 57103340 | 57097196 | -1 | NABP2 |
| ENSBTAG00000026754 | 5 | 57130431 | 57103613 | 1 | RNF41 |
| ENSBTAG00000014697 | 5 | 57161786 | 57140987 | 1 | SMARCC2 |
| ENSBTAG00000010799 | 5 | 57164985 | 57161869 | -1 | MYL6 |
| ENSBTAG00000031217 | 5 | 57168025 | 57165321 | -1 | MYL6B |
| ENSBTAG00000009543 | 5 | 57194004 | 57179946 | -1 | ESYT1 |
| ENSBTAG00000009542 | 5 | 57205232 | 57197914 | -1 | ZC3H10 |
| ENSBTAG00000010451 | 5 | 57214801 | 57208094 | -1 | PA2G4 |
| ENSBTAG00000010444 | 5 | 57236737 | 57215784 | -1 | ERBB3 |
| ENSBTAG00000038896 | 5 | 57283196 | 57279798 | -1 | RPS26 |
| ENSBTAG00000012636 | 5 | 57302803 | 57292528 | -1 | IKZF4 |
| ENSBTAG00000006160 | 5 | 57319225 | 57314953 | -1 | SUOX |
| ENSBTAG00000014129 | 5 | 57337350 | 57321765 | -1 | RAB5B |
| ENSBTAG00000004021 | 5 | 57344446 | 57339030 | -1 | CDK2 |
| ENSBTAG00000004019 | 5 | 57353330 | 57343063 | 1 | PMEL |
| ENSBTAG00000004018 | 5 | 57376105 | 57353452 | -1 | DGKA |
| ENSBTAG00000031146 | 5 | 57399551 | 57378226 | 1 | PYM1 |
| ENSBTAG00000009051 | 5 | 57433048 | 57426768 | 1 | MMP19 |
| ENSBTAG00000009049 | 5 | 57437847 | 57433437 | -1 | TMEM198B |
| ENSBTAG00000020664 | 5 | 57446182 | 57438695 | 1 | DNAJC14 |
| ENSBTAG00000020664 | 5 | 57446182 | 57438695 | 1 | DNAJC14 |
| ENSBTAG00000020663 | 5 | 57449119 | 57446157 | -1 | ORMDL2 |
| ENSBTAG00000020662 | 5 | 57498922 | 57449443 | 1 | SARNP |
| ENSBTAG00000007417 | 5 | 57512983 | 57506100 | -1 | GDF11 |
| ENSBTAG00000051365 | 5 | 57525614 | 57517950 | 1 |  |
| ENSBTAG00000011931 | 5 | 57532768 | 57528943 | 1 | CD63 |
| ENSBTAG00000011927 | 5 | 57537461 | 57533663 | -1 | RDH5 |
| ENSBTAG00000011918 | 5 | 57541849 | 57538198 | -1 | BLOC1S1 |
| ENSBTAG00000012897 | 5 | 57572012 | 57548839 | 1 | ITGA7 |
| ENSBTAG00000012896 | 5 | 57575448 | 57572503 | -1 | METTL7B |
| ENSBTAG00000006605 | 5 | 57609680 | 57608661 | 1 | OR10P25 |
| ENSBTAG00000044490 | 5 | 57611609 | 57611456 | -1 |  |
| ENSBTAG00000031031 | 5 | 57627453 | 57626515 | -1 | OR10P1 |
| ENSBTAG00000051656 | 5 | 57642664 | 57641621 | -1 | OR6C63 |
| ENSBTAG00000051585 | 5 | 57662900 | 57661971 | -1 | OR6C304 |
| ENSBTAG00000052738 | 5 | 57674653 | 57673709 | -1 | OR6C8 |
| ENSBTAG00000054814 | 5 | 57687511 | 57687287 | 1 |  |
| ENSBTAG00000048372 | 5 | 57725043 | 57724117 | -1 | OR6C4 |
| ENSBTAG00000049847 | 5 | 57741833 | 57740889 | -1 | OR6C288 |
| ENSBTAG00000052662 | 5 | 57788656 | 57787718 | 1 | OR6C264 |
| ENSBTAG00000054300 | 5 | 57808585 | 57807632 | -1 | OR6C22 |
| ENSBTAG00000052908 | 5 | 57856406 | 57855480 | -1 | OR6C4C |
| ENSBTAG00000021122 | 5 | 57879057 | 57878122 | -1 | OR6C38 |
| ENSBTAG00000053821 | 5 | 57909721 | 57908786 | -1 | OR6C2F |
| ENSBTAG00000052453 | 5 | 57925916 | 57924954 | -1 | OR6C281 |
| ENSBTAG00000053506 | 5 | 58001322 | 58001219 | -1 | U6 |
| ENSBTAG00000047825 | 5 | 58068868 | 58067915 | -1 | OR6C202 |
| ENSBTAG00000052093 | 5 | 58100579 | 58099644 | -1 | OR6C207 |
| ENSBTAG00000049329 | 5 | 58113238 | 58112291 | -1 | OR6C278 |
| ENSBTAG00000051156 | 5 | 58127632 | 58126697 | -1 | OR6C17 |
| ENSBTAG00000046778 | 5 | 58180520 | 58179582 | -1 | OR6C35 |
| ENSBTAG00000048295 | 5 | 58208403 | 58207468 | -1 | OR6C68 |
| ENSBTAG00000054507 | 5 | 58255851 | 58254940 | 1 | OR6C276P |
| ENSBTAG00000050480 | 5 | 58272053 | 58271115 | -1 | OR6C269 |
| ENSBTAG00000051165 | 5 | 58286139 | 58285201 | -1 | OR6C2 |
| ENSBTAG00000051462 | 5 | 58372442 | 58371500 | -1 |  |
| ENSBTAG00000049219 | 5 | 58419158 | 58418220 | 1 | OR6C1R |
| ENSBTAG00000051274 | 5 | 58449501 | 58448566 | -1 | OR6C280 |
| ENSBTAG00000048779 | 5 | 58464561 | 58463626 | -1 | OR6C268 |
| ENSBTAG00000006313 | 5 | 58517061 | 58516084 | -1 | OR6C76 |
| ENSBTAG00000053837 | 5 | 58551477 | 58550539 | -1 | OR6C75 |
| ENSBTAG00000049581 | 5 | 58595757 | 58594819 | -1 | OR6C1Q |
| ENSBTAG00000049184 | 5 | 58619627 | 58618611 | 1 | OR6C7H |
| ENSBTAG00000048408 | 5 | 58695704 | 58694733 | 1 | OR6C7G |
| ENSBTAG00000024691 | 5 | 58758202 | 58757261 | -1 | OR6C1 |
| ENSBTAG00000051265 | 5 | 58766512 | 58765574 | -1 | OR6C5H |
| ENSBTAG00000042612 | 5 | 37587515 | 37587369 | 1 | SNORA62 |
| ENSBTAG00000008596 | 5 | 38171657 | 38058247 | 1 | PRICKLE1 |
| ENSBTAG00000043639 | 5 | 38088094 | 38087770 | 1 | 7SK |
| ENSBTAG00000049244 | 5 | 38144495 | 38144437 | -1 | bta-mir-12005-2 |
| ENSBTAG00000008595 | 5 | 38354903 | 38179856 | -1 | PPHLN1 |
| ENSBTAG00000019119 | 5 | 38372270 | 38354664 | 1 | ZCRB1 |
| ENSBTAG00000015025 | 5 | 38535924 | 38456114 | 1 | YAF2 |
| ENSBTAG00000015024 | 5 | 38591629 | 38545505 | 1 | GXYLT1 |
| ENSBTAG00000012624 | 5 | 39632049 | 39200973 | -1 | PDZRN4 |
| ENSBTAG00000001079 | 5 | 39421896 | 39416844 | -1 |  |
| ENSBTAG00000020679 | 5 | 40037754 | 39772742 | -1 | CNTN1 |
| ENSBTAG00000044848 | 5 | 39912185 | 39912077 | -1 |  |
| ENSBTAG00000016260 | 5 | 40666425 | 40461232 | -1 | LRRK2 |
| ENSBTAG00000043962 | 5 | 41331737 | 40812848 | 1 | SLC2A13 |
| ENSBTAG00000038043 | 5 | 41573833 | 41480497 | 1 | ABCD2 |
| ENSBTAG00000051251 | 5 | 41599892 | 41599737 | 1 |  |
| ENSBTAG00000004832 | 5 | 41918813 | 41732670 | 1 | KIF21A |
| ENSBTAG00000044680 | 5 | 41732915 | 41732851 | 1 | bta-mir-2428 |
| ENSBTAG00000049684 | 5 | 41770631 | 41770525 | 1 | U6 |
| ENSBTAG00000048924 | 5 | 41985115 | 41981007 | -1 |  |
| ENSBTAG00000020914 | 5 | 42472416 | 42193677 | 1 | CPNE8 |
| ENSBTAG00000015311 | 5 | 42884729 | 42611538 | 1 | PTPRR |
| ENSBTAG00000036950 | 5 | 42690587 | 42690468 | 1 | 5S_rRNA |
| ENSBTAG00000015296 | 5 | 43010556 | 42885405 | 1 | PTPRB |
| ENSBTAG00000052756 | 5 | 43005268 | 43005189 | 1 | bta-mir-2284z-5 |
| ENSBTAG00000003749 | 5 | 43120159 | 43038800 | -1 | KCNMB4 |
| ENSBTAG00000054094 | 5 | 43114855 | 43113516 | 1 |  |
| ENSBTAG00000003748 | 5 | 43266434 | 43134292 | -1 | CNOT2 |
| ENSBTAG00000044943 | 5 | 43201442 | 43201330 | 1 |  |
| ENSBTAG00000014829 | 5 | 43642194 | 43516703 | -1 | MYRFL |
| ENSBTAG00000050148 | 5 | 43651929 | 43637280 | 1 |  |
| ENSBTAG00000031950 | 5 | 43713702 | 43657005 | -1 | RAB3IP |
| ENSBTAG00000002931 | 5 | 43817060 | 43762572 | 1 | BEST3 |
| ENSBTAG00000019157 | 5 | 43866402 | 43863320 | 1 | LRRC10 |
| ENSBTAG00000019156 | 5 | 43884991 | 43869187 | -1 | CCT2 |
| ENSBTAG00000019155 | 5 | 43908506 | 43894715 | -1 | FRS2 |
| ENSBTAG00000048603 | 5 | 43901847 | 43901773 | 1 | bta-mir-2427 |
| ENSBTAG00000042237 | 5 | 43901910 | 43901783 | 1 |  |
| ENSBTAG00000045448 | 5 | 44023629 | 44023516 | 1 | 5S_rRNA |
| ENSBTAG00000002256 | 5 | 44110850 | 44086246 | -1 | YEATS4 |
| ENSBTAG00000011941 | 5 | 44125565 | 44119611 | -1 | LYZ1 |
| ENSBTAG00000044377 | 5 | 44134575 | 44134487 | 1 | U5 |
| ENSBTAG00000048739 | 5 | 44158611 | 44158508 | 1 | U6 |
| ENSBTAG00000022971 | 5 | 44194069 | 44189142 | 1 |  |
| ENSBTAG00000049929 | 5 | 44223299 | 44217916 | 1 |  |
| ENSBTAG00000020564 | 5 | 44252023 | 44246155 | 1 |  |
| ENSBTAG00000054492 | 5 | 44286850 | 44281434 | -1 |  |
| ENSBTAG00000000198 | 5 | 44302777 | 44297841 | 1 |  |
| ENSBTAG00000039170 | 5 | 44325369 | 44320272 | -1 |  |
| ENSBTAG00000026323 | 5 | 44352877 | 44347280 | 1 | LYSB |
| ENSBTAG00000026088 | 5 | 44371561 | 44365581 | -1 | LYZ2 |
| ENSBTAG00000046511 | 5 | 44400094 | 44392339 | -1 | LYZ1 |
| ENSBTAG00000046511 | 5 | 44400094 | 44392339 | -1 | LYZ1 |
| ENSBTAG00000046628 | 5 | 44430774 | 44423778 | -1 | LYZ3 |
| ENSBTAG00000026322 | 5 | 44449173 | 44445035 | -1 |  |
| ENSBTAG00000053948 | 5 | 44461016 | 44460958 | 1 | U6 |
| ENSBTAG00000043369 | 5 | 44505215 | 44505113 | 1 | U6 |
| ENSBTAG00000026779 | 5 | 44515796 | 44506989 | -1 | LYZ |
| ENSBTAG00000026779 | 5 | 44515796 | 44506989 | -1 | LYZ |
| ENSBTAG00000007323 | 5 | 44588478 | 44560547 | -1 | CPSF6 |
| ENSBTAG00000002741 | 5 | 44681707 | 44681351 | -1 |  |
| ENSBTAG00000044636 | 5 | 44708062 | 44707956 | 1 |  |
| ENSBTAG00000013496 | 5 | 44960458 | 44876588 | 1 | CPM |
| ENSBTAG00000010422 | 5 | 45005325 | 44979892 | -1 | MDM2 |
| ENSBTAG00000051975 | 5 | 45019012 | 44983558 | 1 |  |
| ENSBTAG00000031919 | 5 | 45065127 | 45047975 | -1 | SLC35E3 |
| ENSBTAG00000006911 | 5 | 45125506 | 45077381 | -1 | NUP107 |
| ENSBTAG00000042289 | 5 | 45089967 | 45089836 | -1 |  |
| ENSBTAG00000008967 | 5 | 45200873 | 45157005 | -1 | RAP1B |
| ENSBTAG00000019738 | 5 | 45475170 | 45446653 | 1 | MDM1 |
| ENSBTAG00000015407 | 5 | 45529263 | 45523409 | 1 | IL22 |
| ENSBTAG00000015395 | 5 | 45531263 | 45526671 | -1 |  |
| ENSBTAG00000052655 | 5 | 45562759 | 45548436 | 1 | IL26 |
| ENSBTAG00000012529 | 5 | 45629336 | 45624462 | 1 | IFNG |
| ENSBTAG00000033726 | 5 | 47474250 | 47140111 | 1 | GRIP1 |
| ENSBTAG00000028990 | 5 | 47374882 | 47374718 | 1 | U1 |
| ENSBTAG00000020806 | 5 | 47519482 | 47481804 | -1 | HELB |
| ENSBTAG00000053419 | 5 | 47505199 | 47504807 | 1 |  |
| ENSBTAG00000007636 | 5 | 47624668 | 47564309 | -1 | IRAK3 |
| ENSBTAG00000052954 | 5 | 47634494 | 47632527 | -1 |  |
| ENSBTAG00000026993 | 5 | 47661694 | 47645210 | 1 | TMBIM4 |
| ENSBTAG00000002004 | 5 | 47673186 | 47669190 | 1 | LLPH |
| ENSBTAG00000045454 | 5 | 47717752 | 47717635 | -1 | 5S_rRNA |
| ENSBTAG00000044118 | 5 | 47966760 | 47819505 | -1 | HMGA2 |
| ENSBTAG00000030111 | 5 | 47929899 | 47929780 | -1 | bta-mir-763 |
| ENSBTAG00000044017 | 5 | 48512740 | 48332243 | -1 | MSRB3 |
| ENSBTAG00000039435 | 5 | 48614389 | 48542860 | -1 | LEMD3 |
| ENSBTAG00000014758 | 5 | 48779884 | 48687578 | 1 | WIF1 |
| ENSBTAG00000042928 | 5 | 48767299 | 48767193 | 1 | U6 |
| ENSBTAG00000011352 | 5 | 49035846 | 48952181 | -1 | TBC1D30 |
| ENSBTAG00000000237 | 5 | 49018762 | 49017944 | 1 |  |
| ENSBTAG00000017465 | 5 | 49103597 | 49053812 | 1 | GNS |
| ENSBTAG00000051013 | 5 | 49190776 | 49114394 | -1 | RASSF3 |
| ENSBTAG00000047273 | 5 | 49220397 | 49220271 | -1 | 5S_rRNA |
| ENSBTAG00000017401 | 5 | 49315761 | 49272465 | -1 | TBK1 |
| ENSBTAG00000052994 | 5 | 49300857 | 49300788 | 1 | bta-mir-2285f-1 |
| ENSBTAG00000026962 | 5 | 49468018 | 49320765 | -1 | XPOT |
| ENSBTAG00000018861 | 5 | 49456410 | 49376642 | 1 | C5H12orf56 |
| ENSBTAG00000013486 | 5 | 49511314 | 49488842 | 1 | KICS2 |
| ENSBTAG00000011892 | 5 | 49890496 | 49582752 | -1 | SRGAP1 |
| ENSBTAG00000051362 | 5 | 49908149 | 49899198 | -1 |  |
| ENSBTAG00000004753 | 5 | 49945730 | 49919291 | -1 | RXYLT1 |
| ENSBTAG00000007175 | 5 | 50366965 | 50362882 | 1 | AVPR1A |
| ENSBTAG00000011857 | 5 | 50938091 | 50634073 | 1 | PPM1H |
| ENSBTAG00000029994 | 5 | 50983152 | 50983045 | -1 | MIRLET7I |
| ENSBTAG00000011068 | 5 | 51105717 | 50995056 | -1 | MON2 |
| ENSBTAG00000010428 | 5 | 51276079 | 51147112 | -1 | USP15 |
| ENSBTAG00000053892 | 5 | 51156649 | 51155558 | 1 |  |
| ENSBTAG00000047818 | 5 | 51924620 | 51752422 | 1 | TAFA2 |
| ENSBTAG00000040344 | 5 | 52576204 | 52575764 | -1 |  |
| ENSBTAG00000021287 | 5 | 53892992 | 53698084 | -1 | SLC16A7 |
| ENSBTAG00000055198 | 5 | 53786894 | 53785405 | -1 |  |
| ENSBTAG00000053531 | 5 | 53842000 | 53840974 | 1 |  |
| ENSBTAG00000055101 | 5 | 54534687 | 54534181 | -1 |  |
| ENSBTAG00000002227 | 5 | 54612806 | 54560835 | 1 | LRIG3 |
| ENSBTAG00000017543 | 5 | 55539039 | 55516050 | -1 | ATP23 |
| ENSBTAG00000016508 | 5 | 55656768 | 55634355 | 1 | CTDSP2 |
| ENSBTAG00000029869 | 5 | 55652156 | 55652073 | 1 | bta-mir-26a-2 |
| ENSBTAG00000016913 | 5 | 55676222 | 55657654 | 1 | AVIL |
| ENSBTAG00000016912 | 5 | 55685370 | 55677211 | -1 | TSFM |
| ENSBTAG00000016910 | 5 | 55699636 | 55685470 | -1 | EEF1AKMT3 |
| ENSBTAG00000016908 | 5 | 55697768 | 55693579 | 1 | METTL1 |
| ENSBTAG00000016906 | 5 | 55704141 | 55698030 | 1 | CYP27B1 |
| ENSBTAG00000016904 | 5 | 55710394 | 55705886 | -1 | MARCHF9 |
| ENSBTAG00000007160 | 5 | 55717031 | 55713433 | 1 | CDK4 |
| ENSBTAG00000007158 | 5 | 55719301 | 55716601 | -1 | TSPAN31 |
| ENSBTAG00000007156 | 5 | 55736914 | 55721786 | 1 | AGAP2 |
| ENSBTAG00000007152 | 5 | 55779980 | 55731741 | -1 | OS9 |
| ENSBTAG00000050652 | 5 | 55780606 | 55780373 | 1 |  |
| ENSBTAG00000049386 | 5 | 55853442 | 55853206 | -1 |  |
| ENSBTAG00000004494 | 5 | 55863814 | 55856907 | 1 | B4GALNT1 |
| ENSBTAG00000023289 | 5 | 55870734 | 55863909 | -1 | SLC26A10 |
| ENSBTAG00000018003 | 5 | 55879740 | 55872512 | -1 | ARHGEF25 |
| ENSBTAG00000018002 | 5 | 55885193 | 55880132 | -1 | DTX3 |
| ENSBTAG00000003942 | 5 | 55898381 | 55886481 | -1 | PIP4K2C |
| ENSBTAG00000051574 | 5 | 55902019 | 55900690 | -1 |  |
| ENSBTAG00000021336 | 5 | 55931562 | 55902461 | -1 | KIF5A |
| ENSBTAG00000051593 | 5 | 55915193 | 55905833 | 1 |  |
| ENSBTAG00000010624 | 5 | 55946889 | 55932944 | 1 | DCTN2 |
| ENSBTAG00000010616 | 5 | 55953054 | 55947935 | -1 | MBD6 |
| ENSBTAG00000031544 | 5 | 55960509 | 55955676 | 1 | DDIT3 |
| ENSBTAG00000018405 | 5 | 55988093 | 55960451 | -1 | MARS1 |
| ENSBTAG00000044363 | 5 | 55964538 | 55964466 | -1 | bta-mir-2430 |
| ENSBTAG00000018403 | 5 | 55992412 | 55984863 | 1 | ARHGAP9 |
| ENSBTAG00000006631 | 5 | 56003064 | 55992686 | -1 | GLI1 |
| ENSBTAG00000006599 | 5 | 56006928 | 56005014 | -1 | INHBE |
| ENSBTAG00000019084 | 5 | 56022178 | 56011447 | -1 | INHBC |
| ENSBTAG00000018361 | 5 | 56191524 | 56081334 | 1 | R3HDM2 |
| ENSBTAG00000018358 | 5 | 56200300 | 56193684 | 1 | STAC3 |
| ENSBTAG00000031503 | 5 | 56208161 | 56205859 | 1 | NDUFA4L2 |
| ENSBTAG00000031500 | 5 | 56213412 | 56208141 | -1 | SHMT2 |
| ENSBTAG00000047650 | 5 | 56225814 | 56216259 | -1 | NXPH4 |
| ENSBTAG00000010830 | 5 | 56310118 | 56230519 | -1 | LRP1 |
| ENSBTAG00000043214 | 5 | 56321174 | 56321025 | 1 | SNORA62 |
| ENSBTAG00000045218 | 5 | 56324140 | 56324073 | -1 | bta-mir-2431 |
| ENSBTAG00000006335 | 5 | 56339539 | 56325609 | 1 | STAT6 |
| ENSBTAG00000006324 | 5 | 56346527 | 56340377 | -1 | NAB2 |
| ENSBTAG00000031076 | 5 | 56355368 | 56354793 | -1 |  |
| ENSBTAG00000014659 | 5 | 56375772 | 56356649 | 1 | NEMP1 |
| ENSBTAG00000014655 | 5 | 56406111 | 56378741 | 1 | MYO1A |
| ENSBTAG00000021807 | 5 | 56418016 | 56410874 | 1 | TAC3 |
| ENSBTAG00000038498 | 5 | 56434255 | 56432132 | 1 | ZBTB39 |
| ENSBTAG00000039157 | 5 | 56443098 | 56441009 | -1 | GPR182 |
| ENSBTAG00000049045 | 5 | 56450052 | 56449519 | -1 |  |
| ENSBTAG00000049081 | 5 | 56468834 | 56463654 | 1 |  |
| ENSBTAG00000050051 | 5 | 56495223 | 56490433 | 1 |  |
| ENSBTAG00000001392 | 5 | 56537384 | 56531475 | 1 | RDH16 |
| ENSBTAG00000013443 | 5 | 56554855 | 56545369 | 1 | SDR9C7 |
| ENSBTAG00000055155 | 5 | 56602525 | 56599458 | 1 |  |
| ENSBTAG00000051761 | 5 | 56614396 | 56601985 | -1 |  |
| ENSBTAG00000042169 | 5 | 56645657 | 56645578 | 1 |  |
| ENSBTAG00000043104 | 5 | 56645893 | 56645822 | 1 |  |
| ENSBTAG00000011720 | 5 | 56696104 | 56674172 | -1 | HSD17B6 |
| ENSBTAG00000044000 | 5 | 56729540 | 56711750 | 1 | PRIM1 |
| ENSBTAG00000010701 | 5 | 56743636 | 56711826 | 1 | NACA |
| ENSBTAG00000055262 | 5 | 56759353 | 56758238 | 1 |  |
| ENSBTAG00000017967 | 5 | 56782192 | 56760933 | 1 | PTGES3 |
| ENSBTAG00000048365 | 5 | 1.16E+08 | 1.16E+08 | -1 | WNT7B |
| ENSBTAG00000050704 | 5 | 1.16E+08 | 1.16E+08 | 1 |  |
| ENSBTAG00000029772 | 5 | 1.16E+08 | 1.16E+08 | 1 | bta-let-7a-3 |
| ENSBTAG00000045309 | 5 | 1.16E+08 | 1.16E+08 | 1 | bta-mir-2443 |
| ENSBTAG00000036417 | 5 | 1.16E+08 | 1.16E+08 | -1 | bta-mir-3596 |
| ENSBTAG00000048921 | 5 | 1.16E+08 | 1.16E+08 | -1 |  |
| ENSBTAG00000008063 | 5 | 1.17E+08 | 1.16E+08 | 1 | PPARA |
| ENSBTAG00000008065 | 5 | 1.17E+08 | 1.17E+08 | -1 | CDPF1 |
| ENSBTAG00000050005 | 5 | 1.17E+08 | 1.17E+08 | -1 |  |
| ENSBTAG00000054859 | 5 | 1.17E+08 | 1.17E+08 | -1 |  |
| ENSBTAG00000008066 | 5 | 1.17E+08 | 1.17E+08 | -1 |  |
| ENSBTAG00000003017 | 5 | 1.17E+08 | 1.17E+08 | 1 | TTC38 |
| ENSBTAG00000007102 | 5 | 1.17E+08 | 1.17E+08 | 1 | GTSE1 |
| ENSBTAG00000005595 | 5 | 1.17E+08 | 1.17E+08 | 1 | TRMU |
| ENSBTAG00000008036 | 5 | 1.17E+08 | 1.17E+08 | -1 | CELSR1 |
| ENSBTAG00000021803 | 5 | 1.17E+08 | 1.17E+08 | 1 | GRAMD4 |
| ENSBTAG00000046654 | 5 | 1.17E+08 | 1.17E+08 | -1 | CERK |
| ENSBTAG00000054562 | 5 | 1.17E+08 | 1.17E+08 | 1 |  |
| ENSBTAG00000012291 | 5 | 1.17E+08 | 1.17E+08 | 1 | TBC1D22A |
| ENSBTAG00000044449 | 5 | 1.17E+08 | 1.17E+08 | -1 | bta-mir-2285o-5 |
| ENSBTAG00000051431 | 5 | 1.18E+08 | 1.18E+08 | -1 |  |
| ENSBTAG00000022986 | 5 | 1.18E+08 | 1.18E+08 | 1 | TAFA5 |
| ENSBTAG00000030185 | 5 | 1.19E+08 | 1.19E+08 | -1 |  |
| ENSBTAG00000014987 | 5 | 1.19E+08 | 1.19E+08 | 1 |  |
| ENSBTAG00000046760 | 5 | 1.19E+08 | 1.19E+08 | -1 | BRD1 |
| ENSBTAG00000048741 | 5 | 1.19E+08 | 1.19E+08 | 1 |  |
| ENSBTAG00000049889 | 5 | 1.19E+08 | 1.19E+08 | 1 | ZBED4 |
| ENSBTAG00000046173 | 5 | 1.19E+08 | 1.19E+08 | -1 | ALG12 |
| ENSBTAG00000047801 | 5 | 1.19E+08 | 1.19E+08 | 1 | CRELD2 |
| ENSBTAG00000049825 | 5 | 1.19E+08 | 1.19E+08 | -1 |  |
| ENSBTAG00000045904 | 5 | 1.19E+08 | 1.19E+08 | 1 | PIM3 |
| ENSBTAG00000045808 | 5 | 1.19E+08 | 1.19E+08 | -1 | IL17REL |
| ENSBTAG00000011098 | 5 | 1.19E+08 | 1.19E+08 | -1 | TTLL8 |
| ENSBTAG00000021626 | 5 | 1.19E+08 | 1.19E+08 | -1 | MLC1 |
| ENSBTAG00000020341 | 5 | 1.2E+08 | 1.19E+08 | 1 | MOV10L1 |
| ENSBTAG00000046844 | 5 | 1.2E+08 | 1.2E+08 | -1 |  |
| ENSBTAG00000047080 | 5 | 1.2E+08 | 1.2E+08 | 1 |  |
| ENSBTAG00000048285 | 5 | 1.2E+08 | 1.2E+08 | -1 | bta-mir-2894 |
| ENSBTAG00000009297 | 5 | 1.2E+08 | 1.2E+08 | 1 | PANX2 |
| ENSBTAG00000001931 | 5 | 1.2E+08 | 1.2E+08 | 1 | TRABD |
| ENSBTAG00000000647 | 5 | 1.2E+08 | 1.2E+08 | 1 | SELENOO |
| ENSBTAG00000000650 | 5 | 1.2E+08 | 1.2E+08 | -1 | TUBGCP6 |
| ENSBTAG00000011000 | 5 | 1.2E+08 | 1.2E+08 | -1 | HDAC10 |
| ENSBTAG00000019574 | 5 | 1.2E+08 | 1.2E+08 | -1 | MAPK12 |
| ENSBTAG00000030182 | 5 | 1.2E+08 | 1.2E+08 | -1 | MAPK11 |
| ENSBTAG00000014966 | 5 | 1.2E+08 | 1.2E+08 | -1 | PLXNB2 |
| ENSBTAG00000024756 | 5 | 1.2E+08 | 1.2E+08 | -1 | DENND6B |
| ENSBTAG00000018660 | 5 | 1.2E+08 | 1.2E+08 | 1 | PPP6R2 |
| ENSBTAG00000021337 | 5 | 1.2E+08 | 1.2E+08 | -1 | SBF1 |
| ENSBTAG00000051406 | 5 | 1.2E+08 | 1.2E+08 | 1 |  |
| ENSBTAG00000054072 | 5 | 1.2E+08 | 1.2E+08 | 1 | ADM2 |
| ENSBTAG00000002559 | 5 | 1.2E+08 | 1.2E+08 | 1 | MIOX |
| ENSBTAG00000017925 | 5 | 1.2E+08 | 1.2E+08 | -1 | LMF2 |
| ENSBTAG00000012607 | 5 | 1.2E+08 | 1.2E+08 | 1 | NCAPH2 |
| ENSBTAG00000012609 | 5 | 1.2E+08 | 1.2E+08 | -1 | SCO2 |
| ENSBTAG00000026467 | 5 | 1.2E+08 | 1.2E+08 | -1 | ODF3B |
| ENSBTAG00000046800 | 5 | 1.2E+08 | 1.2E+08 | 1 | KLHDC7B |
| ENSBTAG00000046298 | 5 | 1.2E+08 | 1.2E+08 | -1 | SYCE3 |
| ENSBTAG00000016048 | 5 | 1.2E+08 | 1.2E+08 | -1 | CPT1B |
| ENSBTAG00000016050 | 5 | 1.2E+08 | 1.2E+08 | -1 | CHKB |
| ENSBTAG00000030179 | 5 | 1.2E+08 | 1.2E+08 | 1 | MAPK8IP2 |
| ENSBTAG00000016053 | 5 | 1.2E+08 | 1.2E+08 | -1 | ARSA |
| ENSBTAG00000049874 | 5 | 1.2E+08 | 1.2E+08 | -1 |  |
| ENSBTAG00000030180 | 5 | 1.2E+08 | 1.2E+08 | 1 | SHANK3 |
| ENSBTAG00000010182 | 5 | 1.2E+08 | 1.2E+08 | 1 | ACR |
| ENSBTAG00000053921 | 5 | 1.2E+08 | 1.2E+08 | -1 |  |
| ENSBTAG00000004876 | 5 | 1.2E+08 | 1.2E+08 | 1 | RABL2B |
| ENSBTAG00000049261 | 6 | 97116657 | 97093002 | 1 |  |
| ENSBTAG00000013952 | 6 | 97160733 | 97143335 | -1 | HNRNPD |
| ENSBTAG00000014804 | 6 | 97216516 | 97209613 | -1 | HNRNPDL |
| ENSBTAG00000014805 | 6 | 97255729 | 97217195 | 1 | ENOPH1 |
| ENSBTAG00000032588 | 6 | 97388558 | 97276663 | -1 | TMEM150C |
| ENSBTAG00000042503 | 6 | 97300654 | 97300515 | -1 | U4 |
| ENSBTAG00000022449 | 6 | 97637722 | 97461721 | -1 | SCD5 |
| ENSBTAG00000020525 | 6 | 97713798 | 97653816 | -1 | SEC31A |
| ENSBTAG00000022450 | 6 | 97745383 | 97724566 | 1 | THAP9 |
| ENSBTAG00000010546 | 6 | 97815165 | 97753005 | -1 | LIN54 |
| ENSBTAG00000045290 | 6 | 97814674 | 97814598 | -1 | bta-mir-2447 |
| ENSBTAG00000006950 | 6 | 97875459 | 97836116 | 1 | COPS4 |
| ENSBTAG00000049607 | 6 | 97859143 | 97859009 | 1 | SNORA70 |
| ENSBTAG00000031750 | 6 | 97916809 | 97881687 | -1 | PLAC8B |
| ENSBTAG00000009849 | 6 | 97980661 | 97946767 | -1 | PLAC8A |
| ENSBTAG00000005744 | 6 | 98065079 | 98037930 | -1 | COQ2 |
| ENSBTAG00000046700 | 6 | 98061068 | 98059818 | -1 |  |
| ENSBTAG00000005745 | 6 | 98118656 | 98072170 | -1 | HPSE |
| ENSBTAG00000018151 | 6 | 98225864 | 98186192 | -1 | HELQ |
| ENSBTAG00000045132 | 6 | 98196451 | 98196381 | -1 | bta-mir-2446 |
| ENSBTAG00000018155 | 6 | 98231259 | 98226288 | 1 | MRPS18C |
| ENSBTAG00000014076 | 6 | 98250555 | 98233356 | -1 | ABRAXAS1 |
| ENSBTAG00000051440 | 6 | 98289596 | 98288577 | -1 |  |
| ENSBTAG00000017592 | 6 | 98360326 | 98289617 | 1 | GPAT3 |
| ENSBTAG00000043607 | 6 | 98666843 | 98666737 | 1 | U6 |
| ENSBTAG00000002518 | 6 | 99312032 | 99306821 | -1 | NKX6-1 |
| ENSBTAG00000045787 | 6 | 99488979 | 99411738 | 1 | CDS1 |
| ENSBTAG00000013495 | 6 | 99723220 | 99508632 | -1 | WDFY3 |
| ENSBTAG00000026936 | 6 | 13044915 | 12992269 | 1 | ZGRF1 |
| ENSBTAG00000015259 | 6 | 13068319 | 13066827 | 1 | NEUROG2 |
| ENSBTAG00000034875 | 6 | 13220016 | 13098687 | -1 | ALPK1 |
| ENSBTAG00000049422 | 6 | 13237458 | 13228624 | 1 | TIFA |
| ENSBTAG00000003941 | 6 | 13272540 | 13243676 | -1 | AP1AR |
| ENSBTAG00000017375 | 6 | 13375661 | 13332381 | -1 | FAM241A |
| ENSBTAG00000044440 | 6 | 13639576 | 13639445 | 1 |  |
| ENSBTAG00000009059 | 6 | 14851254 | 14831302 | 1 | PITX2 |
| ENSBTAG00000051492 | 6 | 14857020 | 14853110 | 1 |  |
| ENSBTAG00000008332 | 6 | 14992464 | 14912679 | -1 | ENPEP |
| ENSBTAG00000054468 | 6 | 14967667 | 14967428 | 1 |  |
| ENSBTAG00000051202 | 6 | 15003729 | 15002105 | 1 |  |
| ENSBTAG00000044354 | 6 | 15046413 | 15046306 | -1 | U6 |
| ENSBTAG00000049611 | 6 | 15159369 | 15159235 | 1 | SNORA70 |
| ENSBTAG00000052157 | 6 | 99784966 | 99766484 | -1 |  |
| ENSBTAG00000010564 | 6 | 15360477 | 15199666 | 1 | ELOVL6 |
| ENSBTAG00000038368 | 6 | 15219795 | 15219565 | -1 |  |
| ENSBTAG00000051135 | 6 | 15442006 | 15433158 | -1 |  |
| ENSBTAG00000048812 | 6 | 15464657 | 15445061 | -1 | EGF |
| ENSBTAG00000003959 | 6 | 1.01E+08 | 1.01E+08 | 1 | ARHGAP24 |
| ENSBTAG00000046949 | 6 | 15526312 | 15525968 | 1 |  |
| ENSBTAG00000007019 | 6 | 15569552 | 15551780 | -1 | LRIT3 |
| ENSBTAG00000053284 | 6 | 15607997 | 15567535 | 1 |  |
| ENSBTAG00000000751 | 6 | 15590078 | 15577365 | -1 | RRH |
| ENSBTAG00000000748 | 6 | 15604174 | 15597411 | -1 | GAR1 |
| ENSBTAG00000034501 | 6 | 15664735 | 15613508 | 1 | CFI |
| ENSBTAG00000020048 | 6 | 1.01E+08 | 1.01E+08 | -1 | MAPK10 |
| ENSBTAG00000015230 | 6 | 15690031 | 15675549 | 1 | PLA2G12A |
| ENSBTAG00000012537 | 6 | 15715905 | 15694143 | 1 | CASP6 |
| ENSBTAG00000012995 | 6 | 15813728 | 15717981 | -1 | MCUB |
| ENSBTAG00000010945 | 6 | 15906195 | 15825962 | -1 | SEC24B |
| ENSBTAG00000049691 | 6 | 15915585 | 15911457 | 1 |  |
| ENSBTAG00000042213 | 6 | 16526323 | 16526189 | 1 | SNORA70 |
| ENSBTAG00000042137 | 6 | 16544172 | 16544067 | 1 | U6 |
| ENSBTAG00000010284 | 6 | 16573066 | 16554037 | 1 | ETNPPL |
| ENSBTAG00000011611 | 6 | 16644454 | 16632776 | -1 | OSTC |
| ENSBTAG00000014226 | 6 | 16675901 | 16671257 | -1 | RPL34 |
| ENSBTAG00000006844 | 6 | 17200183 | 17083864 | 1 | LEF1 |
| ENSBTAG00000002049 | 6 | 17252208 | 17208776 | -1 | HADH |
| ENSBTAG00000012972 | 6 | 17282966 | 17261763 | -1 | CYP2U1 |
| ENSBTAG00000016805 | 6 | 17391885 | 17297001 | -1 | SGMS2 |
| ENSBTAG00000012961 | 6 | 17601500 | 17489062 | 1 | PAPSS1 |
| ENSBTAG00000012969 | 6 | 18254810 | 18125623 | 1 | DKK2 |
| ENSBTAG00000054885 | 6 | 18159590 | 18158398 | -1 |  |
| ENSBTAG00000050450 | 8 | 30979829 | 30975161 | 1 |  |
| ENSBTAG00000043961 | 8 | 31211135 | 31058380 | 1 | MPDZ |
| ENSBTAG00000010431 | 8 | 31557952 | 31503232 | -1 | LURAP1L |
| ENSBTAG00000020985 | 8 | 31648326 | 31632080 | -1 | TYRP1 |
| ENSBTAG00000053781 | 8 | 32085560 | 32085084 | -1 |  |
| ENSBTAG00000051289 | 8 | 32102912 | 32102806 | 1 | U6 |
| ENSBTAG00000043919 | 8 | 33092466 | 33092369 | 1 | U6 |
| ENSBTAG00000028923 | 8 | 33232513 | 33232371 | -1 | U1 |
| ENSBTAG00000047184 | 8 | 36026060 | 36026002 | 1 | bta-mir-2285bg |
| ENSBTAG00000010178 | 8 | 36600302 | 36145265 | 1 | PTPRD |
| ENSBTAG00000043680 | 8 | 36893702 | 36893387 | 1 | 7SK |
| ENSBTAG00000015963 | 8 | 37096906 | 37095276 | 1 | DMAC1 |
| ENSBTAG00000001926 | 8 | 97057375 | 97012935 | 1 | RAD23B |
| ENSBTAG00000054995 | 8 | 97166056 | 97162258 | -1 |  |
| ENSBTAG00000020355 | 8 | 97179335 | 97174615 | -1 | KLF4 |
| ENSBTAG00000049647 | 8 | 97295405 | 97293782 | 1 |  |
| ENSBTAG00000053678 | 8 | 97485478 | 97479095 | 1 |  |
| ENSBTAG00000043059 | 8 | 97679244 | 97679144 | -1 | U6 |
| ENSBTAG00000048201 | 8 | 98008315 | 98008208 | 1 | U6 |
| ENSBTAG00000000885 | 8 | 98176461 | 98172180 | -1 |  |
| ENSBTAG00000019467 | 8 | 98464130 | 98462724 | -1 | ACTL7B |
| ENSBTAG00000019468 | 8 | 98467807 | 98466288 | 1 | ACTL7A |
| ENSBTAG00000004991 | 8 | 98536474 | 98474958 | -1 | ELP1 |
| ENSBTAG00000004996 | 8 | 98543210 | 98536710 | 1 | ABITRAM |
| ENSBTAG00000004997 | 8 | 98601628 | 98544960 | -1 | CTNNAL1 |
| ENSBTAG00000051855 | 8 | 98607193 | 98606975 | -1 |  |
| ENSBTAG00000038794 | 8 | 98686264 | 98619608 | -1 | TMEM245 |
| ENSBTAG00000029778 | 8 | 98629092 | 98629023 | -1 | bta-mir-32 |
| ENSBTAG00000047947 | 8 | 98676495 | 98676196 | -1 | Metazoa_SRP |
| ENSBTAG00000050794 | 8 | 98676272 | 98676196 | -1 |  |
| ENSBTAG00000017936 | 8 | 98726191 | 98701322 | -1 | FRRS1L |
| ENSBTAG00000017063 | 8 | 98868703 | 98732990 | -1 | EPB41L4B |
| ENSBTAG00000048854 | 8 | 98748450 | 98747570 | 1 |  |
| ENSBTAG00000018841 | 8 | 99092707 | 98927206 | -1 | PTPN3 |
| ENSBTAG00000045486 | 8 | 98991381 | 98991264 | -1 | U2 |
| ENSBTAG00000014471 | 8 | 99721951 | 99312764 | 1 | PALM2 |
| ENSBTAG00000043348 | 8 | 99627699 | 99627597 | -1 | U6 |
| ENSBTAG00000003240 | 8 | 99752613 | 99746343 | -1 | C8H9orf152 |
| ENSBTAG00000002953 | 8 | 99811602 | 99799187 | -1 | TXN |
| ENSBTAG00000049270 | 8 | 99819193 | 99815534 | -1 |  |
| ENSBTAG00000010535 | 8 | 99867723 | 99835331 | -1 | TXNDC8 |
| ENSBTAG00000020243 | 8 | 1E+08 | 99929908 | -1 | SVEP1 |
| ENSBTAG00000043730 | 8 | 99958594 | 99958471 | 1 | 5S_rRNA |
| ENSBTAG00000002744 | 8 | 1E+08 | 1E+08 | 1 | MUSK |
| ENSBTAG00000009654 | 8 | 1.01E+08 | 1E+08 | -1 | LPAR1 |
| ENSBTAG00000043478 | 8 | 1E+08 | 1E+08 | -1 | SNORA71 |
| ENSBTAG00000044489 | 8 | 1.01E+08 | 1.01E+08 | -1 | bta-mir-2285n-5 |
| ENSBTAG00000050421 | 8 | 1.01E+08 | 1.01E+08 | -1 |  |
| ENSBTAG00000010686 | 8 | 1.01E+08 | 1.01E+08 | -1 | OR2K2 |
| ENSBTAG00000006712 | 8 | 1.01E+08 | 1.01E+08 | -1 | ECPAS |
| ENSBTAG00000008101 | 8 | 52419336 | 51911490 | 1 | PCSK5 |
| ENSBTAG00000019345 | 8 | 52459253 | 52453482 | -1 | RFK |
| ENSBTAG00000012757 | 8 | 52568603 | 52522641 | 1 | GCNT1 |
| ENSBTAG00000053347 | 8 | 52718485 | 52677323 | -1 |  |
| ENSBTAG00000012991 | 8 | 52887222 | 52760745 | -1 | PRUNE2 |
| ENSBTAG00000048354 | 8 | 52964761 | 52772680 | -1 |  |
| ENSBTAG00000048391 | 8 | 52837074 | 52836815 | 1 |  |
| ENSBTAG00000053573 | 8 | 52842902 | 52842493 | 1 |  |
| ENSBTAG00000032983 | 8 | 53065420 | 53064146 | 1 | FOXB2 |
| ENSBTAG00000051821 | 8 | 53213188 | 53179167 | 1 |  |
| ENSBTAG00000017734 | 8 | 53450193 | 53287860 | 1 |  |
| ENSBTAG00000021127 | 8 | 53665709 | 53465620 | -1 | GNA14 |
| ENSBTAG00000009789 | 8 | 53996400 | 53685832 | -1 | GNAQ |
| ENSBTAG00000020752 | 8 | 54183987 | 54148387 | 1 | CEP78 |
| ENSBTAG00000013960 | 8 | 54245158 | 54216465 | 1 | PSAT1 |
| ENSBTAG00000024233 | 8 | 54665074 | 54664424 | 1 |  |
| ENSBTAG00000043622 | 8 | 54983975 | 54983872 | -1 | U6 |
| ENSBTAG00000003532 | 8 | 55729912 | 55576283 | 1 | TLE4 |
| ENSBTAG00000053348 | 8 | 56752204 | 56716918 | -1 |  |
| ENSBTAG00000006294 | 8 | 57091938 | 57091711 | -1 |  |
| ENSBTAG00000000256 | 8 | 57625114 | 57533899 | -1 | TLE1 |
| ENSBTAG00000052126 | 8 | 57678978 | 57626717 | 1 |  |
| ENSBTAG00000005667 | 8 | 57838652 | 57835669 | 1 |  |
| ENSBTAG00000050015 | 8 | 58190416 | 58188709 | 1 |  |
| ENSBTAG00000042498 | 8 | 59018095 | 59017960 | -1 |  |
| ENSBTAG00000049991 | 8 | 59139594 | 59131211 | -1 |  |
| ENSBTAG00000017338 | 8 | 59182194 | 59176689 | -1 | FAM205C |
| ENSBTAG00000012809 | 8 | 59267220 | 59245071 | 1 | PHF24 |
| ENSBTAG00000054568 | 8 | 59272352 | 59272246 | -1 | U6 |
| ENSBTAG00000018318 | 8 | 59286526 | 59277670 | 1 | DNAJB5 |
| ENSBTAG00000051343 | 8 | 59318133 | 59314013 | 1 | C8H9orf131 |
| ENSBTAG00000015000 | 8 | 59336523 | 59321791 | -1 | VCP |
| ENSBTAG00000015005 | 8 | 59343676 | 59337760 | -1 | FANCG |
| ENSBTAG00000044135 | 8 | 59351847 | 59345169 | -1 | PIGO |
| ENSBTAG00000011388 | 8 | 59361681 | 59358080 | -1 | STOML2 |
| ENSBTAG00000011394 | 8 | 59373813 | 59362241 | -1 | FAM214B |
| ENSBTAG00000011397 | 8 | 59599764 | 59373966 | 1 | UNC13B |
| ENSBTAG00000011402 | 8 | 59710590 | 59637289 | 1 |  |
| ENSBTAG00000011403 | 8 | 59785504 | 59723669 | 1 | RUSC2 |
| ENSBTAG00000049403 | 8 | 59769489 | 59769431 | -1 | bta-mir-2285da |
| ENSBTAG00000032509 | 8 | 59787520 | 59785446 | -1 | FAM166B |
| ENSBTAG00000011406 | 8 | 59801419 | 59795969 | 1 | TESK1 |
| ENSBTAG00000011409 | 8 | 59808752 | 59799960 | -1 | CD72 |
| ENSBTAG00000011411 | 8 | 59829096 | 59827546 | -1 | SIT1 |
| ENSBTAG00000042458 | 8 | 59833395 | 59833118 | -1 | RNase_MRP |
| ENSBTAG00000011413 | 8 | 59836253 | 59833726 | 1 | CCDC107 |
| ENSBTAG00000011416 | 8 | 59840144 | 59836548 | -1 | ARHGEF39 |
| ENSBTAG00000011420 | 8 | 59855369 | 59849175 | 1 | CA9 |
| ENSBTAG00000011424 | 8 | 59864645 | 59856503 | -1 | TPM2 |
| ENSBTAG00000050230 | 8 | 59867679 | 59867623 | 1 | bta-mir-12021 |
| ENSBTAG00000025868 | 8 | 59901292 | 59869867 | -1 | TLN1 |
| ENSBTAG00000012390 | 8 | 1.01E+08 | 1.01E+08 | 1 | ZNF483 |
| ENSBTAG00000011027 | 8 | 1.01E+08 | 1.01E+08 | -1 | PTGR1 |
| ENSBTAG00000030960 | 8 | 1.01E+08 | 1.01E+08 | 1 | DNAJC25 |
| ENSBTAG00000009087 | 8 | 1.01E+08 | 1.01E+08 | 1 | GNG10 |
| ENSBTAG00000044004 | 8 | 1.01E+08 | 1.01E+08 | -1 | SHOC1 |
| ENSBTAG00000011429 | 8 | 59905938 | 59901572 | 1 | CREB3 |
| ENSBTAG00000053723 | 8 | 1.01E+08 | 1.01E+08 | 1 | bta-mir-7863 |
| ENSBTAG00000011431 | 8 | 59921540 | 59901585 | -1 | GBA2 |
| ENSBTAG00000016073 | 8 | 1.01E+08 | 1.01E+08 | 1 | UGCG |
| ENSBTAG00000036542 | 8 | 1.01E+08 | 1.01E+08 | 1 | 5S_rRNA |
| ENSBTAG00000004813 | 8 | 1.02E+08 | 1.01E+08 | -1 | SUSD1 |
| ENSBTAG00000052536 | 8 | 1.02E+08 | 1.02E+08 | 1 | U6 |
| ENSBTAG00000016750 | 8 | 1.02E+08 | 1.02E+08 | -1 | PTBP3 |
| ENSBTAG00000038335 | 8 | 1.02E+08 | 1.02E+08 | -1 |  |
| ENSBTAG00000031295 | 8 | 1.02E+08 | 1.02E+08 | 1 | HSDL2 |
| ENSBTAG00000016259 | 8 | 1.02E+08 | 1.02E+08 | 1 | KIAA1958 |
| ENSBTAG00000019275 | 8 | 1.02E+08 | 1.02E+08 | -1 | INIP |
| ENSBTAG00000011433 | 8 | 59922869 | 59917058 | 1 | RGP1 |
| ENSBTAG00000044070 | 8 | 1.02E+08 | 1.02E+08 | 1 | SNX30 |
| ENSBTAG00000013670 | 8 | 1.02E+08 | 1.02E+08 | -1 | SLC46A2 |
| ENSBTAG00000051753 | 8 | 1.02E+08 | 1.02E+08 | -1 |  |
| ENSBTAG00000018481 | 8 | 1.02E+08 | 1.02E+08 | -1 |  |
| ENSBTAG00000038777 | 8 | 59922048 | 59921093 | -1 | MSMP |
| ENSBTAG00000049289 | 8 | 59954953 | 59950294 | 1 |  |
| ENSBTAG00000011434 | 8 | 59974722 | 59956219 | 1 | NPR2 |
| ENSBTAG00000047990 | 8 | 1.02E+08 | 1.02E+08 | 1 |  |
| ENSBTAG00000045868 | 8 | 1.02E+08 | 1.02E+08 | -1 | ZFP37 |
| ENSBTAG00000055266 | 8 | 1.02E+08 | 1.02E+08 | -1 |  |
| ENSBTAG00000011442 | 8 | 59977270 | 59974305 | -1 | SPAG8 |
| ENSBTAG00000005115 | 8 | 1.02E+08 | 1.02E+08 | 1 | SLC31A2 |
| ENSBTAG00000011444 | 8 | 59981974 | 59979689 | -1 | HINT2 |
| ENSBTAG00000005116 | 8 | 1.02E+08 | 1.02E+08 | -1 | FKBP15 |
| ENSBTAG00000005222 | 8 | 59998097 | 59988842 | -1 | FAM221B |
| ENSBTAG00000011445 | 8 | 60027284 | 60003644 | 1 | TMEM8B |
| ENSBTAG00000021678 | 8 | 1.03E+08 | 1.02E+08 | 1 | SLC31A1 |
| ENSBTAG00000014430 | 8 | 1.03E+08 | 1.03E+08 | -1 | CDC26 |
| ENSBTAG00000004571 | 8 | 1.03E+08 | 1.03E+08 | 1 | PRPF4 |
| ENSBTAG00000053732 | 8 | 60031708 | 60030737 | -1 | OR13E1 |
| ENSBTAG00000052564 | 8 | 60072943 | 60071969 | -1 | OR13E10 |
| ENSBTAG00000052220 | 8 | 60082226 | 60081288 | -1 | OR13J1C |
| ENSBTAG00000048744 | 8 | 60099946 | 60099008 | -1 | OR13J1F |
| ENSBTAG00000053868 | 8 | 60119603 | 60118665 | -1 | OR13J1 |
| ENSBTAG00000052334 | 8 | 60127138 | 60126153 | -1 |  |
| ENSBTAG00000049107 | 8 | 60135482 | 60134546 | -1 |  |
| ENSBTAG00000052234 | 8 | 60143900 | 60142915 | -1 |  |
| ENSBTAG00000054526 | 8 | 60152260 | 60151322 | -1 | OR13J1D |
| ENSBTAG00000049192 | 8 | 60169861 | 60168923 | -1 | OR13J1G |
| ENSBTAG00000004572 | 8 | 1.03E+08 | 1.03E+08 | -1 | RNF183 |
| ENSBTAG00000052875 | 8 | 60181902 | 60180940 | -1 | OR13E12 |
| ENSBTAG00000005884 | 8 | 1.03E+08 | 1.03E+08 | -1 | WDR31 |
| ENSBTAG00000054837 | 8 | 60188647 | 60187709 | -1 | OR13J1B |
| ENSBTAG00000055315 | 8 | 1.03E+08 | 1.03E+08 | 1 |  |
| ENSBTAG00000055183 | 8 | 60215554 | 60215210 | 1 | HRCT1 |
| ENSBTAG00000044155 | 8 | 1.03E+08 | 1.03E+08 | 1 | BSPRY |
| ENSBTAG00000050436 | 8 | 60220317 | 60220090 | 1 | SPAAR |
| ENSBTAG00000049084 | 8 | 60259952 | 60258993 | -1 | OR13C11 |
| ENSBTAG00000000250 | 8 | 1.03E+08 | 1.03E+08 | -1 | HDHD3 |
| ENSBTAG00000036921 | 8 | 60264010 | 60263895 | -1 | 5S_rRNA |
| ENSBTAG00000052921 | 8 | 60270231 | 60269272 | -1 | OR13C7 |
| ENSBTAG00000000251 | 8 | 1.03E+08 | 1.03E+08 | -1 | ALAD |
| ENSBTAG00000054503 | 8 | 60278648 | 60277689 | -1 | OR13C7L |
| ENSBTAG00000051473 | 8 | 60287928 | 60286972 | -1 | OR13C7B |
| ENSBTAG00000000252 | 8 | 1.03E+08 | 1.03E+08 | -1 | POLE3 |
| ENSBTAG00000009596 | 8 | 1.03E+08 | 1.03E+08 | 1 | C8H9orf43 |
| ENSBTAG00000048381 | 8 | 60294256 | 60293300 | -1 | OR13C7C |
| ENSBTAG00000043406 | 8 | 1.03E+08 | 1.03E+08 | -1 | SNORA72 |
| ENSBTAG00000049595 | 8 | 60307397 | 60306438 | 1 | OR13C7J |
| ENSBTAG00000043854 | 8 | 60312127 | 60311974 | 1 |  |
| ENSBTAG00000043153 | 8 | 60323967 | 60323842 | -1 |  |
| ENSBTAG00000000076 | 8 | 60410644 | 60326356 | 1 | RECK |
| ENSBTAG00000049739 | 8 | 60351107 | 60348744 | 1 |  |
| ENSBTAG00000052319 | 8 | 60358573 | 60358497 | 1 |  |
| ENSBTAG00000045397 | 8 | 60371650 | 60371532 | 1 |  |
| ENSBTAG00000054769 | 8 | 60378043 | 60377695 | 1 |  |
| ENSBTAG00000004499 | 8 | 1.03E+08 | 1.03E+08 | 1 | RGS3 |
| ENSBTAG00000000078 | 8 | 60442493 | 60421784 | 1 | GLIPR2 |
| ENSBTAG00000021696 | 8 | 60448238 | 60445847 | 1 | CCIN |
| ENSBTAG00000001137 | 8 | 60491846 | 60474074 | 1 | CLTA |
| ENSBTAG00000001142 | 8 | 60528253 | 60494277 | -1 | GNE |
| ENSBTAG00000043344 | 8 | 60550481 | 60550350 | 1 | SNORA70 |
| ENSBTAG00000050221 | 8 | 60559339 | 60558700 | 1 |  |
| ENSBTAG00000005519 | 8 | 60713864 | 60593718 | -1 | RNF38 |
| ENSBTAG00000025659 | 8 | 1.03E+08 | 1.03E+08 | 1 | ZNF618 |
| ENSBTAG00000015685 | 8 | 1.03E+08 | 1.03E+08 | -1 | KIF12 |
| ENSBTAG00000021686 | 8 | 60848414 | 60775331 | 1 | MELK |
| ENSBTAG00000052610 | 8 | 1.03E+08 | 1.03E+08 | 1 |  |
| ENSBTAG00000019807 | 8 | 1.04E+08 | 1.03E+08 | 1 | COL27A1 |
| ENSBTAG00000012498 | 8 | 61178197 | 60998035 | -1 | PAX5 |
| ENSBTAG00000025859 | 8 | 61488160 | 61215357 | 1 | ZCCHC7 |
| ENSBTAG00000054508 | 8 | 61540553 | 61508010 | 1 |  |
| ENSBTAG00000019299 | 8 | 61564033 | 61555133 | 1 | GRHPR |
| ENSBTAG00000029902 | 8 | 1.03E+08 | 1.03E+08 | 1 | MIR455 |
| ENSBTAG00000007761 | 8 | 61594251 | 61567467 | -1 | ZBTB5 |
| ENSBTAG00000049177 | 8 | 61581636 | 61581530 | 1 | U6 |
| ENSBTAG00000017294 | 8 | 1.04E+08 | 1.04E+08 | 1 | ORM1 |
| ENSBTAG00000012243 | 8 | 61626165 | 61610188 | 1 | POLR1E |
| ENSBTAG00000017306 | 8 | 1.04E+08 | 1.04E+08 | -1 | AKNA |
| ENSBTAG00000012262 | 8 | 61673542 | 61633982 | -1 | FBXO10 |
| ENSBTAG00000032777 | 8 | 61694543 | 61691238 | -1 | TOMM5 |
| ENSBTAG00000032777 | 8 | 61694543 | 61691238 | -1 | TOMM5 |
| ENSBTAG00000010107 | 8 | 1.04E+08 | 1.04E+08 | -1 | WHRN |
| ENSBTAG00000046574 | 8 | 61865028 | 61704750 | 1 | FRMPD1 |
| ENSBTAG00000055109 | 8 | 1.04E+08 | 1.04E+08 | -1 |  |
| ENSBTAG00000050529 | 8 | 1.04E+08 | 1.04E+08 | -1 |  |
| ENSBTAG00000000203 | 8 | 1.04E+08 | 1.04E+08 | 1 | ATP6V1G1 |
| ENSBTAG00000018922 | 8 | 61881692 | 61868932 | 1 | TRMT10B |
| ENSBTAG00000018925 | 8 | 61888055 | 61883371 | -1 | EXOSC3 |
| ENSBTAG00000050943 | 8 | 61909442 | 61901164 | 1 |  |
| ENSBTAG00000014934 | 8 | 61961976 | 61910844 | 1 | DCAF10 |
| ENSBTAG00000014936 | 8 | 61994835 | 61976292 | -1 | SLC25A51 |
| ENSBTAG00000000204 | 8 | 1.04E+08 | 1.04E+08 | 1 | TMEM268 |
| ENSBTAG00000018069 | 8 | 1.04E+08 | 1.04E+08 | -1 | TNFSF15 |
| ENSBTAG00000014922 | 8 | 62144584 | 62008686 | -1 | SHB |
| ENSBTAG00000044336 | 8 | 62092999 | 62092929 | -1 | bta-mir-2472 |
| ENSBTAG00000045380 | 8 | 62108991 | 62108920 | -1 | bta-mir-2473 |
| ENSBTAG00000044675 | 8 | 62142447 | 62142389 | -1 | bta-mir-2474 |
| ENSBTAG00000038411 | 8 | 62264824 | 62264267 | 1 |  |
| ENSBTAG00000025782 | 8 | 1.04E+08 | 1.04E+08 | -1 | TNFSF8 |
| ENSBTAG00000049826 | 8 | 1.04E+08 | 1.04E+08 | 1 |  |
| ENSBTAG00000051432 | 8 | 1.04E+08 | 1.04E+08 | -1 |  |
| ENSBTAG00000020385 | 8 | 62434231 | 62430631 | 1 | ALDH1B1 |
| ENSBTAG00000014078 | 8 | 62458755 | 62441823 | -1 | IGFBPL1 |
| ENSBTAG00000000575 | 8 | 1.04E+08 | 1.04E+08 | -1 | TNC |
| ENSBTAG00000040034 | 8 | 62522597 | 62476008 | 1 |  |
| ENSBTAG00000015378 | 8 | 62593188 | 62537300 | 1 | CCDC180 |
| ENSBTAG00000003719 | 8 | 62715815 | 62612938 | 1 | TDRD7 |
| ENSBTAG00000049761 | 8 | 62628956 | 62628416 | -1 |  |
| ENSBTAG00000014719 | 8 | 62812715 | 62722964 | 1 | TMOD1 |
| ENSBTAG00000014722 | 8 | 62841230 | 62813437 | -1 | TSTD2 |
| ENSBTAG00000009732 | 8 | 62878151 | 62840870 | 1 | NCBP1 |
| ENSBTAG00000009734 | 8 | 62908594 | 62878687 | -1 | XPA |
| ENSBTAG00000049409 | 8 | 63037905 | 63025993 | -1 |  |
| ENSBTAG00000055139 | 8 | 1.05E+08 | 1.05E+08 | -1 | U6 |
| ENSBTAG00000044523 | 8 | 1.05E+08 | 1.05E+08 | -1 | 5S_rRNA |
| ENSBTAG00000014760 | 8 | 63053678 | 63052557 | 1 | FOXE1 |
| ENSBTAG00000019238 | 8 | 63100080 | 63081995 | -1 | TRMO |
| ENSBTAG00000032206 | 8 | 63121637 | 63102557 | -1 | HEMGN |
| ENSBTAG00000021367 | 8 | 63179637 | 63155632 | 1 | ANP32B |
| ENSBTAG00000019023 | 8 | 63212682 | 63192882 | 1 | NANS |
| ENSBTAG00000024851 | 8 | 63241588 | 63216626 | -1 | TRIM14 |
| ENSBTAG00000007659 | 8 | 63310282 | 63246399 | -1 | CORO2A |
| ENSBTAG00000019550 | 8 | 63357767 | 63312769 | -1 | TBC1D2 |
| ENSBTAG00000004010 | 8 | 1.06E+08 | 1.05E+08 | 1 | PAPPA |
| ENSBTAG00000013810 | 8 | 63749799 | 63383029 | -1 | GABBR2 |
| ENSBTAG00000029115 | 8 | 63639673 | 63639555 | -1 | 5S_rRNA |
| ENSBTAG00000005133 | 8 | 63836341 | 63787511 | -1 | ANKS6 |
| ENSBTAG00000009037 | 8 | 63887891 | 63854829 | 1 | GALNT12 |
| ENSBTAG00000025667 | 8 | 1.07E+08 | 1.06E+08 | -1 | ASTN2 |
| ENSBTAG00000017155 | 8 | 1.06E+08 | 1.06E+08 | 1 | TRIM32 |
| ENSBTAG00000053221 | 8 | 1.07E+08 | 1.07E+08 | 1 |  |
| ENSBTAG00000006240 | 8 | 1.07E+08 | 1.07E+08 | 1 | TLR4 |
| ENSBTAG00000054122 | 8 | 1.08E+08 | 1.08E+08 | 1 | 5S_rRNA |
| ENSBTAG00000015936 | 8 | 1.09E+08 | 1.09E+08 | -1 | BRINP1 |
| ENSBTAG00000002294 | 9 | 49554234 | 49484923 | 1 | SIM1 |
| ENSBTAG00000014104 | 9 | 49838771 | 49838577 | 1 |  |
| ENSBTAG00000052778 | 9 | 49903697 | 49900892 | 1 |  |
| ENSBTAG00000049852 | 9 | 49906134 | 49904082 | -1 |  |
| ENSBTAG00000005449 | 9 | 49948405 | 49923127 | 1 | MCHR2 |
| ENSBTAG00000006095 | 9 | 50205058 | 50197319 | -1 | PRDM13 |
| ENSBTAG00000054078 | 9 | 50229848 | 50229597 | -1 |  |
| ENSBTAG00000042767 | 9 | 50231387 | 50231284 | -1 | U6 |
| ENSBTAG00000015744 | 9 | 50269733 | 50244002 | 1 | CCNC |
| ENSBTAG00000018528 | 9 | 50337074 | 50266970 | 1 | USP45 |
| ENSBTAG00000038931 | 9 | 50277915 | 50274372 | -1 | TSTD3 |
| ENSBTAG00000019730 | 9 | 50372811 | 50346225 | 1 | PNISR |
| ENSBTAG00000019729 | 9 | 50401243 | 50377200 | 1 | COQ3 |
| ENSBTAG00000044061 | 9 | 50488286 | 50419564 | 1 | FAXC |
| ENSBTAG00000005729 | 9 | 50885025 | 50809427 | 1 | FBXL4 |
| ENSBTAG00000024773 | 9 | 50922123 | 50920530 | -1 | POU3F2 |
| ENSBTAG00000053668 | 9 | 52376718 | 52376612 | -1 | U6 |
| ENSBTAG00000000629 | 9 | 52617857 | 52495548 | 1 | MMS22L |
| ENSBTAG00000005301 | 9 | 52797848 | 52625780 | -1 | KLHL32 |
| ENSBTAG00000045319 | 9 | 52666076 | 52665967 | 1 | U5 |
| ENSBTAG00000003174 | 9 | 52891447 | 52882686 | 1 | NDUFAF4 |
| ENSBTAG00000049129 | 9 | 52959636 | 52957058 | -1 |  |
| ENSBTAG00000002915 | 9 | 53022618 | 53021359 | 1 | GPR63 |
| ENSBTAG00000001803 | 9 | 53239319 | 53186120 | -1 | FHL5 |
| ENSBTAG00000020410 | 9 | 53314755 | 53248040 | -1 | UFL1 |
| ENSBTAG00000051626 | 9 | 53254975 | 53253965 | 1 |  |
| ENSBTAG00000044476 | 9 | 53434377 | 53434252 | -1 |  |
| ENSBTAG00000051581 | 9 | 53437324 | 53436932 | 1 |  |
| ENSBTAG00000007989 | 9 | 53646339 | 53645198 | -1 | FUT9 |
| ENSBTAG00000052032 | 9 | 53879543 | 53762297 | -1 |  |
| ENSBTAG00000051787 | 9 | 54200888 | 54200721 | -1 |  |
| ENSBTAG00000005026 | 9 | 54379111 | 54296791 | -1 | MANEA |
| ENSBTAG00000036978 | 9 | 54451594 | 54451476 | -1 | 5S_rRNA |
| ENSBTAG00000043941 | 9 | 55089993 | 55089680 | -1 | 7SK |
| ENSBTAG00000033083 | 9 | 55495746 | 55495443 | 1 |  |
| ENSBTAG00000053795 | 9 | 55800082 | 55799976 | 1 | U6 |
| ENSBTAG00000003743 | 9 | 56645654 | 56643939 | 1 |  |
| ENSBTAG00000021420 | 9 | 56958827 | 56751222 | 1 | EPHA7 |
| ENSBTAG00000055091 | 9 | 57575431 | 57574563 | -1 |  |
| ENSBTAG00000049952 | 9 | 58741713 | 58569557 | 1 |  |
| ENSBTAG00000043746 | 9 | 58616750 | 58616428 | 1 | 7SK |
| ENSBTAG00000002625 | 9 | 59744280 | 59679581 | 1 | MAP3K7 |
| ENSBTAG00000020713 | 9 | 60341240 | 60047260 | 1 | BACH2 |
| ENSBTAG00000011315 | 9 | 60360012 | 60351728 | -1 | GJA10 |
| ENSBTAG00000011313 | 9 | 60403669 | 60369226 | -1 | CASP8AP2 |
| ENSBTAG00000042260 | 9 | 60382739 | 60382675 | 1 | U7 |
| ENSBTAG00000001644 | 9 | 60550024 | 60410450 | 1 | MDN1 |
| ENSBTAG00000026604 | 9 | 60555713 | 60553611 | 1 | LYRM2 |
| ENSBTAG00000003455 | 9 | 60742901 | 60560040 | -1 | ANKRD6 |
| ENSBTAG00000053008 | 9 | 60574953 | 60574891 | -1 | bta-mir-7862 |
| ENSBTAG00000044854 | 9 | 60708608 | 60708504 | 1 | U6 |
| ENSBTAG00000050934 | 9 | 60754445 | 60753191 | 1 |  |
| ENSBTAG00000018438 | 9 | 60800465 | 60762595 | 1 | RRAGD |
| ENSBTAG00000018436 | 9 | 60836538 | 60811836 | 1 | UBE2J1 |
| ENSBTAG00000047037 | 9 | 60813013 | 60812926 | 1 | bta-mir-2903 |
| ENSBTAG00000011672 | 9 | 60946360 | 60845210 | 1 | GABRR1 |
| ENSBTAG00000044651 | 9 | 60893499 | 60893409 | -1 | U6 |
| ENSBTAG00000011667 | 9 | 60971066 | 60958094 | -1 | PM20D2 |
| ENSBTAG00000049831 | 9 | 60966420 | 60965053 | 1 |  |
| ENSBTAG00000046235 | 9 | 61016030 | 60999045 | 1 | SRSF12 |
| ENSBTAG00000019110 | 9 | 61035355 | 61031143 | -1 | PNRC1 |
| ENSBTAG00000002725 | 9 | 61335342 | 61102065 | 1 | RNGTT |
| ENSBTAG00000015335 | 9 | 8247107 | 7702860 | 1 | ADGRB3 |
| ENSBTAG00000054672 | 9 | 8483031 | 8476145 | -1 |  |
| ENSBTAG00000016164 | 9 | 8739663 | 8607136 | -1 | LMBRD1 |
| ENSBTAG00000035054 | 9 | 9319960 | 9301759 | -1 | COL9A1 |
| ENSBTAG00000045999 | 9 | 9483137 | 9482757 | 1 |  |
| ENSBTAG00000052350 | 9 | 9495291 | 9493608 | -1 |  |
| ENSBTAG00000043707 | 9 | 9502632 | 9502509 | 1 | U4 |
| ENSBTAG00000002171 | 9 | 9672872 | 9517906 | 1 | FAM135A |
| ENSBTAG00000027049 | 9 | 9726933 | 9683242 | 1 | SDHAF4 |
| ENSBTAG00000038190 | 9 | 9974224 | 9785197 | 1 | SMAP1 |
| ENSBTAG00000020817 | 9 | 10070666 | 9970661 | -1 | B3GAT2 |
| ENSBTAG00000053784 | 9 | 10343943 | 10342907 | -1 |  |
| ENSBTAG00000050836 | 9 | 10364290 | 10364232 | -1 | bta-mir-2285cx |
| ENSBTAG00000016374 | 9 | 10413050 | 10397908 | 1 | OGFRL1 |
| ENSBTAG00000029836 | 9 | 10494902 | 10494819 | -1 | bta-mir-30f |
| ENSBTAG00000029804 | 9 | 10532237 | 10532171 | -1 | bta-mir-30a |
| ENSBTAG00000048046 | 9 | 10633051 | 10632692 | 1 |  |
| ENSBTAG00000020238 | 9 | 11695382 | 11090230 | 1 | RIMS1 |
| ENSBTAG00000007791 | 9 | 12648301 | 12429180 | 1 | KCNQ5 |
| ENSBTAG00000034925 | 9 | 12784654 | 12753066 | 1 | KHDC3L |
| ENSBTAG00000019600 | 9 | 12766533 | 12765347 | -1 | OOEP |
| ENSBTAG00000021814 | 9 | 12802327 | 12786479 | 1 |  |
| ENSBTAG00000000892 | 9 | 12958092 | 12934833 | -1 | CGAS |
| ENSBTAG00000016839 | 9 | 12990379 | 12969537 | 1 | MTO1 |
| ENSBTAG00000014534 | 9 | 13020524 | 13011231 | -1 | EEF1A1 |
| ENSBTAG00000044053 | 9 | 13161408 | 13086269 | -1 | SLC17A5 |
| ENSBTAG00000043542 | 9 | 13129871 | 13129769 | 1 | U6 |
| ENSBTAG00000013222 | 9 | 13341118 | 13201927 | 1 | CD109 |
| ENSBTAG00000042148 | 9 | 13425644 | 13425540 | -1 | U6 |
| ENSBTAG00000054709 | 9 | 13659405 | 13658642 | -1 |  |
